# Archival and palaeoenvironmental documentation of extreme climate events reveals generational amnesia in collective memory

**DOI:** 10.1038/s41467-026-78922-x

**Published:** 2026-11-09

**Authors:** Shuang Song, Bo Hu, David Max Findley, Xutong Wu, Wei Liu, Robert W. Middeke-Conlin, Victor L. Caetano-Andrade, Yuda Yang, Shuai Wang, Jürgen Renn, Patrick Roberts

**Affiliations:** 1 Department of Co-evolution of Land Use and Urbanisation, Max Planck Institute of Geoanthropology, Jena, Germany; 2 Department Structural Changes of the Technosphere, Max Planck Institute of Geoanthropology, Jena, Germany; 3 State Key Laboratory of Earth Surface Processes and Disaster Risk Reduction, Faculty of Geographical Science, Beijing Normal University, Beijing, China; 4 School of Social and Behavioral Sciences, Nanjing University, Nanjing, China; 5 Institute of Chinese Historical Geography, Fudan University, Shanghai, China; 6 Department of Atmospheric and Oceanic Sciences, Fudan University, Shanghai, China; 7 Research Group ASTRA, Max Planck Institute for the History of Science, Berlin, Germany; 8 Institut für Orientalistik, Indogermanistik, Ur- und Frühgeschichtliche Archäologie, Friedrich Schiller Universität, Jena, Germany

**Keywords:** Climate change, Climate-change adaptation, Human behaviour

## Abstract

Collective memory shapes how societies respond to extreme climate events, yet long-term evidence on how the collective memory of such events becomes perceptually biased over time is lacking. Here, we utilise four centuries (1470–1900) of climatic archives from northern China, along with tree–ring–based hydroclimate reconstructions, which are validated against instrumental observations of Wet/Dry Index (1901–2000) to fill the gap. We find that historical records reveal systematic, non-random mismatches, where severity assessments are often biased toward recent experience rather than absolute climatic anomalies. Correlations between historical and natural records increase when the natural series is re-standardised using 20–40-year sliding windows, reflecting a generational offset. A heuristic agent-based model reproduces this pattern, suggesting that generational amnesia is sufficient to explain the offsets in perception of environmental change. We argue that historical archives carry a generational imprint of perceptual bias, in which the severity of climate extremes is judged against recent lived experience rather than long-term climate variability. This has implications for bias-aware corrections for risk assessment, adaptation, and paleoclimate reconstruction.

## Abstract (web summary)

Collective memory shapes how societies respond to climate extremes, but its long-term biases are hard to observe. Here, the authors show that historical severity assessments of droughts and floods in northern China over 430 years are biased toward recent experience, consistent with an agent-based model of generational amnesia.

## Introduction

Collective memory, defined as the storage, retrieval, and forgetting of information by a society^1,2^, interacts with diverse cultural, economic, and political contexts^3^ to shape how societies experience, document, and respond to extreme events^4,5^. Given its role in fostering cooperative and adaptive responses to climatic change^6^, collective memory should be treated as a central component of efforts to confront environmental risks in the 21st century^7^. This is especially true in highly active fluvial environments, where experiences of, and responses to, drought and flooding are dependent on how collective memory shapes individual perceptions and adaptations^8,9^. To model contemporary and future human-Earth system interactions in the Anthropocene, it is essential to incorporate the slow variable^10^ of collective memory and examine its relationship to human perceptions. To do so effectively, however, we must turn our attention to this phenomenon in the past for long-term analysis, which offers an opportunity to observe the variable’s effects and interactions across different contexts and time-scales.

Historical documents, inscriptions, monuments, and folk stories are the most common expressions and sources of collective memory (also known as cultural memory, a type of social representation within collective memory)^11^. For instance, traditional knowledge systems include legends and stories about floods, droughts, and many other hazards^12^. They can provide lessons from history but were also sometimes distorted (e.g., mixed with fiction or exaggerated) ^13^. On the other hand, collective memory influences the long-term adaptations of human communities to changing environments ^14^. Societies that have experienced extreme events, for example, may choose not to reside in areas with climate risks ^5^ or gradually develop adaptive responses to similar extreme events^15^. When the reservoirs of knowledge constructed by collective memory fade, societies may forget past catastrophic events^16^ and return to high-risk areas ^5^ (e.g., the levee effect in fluvial contexts ^17,18^). Understanding the relationship between collective memory and environmental extremes can thus play an important role in long-term social resilience and adaptation to hazards ^19^ (i.e., remembering as a form of resilience ^9^). In this context, care must be taken to address potential bias in historical archives to build resilience by learning from the past.

**Fig. 1:**
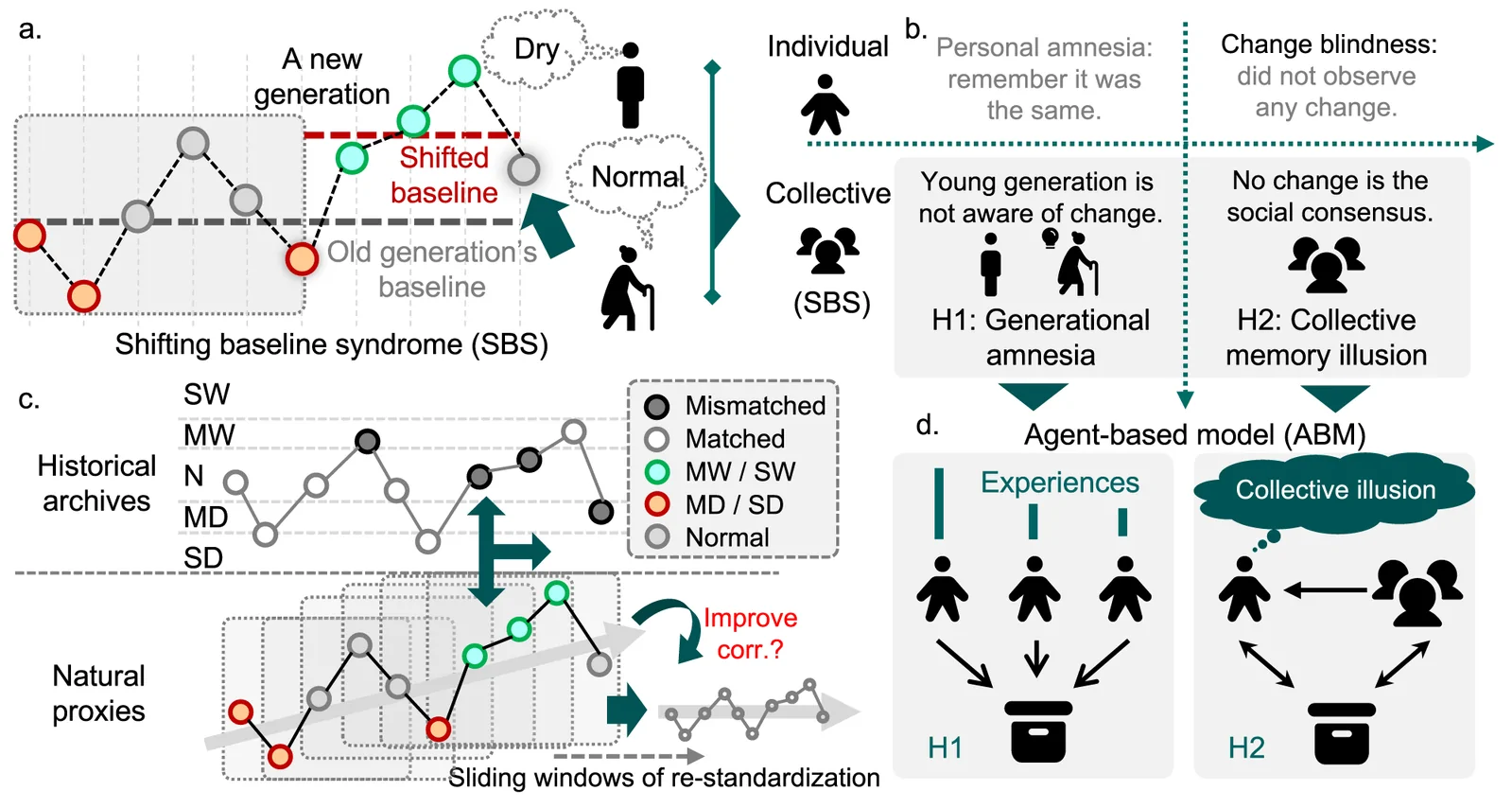
Study design. **a** Shifting Baseline Syndrome (SBS): each generation grows up in a different environment and adopts a different reference baseline; in a climate that becomes wetter, a relatively dry year appears normal to earlier generations, whereas a normal year appears dry to later ones. **b** Two collective sources of perceptual bias proposed by SBS theory: differences in lived experience across generations (H1: generational amnesia) and a shared, misleading consensus about the past environment (H2: collective memory illusion). **c** The two Wet/Dry Index (WDI) series, from historical archives and natural proxies, are each classified by z-score into five levels: Severe Wet (SW), Moderate Wet (MW), Normal (N), Moderate Dry (MD) and Severe Dry (SD) (Supplementary Information Fig. S3). We quantify their mismatches and test whether re-standardising the natural series within sliding windows, that is, against a moving baseline, increases their correlation ('Methods'). **d** An agent-based model tests whether agents that judge the climate against their own experience (H1) or against a shared archive (H2) reproduce the observed generational offset. Icons adapted from Phosphor Icons (person, group of three people and light bulb; ⓒ 2023 Phosphor Icons, MIT License), Tabler Icons (archive box; ⓒ 2020–2026 Paweł Kuna, MIT License) and Google Material Symbols (young person and elderly woman; ⓒ Google LLC, Apache License 2.0).

Distinct from natural records of climate (such as tree rings, sedimentary proxies, and isotope records), human-produced representations of history usually reflect the contemporaneous society’s perceptions of climatic extremes ^20^. The key to testing the interplay between collective memory and climate adaptation is thus to identify patterns in long-term human perceptions of climate from historical (or other long-term) archives^21^. Research has suggested that people’s perceptual biases, such as attention bias, recall bias, and social appraisal bias, are associated with differences in views and actions on climate change across groups ^22,23^. Among the many perceptual biases, Shifting Baseline Syndrome (SBS)^24^, also referred to as Environmental Generations Amnesia (Fig. 1a) ^25^, suggests that the decayed memory of environmental changes across generations arises from comparisons to a shifting baseline. The baseline serves as a reference point for comparison and defines what is perceived as normal, based on limited memory and experience^25^, and is accordingly associated with cross-generational inconsistencies in environmental perception and with a weakened potential for collective action ^6,26^. This inconsistency in collective memory may stem from multiple sources, but collective factors, including generational amnesia or collective illusion, have been suggested as the main roots of SBS (Fig. 1b)^25^. Generational amnesia occurs when younger generations lack knowledge of past environmental conditions, whereas collective memory illusion arises when shared narratives or expectations distort how past conditions are remembered^25^. A recent study suggests that SBS is widespread across diverse contexts^27^. Nevertheless, long-term evidence remains lacking on whether and how SBS shapes collective memory of environmental change.

We aim to address this by comparing centuries of historical archive data on extreme events with palaeoclimate proxies from the same time period. We select the Yellow River Basin in North China as our case study (see Supplementary Information Fig. S1) for two reasons. Firstly, it possesses an abundance of archival records, detailing a region where urban settlement, writing traditions, and climatic impacts on hydrological systems have constantly interacted^28^. Secondly, the climate of this region is dominated by the Asian monsoon, resulting in distinct seasons and clearly defined dry and wet summer extremes^29^, which are also closely linked to the hydrological conditions of the Yellow River^30^. We compare pre-instrumental climatic extremes (Wet/Dry Index, WDI), compiled from historical archives between 1470 and 1900 CE (hereafter the historical-archive WDI, H-WDI), to non-anthropogenic proxy indicators from tree-ring analyses (the natural-proxy WDI, N-WDI), which provide a similar annual (sometimes intra-annual) resolution. Both series are interpreted reconstructions. The H-WDI is mediated by contemporaneous human perception and is therefore comparatively more exposed to perceptual bias, whereas the N-WDI, though itself shaped by sampling and methodological choices, does not pass through human perception and thus provides a relatively independent reference. Differences and correlations between the two thus allow us to identify signatures of perceptual bias in collective memory. Therefore, we test two hypotheses: (1) historical archives of climatic extremes show signatures consistent with contemporaneous perceptual bias and (2) this perceptual bias is associated with a generational offset in collective memory relative to natural observations of climatic variation. We validate the N-WDI against instrumental data for 1901–2000 CE, quantify the matches, mismatches and correlation between the two WDIs (Fig. 1c), test whether re-standardising the natural series within sliding windows, which corrects for generational differences in the baseline, increases their agreement, and use a heuristic agent-based model (ABM) to compare the two hypotheses (Fig. 1d).

Here, we show that mismatches between archival and natural records of climatic extremes in the Yellow River Basin follow a systematic pattern: archival severity is over- or underestimated when a year is only slightly wetter or drier than comparable earlier years. Re-standardising the natural series within sliding windows of 20–40 years, roughly one human generation, increases the correlation between the two records, and the ABM reproduces this generational optimum when agents judge climate against their own lived experience (generational amnesia) but not against a shared archive (collective memory illusion). These results indicate that historical archives carry a generational-scale perceptual imprint relevant to how past societies recorded, experienced and adapted to climatic extremes.

## Results

### Reconstruction and validation of the WDI datasets

**Fig. 2:**
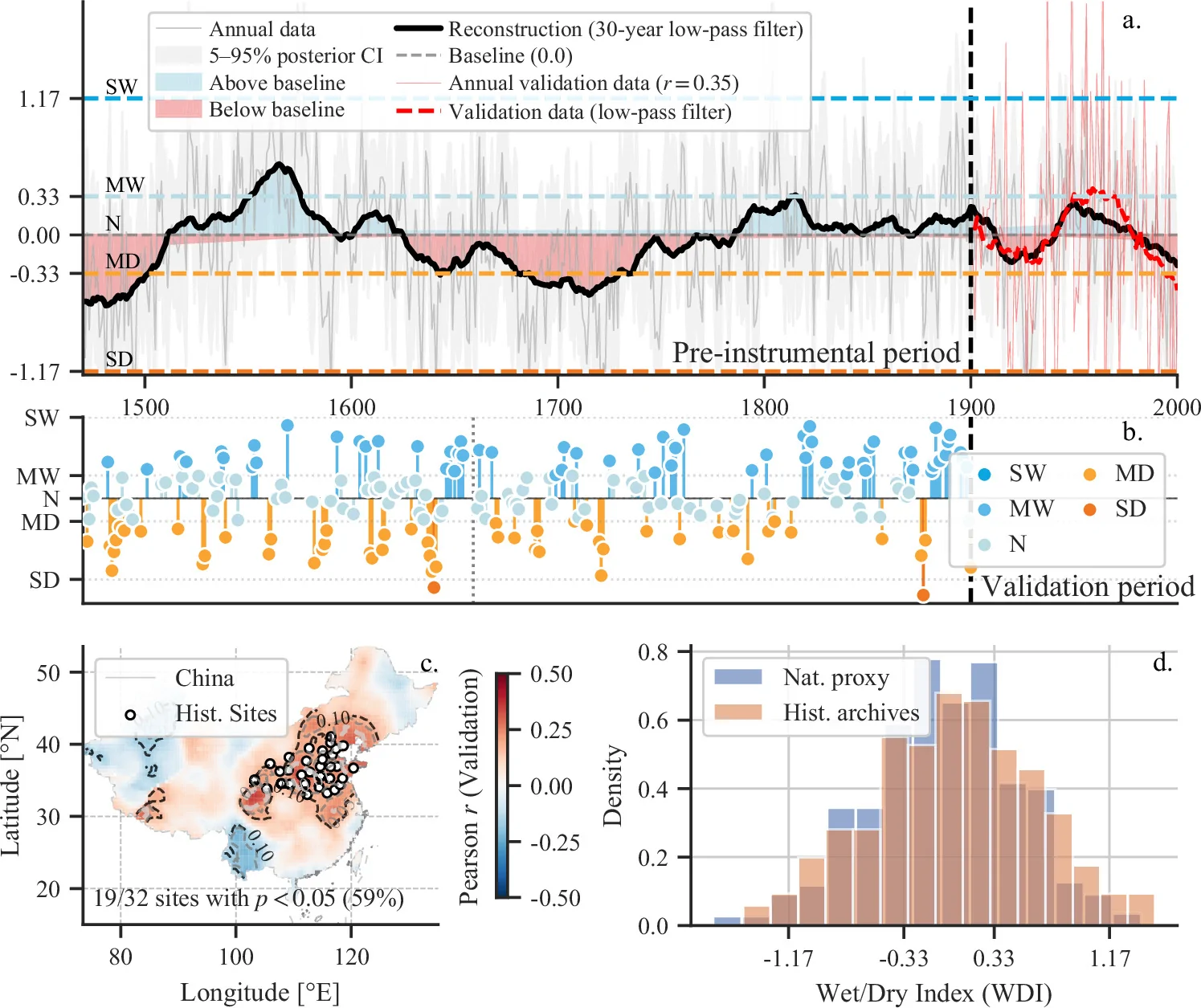
Reconstruction and validation of the WDI datasets. **a** Natural-proxy WDI (N-WDI) integrated from tree-ring reconstructions (Methods; Supplementary Note 2). Thin grey line, annual posterior mean; black line, 30-year low-pass filter; grey band, posterior mean  ± 1.645 posterior standard deviations (approximate 90% credible interval). Blue and red shading mark years above and below the baseline (z-score = 0); dashed lines mark the thresholds between the five WDI levels (SD, MD, N, MW, SW). Over the validation period (1901–2000 CE), thin and thick red lines show the annual and 30-year low-pass instrumental series (*r* = 0.35). **b** Regional historical-archive WDI (H-WDI), the mean of up to 32 site records per year, coloured by level from orange (dry) to blue (wet). **c** Pearson correlation between the N-WDI and the gridded instrumental data over the validation period (*n* = 100 years). Circles mark the 32 archive sites, 19 of which are significant at *p* < 0.05 (two-sided test at the nearest grid cell, not adjusted for multiple comparisons; exact *r* and *p* in Supplementary Information Table S2). Dashed contours mark two-sided *p* = 0.10 (black), 0.05 (grey) and 0.01 (light grey). The national boundary is reproduced without modification from the Standard Map Service of the Ministry of Natural Resources of China (approval number GS(2020)4619, http://bzdt.ch.mnr.gov.cn). d. Distributions of the N-WDI (*n* = 431 years) and H-WDI (n = 208 years) over the pre-instrumental period (two-sided Shapiro–Wilk test: N-WDI *W* = 0.998, *p* = 0.812; H-WDI *W* = 0.982, *p* = 0.008).

We collected and integrated multiple reconstructed hydroclimate datasets (see Methods and Supplementary Note 2 for more details) of summer (July-August-September) precipitation and Yellow River runoff based on natural proxies into N-WDI. For the validation period (1901–2000 CE), during which abundant instrumental measurements were available, the integrated N-WDI series was tested against three different summer precipitation datasets (see Methods and Supplementary Note 2 for more details). The year-to-year Pearson correlation coefficient between the N-WDI and the instrumental validation data is *r*(98) = 0.35 (two-sided *p* < 0.001; 95% confidence interval 0.16–0.51; *n* = 100 years; both series consistent with normality, see Methods), which is similar to the collected natural proxy datasets (see Supplementary Information Table S8 and Fig. 2a). The instrumental validation series (thin red line shown only for 1901–2000 CE) has a greater amplitude than the N-WDI, which reflects the variance attenuation inherent in tree-ring reconstructions (calibration regression damps high-frequency variance, retaining only the fraction explained). Under a 30-year low-pass filter the N-WDI closely tracks the instrumentally-based WDI over the validation period (Fig. 2a) and follows the same wet/dry phases as the H-WDI over the pre-instrumental period (1470–1900 CE, Fig. 2b; see 'Methods'). Spatially, 19 of the 32 historical-archive sites we used were located in areas that showed a significant positive correlation with the N-WDI series (two-sided test of Pearson’s *r* at the nearest grid cell, *n* = 100 years, not adjusted for multiple comparisons; site locations are listed in Supplementary Information Table S2; Fig. 2c; other validation datasets in Supplementary Information Fig. S4). The H-WDI was inferred from historical archives across all sites (see Supplementary Note 1), and the good spatial-temporal correlations indicate the reliability of our data aggregation. Alternative combinations and estimation methods of the historical-archive WDI, and alternative selections of natural-proxy datasets, yield qualitatively similar results (see Supplementary Note 2), supporting methodological robustness. We also verified that the two reconstructed WDI datasets during the pre-instrumental period have approximately symmetrical distributions (skewness −0.09 for the N-WDI, *n* = 431 years; −0.28 for the H-WDI, *n* = 208 years). A two-sided Shapiro–Wilk test did not reject normality for the N-WDI (*W* = 0.998, *p* = 0.812) but did for the H-WDI (*W* = 0.982, *p* = 0.008, Fig. 2d). Based on these observations, the two series (the perception-mediated H-WDI and the more independent, non-perceptual N-WDI) provide methodologically independent representations of the same climatic anomalies and can be compared.

### Mismatch of levels between N-WDI and H-WDI

**Fig. 3:**
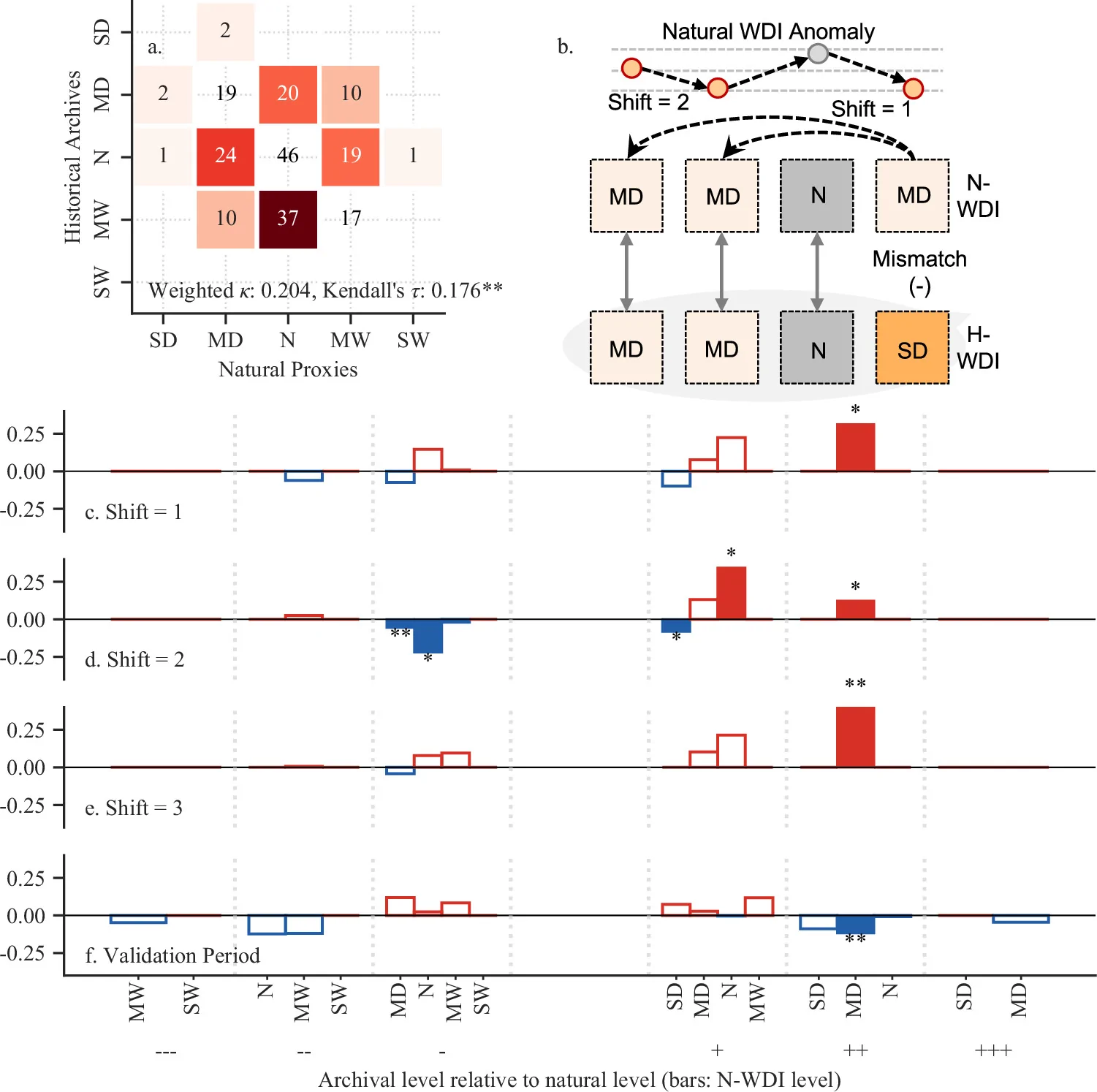
Mismatches between N-WDI and H-WDI levels. **a** Confusion matrix of the five WDI levels from natural proxies and historical archives (diagonal, matches; off-diagonal, mismatches; *n* = 208 years), annotated with Cohen’s weighted *κ* and Kendall’s *τ* (two-sided *p* = 0.004). **b** Shifted comparison: each mismatched archival record (orange box) is compared with the shift-th earlier year in which the N-WDI had the same level (curved dashed arrows), to test whether the misclassification follows the change in N-WDI anomaly between the 2 years. **c**–**e** Mean offset of the N-WDI z-score between the current and the earlier year (y-axis) for each type of archival misclassification (x-axis: groups give how many levels wetter (+) or drier (-) the archival level is; bars are labelled with the N-WDI level) at shift = 1, 2 and 3. Significance was assessed by a two-sided Monte Carlo randomisation test (10,000 iterations; Methods), not adjusted for multiple comparisons; filled bars mark *p* < 0.05 (* *p* < 0.05; ** *p* < 0.01). Exact *p* values (natural to archival level; *n*, mismatched pairs): **c** MD to MW, *p* = 0.028 (n = 10); **d** MD to SD, *p* = 0.0096 (*n* = 2); SD to MD, *p* = 0.012 (*n* = 1); N to MD, *p* = 0.019 (*n* = 19); MD to MW, *p* = 0.037 (*n* = 10); N to MW, *p* = 0.047 (*n* = 37); **e** MD to MW, *p* = 0.002 (*n* = 10). **f** The same analysis for the reconstructed versus instrumental N-WDI over the validation period (1901–2000 CE, *n* = 100 years, shift = 1); the only cell with *p* < 0.05 (MD to MW, *n* = 1 pair, *p* < 0.001) has the opposite sign (Supplementary Information Fig. S11).

During the pre-instrumental period (1470–1900 CE), the categorised H-WDI shows modest but significant agreement with the categorised N-WDI (Cohen’s weighted *κ* = 0.2, 95% bootstrap confidence interval 0.07–0.33, two-sided permutation *p* = 0.004; classification accuracy = 0.39), with a slight but significant positive rank correlation between the two categorical series (Kendall’s *τ* = 0.176 for H-WDI versus N-WDI, 95% bootstrap confidence interval 0.054–0.293, two-sided *p* = 0.004; *n* = 208 years; Fig. 3a). This level of agreement is lower than, but of the same order as, that achieved by the proxy itself against independent instruments over the validation period (1901–2000 CE), where the N-WDI and the instrumental series agree at Kendall’s *τ* = 0.214 (95% bootstrap confidence interval 0.063–0.361, two-sided *p* = 0.012; Cohen’s weighted *κ* = 0.24, 95% bootstrap confidence interval 0.08–0.38, two-sided permutation *p* = 0.009; *n* = 100 years; see Supplementary Note 4 and Fig. S11), indicating that the H-WDI/N-WDI disagreement may reflect both uncertainties of the proxy and misaligned historical records. There are 126 mismatching data points between the N-WDI and H-WDI, which mainly occur between adjacent levels of severity categorisation (i.e., Moderate Dry/Wet to Normal and vice versa, Fig. 3a; their distribution over time is shown in Supplementary Note 4 and Fig.e S10). To further explore the process of comparing to the past implied by the SBS theory, we compared each mismatched H-WDI data with a similar prior event which occurred once, twice, or three times (shift = 1, 2, 3, respectively) in the past (Fig. 3b). In these mismatch cases, the H-WDI is usually categorised as drier (or wetter) than N-WDI when the z-score values of N-WDI are only slightly lower (or higher) compared to a former year. This shifted-correlation pattern is most apparent when shift = 2 (Fig. 3c.-e.). This means that the H-WDI systematically overestimated climate events when they were only slightly drier or wetter than similar events that occurred two times earlier, according to the N-WDI. This indicates a discernible directional pattern: a positive difference between the current and last data points is associated with an overestimation of the severity level, and a negative difference is associated with an underestimation. This shifted-correlation pattern in the historical archives exceeds that expected under a Monte Carlo Randomisation Null (10,000 iterations; see 'Methods'), in which archival severity categories are independently redrawn at fixed marginal frequencies. Five of the eight tested cells reached two-sided significance (*p* < 0.05) at shift = 2 (exact *p* = 0.0096, 0.012, 0.019, 0.037 and 0.047, not adjusted for multiple comparisons), compared with one cell at shift = 1 (*p* = 0.028) and one at shift = 3 (*p* = 0.002). Between the reconstructed and the instrumental N-WDI during the validation period (from 1901 to 2000 CE), only one cell reached *p* < 0.05 at shift = 1 (Moderate Dry to Moderate Wet, *n* = 1 pair, two-sided *p* < 0.001), and its offset has the opposite sign to the pattern in the historical archives (Fig. 3f and also Supplementary Information Fig. S11).

### Restandardisation in sliding windows increases the correlation

**Fig. 4:**
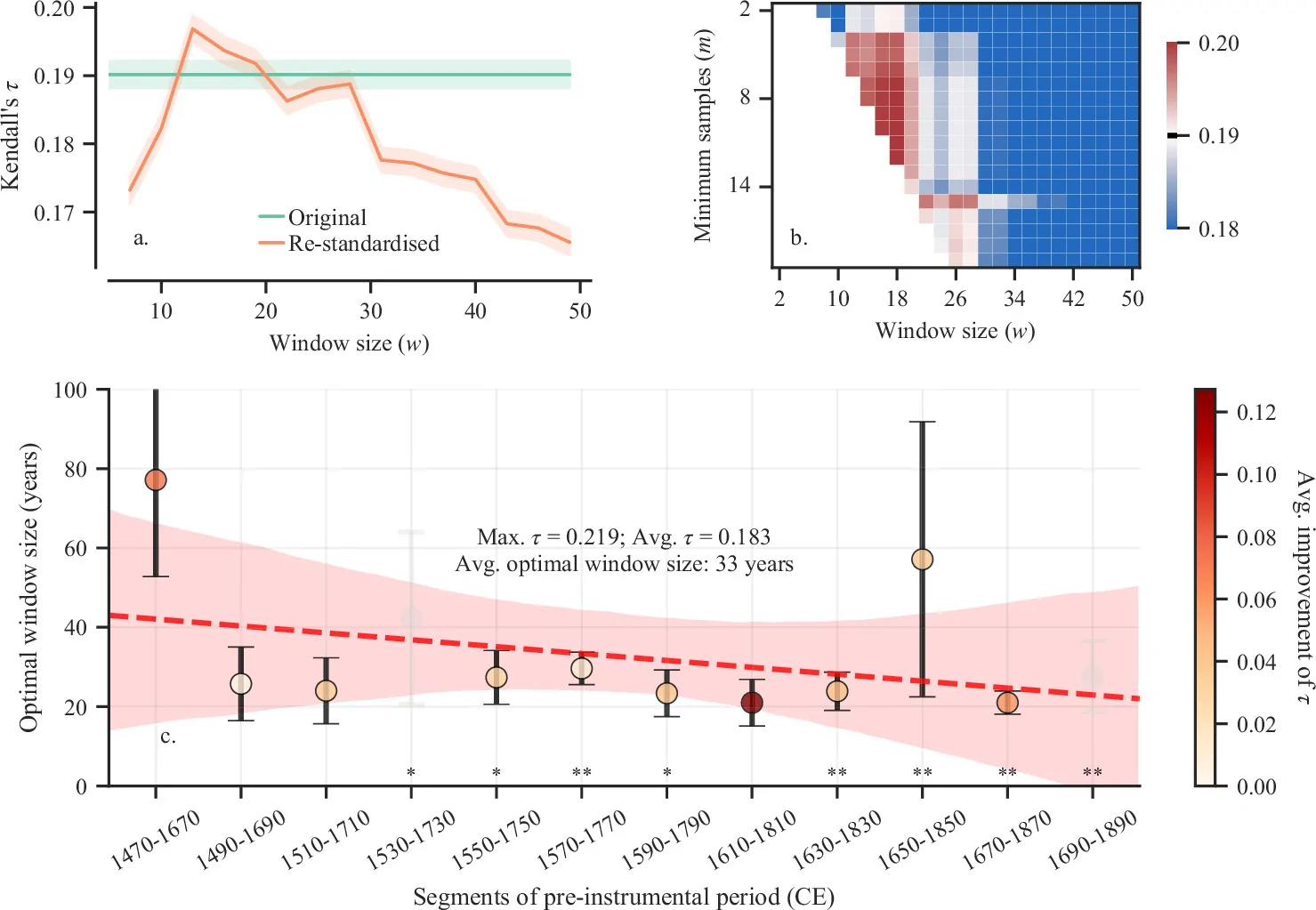
Correlation between the N-WDI and H-WDI under sliding-window re-standardisation. **a** Kendall’s *τ* between the H-WDI and the N-WDI with (Re-standardised) and without (Original) re-standardisation of the N-WDI, by window size *w*. Lines, mean of *n* = 100 stochastic realisations of the H-WDI (*n* = 208 years, 205 after re-standardisation); bands, 95% bootstrap confidence interval of the mean (1000 resamples). **b** Kendall’s *τ* across window sizes *w* and minimum sample sizes *m* relative to the whole-period benchmark (*τ* = 0.190, two-sided *p* < 0.001, *n* = 208 years): red, increase; blue, decrease; blank, *m* > *w*. All plotted cells are significant (two-sided *p* ≤ 0.004); n per cell is the 208 years minus those whose window holds fewer than *m* values (197 to 207 years). **c** Optimal window size in 200-year segments starting every 20 years from 1470 CE. Points and error bars, mean and standard deviation of the windows with the top 10% of *τ* in each segment (*n* = 8 windows); colour, mean improvement of *τ*; transparent points, no improvement; red dashed line and shading, linear trend with its 95% bootstrap confidence interval. Max. *τ* and Avg. *τ*, segment means of the maximum re-standardised and the benchmark *τ*. Asterisks mark segments with a significant benchmark *τ* (two-sided, not adjusted for multiple comparisons; * *p* < 0.05, ** *p* < 0.01): 1530–1730, *p* = 0.013 (*n* = 100 years); 1550–1750, *p* = 0.027 (*n* = 98); 1570–1770, *p* = 0.005 (*n* = 98); 1590–1790, *p* = 0.046 (*n* = 99); 1630–1830, *p* = 0.005 (*n* = 93); 1650–1850, *p* = 0.004 (*n* = 90); 1670–1870, *p* = 0.005 (*n* = 90); 1690–1890, *p* = 0.004 (*n* = 92); not statistically significant: 1470–1670, 1490–1690, 1510–1710 and 1610–1810, *p* = 0.098, 0.112, 0.093 and 0.088 (*n* = 101, 99, 103 and 97).

By applying restandardisation to sliding windows, the correlation between historical and natural data increases and the function of Kendall’s *τ* to *w* exhibits a unimodal pattern of correlation between restandardised N-WDI and H-WDI (Fig. 4a). We tested combinations of two key parameters (window size *w* and required minimum samples *m*) for the sliding window, and the results indicate that the unimodal pattern of optimal sliding window size *w* keeps existing in a specific range (Fig. 4b). Compared to the benchmark scenario without restandardisation (the whole-period Kendall’s *τ* = 0.19 between the H-WDI and the N-WDI before any re-standardisation; two-sided *p* < 0.001, n = 208 years), a greater correlation gradually emerges as the tested window size *w* increased to around 18 years, from 1470 to 1900 CE (maximum increased by 5.37%, Fig. 4b). Across the different proxy-integration and validation scenarios we tested, the correlation improvements from restandardisation persist regardless of the method used to combine the composite datasets of N-WDI (see Supplementary Note 2, Figure S5 and Table S6). Comparing the  restandardisation against a changing (sliding) normal baseline, the improved correlation is consistent with a shifting-baseline interpretation of the historical archives. Moreover, the increased correlations diminish below the baseline when expanding the window *w*. To test robustness over time, we examined each two-century period during the pre-instrumental period (Fig. 4c; see 'Methods'). The key sliding windows during which restandardisation outperformance appears are generally between 20 and 40 years. Specifically, the optimal window size, averaged across temporal segments, is 33 years, yielding a maximum increase in correlation of 19.65% (Fig. 4c). The range of this optimal window is similar to the reported population’s life expectancy during the study periods (Supplementary Information Fig. S6).

### ABM based on generational amnesia reproduces the unimodal pattern

**Fig. 5:**
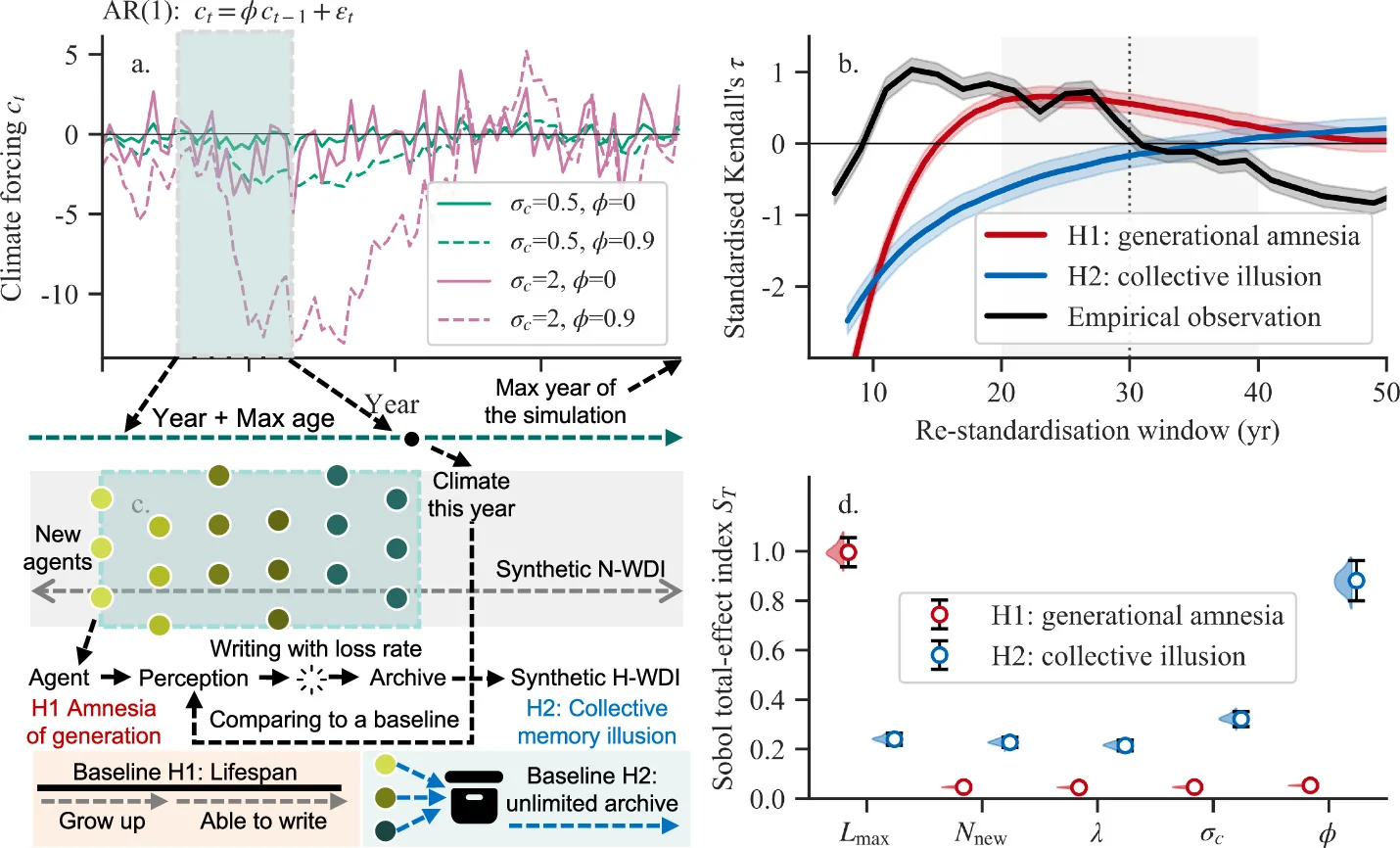
The agent-based model (ABM) and its results. **a** Annual climate forcing *c*_*t*_ generated by an AR(1) process (*c*_*t*_ = *ϕ* *c*_*t*−1_ + *ε*_*t*_), shown for the four corners of the Sobol parameter range (colour, climate variability *σ*_*c*_; line style, climate persistence *ϕ*); independent and linear-trend forcings are tested in Supplementary Note 3. The shaded box marks the lifespan of one agent cohort, detailed in (**c**). **b** Kendall’s *τ* between the simulated archive (synthetic H-WDI) and the climate forcing (synthetic N-WDI) as a function of the re-standardisation window under H1 (generational amnesia, red) and H2 (collective memory illusion, blue), compared with the empirical H-WDI versus N-WDI curve (black). Each curve is standardised by its own mean and standard deviation across windows so that their shapes can be compared. Lines, mean of *n* = 100 model runs per scenario (empirical curve: *n* = 100 stochastic realisations of the H-WDI, excluding correlations with *p*≥0.05); bands, 95% bootstrap confidence interval of the mean (1000 resamples). Grey shading, 20–40-year generational band; dotted line, 30 years. **c** Model structure. New agents enter every year, can write records from adulthood until their maximum lifespan, perceive each year’s climate relative to a baseline, and may write it into a shared archive that loses records at a fixed rate. The baseline is the agent’s own lived experience under H1 and the whole shared archive under H2. **d** Sobol total-effect index *S*_*T*_ of the location of the optimal window for lifespan ${L}_{\max }$, new agents per year *N*_new_, archive loss rate *λ*, climate variability *σ*_*c*_ and climate persistence *ϕ*. Half-violins, distribution of the *n* = 100 bootstrap estimates of *S*_*T*_; open circles and error bars, the estimate and its 95% bootstrap confidence interval; *N* = 1024 base samples per scenario (7168 parameter sets, each averaged over 30 stochastic runs). The archive-box icon in (**c**) is adapted from Tabler Icons (ⓒ 2020–2026 Paweł Kuna, MIT License).

The heuristic ABM represents climate as a stationary AR(1) stochastic process (*c*_*t*_ = *ϕ* *c*_*t*−1_ + *ε*_*t*_, Fig. 5a; alternative independent and trended forcings are tested for robustness in Supplementary Note 3 and Fig. S9) observed by a continuously turning-over population of finite-lifespan agents. Each agent judges the current anomaly against a baseline, and is more likely to log an event the more it departs from that baseline (Fig. 5c). We designed scenarios to represent the baseline, based on lived experience or shared archives of agents, corresponding to the two hypotheses of H1 (generational amnesia) and H2 (collective illusion) (Fig. 5c), with two other scenarios tested for robustness (see Supplementary Note 3, Table S11 and Fig. S8). The archive contains logged severities from all agents and is aggregated and classified into the same five-level WDI series, so the simulated and empirical records are compared identically (see Methods). Reproducing the empirical observation measured in historical archives, the correlations between recorded climate by agents and the input climate forcing show the unimodal pattern at an intermediate generational re-standardisation window under the scenario of H1 (generational amnesia) (Fig. 5b, red and black lines). In contrast, the H2 (collective illusion) scenario shows a monotonically increasing trend with window length and no generational optimum (Fig. 5b, blue line). Global (Sobol) sensitivity analysis identifies which of the five parameters (max age, number of new agents, archive loss rate, climate persistence, and variability; Fig. 5d) account for this divergence. Under the scenario H1, the location of the optimal window is dominated by an agent’s lifespan (total-effect index *S*_*T*_ = 1.00 ± 0.06, 95% bootstrap confidence interval; Fig. 5d) and is directly proportional to it (see Supplementary Information Fig. S7). In contrast, if one assumes that agents use the shared archive as the baseline (H2), the simulations did not reproduce the above pattern within the tested parameter ranges (Fig. 5d), including when the length of archive access was varied (Supplementary Information Table S11 and Fig. S8). This heuristic model suggests that generational amnesia (assuming individuals rely on their lived experience) is sufficient to generate generational inconsistencies in the model, whereas collective illusion did not reproduce the observed mismatch pattern in the empirical WDIs.

## Discussion

Our analysis, which compares natural proxies (non-human-produced records, the N-WDI) with historical archives (human-produced, perception-mediated records, the H-WDI) of climatic extremes spanning centuries (Fig. 2), reveals systematic differences consistent with a perceptual bias in collective memory (Figs. 3 and 4). SBS assumes that humans perceive changing environments by comparing them to a memory-based baseline^24–26^, which has been detected in data analyses of both static (shifted-correlation pattern; the over- or underestimated severity extreme is related to previous similar incidents, Fig. 3) and dynamic (improved relevance by restandardisation, Fig. 4) contexts. Since previous SBS cases typically focus on demonstrating a decayed awareness of environmental changes^25^, our approach, which combines archival and paleoenvironmental records, suggests that these biases also emerge in historical narratives and records of climate change—an important form of collective memory in the context of climate monitoring. In historical research, there is a long-standing, well-known trait of recording anomalies while omitting normalities ^31^, which supports the base argument of SBS, i.e., the loss of awareness of the normal. For recorded extremes, comparisons with conditions in previous years are also common, especially when social entities seek to emphasise the unexpected nature of droughts or floods. For example, we can find statements like “This year, the Tao River has risen and swelled more than in previous years” ("今岁洮水涨发，较往年为大”, October 1746 CE) in Chinese historical archives (see Table S4 in Supplementary Information for more examples). Such statements are consistent with the cognitive processes proposed for SBS once a baseline (normality) is set, whereby subsequent perceptions are biased by anchoring^32^ and accessibility-based assimilation following exposure to similar events^33^. These are typically expressed as simplified binary comparisons (e.g., higher/lower) rather than memorising nuanced continuous data ^34^, as in the shifted-correlation pattern we observed.

The shifted-correlation pattern is expected to introduce autocorrelation in time series, which is consistent with our sliding-window restandardisation improving correlations between two WDI series. With an optimal sliding window size of 20–40 years (Fig. 4 and Supplementary Information Table S7), the temporal pattern of correlation improvement reveals another aspect of SBS: inconsistent cross-generational perceptions^25^. Similar generational forgetting has also been identified, for instance, a faded memory of floods in fluvial areas^5^ and hunters’ misassessments of changes in the forest ^27^. These historical or modern, elite or indigenous cases of communal amnesia suggest that generational gaps may be widespread in the context of environmental change. It aligns well with the theory of SBS: when lacking past information, lived experience, or the retrieval of historical conditions, each new generation treats the environment in which it was raised as the normal baseline and builds its environmental perceptions on that baseline^26^. Our optimal sliding window sizes (ranging from 20 to 40 years) are also approximately equal to the generational life expectancy of humans in ancient North China ^35^ (see Supplementary Information Fig. S6). This pattern and its peak in correlation improvements can be reproduced by a heuristic ABM under the assumption of generational amnesia, but not under the assumption of collective memory illusion (Fig. 5). This implies that the emergence of SBS is consistent with biased perceptions shaped by members of the same generation through their experiences, rather than the illusion of shared narratives. Further sensitivity analyses also show that the agents’ life expectancy is the dominant parameter in this scenario, which is sufficient to reproduce the empirical pattern (Supplementary Information Fig. S7).

These findings are consistent with cognitive psychology research suggesting that individual memory is bounded by lifetime experience, so what counts as normal is defined by seasonal patterns within one’s life. We suggest that extreme events serve as triggers that prompt comparisons with this personal baseline, but such judgments are often culturally coherent rather than objectively accurate ^36^. Our results are consistent with triggering events being measured against a shortened frame of roughly one generation, consistent with our optimal sliding window, which reflects generational memory boundaries. A sliding window restandardisation improves correlations, which is consistent with compensating for the systematic bias introduced by memory-based baselines that lack sufficient temporal depth. The embedded and embodied nature of cognition means that, under normal conditions, environmental processes unconsciously enact bodily senses; however, extreme events necessitate active mental engagement, altering perceptions of both normal and extreme events^37^. This cognitive mechanism, operating within socially and culturally governed niches^38^, may underlie the systematic perceptual bias observed in historical climate records. This would imply that each generation sets its baseline from lived experience rather than from long-term variability. These mechanisms of being anchored to past information and more focused on extreme events also form the foundation of our construction of the heuristic ABM (see Supplementary Note 3).

Several limitations warrant consideration. First, historical archives, shaped by institutions, also incorporate social responses that modulate the societal impact in addition to individual perceptions ^39^. This study uses data sources that, by filtering out archives with disaster occurrences but without descriptions of natural phenomena (such as prolonged rainfall or drought), should minimise this data-processing bias. For example, a record of a breached embankment would not be classified as a climatic record in itself unless there are records of continuous heavy rain for months in the same year, which indicate an observation of an extreme wet year (see the principles in Methods and Supplementary Note 1 for more details). Moreover, our consideration of temporal slices addresses issues of changing political context, with similar results across two major Chinese dynasties (Supplementary Note 1 and Fig. S2), suggesting the robustness of our analysis under different institutions (Fig. 4c). For example, around the time of the Emperor Wanli (万历, 1573-1620 CE), a period of prosperity turned to decline for the Ming Dynasty and far fewer fiscal resources were allocated to disaster relief than from 1649 to 1849 CE, when the emperors in the Qing Dynasty established a complex disaster response system. Nevertheless, the unimodal pattern was also observed in both periods (Fig. 4c). There are also some inherent accuracy challenges in palaeoclimate reconstruction when using natural proxies. The uncertainties recorded in tree-ring chronologies inevitably impact the mismatch analysis, but this is already the most temporally precise data source that can be directly matched with historical archives. Here, we collected multiple openly accessible datasets and tested the integrated data against validation data from 1901 to 2000 CE (Fig. 2). Between proxies and instrument-based validation data, the only cells with a statistically significant offset contained one to three mismatched pairs and mostly had the opposite sign (see Supplementary Information Fig. S11); the directional pattern found in the historical archives was therefore not detected in this control comparison. The statistically significant correlation between the N-WDI and instrumental precipitation at most historical-archive sites (19 of 32; Fig. 2c), together with the hydrological connection of these sites through the Yellow River, supports our assumption that the N-WDI is representative. We tested different integration approaches of proxy datasets, and the main conclusion of this study remains unchanged (see Supplementary Note 2). Finally, the ABM is a deliberately minimal, heuristic model rather than a demographic reconstruction. This is mainly because the groups that produced the historical archives do not represent the entire population, and our global sensitivity analysis (Fig. 5d) shows that their life expectancy matters. We have confirmed the key parameter in this simplified demography is consistent with population data we have collected (Supplementary Information Fig. S6). In summary, despite these challenges, our analysis remains robust using current, accessible materials.

The implications of our findings extend beyond historical climatology. Our results suggest that perceptual biases can operate consistently over long timescales, and that the cognitive mechanisms of SBS identified in contemporary contexts^27,40^ have deep historical roots and potentially broad applicability. Evidence of SBS and generational amnesia has been discussed across diverse fields, including public perceptions of urban social incidents^41^, settlement in flood-prone areas^5^, and assessments of the historical condition of degraded ecosystems^26^. As collective memory can be purposefully cultivated to enhance resilience ^9^, it underscores the value of science and history education in establishing public awareness of climate change. This remains relevant today. Although we have instrument-based measurements and more accurate data, the duration of collective memory can disappear more rapidly^42^, with similar decaying patterns across different loss rates^11^. This cognitive bias may contribute to a communication gap between scientists and the public^43^, which can undermine the potential for collective action. Our findings also represent a step toward integrating collective memory into coupled human-natural models to manage long-term disaster risks^18^. For instance, meteorological disaster records should be remembered and evoked by societies, as reflected in research on society-flood feedback ^44^. As one of the oldest and deadliest disasters, many flood experiences are ultimately documented in archives, monuments, and legends^45^. Research shows that this enhances adaptability, sometimes even yielding greater social resilience than relying solely on engineering infrastructure such as levees ^19,46^. In this context, our method demonstrates the feasibility of integrating collective memory with empirical data into a long-term study of extreme events and shocks (e.g., human–flood).

Moreover, our methodology provides a transferable framework for studying long-term collective memory dynamics by combining empirical data from archives and paleo-environmental proxies with computational modelling. It illustrates how slow variables (such as collective memory^18^) can be incorporated into studies of the co-evolution between humans and the environment during the Anthropocene^47^. Our approach can be applied when proxy data of climate and cultural memory are available, as well as in other societal domains where long-term decision-making is necessary^48^. It also highlights the importance of retrieving high-resolution climate records, such as tree rings and reliable archival records of past climate^49^. In this context, our results also provide hints for refining current narratives about climate–society linkages. Prior reviews ^50^ note that many studies assume linear mappings from climate onto social response while overlooking biases introduced during data production and interpretation^51^. By quantifying a structured, generational-scale perceptual imprint, we show that departures between physical variability and social documentation can be systematic, directional, and temporally organised. This argues for treating historical archives as behaviourally transformed climate signals. Cultural and social contexts (e.g., social norms, structure, and networks) likely influence how strongly and where this imprint appears^52–54^, but the pattern itself — emerging from limited experiential memory—may be broadly transferable. Comparative tests across regions, archive genres, and governance regimes can further suggest which features are culture-specific shared misperceptions^55^ and which arise from general cognitive constraints.

Future climate research could benefit from recognising that human climate perception is not a passive recording of physical conditions but an active cognitive process shaped by memory, comparison, and social transmission^56^. In the Anthropocene, where human responses to climate variability increasingly alter Earth system dynamics^47^, incorporating these perceptual mechanisms into climate modelling and policy frameworks helps for understanding and communicating the scale of changes in coupled human-natural systems ^57^. As climate change accelerates, understanding how each generation responds to a new normal or baseline^26^ will become increasingly important. Progress will require studies across regions and cultural settings that combine high-resolution climatic archives with well-curated documentary datasets to probe how extremes are recorded. In particular, the influences of political processes and new media technologies and information flows  on collective memory deserve careful attention, both in reinforcing remembrance and in amplifying forgetting. Moreover, co-evolutionary models developed to study human-Earth system interactions should incorporate systematic data on the role of collective action in adaptation and extreme events^58^. Our work provides both empirical evidence and methodological tools for this integration, supporting further research at the intersection of climate science, cognitive psychology, and historical analysis, and providing an avenue for exploring and appreciating the role of collective memory in societal resilience to extreme climates such as those increasingly likely to be encountered as the 21st century progresses.

## Methods

This research complies with all relevant ethical regulations. This study involved no human participants, human data or animals, and therefore did not require ethical approval. We treat the natural-proxy WDI (N-WDI) reconstructed from tree-ring data as a relatively independent, non-perceptual reference for climatic anomalies. It is independent not because it is free of interpretation (it, too, is a reconstructed proxy shaped by sampling and methodological choices), but because it does not pass through human memory and is compared with the historical-archive WDI (H-WDI), estimated from documentary records and mediated by contemporaneous perception. The differences and mismatched patterns between the two, therefore, can reflect the perceptual biases behind historical archives as a carrier of collective memory. To this end, we first processed two types of data, then applied comparison approaches to the data, and finally explained how to use an ABM to link the data pattern and the SBS theory.

### Data sources and processing

This research compares the severity levels of climatic extremes in North China using two types of datasets: historical archives and tree-ring proxies. Historical archives spanning 1470 to 1900 CE were collected and classified into 5 levels to represent the perception-mediated severity of climatic extremes in collective memory (the H-WDI). Reconstructed climatic variables based on tree-ring proxies were collected and integrated to define the corresponding non-perceptual palaeoclimatic extremes over the same period (the N-WDI). Climate data measured from 1901 to 2000 CE using instrument-based grid datasets were collected to validate the integrated natural proxy data over this period ^59^.

#### Historical archive datasets

This study utilises extreme drought and flood data from the Atlas of Extreme Droughts and Floods over the Past Millennium^60^, which synthesises multi-source historical archives from 1000 to 1949 CE, including primarily official histories (e.g., the History of Ming), local gazetteers (e.g., the Gazetteers of Beijing), Veritable Records (e.g., the Veritable Records of Qing), personal diaries and inscriptions (see Section 1 in Supplementary Note 1 and Supplementary Information Table S1 for differences between the archive sources). Due to the poor retention rate (25.17%, measured as the ratio of archived meteorological data to the total quantity; see Supplementary Note 1) and obvious source bias (mainly based on official histories, with a relative lack of local and folk materials) in the early period (1000–1469 CE), we begin our analysis in 1470 CE (see Supplementary Information Fig. S2). From then on, our analysis period spans two dynasties (Ming and Qing) in ancient China, as recorded in both official histories and local gazetteers. The data retention rates for these two periods are 66.46% and 80.00%. We select 1900 CE as the end year of analysis because this is when instrumental measurement data began to play a core role in climate monitoring.

Throughout the pre-instrumental period (1470–1900 CE), we applied consistent archival data selection standards when decoding the archives, which include the following principles (also see Supplementary Note 1): (1) Priority is given to climatic extremes in summer because of the dominance of the monsoon in influencing regional climate ^29^; (2) recorded societal outcomes of disasters (i.e., description of the social tragedy) also require references to their natural contexts (e.g., flooding, droughts, continuous little/much rains) in corresponding archives; and (3) when information conflicts, data with a more extended impact was prioritized (e.g., a state’s drought record is more important than a normal record of one of its counties.). All archives related to drought-flood events were then classified into five levels of severity: Severe Dry (SD), Moderate Dry (MD), Normal (N), Moderate Wet (MW) and Severe Wet (SW), with the target frequencies of the five levels approximately controlled at 10%, 25%, 30%, 25%, and 10% (i.e., category quantile widths, see Table 1 and Supplementary Note 1 and Fig. S3). This approach, a common practice for inferring Wet/Dry Index (WDI) levels from historical archives^60,61^, was applied to each historical site (see Supplementary Information Table S2).

**Table 1:** Five severity levels of the Wet/Dry Index (WDI) and representative archival examples

Level	Abb.	Z-score	Archive example	Translation
Severe dry	SD	≤ − 1.17*σ*	旱荒，发赈账济，全活甚众。	Due to the drought and famine, relief funds were distributed widely, saving many lives.
Moderate dry	MD	[ − 1.17*σ*, − 0.33*σ*]	夏大旱，至八月始雨，禾尽稿	In the summer, there was a great drought; it did not rain until August, and all the grain withered.
Normal	N	[ − 0.33*σ*, 0.33*σ*]	大稔	Great harvest.
Moderate wet	MW	[0.33*σ*, 1.17*σ*]	九月葵未，诏顺天、保定、河间、永平四府水涝伤稼	In September, accumulated water soaked the grain. Yongding, Baoding, and Hejian prefectures were flooded.
Severe wet	SW	≥1.17*σ*	夏大雨，黄河水溢，城崩	In summer, heavy rains overflowing the Yellow River, the city collapsed.

Generally, concrete determinations of floods or droughts, along with descriptions of severe societal outcomes of disasters, are classified at the Severe level (SD or SW), while alone records of natural conditions (such as heavy rains or prolonged droughts) are classified at the Moderate level (MD or MW). In contrast, a normal (N) year refers to records containing keywords such as harvest, abundant year, ripe, and bountiful, or non-abnormal events in the current year, with continuous records available in the preceding or following years (Supplementary Information Table S3 provides more examples). To integrate archives across the study area, site-year ordinal grades were first converted to continuous z-scores, because ordinal levels cannot be averaged meaningfully. Each grade maps to a z-interval bounded by the same thresholds used for WDI classification (rather than a single point; see Table 1). This means, for the site *i* and year *t*, we drew 100 values from a truncated normal within that interval and used their mean as the site-level continuous WDI, *h*_*i*,*t*_. The regional H-WDI was then *H*_*t*_ = mean({*h*_*i*,*t*_}) over all sites with non-missing records in the year *t* (*n*_*t*_ ≤ 32). Sites without archival evidence were excluded rather than imputed, and years with no reporting sites were left missing. This stochastic mapping is a tie-breaking convenience for cross-site averaging. No statistical method was used to predetermine sample size; the sample comprises all site-years with archival evidence within the study area and period. Data were excluded only as follows: the early period 1000–1469 CE, because of its low retention rate and source bias (see above); site-years without archival evidence, which were left missing rather than imputed; and natural-proxy series that did not meet the selection criteria in Supplementary Note 2. The archival grading was not blinded to the natural-proxy series.

#### Natural proxy datasets

The reconstructed datasets are primarily collected from publicly available NOAA repositories (see Supplementary Note 2 for details of data selection and processing). They are mainly based on tree-ring proxies of summer precipitation and Yellow River streamflow in northern China^30^, and each original tree-ring reconstruction had achieved, in its source publication, calibration-period correlations with instrumental climate above 0.4 (see Supplementary Note 2, Table S8). These publicly available reconstruction datasets sometimes also use other proxy indicators (e.g., oxygen isotopes or historical documents). Therefore, we also tested different data combination scenarios (see Supplementary Information Table S5) and used the scenario with all purely natural data in the main text (to ensure independence). We then employed a Bayesian latent-variable model to integrate these datasets into a standardised N-WDI from 1470 to 2000 CE. Each year’s true anomaly received a weakly informative ${{{\mathcal{N}}}}(0,1)$ prior, matching the unit variance of the standardised inputs, and each reconstruction was modelled as a Student’s *t* observation centred on it. The shared degrees of freedom *ν* were estimated under a weakly informative Gamma (2, 0.1) prior^62^ (posterior mean 36, 94% highest-density interval [18, 58], i.e. close to Gaussian; see Supplementary Note 2 and Table S9). Weighting each series by its local (within-series) rolling variability, the model downweights noisier series and carries their year-to-year disagreement into the posterior credible interval. Each series  was fitted by Markov Chain Monte Carlo (MCMC) in PyMC (version 5.28.4) using four No-U-Turn Sampler (NUTS) chains of 2000 draws each (1000 tuning steps, target acceptance 0.95; all $\hat{R}\le 1.01$), and posteriors were analysed with ArviZ (version 0.20.0). The N-WDI is the per-year posterior mean; the main results did not change under seven alternative priors (Supplementary Note 2 and Table S10).

Extreme wet and dry years were identified by classifying the posterior-mean N-WDI with quantile-based thresholds (e.g., upper and lower 10% for a 20% double-tail probability). To maintain consistency with the classification standards in H-WDI, we have chosen the same target category frequencies (quantile widths) for the five levels with same names (see Table 1). Accordingly, the standardised thresholds are empirical boundaries rather than theoretical normal quantiles so that the resulting grade frequencies match the preset targets, and this empirical  ± 1.17*σ* differs slightly from the theoretical  ± 1.28*σ* in an idealised normal distribution. We adopt the same thresholds for natural-proxy binning, ensuring both datasets share a consistent grading scheme. For the period from 1901 to 2000 CE, which has abundant modern instrumental data, summer precipitation from July to September is used as the variable, standardised to z-scores over 1901–2000 CE only, and its anomaly spectrum is also divided into five severity levels using the same thresholds to determine drought and flood levels. These are then compared with drought-flood levels reconstructed from tree-ring proxies for the period 1901–2000 to assess the reliability of the integrated natural proxy data. The year-to-year agreement was measured with Pearson’s *r*, reported as *r*(df) with df = *n* − 2. Both validation-period series were consistent with normality (two-sided Shapiro–Wilk test, n = 100 years: N-WDI *W* = 0.984, *p* = 0.270; instrumental series *W* = 0.986, *p* = 0.399), and a two-sided permutation test (9,999 permutations), which does not assume normality, gave the same result (*p* < 0.001). We tested three different sets of modern instrumental measurements and three scenarios of natural proxy combination data; all yielded qualitatively similar results (Supplementary Note 2, Fig. S4 and Table S6).

### Records comparison

#### Confusion matrix and correlation

The quantification of classification accuracy between different datasets (proxy data against measured data or historical data against proxy data) is based on the construction of confusion matrices from the categorised severity levels. Perfect matches appear on the diagonal, and mismatches appear off-diagonally. Statistical validation of classification agreement was done employing Cohen’s weighted Kappa coefficient^63^ with quadratic weighting (${\kappa }_{w}=\frac{{p}_{o}-{p}_{e}}{1-{p}_{e}}$), where *p*_*o*_ represents observed agreement and *p*_*e*_ denotes expected agreement under random classification. The quadratic weighting scheme assigns penalties proportional to the squared distance between classified categories, recognizing that misclassifying severe drought as severe wet constitutes a more substantial error than adjacent category confusion. Moreover, Kendall’s *τ*^64^, selected for its robustness to ranked categorical variables, was used to test the correlation between the five-level categorised datasets (including historical archival data and natural WDI data after binning). We determine statistical significance by using a two-sided *α* = 0.05 threshold for Kendall’s *τ*. The significance of *κ*_*w*_ was assessed with a two-sided permutation test (10,000 permutations of the archival categories against the natural ones, which preserves both marginal distributions), and 95% confidence intervals for Kendall’s *τ* and *κ*_*w*_ were obtained with a percentile bootstrap over years (10,000 resamples). Kendall’s *τ*, the permutation test for *κ*_*w*_ and the bootstrap intervals are non-parametric and do not assume normality or equal variances.

#### Bias of mismatches detection

We write *N*_*t*_ and *H*_*t*_ for the five-level (ordinal) categories assigned at year *t* by the natural proxy (N-WDI) and the historical archive (H-WDI), respectively, and *x*_*t*_ for the underlying continuous N-WDI value. A mismatch is a year where *H*_*t*_ ≠ *N*_*t*_. We tested whether mismatches show a systematic signature of comparing the current year to earlier years with the same natural severity category. For each mismatch year *t* we therefore identified a reference year $t{\prime}$ at which the N-WDI had previously been assigned the same category. Formally, $t^{\prime}$ is the calendar year of the shift times prior observation with the same natural severity category, i.e. ${N}_{t{\prime} }={N}_{t}$, $t^{\prime} < t$, where the integer shift ∈ {1, 2, 3} denotes the first, second, or third such prior occurrence (Fig. 3b). Note that the matching is on the discrete category *N*, not on a continuous scale. We then asked how much the underlying natural climate had actually changed between these two years sharing the same natural category of N-WDI, quantified as the z-score difference of the continuous N-WDI values *x*_*t*_ and ${x}_{t^{\prime} }$ only for the mismatches (*H*_*t*_ ≠ *N*_*t*_), i.e., ${{{\Delta }}}_{t,t^{\prime} }={x}_{t}-{x}_{t^{\prime} }$; where *x*_*t*_ and ${x}_{t^{\prime} }$ are the continuous N-WDI (natural-proxy) values at the current and reference years. As a control comparison, we also input the severity levels inferred from instrumental data (validation period, 1901–2000 CE) as a synthetic historical sequence *H*_*t*_ into the above approach to calculate ${{{\Delta }}}_{t,t^{\prime} }$, assessing whether the mismatching pattern appears only in historical archives.

A non-zero ${{{\Delta }}}_{t,t^{\prime} }$ of continuous natural values, therefore, indicates the baseline has drifted and its sign gives the direction (a positive ${{{\Delta }}}_{t,t^{\prime} }$ indicates the archive over-estimated severity relative to the earlier event, and vice versa). Averaging ${{{\Delta }}}_{t,t{\prime} }$ within each cell (*i*, *j*) (natural level *i* against archival level *j*) yields the observed bias matrix ${{{\Delta }}}_{ij}^{\,{\mbox{obs}}\,}$ over mismatch years only. We assessed the statistical significance of this bias pattern with a Monte Carlo randomisation test that compares the observed bias matrix against a null model in which archival severity categories are assigned independently of the natural climate sequence. The null hypothesis is that archival classifications depend only on their fixed marginal frequencies *P*(*H* = *j*) = *p*_*j*_ ({0.10, 0.25, 0.30, 0.25, 0.10}), with no association between archival grades and the natural series. Each iteration generated a synthetic dataset in which natural climate values ${x}_{{{{\rm{sim}}}}} \sim {{{\mathcal{N}}}}(0,1)$ were binned into five severity levels using the same thresholds applied to the proxy data, and archival classifications were independently redrawn from *p*_*j*_. We ran 10,000 iterations, each with a sample size matching the empirical record length (*n* = 431). For each iteration *k*, the bias matrix Δ^(*k*)^, indexed by natural level *i* and archival level *j*, was recomputed with the identical look-back algorithm used on the empirical data (the same shift and within-grade pairing ${N}_{t{\prime} }={N}_{t}$, $t{\prime} < t$). This null preserves the archival marginals and the look-back mechanics but destroys any dependence between archival assignments and the natural climate sequence. For each matrix cell (*i*, *j*), we computed standardized scores ${z}_{ij}=({{{\Delta }}}_{ij}^{\,{\mbox{obs}}}-{\mu }_{ij}^{{{{\rm{null}}}}})/{\sigma }_{ij}^{{\mbox{null}}\,}$, where ${\mu }_{ij}^{\,{\mbox{null}}\,}$ and ${\sigma }_{ij}^{\,{\mbox{null}}\,}$ are the mean and standard deviation of ${{{\Delta }}}_{ij}^{(k)}$ across the 10,000 iterations, and obtained two-tailed *p*-values as *p*_*ij*_ = 2(1 − Φ(∣*z*_*ij*_∣)), which assumes that the null distribution of each cell is approximately normal. These cell-level *p* values were not adjusted for multiple comparisons. The null simulations are seeded (random seed 42) so that the reported *p* values are reproducible.

#### Sliding window and re-standardisation

Given the hypothesis of SBS that limited experience and perceptual bias matter, a rolling window approach may potentially address systematic patterns in the correlation strength between the natural-proxy (N-WDI) and historical-archive (H-WDI) data. We generated a correlation surface through systematic evaluation across the two parameter spaces defined by window sizes *w* years and minimum sample requirements *m*. For each combination of parameters (*w*, *m*), we computed Kendall’s *τ* coefficient *τ*_*w*,*m*_ between the re-standardised natural data and historical data. The re-standardisation employs a standard-deviation-based transformation designed to emphasize temporal anomalies relative to recent *w* years of experience. At time *t*, the re-standardised value ${f}_{t}^{(w)}$ is calculated as ${f}_{t}^{(w)}=\frac{{x}_{t}-{\mu }_{t}^{(w)}}{{\sigma }_{t}^{(w)}}$, where ${\mu }_{t}^{(w)}=\frac{1}{w}\mathop{\sum }_{i=t-w+1}^{t}{x}_{i}$ and ${\sigma }_{t}^{(w)}=\sqrt{\frac{1}{w-1}\mathop{\sum }_{i=t-w+1}^{t}{({x}_{i}-{\mu }_{t}^{(w)})}^{2}}$ represent rolling window mean and standard deviation respectively. We applied this re-standardisation to the natural data, and the baseline correlation *τ*_0_ was computed between the un-re-standardised natural reconstructions *x*_*t*_ and historical classifications *H*_*t*_, enabling identification of whether the assumption of perceptual bias improves the correlation. Statistical significance assessment employed two-tailed tests with *α* = 0.05 significance threshold. Temporal stratification accounted for varying historical data stages and thus analysis proceeded separately for pre-instrumental periods (1470–1900 CE), but was also tested during different periods with the similar period length for robustness.

### Agent-based model (ABM)

We further developed an ABM to investigate how collective climate perception can emerge from individual observers’ experiences and perceptual biases. The model simulates a population of climate observers who record climatic extremes based on two core cognitive mechanisms: (1) more abnormal extremes are more likely to be recorded, implementing a negativity bias^65^ in the recording propensity (the probability *p*_*t*_ defined below); and (2) observers assess the severity of an extreme relative to a memory anchor ^33^ (the standardised-deviation perception *z*_*i*,*t*_ defined below). We test two scenarios (limited experience or collective archives) and expect similar patterns to emerge between the synthesised N-WDI and H-WDI and the real-world datasets.

#### Model architecture

The climate forcing *c*_*t*_ is generated by a stationary stochastic process over a simulation period of several centuries. In the main analysis it follows an AR(1) autocorrelated process, *c*_*t*_ = *ϕ* *c*_*t*−1_ + *ε*_*t*_, with climate persistence *ϕ* = 0.5 and innovations scaled to climate variability *σ*_*c*_ = 1. Independent Gaussian noise (*ϕ* = 0) and linear-trend variants are evaluated for robustness (see Supplementary Note 3 and Figure S9). The model maintains a global archive ${{{{\mathcal{A}}}}}_{t}=\{{e}_{t,1},{e}_{t,2},\ldots,{e}_{t,{n}_{t}}\}$ recording all extreme events documented at time *t*, where *n*_*t*_ represents the number of active observers capable of recording events. Climate observers are implemented as *N*_agent_ autonomous agents characterized by finite lifespans, where typically ${L}_{\min }=10$ and ${L}_{\max }=40$ years represent their start and end ages for recording. Each observer maintains memory capacity implemented ${M}_{i}(t)=\{{c}_{t-{L}_{i}+1},{c}_{t-{L}_{i}+2},...,{c}_{t}\}$ constraining itself to recall experiences of climate. Climate perception operates through a standardized deviation calculation relative to the agent’s reference baseline (personal or collective; see below). For agent *i* at time *t*, the perceived climate anomaly is ${z}_{i,t}=\frac{{c}_{t}-{\mu }_{i}(t)}{{\sigma }_{i}(t)}$; where *μ*_*i*_(*t*) and *σ*_*i*_(*t*) are the mean and standard deviation of the baseline that agent *i* is referring to. The setup of baselines is shown in the section of experimental settings below.

According to a probability *p*_*t*_, the observer may record its perception in severity of climatic extreme ${s}_{i}^{t}$ at each time step *t*, so that the global archive is the average judgment from all observers ${e}_{t}=\frac{1}{n}(\mathop{\sum }_{i}^{n}{s}_{i}),n\le {N}_{{{{\rm{agent}}}}}$. Not every recorded judgement enters the public archive: each event is retained only with probability 1 − *λ* and is otherwise forgotten (the archive loss rate *λ*, default 0.4). The probability *p*_*t*_ is estimated by *p*_*t*_ = *f*_0_ + 0.5 − *S*(∣*z*_*i*,*t*_∣), where *f*_0_ represents baseline recording probability typically set to 0.1, and *S*( ⋅ ) = 1 − Φ( ⋅ ) denotes the survival function of the standard normal distribution (scale 1). This formulation ensures that extreme deviations approach a maximum recording probability of *f*_0_ + 0.5 (0.6 by default), while normal years maintain low but non-zero recording likelihood (see Supplementary Note 3 for details). The per-year aggregate record *e*_*t*_ is then discretised into the same five ordinal WDI categories used for the archives (using the identical standardised thresholds applied to the empirical data), yielding a simulated archival series. This series and the underlying climate *c*_*t*_ are compared using the same sliding-window re-standardisation with Kendall’s *τ* used in the empirical analysis. The model maintains demographic stability through continuous population turnover. At each time step, *N*_new_ agents enter the population while agents exceeding maximum lifespan are removed, thus creating overlapping generational cohorts with varying memory spans. Model outputs are produced only after the number of observers stabilises at the maximum *N*_agent_.

#### Experimental settings

In the model, there are two types of baselines available for agent comparison: a personal baseline (i.e., *μ*_*i*_ and *σ*_*i*_ are calculated from the agent’s own memory *M*_*i*_) and a collective baseline (i.e., *μ*_*i*_ and *σ*_*i*_ are calculated from the public climate record accumulated over the full elapsed period, *e*_1_, …, *e*_*t*−1_, *e*_*t*_). This mechanism simulates how current climatic extremes are contextualized against historical precedents, potentially leading to systematic over- or under-estimation depending on the baseline. This setting responds to the SBS assumption that the next generation adopts a new reference baseline, and it is controlled by a single switch parameter in the model. The experience scenario (each agent referring only to its own lived memory *M*_*i*_) instantiates hypothesis H1, generational amnesia, whereas the archive setting (agents referring to the shared archive accumulated over the full elapsed period) instantiates H2, collective memory illusion; the experimental results are then compared under these two scenarios. Two further baseline variants are evaluated only for robustness (see Supplementary Information Table S11 and Fig. S8).

The model generates output time series spanning centuries, enabling correlation analysis between agent-recorded events *H*_*t*_ and underlying climate reality *c*_*t*_. We employ the same correlation calculations and rolling window methodology as in the empirical analysis, with Kendall’s *τ* computed across various window sizes and compared to the benchmark correlation. We also conduct a global, variance-based Sobol sensitivity analysis (Saltelli sampling, *N* = 1024 base samples, each parameter vector evaluated over 30 stochastic repeats), reporting both first-order (*S*_1_) and total-effect (*S*_*T*_) indices over five parameters: (1) maximum lifespan ${L}_{\max }$, (2) the number of new agents per year *N*_new_, (3) the archive loss rate *λ*, (4) climate variability *σ*_*c*_ and (5) climate persistence *ϕ*, to identify which factors govern the location of the generational optimum. The ABM’s ODD+D protocol can be found in Supplementary Note 3.

### Reporting summary

Further information on research design is available in the Nature Portfolio Reporting Summary linked to this article.

## Supplementary information

Supplementary Information

Reporting Summary

Supplementary Dataset 1

Description of additional supplementary files

Transparent Peer Review file

## Data Availability

The historical Wet/Dry Index (H-WDI) analysed here is derived from the Atlas of Extreme Droughts and Floods over the Past Millennium in China ^60^ (China Cartographic Publishing House and Zhonghua Map Society, 2024; ISBN 978-7-5588-0466-3), a copyrighted commercial publication available for purchase from the publisher. The annual N-WDI (posterior mean, standard deviation, and 94% highest-density interval, 1470–2000 CE) and the regional H-WDI (1470–1900 CE, with the number of contributing sites), each with its five-level class, generated in this study are provided as Supplementary Data 1. All tree-ring hydroclimate reconstructions used to construct the natural-proxy WDI (N-WDI) are publicly available from the NOAA/NCEI World Data Service for Palaeoclimatology; study pages are listed in Supplementary Table S8. Instrumental validation data are publicly available: the 1 km monthly precipitation dataset for China^59^ at https://doi.org/10.5281/zenodo.3114194; GPCC Full Data Monthly Version 2022 at 0.5^∘^^66^ at https://doi.org/10.5676/DWD_GPCC/FD_M_V2022_050; and CRU TS4.08 ^67^ from the Centre for Environmental Data Analysis at https://catalogue.ceda.ac.uk/uuid/715abce1604a42f396f81db83aeb2a4b, where access requires free user registration. Historical administrative geography was obtained from the China Historical Geographic Information System (CHGIS) Version 6 (https://dataverse.harvard.edu/dataverse/chgis), which is licensed for non-commercial academic use only. All main-text and Supplementary display items can be reproduced from the datasets listed above together with the analysis code (see Code availability).

## Code availability

The analysis pipeline and the agent-based model are available at https://github.com/SongshGeo/shifting_baseline; the version used in this study is archived on Zenodo at https://doi.org/10.5281/zenodo.23223235^68^. The agent-based model is implemented with the open-source ABSESpy framework^69^ (ABSESpy version 0.11.5; https://github.com/SongshGeoLab/ABSESpy); its full ODD+D protocol is provided in Supplementary Note 3. Analyses were run in Python 3.11 with NumPy 2.4.4, pandas 3.0.2, SciPy 1.17.1, scikit-learn 1.8.0, xarray 2026.4.0, PyMC 5.28.4, ArviZ 0.20.0 and SALib 1.5.2; maps were drawn with GeoPandas 0.14.4, matplotlib 3.10.8 and QGIS 4.2.0. PaperBell 5.0.3 was used to organise and typeset the study.
