# Supplementary material for: Archival and palaeoenvironmental documentation of extreme climate events reveals generational amnesia in collective memory
Source: Nat Commun. 2026 Nov 9;17:11327. doi: 10.1038/s41467-026-78922-x (PMC13748574; doi:10.1038/s41467-026-78922-x)
Supplement: Supplementary file 1 [file 41467_2026_78922_MOESM1_ESM.pdf]

Supplementary Information for "Archival and palaeoenvironmental documentation of extreme climate events reveals generational amnesia in collective memory"

Shuang Song 宋爽[1,2,3*], Bo Hu 胡博[4], David Max Findley[1], Xutong Wu 武旭同[3], Wei Liu 刘威[5,6], Robert W. Middeke-Conlin[2,7], Victor L Caetano-Andrade[1], Yuda Yang 杨煜达[5], Shuai Wang 王帅[3], Jürgen Renn[2], Patrick Roberts[1,8]

[1] Department of Co-evolution of Land Use and Urbanisation, Max Planck Institute of Geoanthropology, Jena, Germany.
[2] Department Structural Changes of the Technosphere, Max Planck Institute of Geoanthropology, Jena, Germany.
[3] State Key Laboratory of Earth Surface Processes and Disaster Risk Reduction, Faculty of Geographical Science, Beijing Normal University, Beijing, China.
[4] School of Social and Behavioral Sciences, Nanjing University, Nanjing, China.
[5] Institute of Chinese Historical Geography, Fudan University, Shanghai, China.
[6] Department of Atmospheric and Oceanic Sciences, Fudan University, Shanghai, China.
[7] Research Group ASTRA, Max Planck Institute for the History of Science, Berlin, Germany.
[8] Institut für Orientalistik, Indogermanistik, Ur- und Frühgeschichtliche Archäologie, Friedrich Schiller Universität, Jena, Germany.

*Corresponding author: song@gea.mpg.de

1 Supplementary Note 1: Details of the historical archives

1.1 Formation and preservation of historical source types

Historical climate Wet/Dry Index (WDI) from the data source Atlas of Extreme Droughts and Floods over the Past Millennium[1]. Because it has long been China's political centre, northern China has the most comprehensive data and is mainly located in the large Yellow River basin (Figure S1). The data collection spans three phases that reflect changing source availability: Phase I, dominated by official histories (1000-1470 CE), Phase II with balanced emphasis on official histories and local gazetteers (1470-1649 CE), and Phase III primarily relying on local gazetteers supplemented by official histories (from 1649 CE to the instrumental period). Each phase is characterised by distinct archival formations, compilation processes, and preservation patterns that directly influence the reliability and scope of climate documentation.

The 1470–1900 CE study window is set by the density of the documentary material itself. Counting the number of Northern-China gazetteer works compiled per decade shows that documentation is genuinely sparse before

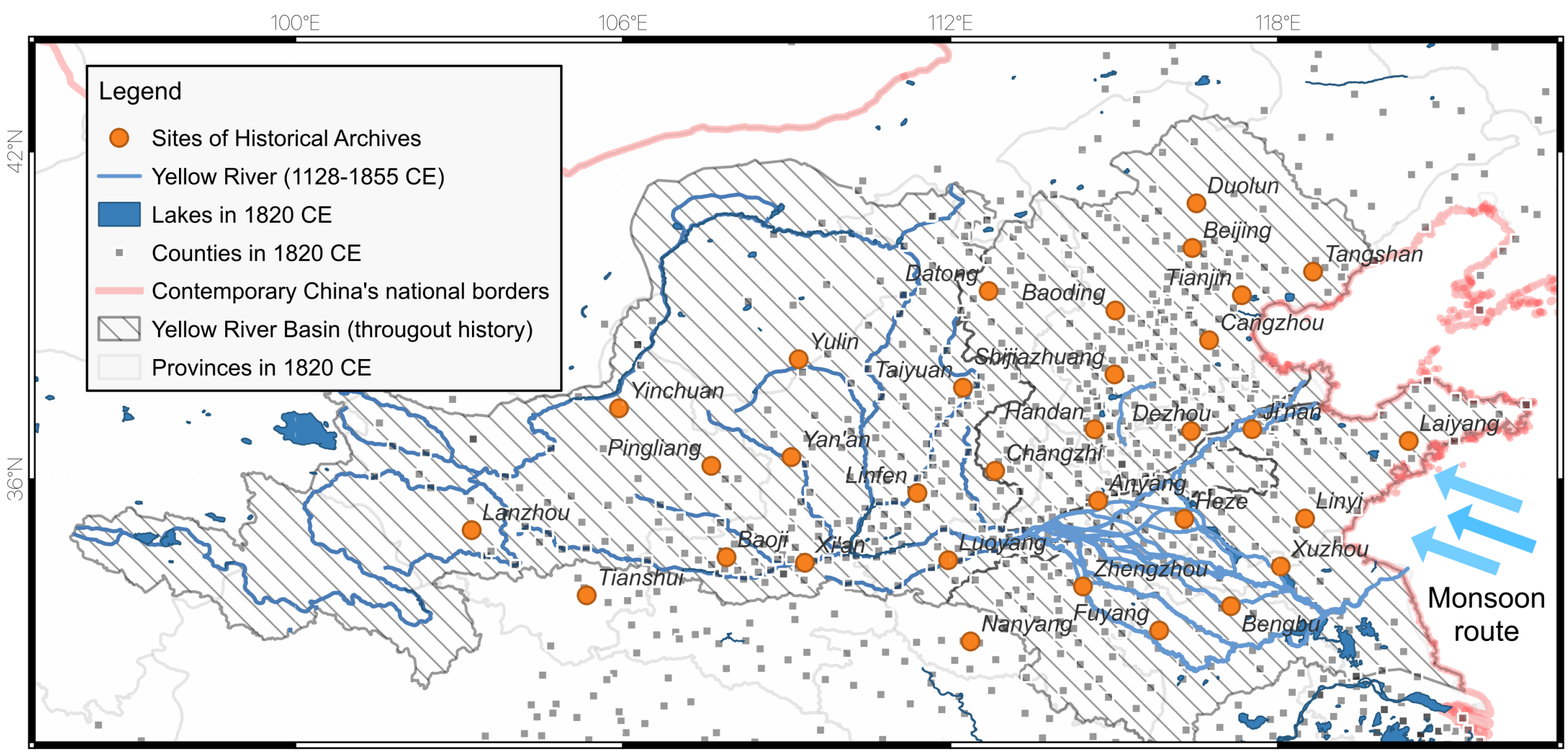

Figure S1 – The study area, North China, has been hydrologically linked by the Yellow River and its climate is dominated by monsoon. The sites of historical archives representing climatic extremes (1470-1900 CE) are shown in orange circles. Provinces, counties and lakes in 1820 CE are from CHGIS Version 6 ((c) Fairbank Center for Chinese Studies and the Institute for Chinese Historical Geography at Fudan University, Dec 2016), shown for the study area only. CHGIS data are licensed for non-commercial academic use only; these layers are therefore not covered by the article's CC BY licence and may not be reused for commercial purposes. The historical courses of the Yellow River (1128–1855 CE) were provided by the author of a previous study[2]; contemporary national borders are from the Standard Map Service of the Ministry of Natural Resources of China (approval number GS(2020)4619). The monsoon arrows are schematic and were drawn by the authors; the map was compiled by the authors in QGIS (version 4.2.0).

about 1470 (0–1 work per decade), becomes usable through the late Ming, and surges with Qing consolidation after 1644, remaining dense through 1900 (Figure S2). The window, therefore, spans two successive dynasties (Ming and Qing) with different administrative and compilation institutions.

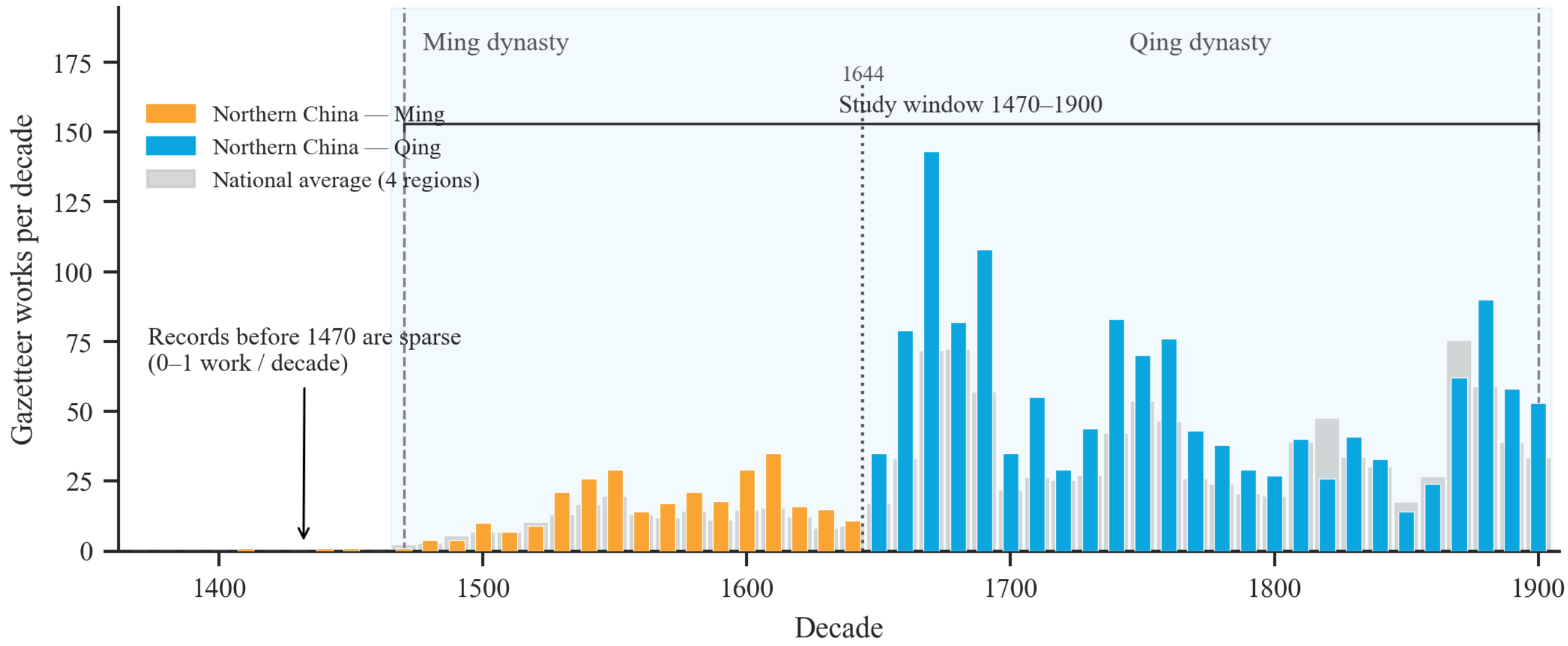

Figure S2 – Decadal density of Northern-China gazetteer works over 1370–1900 CE. Northern-China works are coloured by dynasty (orange = Ming, blue = Qing); faint grey bars show the national average across four macro-regions for reference, and the shaded band marks the 1470–1900 CE study window (dotted line at the 1644 Ming–Qing transition). n = 1,707 Northern-China gazetteer works over 54 decades.

1.1.1 Official Dynastic Histories

Official dynastic histories represent the most prestigious category of Chinese historical documentation, compiled posthumously by subsequent regimes using standardised editorial protocols. These state-sponsored compilations systematically drew on veritable records (see below), administrative memorials, imperial archives, biographical collections, and earlier historical works, organising the content into conventional sections, including annals, treatises, biographies, and chronological tables. Court historians and specialised editorial offices, particularly Hanlin (翰林) academicians operating under direct imperial mandate, served as primary compilers. Notable examples include the History of the Ming, compiled under Qing supervision, and the Draft History of the Qing, compiled during the early Republic, though the latter was never formally canonised. Preservation occurred through imperial libraries, palace repositories, and major court-sponsored collections such as the comprehensive Four Treasuries project, with copies later transferred to national libraries. These sources exhibit characteristic biases, including normative Confucian interpretative frameworks, teleological and moralistic judgments, legitimating narratives supporting ruling dynasties, selective omission and censorship of sensitive materials, retrospective editorial smoothing, rich prosopographical detail for elites, and uneven coverage of lower social strata.

1.1.2 Veritable Records

Veritable Records (实录、起居注) constitute a second major category of imperial documentation, formed through systematic recording of daily court activities in court diaries that were later synthesised into comprehensive Veritable Records at the conclusion of each emperor's reign. These compilations drew extensively from imperial edicts, administrative memorials, and detailed audience notes maintained by court scribes. Dedicated Veritable Records bureaus, supervised by court diarists, managed the compilation process, with senior editors overseeing stylistic consistency and content sensitivity. Palace archives served as primary preservation sites, with the Ming Veritable Records and Qing Veritable Records achieving substantial survival rates. However, many raw court diaries were subject to tight control and occasional destruction after they were synthesised into official records. These sources exhibit chronological organisation and temporal proximity to recorded events, while demonstrating systematic curation designed to protect imperial authority. Sensitive content is channelled through confidential mechanisms and is frequently redacted, euphemised, and post-facto editorialised. Records of climatic and disasters are usually relatively well preserved.

1.1.3 Local Gazetteers

Local gazetteers (地方志) represent the most abundant and geographically comprehensive category of historical documentation, compiled periodically on a jurisdiction-based system covering county, prefecture, and provincial administrative levels, typically every few decades. These compilations were built systematically upon earlier gazetteers, local documentary collections, and contemporary surveys to create comprehensive regional records. Local magistrates served as editors-in-chief while local literati and administrative clerks collected diverse materials, including textual records, cartographic documentation, statistical compilations, and epigraphic inscriptions. The compilation process also incorporated administrative archives and registers generated by local clerks, fiscal offices, and banner administrators through periodic copying and recertification procedures. Preservation occurred through yamen libraries, private scholarly collections, and provincial academies, with later inclusion in large-scale compilations and national library systems. While the early Ming period suffered severe losses due to political upheaval, both the Ming and Qing period gazetteers achieved excellent preservation rates. These sources demonstrate characteristic features including local boosterism and promotional content, conservative textual copying practices, variable data quality across different localities, systematic underreporting of social disorder and political instability, rich documentation of topographical features, institutional arrangements, and notable individuals, excellent coverage for demographic time-slice analysis, but uneven temporal continuity for series data.

1.1.4 Personal Diaries

Personal diaries (个人日记) provide intimate perspectives on historical events through daily notes that were often later recopied and edited by their authors, encompassing travel journals, event logs, and personal observations of contemporary conditions. These sources were created primarily by literati, government officials, and merchants, with subsequent editorial processing by the original authors, their descendants, or commercial

publishers seeking to preserve or disseminate important accounts. Preservation occurred through family holdings, private library collections, and later inclusion in scholarly series, though survival rates remain fragmentary due to the personal nature of these materials. Personal diaries offer a high degree of immediacy and descriptive detail, along with candid observations of contemporary events, while exhibiting biases, including conscious self-fashioning by authors, systematic gaps in coverage, perspectives limited to the literate elite, and later editorial pruning that may have altered the original content.

1.1.5 Inscriptions

Inscriptions (碑刻、摩拓) constitute a distinctive category of historical documentation through commemorative texts carved on stone for public works, temples, academies, and official monuments, typically preserved through systematic copying as ink rubbings. Stone carvers worked from supplied texts while antiquarian scholars compiled descriptive catalogues of notable inscriptions. Preservation occurred through original stone monuments, museum collections, and circulated printed rubbings, enabling the wide distribution of textual content. These sources exhibit public-facing and celebratory characteristics, are characterised by formulaic language conventions, and have relatively limited climate data.

1.1.6 Comparison of data reliability and Information volume

Official Dynasty climate records and Veritable Records are generally reliable (especially in the Qing Dynasty) due to multiple reporting systems and strict oversight, including imperial review. Personal writings are also trustworthy as they reflect direct observations and in-time records. Although individuals can be unreliable, the vast majority of people capable of keeping a diary are usually well-educated and reliable. Local gazetteers, although useful for officials, are less reliable due to social bias, memory loss, copying errors, and variations in detail at different administrative levels. Inscriptions recording weather are relatively few compared to other sources but are heavily biased toward extreme events; overall, they are highly reliable. According to Ge (1990)[3], climatic information in Chinese Archives tends to exhibit a temporal distribution, and the normal distribution is more commonly used; the five-level classification is also the most appropriate. Based on this, the climate information ratio (i.e., the ratio of useful information to uncertainty) can be summarised in (Table S1):

Table S1 – Climate information in normal distribution from different archives.

Archival type	Climate information values	Information ratio
Official Histories	0.67	0.32
Local Gazetteers	0.23	0.111
Personal Diaries	0.44	0.211

In addition, since local gazetteers are reference books compiled periodically, Ge's research also discusses the potential impact of this memory decay. It provides statistics on the compilation cycles of some typical local gazetteers, which average approximately 55 to 120 years. The shortest interval between two compilations was

only 14 years, while the longest exceeded two centuries (244 years). Therefore, the intergenerational cycle phenomenon identified in this study cannot be explained by the compilation cycles of local gazetteers.

1.2 Principles and Operation in Selection

According to the practical convention of inferring the Wet/Dry Index (WDI) using historical archives, the study area (North China) is divided into 32 sites (see Table S2; site coordinates from the atlas[1]) to represent the climatic conditions of their surrounding areas, and these sites have spatial correspondence with the earliest contemporary instrumental stations. Since historical archives must be interpreted within appropriate seasonal contexts, particularly in the study area, where precipitation patterns exhibit substantial seasonal variability and a monsoon-influenced climate, summer rainfall (June-September) is hydrologically the most important and thus takes priority.

Several categories of historical disasters require careful distinction from true hydrological extremes. Locust plagues, while frequently documented and agriculturally important, do not necessarily indicate hydrological conditions and are excluded from Wet/Dry conditions unless explicitly linked to hydroclimate anomalies. Small-scale meteorological phenomena (e.g., floods) cause agricultural damage but lack regional hydrological relevance. Records mentioning only hail or isolated rain-hail events are systematically excluded from the grading system. Famine reports and relief efforts documented in the spring often reflect consequences of the previous year's disasters rather than current-year hydrological conditions.

Based on evaluations of data retention rates across sites, probability densities are set to select elements based on data retention characteristics at different stages. In line with the widely recognised principle in historical literature that "record anomalies, omit normalities", we assume that, as long as the documentation system itself is not interrupted, most extreme disaster information with a 10% probability of occurrence will still be retained, even when overall data retention is relatively low. As discussed in the previous section, we follow a five-level Wet/Dry Index (WDI). In this classification, $\pm 1.17\sigma$ is an empirical threshold derived by the compilers from a reverse inference of the frequency distribution of actual precipitation data[1]. Precipitation data roughly follow a normal distribution but are not strictly normal, so their empirical quantiles ($\pm 1.17\sigma$) deviate somewhat from the theoretical quantiles of the standard normal distribution (for example, the 10% quantile corresponds to -1.28σ, (Figure S3)).

1.3 Classification Criteria

After selecting and processing the original meteorological data cards and accounting for slight differences in material sources across different periods (as previously mentioned, mainly around the year 1649), the following WDI grading method is adopted. Because source texts vary in purpose and editorial conventions, we implemented a rigorous protocol to separate hydrometeorological information from agricultural, social, or administrative consequences.

Table S2 – Historical-archive sites, their location, and the site-level validation statistics behind main-text Figure 2c.

Index	Name	Longitude	Latitude	r	p	n
0	Lanzhou	103°13′49″E	35°4′8″N	0.16	0.108	100
1	Tianshui	105°20′17″E	33°52′32″N	0.22	0.028	100
2	Yinchuan	105°55′50″E	37°18′1″N	0.16	0.106	100
3	Yulin	109°13′15″E	38°11′55″N	0.15	0.131	100
4	Yan'an	109°5′20″E	36°24′24″N	0.22	0.028	100
5	Baoji	107°53′59″E	34°34′23″N	0.19	0.062	100
6	Xi'an	109°20′11″E	34°28′17″N	0.22	0.031	100
7	Duolun	116°30′40″E	41°3′13″N	0.16	0.104	100
8	Tangshan	118°38′49″E	39°48′11″N	0.27	0.006	100
9	Beijing	116°26′7″E	40°14′14″N	0.19	0.054	100
10	Tianjin	117°20′34″E	39°22′18″N	0.24	0.017	100
11	Baoding	115°1′28″E	39°5′29″N	0.18	0.068	100
12	Cangzhou	116°44′31″E	38°32′50″N	0.21	0.039	100
13	Shijiazhuang	115°0′29″E	37°55′17″N	0.22	0.025	100
14	Handan	114°38′29″E	36°54′59″N	0.21	0.036	100
15	Dezhou	116°24′41″E	36°53′0″N	0.20	0.044	100
16	Ji'nan	117°31′54″E	36°55′3″N	0.23	0.023	100
17	Laiyang	120°23′57″E	36°42′5″N	0.27	0.006	100
18	Linyi	118°30′2″E	35°16′55″N	0.24	0.016	100
19	Heze	116°17′20″E	35°16′30″N	0.22	0.029	100
20	Datong	112°42′11″E	39°26′52″N	0.18	0.072	100
21	Taiyuan	112°13′56″E	37°40′40″N	0.21	0.040	100
22	Linfen	111°23′51″E	35°44′57″N	0.34	< 0.001	100
23	Changzhi	112°49′16″E	36°9′23″N	0.26	0.010	100
24	Anyang	114°42′33″E	35°36′27″N	0.25	0.012	100
25	Zhengzhou	114°25′49″E	34°2′15″N	0.24	0.018	100
26	Luoyang	111°57′42″E	34°31′5″N	0.29	0.004	100
27	Nanyang	112°22′16″E	33°1′49″N	0.18	0.072	100
28	Fuyang	115°49′37″E	33°13′53″N	0.18	0.076	100
29	Bengbu	117°8′27″E	33°40′38″N	0.18	0.075	100
30	Pingliang	107°37′16″E	36°14′48″N	0.17	0.084	100
31	Xuzhou	118°3′14″E	34°24′9″N	0.19	0.055	100

Coordinates (WGS84) are the site centres provided by the atlas authors. r is Pearson's correlation coefficient between the N-WDI and the instrumental validation grid cell nearest to each site over the validation period (1901-2000 CE), with the two-sided p of the corresponding t test (df $= n - 2$) and the number of paired years n; p values are not adjusted for multiple comparisons. 19 of the 32 sites reach $p < 0.05$ (main-text Figure 2c).

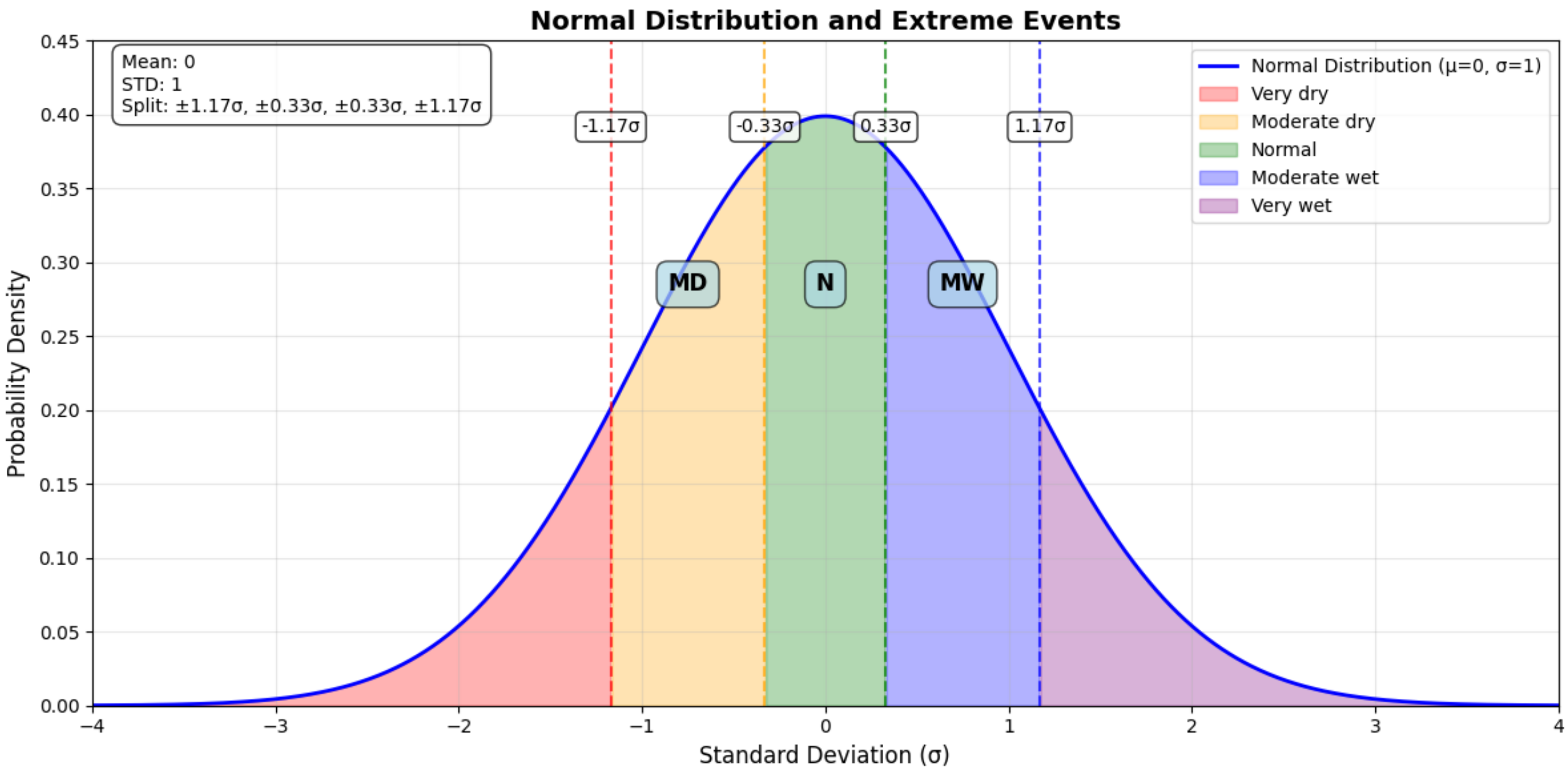

Figure S3 – The distribution of the five-level Wet/Dry Index (WDI). We grade WDI for each site of historical archives into five categories: Severe Wet (SW), Moderate Wet (MW), Normal (N), Moderate Dry (MD) and Severe Dry (SD). The curve is a theoretical standard normal distribution (mean 0, standard deviation 1) with the classification thresholds at ± 0.33 and ± 1.17; it illustrates the classification scheme and is not fitted to data.

1.3.1 1470-1649 CE

During this period, regional documentation coverage exceeded 60% at most sites, and the retention rate was 66.46% (measured as the ratio of archives recording meteorological data to the total number of archives), representing a marked improvement in archival density. The proliferation of local gazetteers during these centuries provided more detailed information on regional climate. Enhanced source availability facilitated the implementation of stricter classification criteria for extreme events, substantially improving the precision of severe-event identification while maintaining systematic consistency across regions. This incorporates explicit spatial coverage considerations within designated station areas while preserving more flexible standards for remote regions where documentary sources remain limited.

Severe Wet (SW) requires clear documentation of flood disasters, including comprehensive descriptions with precise timing, geographical scope, and governmental or social response measures. Extensive or prolonged heavy precipitation manifests through continuous heavy rainfall causing rapid river level rise and dam or embankment destruction, documented with historical phrases such as "incessant heavy rain" (霪雨不止), "river overflow" (河水泛溢), or "water depth several feet" (水深数尺), or through flood duration exceeding half a month within administrative boundaries exemplified by descriptions like "great flood did not recede for half a month" (大水半月不退), or when counties directly recording floods comprise more than one-fifth of total county-level administrative units within the station area. Urban infrastructure damage includes flood destruction of city walls, urban areas, or floodwater entering cities, documented through phrases such as "boats navigating within the city" (城中行舟), "swept away ten thousand homes" (漂没万家), or "destroyed dwellings" (荡庐

舍). Agricultural collapse and food crisis encompass complete crop failure accompanied by dramatic grain price increases, documented through formulaic expressions such as “flood, great famine” (水，大饥) or “great flood, no grain” (大水，无禾). Large-scale government intervention is evident in records of flood disasters, often accompanied by extensive tax exemptions or relief efforts coordinated at the prefecture or provincial administrative levels. Severe social disruption includes flood-induced social impacts encompassing large-scale population migration, exemplified by “many people relocated” (人多迁徙), population losses through descriptions such as “countless drowned” (溺死无算) or “people eating people” (人相食), social unrest, or displaced populations turning to banditry documented through phrases like “roving bandits appeared” (流寇出没) or “thieves swarmed” (盗贼蜂起).

Moderate Wet (MW) encompasses clear records of flood occurrence with simple descriptions such as “flooding in a certain month” (某月，涝) or “great flood in a certain place” (某地，大水), characterized by relatively short duration, crop yield reduction without complete agricultural failure exemplified by descriptions like “great flood, crops damaged” (大水，伤稼), and short-term heavy precipitation that destroys infrastructure such as bridges while causing no reported casualties.

Normal Years (N) are primarily documented through terms indicating agricultural success, including “great abundance” (大稔), “great prosperous year” (大有年), “good harvest” (熟), or “abundant” (稔). Note that not all missing values correspond to normal years; they only encompass years with no climate-related records when adjacent years demonstrate drought or flood documentation.

Moderate Dry (MD) includes clear records of drought occurrence without detailed disaster descriptions or explicit severity-indicating terminology, limited to simple location-based drought mentions such as “drought” (旱), “drought and famine” (旱、饥), “drought damage” (旱伤), or “great drought” (大旱). This classification also encompasses simple drought descriptions with fundamental severity indicators exemplified by “drought disaster and famine” (旱灾荒), “intercalary ninth month, great drought” (闰九月，大旱), “no grain in autumn” (秋无禾), or “drought in Taizhou prefecture counties” (泰州属县旱), individual counties within station areas recording “great drought”, and individual counties experiencing disaster-related delayed taxation or tax exemptions for current year grain, seeds, or taxes.

Severe Dry (SD) necessitates clear, detailed records of drought disasters. Extended regional drought manifests through prolonged drought within administrative boundaries exemplified by “summer-autumn drought” (夏秋旱) or “no rain from summer through autumn” (自夏徂秋不雨), drought causing water supply difficulties documented through descriptions like “wells and springs all dried up” (井泉俱竭), vivid landscape descriptions such as “scorched earth for thousands of li” (赤地千里), or when counties directly recording drought exceed one-fifth of total counties within the station area. Severe agricultural impacts and food crises encompass massive crop yield reductions or complete agricultural failures, accompanied by dramatic increases in grain prices that fundamentally disrupt local food security. Large-scale government relief is provided through the court or provincial-level administrative departments, offering extensive tax exemptions or coordinated relief efforts specifically targeting drought disasters. Severe social disruption includes drought-induced social impacts encompassing large-scale population migration exemplified by “many people relocated” (人多迁徙), population losses documented through descriptions such as “nine out of ten did not survive” (十不存一), “nine house-

holds out of ten empty” (十室九空), “countless starved to death” (饿死无算), “corpses of the starved filled the roads” (饿殍盈路), or “people eating people” (人相食), drought-induced social unrest and rebellions, or displaced populations turning to banditry as recorded through phrases like “roving bandits appeared” (流寇出没) or “thieves swarmed” (盗贼蜂起).

1.3.2 1649-1870 CE

This phase demonstrates enhanced source diversity, with data retention rate reaching approximately 80.00%. It incorporates expanded archival sources, comprehensive local gazetteers, and emerging newspaper coverage, providing the highest density of documentation among the periods considered here. The evaluation criteria adhered to the standards of the previous phase while accommodating the increased complexity of the source materials. However, the abundance of archival sources can lead to conflicting reports of simultaneous flooding and drought. Resolution of such conflicts follows systematic priority rules: (1) summer season reports receive precedence over other seasonal documentation, (2) more severe classifications take priority over moderate assessments, (3) appropriate contextual downgrades when evidence suggests mixed conditions, and (4) spatial proportion of affected areas within station boundaries guides final classification determination. This period also marks a crucial transition toward modern observational standards, as instrumental meteorological data began to appear after 1870 CE but became redundant only after the 20th century. In data processing, post-1870 instrumental observations, when available for extended periods exceeding twenty years, take methodological precedence using statistical analytical approaches. In contrast, shorter or incomplete instrumental records serve supplementary roles to traditional document-based assessments. Consequently, our analysis, which focuses on the period up to 1900 CE, is unaffected by measurement errors in the instruments.

1.4 Archive Examples

We have listed some examples of WDI grading determinations in Table S3. In the main text, we noted that historical materials often include comparisons with previous events. In Table S4, we listed some excerpted examples.

Table S3 – Examples of WDI grading determinations.

Year	Site and Level	English Translation	Chinese
1482 CE	Beijing, SW	In October, during the jia-shen day, due to flooding, more than 3,390 Shi of grain and over 44,120 bundles of fodder were exempted in Longqing Prefecture and Yongning County of Zhili.	十月甲申，以水灾免直隶隆庆州并永宁县粮三千三百九十余石，草四万四千一百二十余束。
1486 CE	Shaanxi, Baoji, SD	In northwest Shaanxi and Baoji, there was a severe drought in the second month of winter. People exhausted their stored grain, fleeing over 70,000 individuals.	陕西连岁大旱，百姓流亡尽，人相食。成化二十一年至二十二年，关中莲岁大旱，百姓流亡殆尽，人相食，十七八九。
1490 CE	Fenyang, MD	Famine, donated money for relief, and many people depended on it.	岁饥，输金赈济，人多依赖焉。
1519 CE	Ankang, SW	In summer, the Han River flooded, sweeping away many households.	夏，汉江水涨，漂没庐舍甚众。
1556 CE	Fenyang, N	Great harvest.	大稔。
1690 CE	Tianjin, SW	(1) Great flood. (2) In the fifth month of summer, there was continuous rain for ten days; water surged from Mount Pan, carrying rocks and splitting mountains. (3) Great flood; both people and crops were submerged. An edict was issued to open the granaries for relief and exempt taxes. (4) Great flood; an edict was issued to open the granaries for relief and exempt taxes. (5) Drought and locusts became a disaster.	(1) 大水。(2) 夏五月，霪雨十日，盘山水涌，挟石裂山。(3) 大水，民禾俱没。诏发库赈之，并免租。(4) 大水，诏发库赈之，并免租。(5) 旱蝗成灾
1708 CE	Handan, MD	(1) In the summer, there was a great drought; it did not rain until August, and all the grain withered. (2) The harvest failed. (3) Drought and locusts.	(1) 夏大旱，至八月始雨，禾尽稿。(2) 岁不登。(3) 旱，蝗。
1724 CE	Linfen, SD	(1) There was a severe drought in Pingyang, no wheat grew, and a dou of rice cost eight to nine qian. (2) That year there was a great drought, no wheat grew, the people suffered from severe hunger, and a dou of rice cost eight to nine qian of silver. Officials provided relief with blood donations and also granted partial exemptions from taxes and grain levies. (3) Due to the drought and famine, relief funds were distributed widely, saving many lives.	(1) 平阳署属大旱无麦，斗米至八九钱。(2) 是岁大旱，无麦，民大饥，斗米银八九钱。通官赈血，仍蠲免钱粮有差。(3) 旱荒，发赈账济，全活甚众。

Year	Site and Level	English Translation	Chinese
1795 CE	Beijing, MW	(1) Rain in May. (2) In September, accumulated water soaked the grain. Yongding, Baoding, and Hejian prefectures were flooded, exempting rent and reducing land taxes. Transported rice by 4.75 million stones to compensate the flat crop.	(1) 夏五月，雨。(2) 九月葵未，诏顺天、保定、河间、永平四府水涝伤稼，免明年地丁钱粮，仍运米四万石前往平粜。

Table S4 – Examples of self-comparison in historical climate records.

Year	English Translation	Chinese
1746 CE	“Another edict stated: ‘According to Acting Director-General of the Grand Canal, Gu Cong, this year’s canal waters rose higher than in Yongzheng Year 8. The river works were in danger in many places; I urgently ordered officials to guard day and night and take all precautions. Now the waters have receded and the embankments remain secure.’This year’s canal freshet was greater than in previous years; although the midsummer freshet has passed, the autumn freshet is imminent.”	（丁酉），又谕：“据署河道总督顾琮奏称‘今年运河水涨，较雍正八年为大。河工在在危险，臣飞饬道厅等官，昼夜抢护，竭力防范。今水势消退，保固无恙’等语。今年运河汛水，较往年盛涨，目今伏汛虽过，而秋汛踵至。”
1746 CE	This year the Tao River rose and swelled more than in previous years.	今岁洮水涨发，较往年为大。
1761 CE	Moreover, this spring and summer, timely rains have been plentiful; unlike previous years, river waters should be abundant.	况今岁春夏，时雨霑足，较往年大不相同，河水谅应充裕。
1764 CE	During this year’s late-summer and autumn freshet, the Wen River was not large; little water flowed in, and the level was more than two chi lower than in previous years.	今年伏秋汛内，汶水不大，進水无多，较往年少二尺餘寸。
1766 CE	This time the Yellow River rose for a prolonged period, somewhat greater than in previous years, leading to overflow; embankment works had to be urgently reinforced and were of the utmost urgency.	此次黄水长发，较往年稍大，以致漫溢，隄工抢筑，最为紧要。
1804 CE	Backflow into the Grand Canal across the Huai–Yang region was more severe than in previous years.	淮扬一带运河，倒灌较往年为甚。

2 Supplementary Note 2: Tree-ring proxy data sources and integration

2.1 Paleoclimate Proxy Data Sources

Our analysis incorporates high-resolution tree-ring-based hydroclimatic reconstructions from northern China, including summer precipitation and the Yellow River’s runoff. The dataset selection was based on the following criteria: (1) annual resolution maintained throughout the study period; (2) adequate sample replication with expressed population signal values exceeding 0.85 and correlation exceeding 0.4; (3) statistically significant calibration statistics against instrumental records where available; (4) spatial distribution representative of regional climate variability patterns, and; (5) exclude those with an obvious chronological relationship, such as later studies that used all the materials from previously published research. All datasets except one (Table S8) were obtained from the NOAA/NCEI World Data Service for Palaeoclimatology, ensuring standardised quality control, peer-reviewed validation, and comprehensive metadata documentationThis approach guarantees reproducibility and maintains consistency with international palaeoclimate data standards.

Given the abundance of historical documents in the study area, many studies have integrated tree-ring data with

historical records to reconstruct hydroclimate. To ensure analytical independence in our research, we selected reconstruction datasets that use only natural proxy indicators for analysis in the main text. Here, we conducted tests under three different data combination scenarios (Table S5): (1) Pure-natural: datasets obtained through screening that only use natural proxies, (2) Best-correlated: a subset of the pure-natural scenario, using datasets that achieve the best correlation during the validation period (1901–2000 CE). (3) All-mixed: using all datasets that meet our screening criteria, though some of the natural proxy data were integrated with certain historical documents. The dataset used in the main analysis is the pure-natural scenario, but others are also examined for robustness (see below).

Table S5 – Tested scenarios for screening different hydroclimate reconstruction data.

Scenario	Included Dataset (Referring to the datasets in Table S8)	n datasets
Pure-natural	Li 2015; Li 2019; Liu 2020; Xu 2023; Yang 2019	5
Best-correlated	Li 2015; Xu 2023	2
All-mixed	All available datasets in the Table S8	9

For the integrated natural proxy indicator data, we utilised three instrumental datasets (China[4], GPCC[5], and CRU[6]) to validate and assess the robustness of our data aggregation. The results show that all data combination scenarios exhibit good correlation under all validation data tests (Figure S4). Since the Chinese dataset is generally well correlated with historical material sites (Table S6), we used the results of this scenario in the main text.

2.2 Robustness test for different natural datasets

We conducted experiments under all three data combination scenarios. Results show that the patterns identified are also present in all three cases (Table S6 and Figure S5).

After excluding each proxy indicator dataset from the data integration process, we also calculated the impact on the correlation coefficient during the validation period and the final results (improvement of correlation and the optimal sliding window size). The results show that when removed, none of them substantially alters our findings (Table S7).

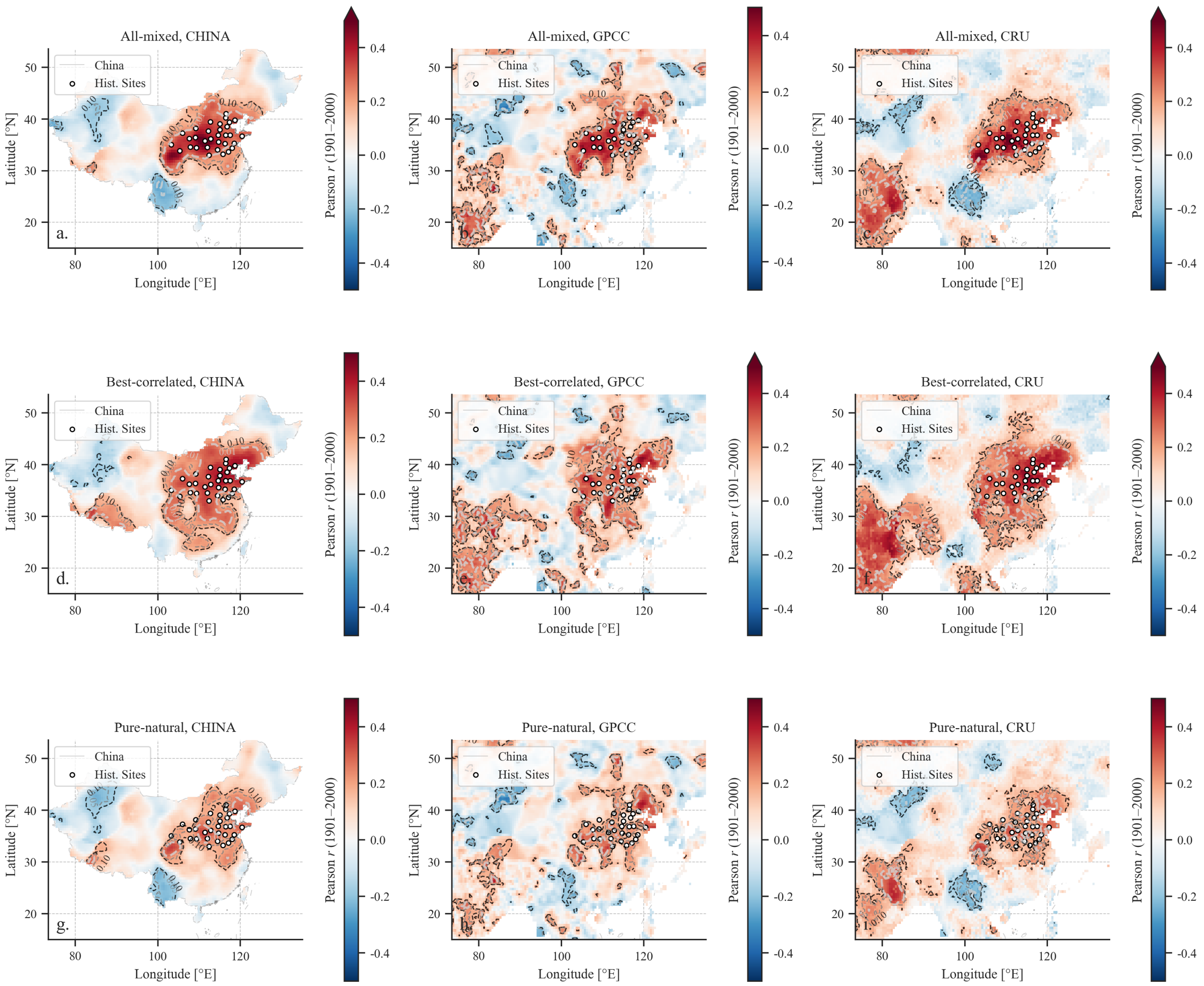

Figure S4 – Spatial correlations between the N-WDI and three gridded instrumental datasets over the validation period (1901–2000 CE, n = 100 years). Rows are data scenarios: (a.–c.) All-mixed; (d.–f.) Best-correlated; (g.–i.) Pure-natural (Table S5; reconstructions in Table S8). Columns are validation datasets: China[4], GPCC[5] and CRU[6]. Colours show Pearson's r at each grid cell; dashed contours mark two-sided p = 0.10 (black), 0.05 (grey) and 0.01 (light grey), not adjusted for multiple comparisons. Circles mark the 32 historical-archive sites (Table S2); the number of sites significant at $p < 0.05$ is given in Table S6. The national boundary is from the Standard Map Service of the Ministry of Natural Resources of China (approval number GS(2020)4619), used without modification.

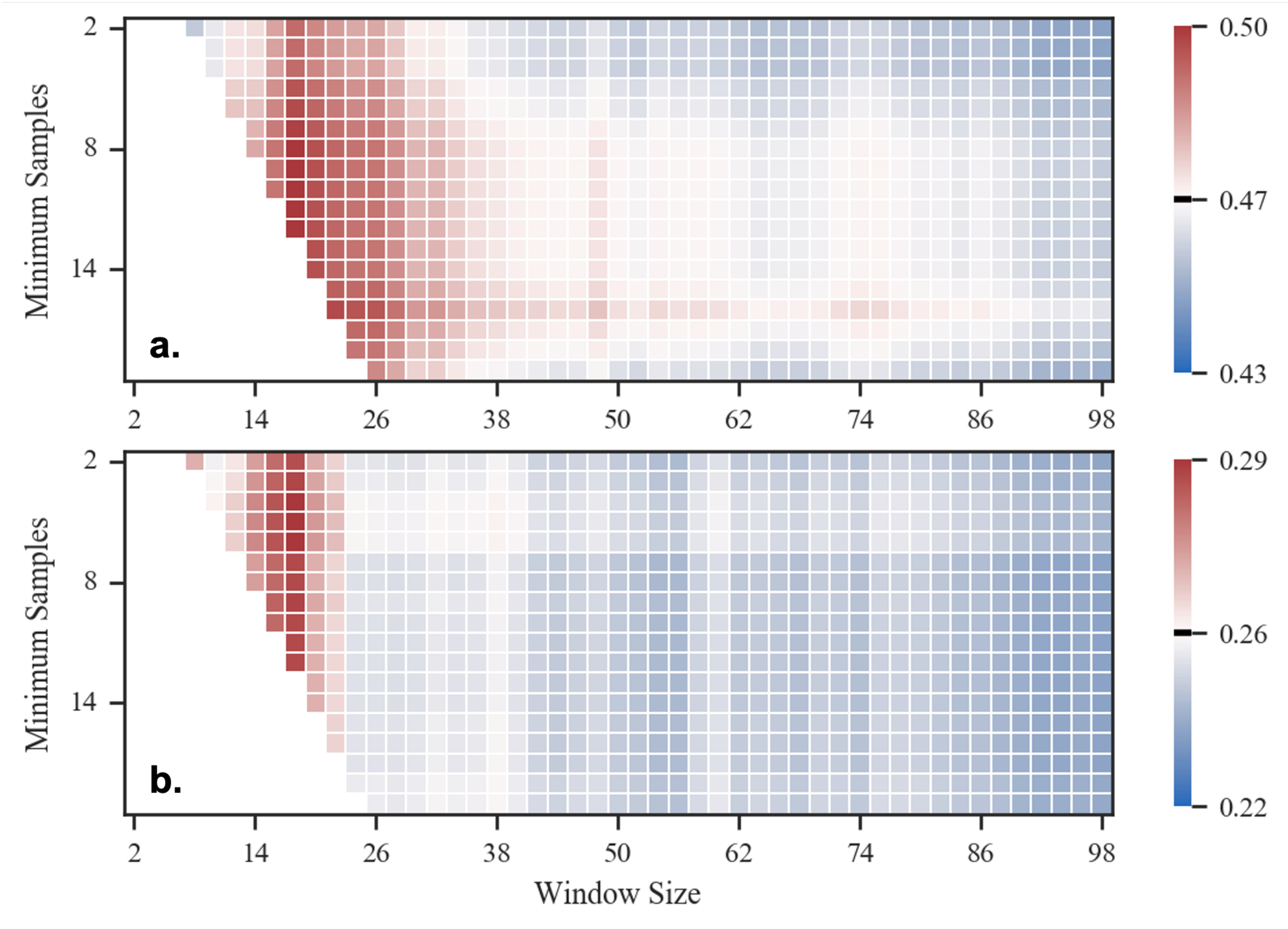

Figure S5 – Re-standardisation also increases the correlation under the two other natural-proxy scenarios (Table S5). a. All-mixed scenario; b. Best-correlated scenario. Colour, Kendall's τ between the H-WDI and the re-standardised N-WDI for each window size w (x-axis, years) and minimum sample size m (y-axis); the black tick on each colour bar marks the whole-period benchmark τ without re-standardisation (red, increase; blue, decrease; blank, $m > w$). n = 208 H-WDI years before re-standardisation; years whose window holds fewer than m values are dropped. As in the pure-natural scenario of the main text (Figure 4b), correlations peak at intermediate windows.

Table S6 – Main results under different data integration scenarios.

Data scenario	Val. data	Val. Corr.	Val. Corr. p	Sites with $p < 0.05$	Corr. Improvement^{a}	Optimal w
Pure-natural	China	0.35	< 0.001	19/32 (59%)	5.37%	33
	GPCC	0.37	< 0.001	18/32 (56%)		
	CRU	0.35	< 0.001	20/32 (63%)		
Best-correlated	China	0.52	< 0.001	30/32 (94%)	14.00%	26
	GPCC	0.52	< 0.001	27/32 (84%)		
	CRU	0.52	< 0.001	29/32 (91%)		
All-mixed	China	0.54	< 0.001	32/32 (100%)	7.36%	22
	GPCC	0.55	< 0.001	26/32 (81%)		
	CRU	0.53	< 0.001	31/32 (97%)		

Val. Corr. is Pearson's r between the N-WDI and the regional instrumental series over the validation period (1901–2000 CE, n = 100 years), reported with the two-sided p of the corresponding t test (df $= 98$); all $p < 0.001$. Sites counts the 32 historical-archive sites whose nearest grid cell correlates with the N-WDI at two-sided p < 0.05 (Pearson test, n = 100 years, not adjusted for multiple comparisons). [a] Improvement of correlation for the whole period (corresponding to the main text Figure 4b); optimal w is the mean optimal window across 200-year segments (main text Figure 4c).

Table S7 – Removing any input data will not have more than a 15% impact.

Datasets	Changes of	Changes from	Changes from	Changes from
	Corr. *a	All-mixed *b	Pure-natural	Best-correlated
Li2015[7]	-0.0244	0.01 (19)	0.0 (31)	0.01 (23)
Xu2023[8]	-0.0976	0.02 (19)	0.0 (32)	-0.02 (42)
Li2019[9]	0	0.02 (18)	0.03 (26)	
Liu2020[10]	0.0732	0.05 (17)	0.05 (34)	
Yang2019[11]	0.1248	0.0 (21)	0.0 (28)	
Yi2010[12]	-0.0732	-0.03 (24)		
Yang2016[13]	-0.0732	0.0 (17)		
Yi2012[14]	-0.1463	0.0 (19)		
Tan2011[15]	-0.0244	0.01 (19)		

[a] Pearson Correlation Coefficient, calculated by comparing the China validation dataset. [b] Changes in Kendall's τ after re-standardisation (and the optimal window size).

Table S8 – Natural-proxy reconstructions used in this study.

Dataset	Proxy Types	Variable	Number of Sampling	Period	Validation (as reported)	Data source
Li2019[9]	Tree-ring	streamflow	68 chronologies	800 - 2010 CE	r = 0.41–0.76 (p not reported)	NOAA/NCEI study 26230
Liu2020[10]	Tree-ring	natural runoff	31 chronologies	1492 - 2013 CE	r = 0.76, $p < 0.001$	Digitised from the published figure
Xu2023[8]	tree ring, stable oxygen isotope	precipitation index	128 trees, 6 tree ring cellulose oxygen isotope chronologies	1588 - 2013 CE	r = 0.78 / 0.65 (p not reported)	NOAA/NCEI study 37739
Yi2012[14]	Tree-ring, documents*		92 chronologies from 46 trees (58 chronologies from 29 trees in Luya; 34 chronologies from 17 trees in Huashan)	1470 - 2002 CE	r = 0.436–0.660 (p not reported)	NOAA/NCEI study 10769
Yang2019[11]	Tree-ring	summer (May-June) moisture	435 cores from 243 trees	455 - 2011 CE	r = 0.63, $p < 0.01$	NOAA/NCEI study 29732
Yang2016[13]	Tree-ring, documents*	summer (May-Sep.)	39 chronologies	1470 - 2000 CE	average r = 0.66 (p not reported)	NOAA/NCEI study 19683
Li2015[7]	Tree-ring, stable oxygen isotope	precipitation anormaly	37 chronologies from 17 trees	1872 - 2003 CE	r = 0.72, $p < 0.0001$	NOAA/NCEI study 30272

**Dataset**	**Proxy Types**	**Variable**	**Number of Sampling**	**Period**	**Validation (as reported)**	**Data source**
Tan2011[15]	stable oxygen isotope, document*	precipitation index	NA	190 - 1980 CE	not reported	NOAA/NCEI study 13624
Yi2010[12]	Tree-ring, documents*	summer (June to August) precipitation	NA	1600 - 2000 CE	average r = 0.408 (p not reported)	NOAA/NCEI study 9903

* In the process of reconstructing hydroclimate, these annotated datasets have already used some historical documents as a supplement to tree-ring data. Validation statistics are those reported in the source publications (not recomputed here). A range or a pair of values corresponds to several chronologies or to a calibration/verification split. Data source gives the NOAA/NCEI World Data Service for Paleoclimatology study identifier (https://www.ncei.noaa.gov/access/paleo-search/study/).

2.3 Bayesian integration of natural-proxy reconstructions

The screened reconstructions were merged into a single standardised natural WDI (N-WDI) with a Bayesian latent-variable model (PyMC 5.28.4; posteriors analysed with ArviZ 0.20.0). Each series was first standardised to a mean of 0 and a variance of 1. For each year t, an unobserved true anomaly θ_t received a weakly informative prior $\theta_t \sim \mathcal{N}(0,1)$, matching the unit variance of the standardised inputs, with no inter-annual smoothing, so that years without any reconstruction are governed by the prior rather than by data. Each standardised reconstruction i was modelled as a Student's t observation centred on the latent value, $y_{i,t} \sim \text{StudentT}(\nu,\ \mu = \theta_t,\ \sigma_{i,t})$, contributing a likelihood term only in years where it is observed (missing values were masked).

The Student's t likelihood was chosen for robustness to occasional reconstruction outliers. Rather than fixing its degrees of freedom, we placed on it the weakly informative $\nu \sim \text{Gamma}(2, 0.1)$ prior of Juárez and Steel[16] (prior mean 20), which leaves the tail weight to the data, and estimated a single shared ν jointly with the latent series; the posterior was $\hat{\nu} = 36$ (94% highest-density interval [18, 58]), i.e. tails only mildly heavier than Gaussian. This is consistent with an independent distribution fit of the integrated series, in which a normal and a Student's t are statistically indistinguishable and both rank first, the fitted t degenerating towards the normal (Table S9).

Table S9 – Distribution fit of the integrated annual N-WDI, ranked by AIC; normal and Student's t are indistinguishable, corroborating the near-Gaussian posterior for ν.

Distribution	Sum-sq. error	AIC	BIC	KS statistic	KS p-value
Normal	0.901	1835.76	1845.6	0.02	0.806
Student's t	0.901	1837.76	1852.53	0.02	0.806
Gamma	0.9012	1837.78	1852.55	0.0198	0.815
Log-normal	0.9026	1837.99	1852.75	0.0209	0.759

Fitted to the annual N-WDI posterior mean, 1000–2013 CE (n = 1,014 years). KS: two-sided one-sample Kolmogorov–Smirnov test against each fitted distribution; because the parameters are estimated from the same data, the KS p values are approximate.

The observation scale $\sigma_{i,t}$ was set from each series' local variability (a centred 10-year rolling standard deviation of the standardised series, clipped to 0.2–2 times its mean), so that series or intervals with larger within-series variability receive lower effective weight. It is a fixed input rather than a parameter, so it has no prior, and it is distinct from the calibration statistics ($r > 0.4$) used as an inclusion criterion, which are not entered as model weights. Because standardisation is applied both on loading and within the integration routine, the second pass is close to the identity for the already-standardised inputs and only rescales the rolling uncertainties.

The model was sampled with four NUTS chains of 2000 draws each (1000 tuning steps, target acceptance 0.95). Convergence was satisfactory for all sampled parameters, including ν: the Gelman-Rubin statistic was $\hat{R} \leq 1.01$ throughout, and the minimum effective sample size exceeded 3,900. The reported N-WDI is the per-year posterior mean, and its uncertainty refers to the posterior credible interval of θ_t (median posterior standard deviation 0.40; median 94% HDI width 1.49), which widens in years where reconstructions disagree — a property a simple per-year average cannot provide. Main text Figure 2a shows it as the 5–95% interval (posterior mean ± 1.645 posterior standard deviations).

To test the sensitivity of the N-WDI to the priors, we refitted the model seven times, changing one prior at a time: $\theta_t \sim \mathcal{N}(0, 0.5)$, $\mathcal{N}(0, 2)$ or $\mathcal{N}(0, 10)$; $\nu \sim$ Gamma$(2, 1)$ or Exponential$(1/30)$; ν fixed at 4; or a Gaussian likelihood. All fits converged ($\hat{R} \leq 1.01$), and each refitted N-WDI replaced the baseline in the main-text analyses (Table S10).

The N-WDI was nearly unchanged ($r \geq 0.993$ with the baseline). The posterior of ν followed its prior (means 16–48), but fixing ν at 4 or removing the t tails did not alter the index. The narrowest θ_t prior, half the unit scale of the standardised inputs, moved 21% of years into another category. No conclusion changed: Kendall's τ between H-WDI and N-WDI stayed between 0.166 and 0.199 (all $p \leq 0.01$), and the optimal re-standardisation window between 28 and 35 years (baseline 33).

Table S10 – Sensitivity of the N-WDI and the main results to the priors.

Prior change	Posterior ν	r	Same class (%)	τ (H vs N)	κ (H vs N)	Optimal w (yr)
Baseline	36 [18, 58]	1.000	100	0.176	0.20	33
θ ~ N(0, 0.5)	28 [15, 45]	0.993	79	0.199	0.22	35
θ ~ N(0, 2)	30 [16, 49]	0.998	91	0.183	0.22	34
θ ~ N(0, 10)	26 [14, 41]	0.996	87	0.183	0.22	33
ν ~ Gamma(2, 1)	16 [12, 20]	1.000	98	0.166	0.19	34
ν ~ Exp(1/30)	48 [18, 93]	1.000	99	0.174	0.20	33
ν = 4 (fixed)	–	0.996	95	0.173	0.20	28
Gaussian likelihood	–	1.000	99	0.182	0.21	34

Each row changes one prior relative to the baseline ($\theta_t \sim \mathcal{N}(0, 1)$, $\nu \sim$ Gamma$(2, 0.1)$, shape–rate). Posterior ν: mean [94% HDI]. r and Same class: agreement of each N-WDI with the baseline, as Pearson's r and the share of years in the same five-level class. τ, κ (weighted) and optimal w are recomputed as in the main text (H-WDI vs N-WDI, 1470–1900 CE; w averaged over 200-year segments).

3 Supplementary Note 3: ODD+D protocol of the agent-based model

3.1 Overview

3.1.1 Purpose

The purpose of this agent-based model is to investigate the mechanistic basis for systematic temporal patterns observed in historical climate documentation, specifically addressing how individual cognitive biases and bounded memory aggregate to produce collective memory dynamics at generational timescales. The model is designed for researchers studying historical climate records, cognitive biases in environmental perception, and the interface between individual psychology and collective knowledge formation. By simulating populations of climate observers with realistic memory constraints and psychological biases, the model aims to explain the empirical finding that correlations between historical climate and humans' records of it are not well-matched due to Shifting Baseline Syndrome (SBS).

3.1.2 Entities, State Variables, and Scales

The model contains two primary entity types. Climate observers represent individual agents who perceive, evaluate, and selectively record climate events based on their personal experience and cognitive biases. Each observer is characterised by age (from 1 to the maximum lifespan), a bounded personal memory implemented as a "first-in–first-out (FIFO)" queue storing recent climate experiences up to the current age, a minimum age for recording eligibility (default 10 years), and a maximum lifespan (default 40 years). The climate environment generates a stationary stochastic time series representing regional climate variability. In the main analysis, this follows an AR(1) autocorrelated process, $c_t = \varphi \cdot c_{(t-1)} + \varepsilon_t$, with persistence $\varphi = 0.5$ and innovations scaled to unit variability ($\sigma_c = 1$); independent Gaussian noise ($\varphi = 0$) and linear-trend variants are retained as robustness alternatives (`climate_process` ∈ {iid, ar1, trend_plus_noise, ar1_trend}). The collective archive stores recorded climate events by simulation year and computes an integrated yearly estimate by mapping discrete levels {SD, MD, N, MW, SW} (see Figure S3) to truncated-normal draws within their threshold intervals and averaging the resulting samples (uncertainty-aware integration with one draw per level by default).

Space is implicit (regional scale, no spatial heterogeneity). Time advances in annual ticks. The analyzable output typically spans a configurable number of years (e.g., 100–300) following a spin-up calculated as `spin_up_years` = `new_agents` × (`max_age` − `min_age` + 1). The population scale is dynamic, with new agents entering each year and observers being removed when they exceed their maximum lifespan. The default configuration in the current implementation allows 5 new agents per year; these values are fully configurable via the run configuration. The default `max_age = 40` approximates the adult recording lifespan of an observer in this historical context (roughly the working/adult span of a generation) and is consistent both with the life expectancy at birth estimated for historical China[17] ($e_0 \approx 30$–40 years; based on multiple independent sources[?,?,?,?,?,?], including Genealogy, Banner register, Imperial lineage, Retrospective survey, and global references for pre-industrial values in England, Sweden, and Japan (Figure S6)). Because the sensitivity analysis varies `max_age` over 15–80 years, the emergent optimal window scales with it.

3.1.3 Process Overview and Scheduling

Each simulation year follows a standardised sequence. The model advances the global clock. New observer agents enter with age one and empty memories. All active agents then: (i) increment age; (ii) append the current climate value to personal memory; (iii) if above the minimum recording age, perceive the current climate as a standardised deviation relative to a configurable baseline (`memory_baseline` ∈ {`personal`, `collective`, `collective_lifetime`, `model`}; see Table S11 for the 2×2 baseline design) and probabilistically decide whether to record; and (iv) transmit the record to the archive with probability (1 − `loss_rate`). Integration computes the yearly collective estimate via truncated-normal mapping and averaging. Observers exceeding maximum lifespan are removed. After completion, the model computes rolling-window correlations between the collective series and the underlying climate using a standard-deviation filter applied to the natural climate series used as the objective reference.

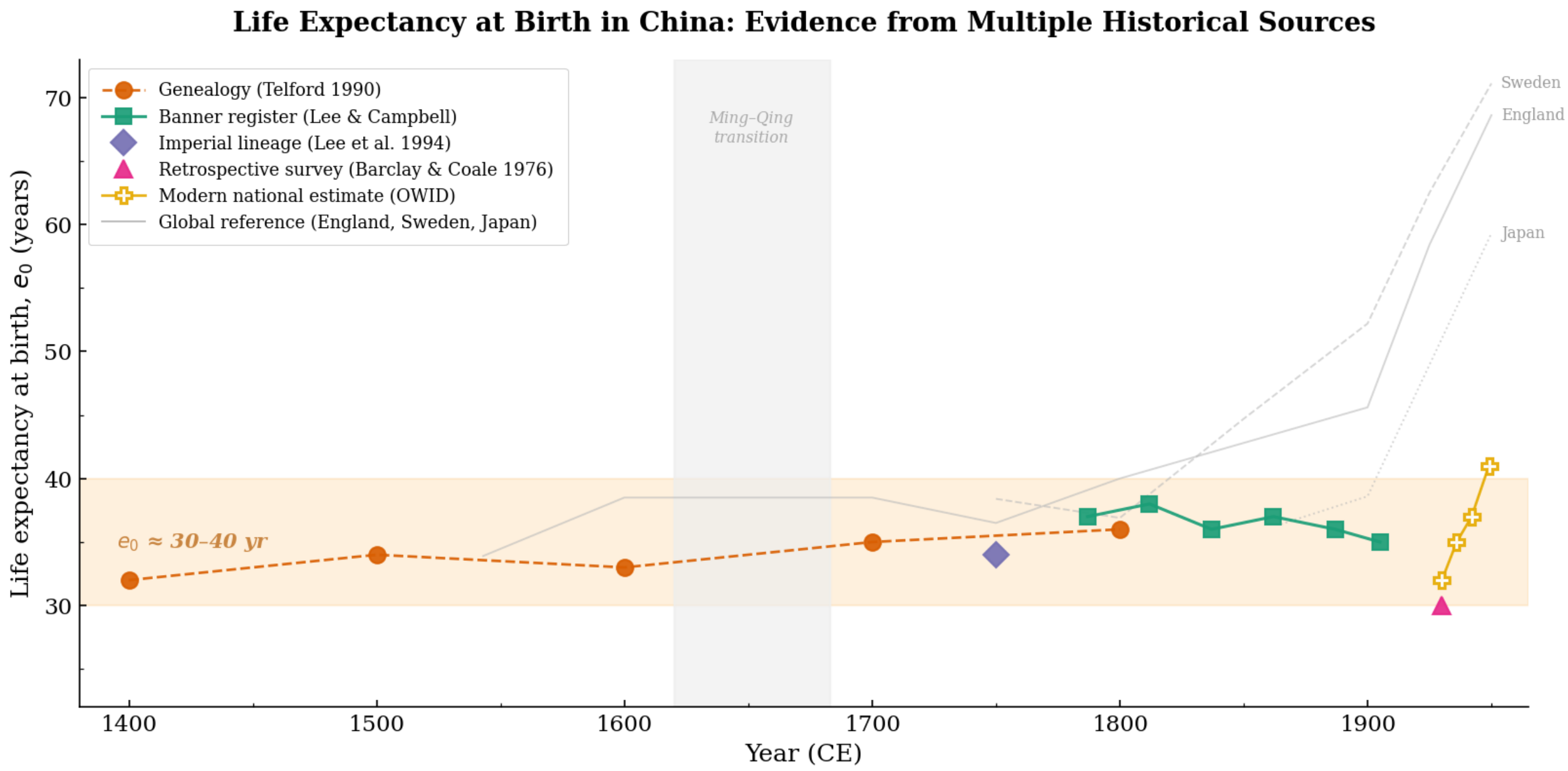

Figure S6 – Life expectancy at birth (e_0) in historical China compiled from multiple independent historical sources (genealogies, banner registers, imperial-lineage and retrospective-survey estimates), with pre-industrial reference series for England, Sweden, and Japan shown in grey. Chinese estimates remain within $e_0 \approx 30$–40 years (shaded band) across 1400–1900 CE, consistent with the 20–40-year generational window recovered from the archives and with the ABM's maximum lifespan of 40 years.

3.2 Design Concepts

3.2.1 Theoretical and Empirical Background

The model builds upon established theories from cognitive psychology and social memory research. A theoretical foundation rests on negativity bias, the well-documented psychological tendency to allocate greater attention and memory resources to negative or extreme events compared to neutral conditions. In the climate context, this manifests as a preference for recording drought and flood events over normal precipitation years[18,19]. The model implements this through a smooth probability function in which the recording likelihood increases monotonically with the absolute magnitude of the perceived climate deviation, avoiding arbitrary thresholds while preserving a bias toward extremes.

The second theoretical pillar is the SBS (collective scale) and anchoring effect (individual scale)[20,21], which captures how current assessments are influenced by similar past experiences serving as reference points. The current implementation focuses on perception relative to baselines without any retrospective adjustment mechanism. The model achieves this by setting up a baseline comparison for each agent before recording. Four baselines are implemented; they are most easily understood as a 2×2 design that crosses two binary factors (Table S11) (i) the source of the remembered baseline (the agent's own experience vs. the shared collective archive) and (ii) its temporal horizon (restricted to a single agent-lifetime, i.e. generational, vs. the full elapsed history, i.e. cumulative):

Table S11 – The 2x2 baseline scenario matrix of the ABM, crossing the source of the remembered baseline (the agent's own experience vs the shared collective archive) with its temporal horizon (a single agent-lifetime vs the full elapsed record). The diagonal cells are the two main-text hypotheses (personal, H1 generational amnesia; collective, H2 collective illusion), while collective_lifetime and model are the decomposition and non-perceptual benchmark controls.

	**Generational horizon (one agent-lifetime)**	**Cumulative horizon (full elapsed history)**
Individual source (own memory)	personal — H1: generational amnesia	(idealised limit → model, see below)
Collective source (shared archive)	collective_lifetime	collective — H2: collective illusion

The two cells reported in the main text are the diagonal hypotheses. Under `personal` (H1), each agent re-standardises the current year against the mean and standard deviation of only its own lived memory M_i (bounded by its lifespan). Under `collective` (H2), every agent instead uses the mean and standard deviation of the entire accumulated archive $\{e_1, \cdots, e_t\}$. The two baselines not foregrounded in the main text decompose these two factors:

- `collective_lifetime` shares a row with `collective` (it draws on the shared archive) but a column with `personal` (its window is bounded to the observer's own lifetime, $[t - age, t]$). It therefore dissociates the horizon factor from the source factor: if the unimodal, generation-scaled correlation pattern arises from a bounded memory horizon rather than from individual vs collective pooling, `collective_lifetime` should reproduce H1; if it instead requires individual experience, `collective_lifetime` should behave like H2. It thus serves as a control that identifies which of the two factors drives the empirical pattern.
- `model` fills the remaining (individual × cumulative) corner in its idealised limit: an observer whose reference spans the entire record with no perceptual filtering. Mechanically, it sets the baseline to the mean and standard deviation of the objective climate forcing c_t itself, rather than of any recorded series, so it carries no Shifting-Baseline mechanism. It serves as a non-perceptual benchmark that bounds the attainable correlation when no baseline drift is present, assuming the observer has full access to the true information.

Only the diagonal H1/H2 contrast is foregrounded in the main text; `collective_lifetime` and `model` are reported here as decomposition/benchmark controls, and their full correlation profiles are presented with the robustness analyses below (Figure S8). The empirical basis for the model is derived from analyses of historical climate records from northern China (1470–1900 CE), tree-ring reconstructions (validated for 1901–2000 CE), and the psychological literature on cognitive biases in environmental perception (see main texts). This empirical phenomenon inspires the model, but no parameters are derived from data analysis. Therefore, conceptually, this is a heuristic model intended to analyse potential mechanisms of SBS.

3.2.2 Individual Decision-Making

Decision-making occurs at the individual agent level, with each observer independently evaluating whether to record the current year based on their personal perception of the climate. The basic rationality assumes that agents are more likely to document years that appear unusual relative to their own experience, without explicit optimisation objectives or success criteria beyond this general tendency. Agents make decisions through a two-stage process: first, computing a z-score indicating how the current climate deviates from their personal memory; then, drawing a Bernoulli trial with probability determined by a survival function that assigns higher likelihoods to extreme deviations.

Agents adapt their behaviour to changing endogenous conditions by accumulating climate experiences in bounded memory, gradually shifting their baseline for assessing extremeness as they age. When the retrospective module is active, agents also respond to exogenous collective memory by comparing current conditions with historical precedents, potentially adjusting their initial classification based on inter-annual climate differences. Social norms and cultural values are not explicitly modelled, though the shared tendency toward negativity bias represents a form of common psychological disposition. Spatial aspects play no role in the current implementation, whereas temporal elements are central, operating through both personal memory accumulation and optional reference to collective historical precedents.

3.2.3 Learning

Individual learning is not implemented in the traditional sense of updating decision rules based on outcomes. Instead, agents exhibit experiential learning through memory accumulation, in which expanding experience gradually alters their baseline for assessing current conditions without changing the underlying decision algorithm. This represents implicit adaptation to changing conditions through experience rather than explicit rule modification. Collective learning occurs indirectly through the shared archive, where the accumulated decisions of past agents influence current agents through the retrospective re-judgment mechanism, creating a pathway for collective knowledge to shape individual decisions without direct agent-to-agent communication.

3.2.4 Individual Sensing

Agents sense only the current climate value and perceive it as a standardised deviation relative to the selected baseline — personal memory, the collective archive (over the full history or windowed to the observer's lifetime), or the objective model climate series (`memory_baseline` ∈ {personal, collective, collective_lifetime, model}). Perception thus yields subjective, experience-dependent assessments rather than objective measurements. The spatial scale of sensing is irrelevant in the current spatially-implicit implementation.

3.2.5 Individual Prediction

Agents do not employ explicit internal models for predicting future conditions. Instead, they exhibit implicit expectations that the future climate will resemble their personal experience, which influences their assessment

of current extremeness through the baseline established by personal memory. Prediction errors are not explicitly modelled, though the bounded and experience-dependent nature of personal memory creates systematic differences between agent expectations and objective climate statistics that could be interpreted as prediction biases.

3.2.6 Interaction

Direct interactions between agents do not occur. Indirect interaction is possible through the shared collective archive. In its current configuration, the archive primarily serves as an analytical tool and does not influence individual decisions.

3.2.7 Collectives

Individual agents form a collective by integrating their recordings into a shared archive. The collective is represented as a time series of yearly assessments, computed by mapping discrete levels to truncated normal samples within threshold intervals and averaging. This preserves ordinal structure while enabling uncertainty-aware aggregation. In prospective extensions with re-judgment, the archive may influence individual decisions by providing precedents. When agents need to perceive the current severity of extreme climate using historical archives (the H2: collective illusion scenario), individual decision-making is influenced by stored collective knowledge.

3.2.8 Heterogeneity

Agents exhibit heterogeneity in age, resulting in a continuous distribution ranging from newborn agents to those approaching maximum lifespan. This age heterogeneity translates into differences in personal memory content and length, as agents born at different times experience different climate sequences and accumulate varying amounts of experience. Decision-making processes are identical across agents, with heterogeneity arising from different personal histories rather than different decision algorithms. Stochastic components in recording and re-judgment decisions also create behavioural variation among agents experiencing identical conditions.

3.2.9 Stochasticity

Climate generation follows a stationary AR(1) process in the main analysis (independent-noise and linear-trend variants are tested for robustness). Recording decisions are stochastic via the propensity function. Collective integration adds randomness via truncated normal sampling when mapping levels to continuous values. Whether agents record an extreme event perceived by an agent is random, governed by a smooth probability function in which the likelihood of recording increases monotonically with the absolute magnitude of the perceived climate deviation. Record transmission to the archive has stochastic loss with probability `loss_rate` (default 0.40), a per-record Bernoulli trial applied independently to each record.

3.2.10 Observation

Data collection focuses on the primary output of collective climate records, which is computed as the truncated normal–mapped average of individual recordings for each year. Additional metrics include the window-by-Kendall's tau profile against the underlying climate using a rolling standard-deviation filter applied to the N-WDI. Key emergent properties include systematic temporal patterns in correlation strength, consistent with generational timescales emerging from bounded memory.

3.3 Details

3.3.1 Implementation Details

The model is implemented in Python 3.11 using the ABSESpy 0.11.5[22]. Core dependencies include NumPy 2.4.4, Pandas 3.0.2, and SciPy 1.17.1; scikit-learn 1.8.0, xarray 2026.4.0, PyMC 5.28.4, Geopandas 0.14.4. Model utilities implement classification thresholds and truncated-normal mapping for ordinal levels. Structure and parameters are separated via configuration, enabling systematic exploration without code changes.

3.3.2 Initialization

The simulation begins with an empty population and generates a climate time series of length (spin-up + analysis years). New agents enter each tick with age 1 and an empty memory. The spin-up period equals `new_agents` × (`max_age` − `min_age` + 1) (e.g., 5 × (40 − 10 + 1) = 155 years under the default settings), ensuring demographic stability before analysis. During spin-up, outputs are discarded.

3.3.3 Input Data

The model requires no external climate data. Key configurable parameters include maximum age (default 40), minimum recording age (default 10), new agent entry rate (default 5 per year), simulation duration (default 100 analysis years), record loss rate (default 0.40), and the perception baseline (`memory_baseline` ∈ {personal, collective, collective_lifetime, model}, default personal). Correlation analysis parameters include the window size range (2–99 years), the minimum number of observations per window (default: 10), the correlation method (default: Kendall), and the filter side (default: True, on climate series).

3.3.4 Submodels

Climate classification discretises continuous climate into five ordinal categories using symmetric thresholds at −1.17, −0.33, 0.33, and 1.17 standard deviations. Perception computes a standardised deviation as (current − baseline_mean) / baseline_std, where the baseline is selected by `memory_baseline`. Recording probability follows P(record) = f0 + 0.5 − SF(|z|; scale), with SF the standard normal survival function, f0 the baseline probability (default 0.10), and scale controlling steepness (default 1.0). Collective integration maps levels to truncated normal draws within their threshold intervals and averages them to produce a yearly continuous estimate.

This continuous mapping serves only to smooth the across-observer yearly average; because the downstream comparison uses rank correlation (Kendall's τ), it affects only tie-breaking among equal level-averages, not the rank structure that drives the window-dependence. When replacing stochastic truncated-normal sampling with a deterministic midpoint mapping, the window–τ profile remains essentially unchanged — the peak window is identical and $|\Delta\tau| \leq 0.03$ across all windows, for both the empirical H-WDI/N-WDI comparison and the ABM collective archive.

3.3.5 Model Output and Analysis

Primary output consists of annual collective climate records aligned with the continuous climate. Validation analysis mirrors the empirical pipeline: Kendall's tau between collective records and climate after applying a rolling standard deviation filter to the climate series. Window sizes span 2–99 years. For the global sensitivity analysis, we quantify how five model parameters govern the location of the correlation peak (the optimal re-standardisation window). We use a variance-based Sobol analysis (Saltelli sampling, N = 1024 base samples; each parameter vector evaluated over 30 stochastic replicates), reporting first-order (S□) and total-effect (S_T) indices for maximum lifespan (max_age, 15–80 yr), the number of new agents per year (new_agents, 1–15), the archive loss rate (`loss_rate`, 0–0.8), climate variability (climate_sigma, 0.5–2.0), and climate persistence (climate_phi, 0–0.9). The analysis is run separately under each baseline. Under H1 (`memory_baseline` = `personal`), the peak-window location is governed almost entirely by agent lifespan (total-effect S_T ≈ 1 for max_age, with all other parameters near zero) and scales in direct proportion to it (Figure S7), whereas under H2 (`memory_baseline` = `collective`) no parameter reproduces a generation-scaled optimum, and the peak-window location instead tracks the climate's own persistence (Figure S7). This decomposition identifies a bounded individual lifespan as the single factor that determines the generational timescale of the emergent pattern (Figure 5d; see also Figure S8).

3.3.6 Robustness Analyses

First, we compared all four baselines of the 2×2 design (Table S11) under identical conditions (Figure S8). In all four `memory_baseline` options, only the `personal` baseline (H1) produces a unimodal window–τ profile that peaks within the 20–40-year generational band. The full-archive `collective` baseline (H2) increases monotonically with window length and shows no generational optimum, and the lifespan-windowed `collective_lifetime` control behaves like H2 rather than like H1. Because `collective_lifetime` shares H1's bounded horizon but draws on the shared archive rather than on lived experience, its failure to reproduce the optimum shows that the generational window arises from individual lived experience itself, not from a bounded memory horizon alone. The non-perceptual `model` benchmark, which uses objective climate forcing as its reference, lacks a Shifting-Baseline mechanism and imposes a limit on attainable correlation when no baseline drift is present.

Second, we re-ran the model under a climate-forcing robustness matrix that crosses three stochastic processes (i.i.d. Gaussian noise, AR(1) persistence, and a linear trend with noise) with two temporal resolutions (annual and sub-annual at four steps per year aggregated back to annual units), evaluating each combination over multi-

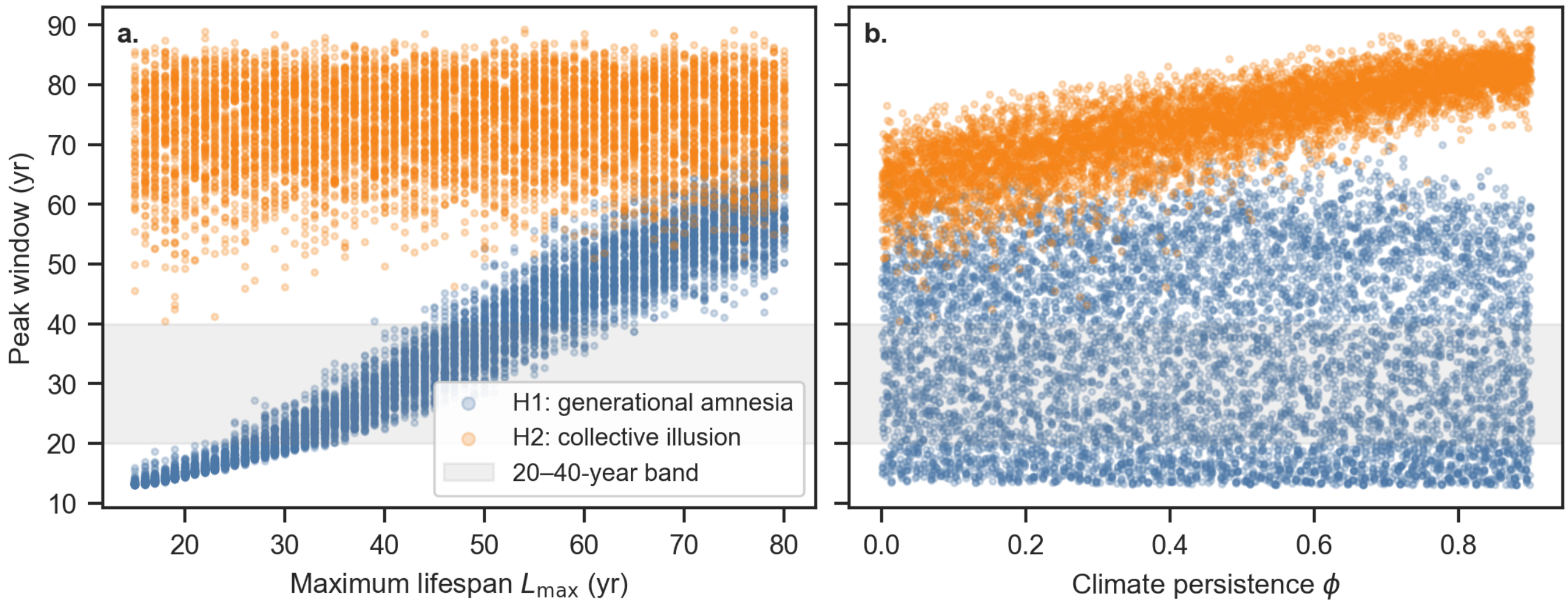

Figure S7 – Drivers of the peak (optimal) re-standardisation window in the two diagonal scenarios. (a) Peak window versus agent maximum lifespan: under H1 (personal baseline, blue) the peak window increases in direct proportion to lifespan and passes through the 20–40-year generational band (shaded) at the empirically plausible maximum lifespan of ≈ 40 years, whereas under H2 (collective baseline, orange) it stays at much longer windows and is essentially independent of lifespan. (b) Peak window versus climate persistence (φ): under H2 the peak window rises with climate persistence (the collective baseline merely tracks the climate's own autocorrelation), whereas under H1 it is insensitive to φ. Each point is one Sobol parameter set (n = 7,168 per scenario from N = 1,024 base samples), with the peak window averaged over 30 stochastic runs. Together the panels show that the generational timescale under H1 is set by individual lifespan, not by the statistics of the climate forcing.

ple stochastic replicates (Figure S9). Across all six combinations, the emergent optimal window remains within the 20–40-year generational band, so the result remains robust on different climatic assumptions.

3.4 Additional Elements (ODD+D)

3.4.1 Model Testing and Validation

Component testing verifies the expected behaviour of individual submodels, including climate classification accuracy, perception model calculations, and recording probability functions. Sensitivity analysis examines model behaviour across parameter ranges, with particular attention to maximum age effects (tested from 15 to 80 years), retrospective module activation, and recording probability settings. Empirical validation compares model outputs with the mismatch pattern between historical archives and natural proxy-based climate records from North China, focusing on correlation patterns and optimal window sizes rather than on absolute correspondence with specific historical events.

3.4.2 Model Replication

Complete model code and configuration files are available in the projects GitHub repository to ensure reproducibility, with controlled random seed specification enabling exact replication of results. All parameters are documented with default values, acceptable ranges, and behavioural interpretations. The implementation design

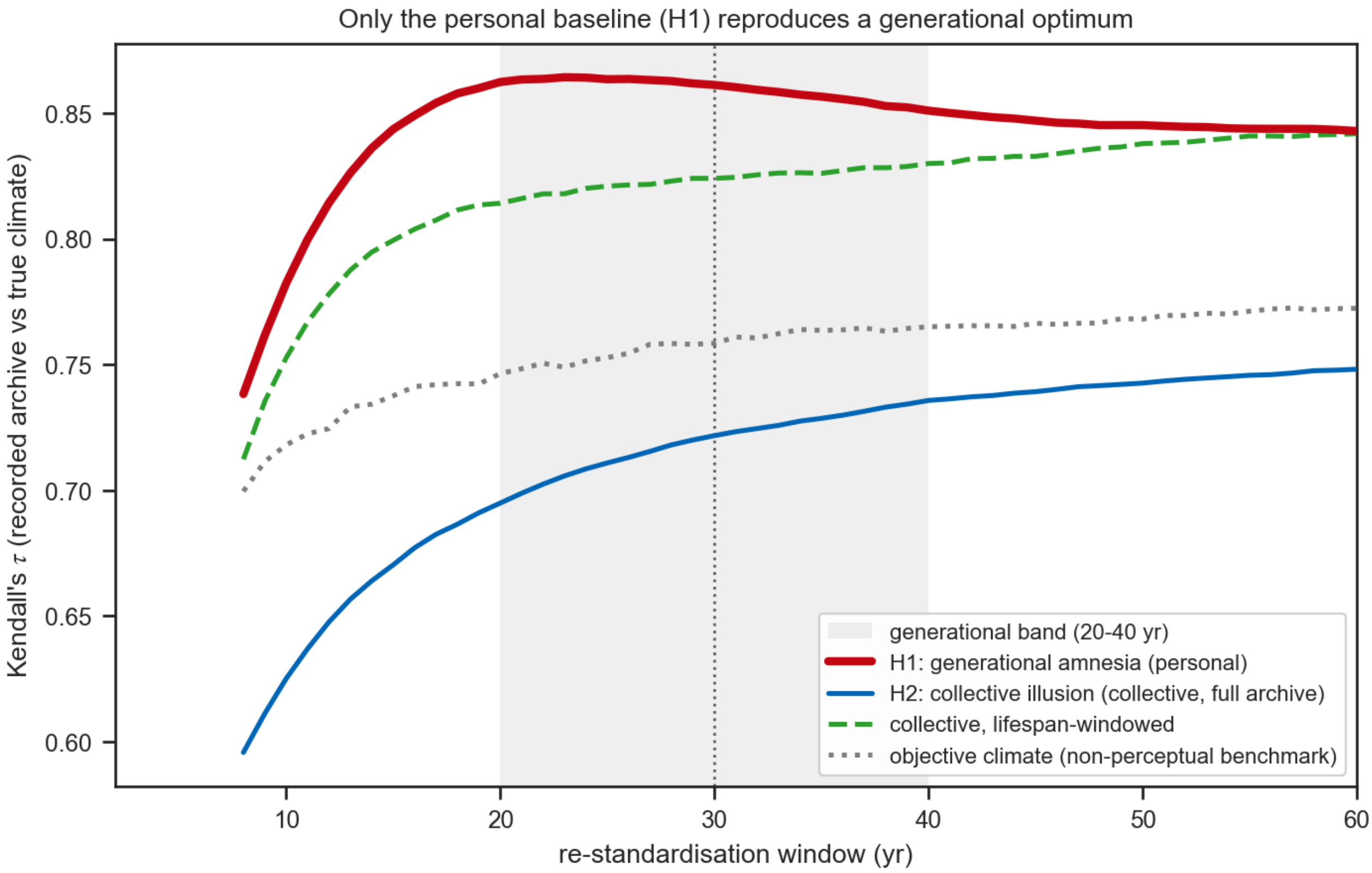

Figure S8 – Window–τ correlation profiles for all four baselines of the 2×2 design, each averaged over replicate runs under the AR(1) main forcing (n = 100 runs for H1 and H2, 24 for the lifetime-bounded collective control and 10 for the objective-climate benchmark). Only the personal baseline (H1: generational amnesia, red) peaks within the 20–40-year generational band (shaded; dotted line at 30 yr). The full-archive collective baseline (H2: collective illusion, blue) rises monotonically with window length; the lifetime-bounded collective control (green dashed) tracks H2 rather than H1 despite sharing H1's bounded horizon; and the non-perceptual objective-climate benchmark (grey dotted), referenced to the objective climate, shows no generational peak. The contrast isolates individual lived experience — not a bounded memory horizon per se — as the source of the generational optimum.

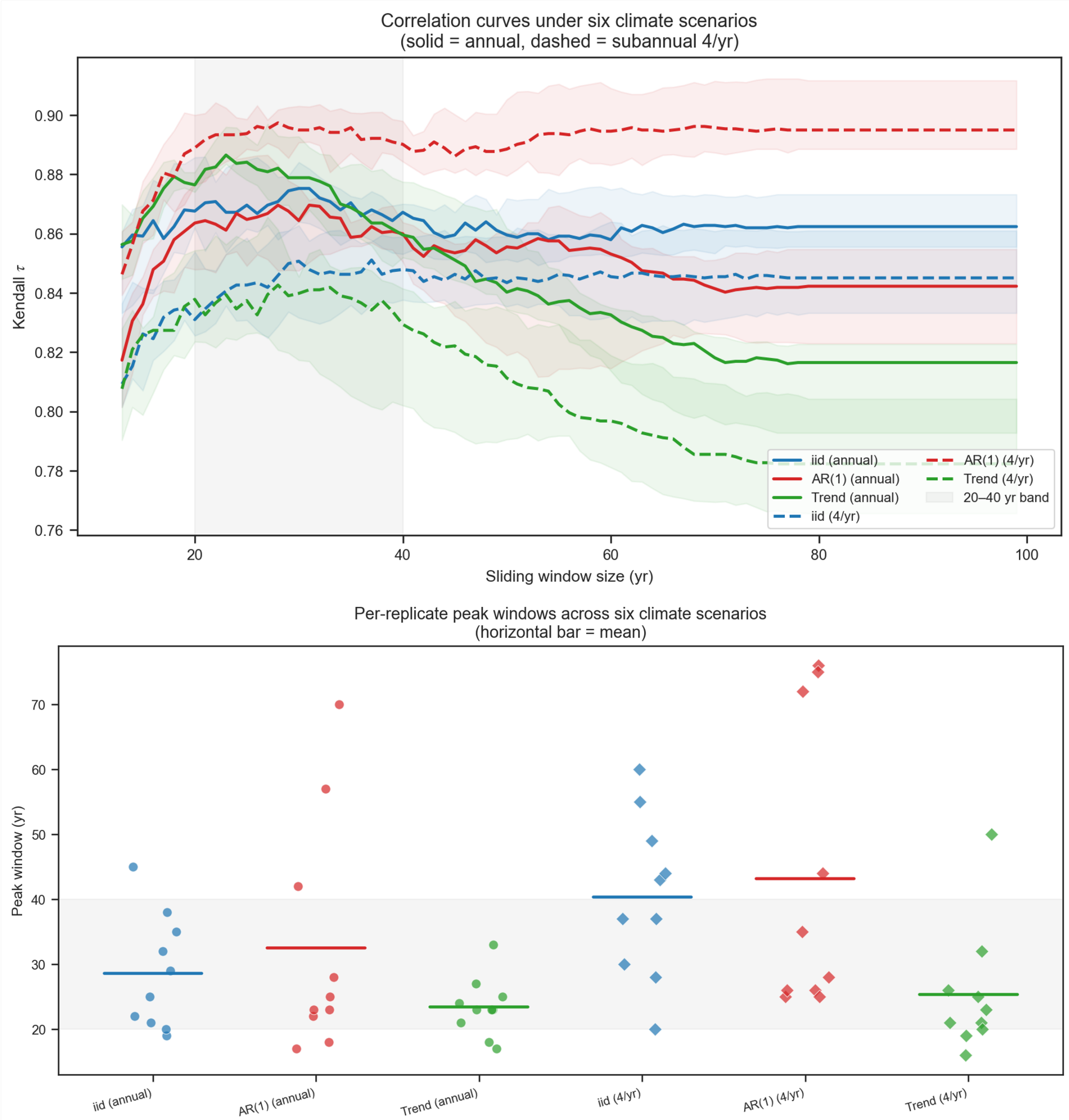

Figure S9 – Climate-forcing robustness matrix for the H1 (personal baseline) scenario. Top: window–τ correlation curves for six forcing scenarios — three stochastic processes (i.i.d., AR(1), trend-plus-noise) at two temporal resolutions (solid = annual; dashed = sub-annual, four steps per year aggregated to annual units); the shaded band marks the 20–40-year generational range. Bottom: per-replicate peak (optimal) windows for the same six scenarios (points, individual runs; horizontal bars, means; n = 10 runs per scenario). Across every forcing the peak windows cluster within the generational band, confirming that the emergent generational optimum does not depend on the choice of climate process or temporal resolution.

separates model logic from parameter specification, allowing independent researchers to explore alternative settings through configuration modification without requiring code changes.

3.4.3 Model Extensions

The current implicit design could be extended to incorporate spatial heterogeneity in climate exposure and observer networks, enabling the investigation of regional collective memory patterns and the spatial diffusion of climate information. Direct agent-to-agent learning mechanisms could supplement the current collective archive approach, exploring how social networks and communication patterns influence collective memory formation. The main analysis already drives the model with an AR(1) autocorrelated forcing, with independent-noise and linear-trend variants tested for robustness; further extensions could introduce cyclical or regime-shifting climate forcing.

3.4.4 Computational Requirements

Typical simulations complete rapidly on standard computational hardware, with 300-year runs under default parameters finishing in under five minutes. Memory requirements remain minimal due to bounded agent memory structures and efficient data management. The implementation supports parallel execution across multiple CPU cores for parameter-sweep experiments, enabling comprehensive exploration of the parameter space for sensitivity analysis and calibration studies. This ODD+D protocol provides a comprehensive specification enabling independent implementation and replication of the agent-based model, supporting transparent evaluation of the proposed mechanisms linking individual psychology to collective climate memory dynamics.

4 Supplementary Note 4: Supplementary results for the mismatch analysis

The mismatch analysis in the main text (Figure 3) establishes that, where the historical-archive WDI (H-WDI) and the natural-proxy WDI (N-WDI) disagree, the disagreement carries a systematically shifted-comparison signature rather than being random noise. A natural follow-up question is whether these mismatches are also organised in time, for example, clustering toward the end of sustained wet or dry epochs, as a shifting baseline would predict if a generation's reference point drifts with prolonged conditions. To examine this, we plotted the per-year mismatch as the categorical offset between the two series (Δ = H-WDI level $-$ N-WDI level; positive values mean the archive judged the year wetter than the proxy, negative values drier) and overlaid the wet and dry epochs, defined exactly as in the main text (the sign of the 30-year low-pass N-WDI: wet where the smoothed trend is positive, dry where negative) (Figure S10).

Two observations emerge. First, the annual offset is weakly anti-correlated with the smoothed wet/dry state (Pearson's $r = -0.23$ between the annual offset and the smoothed state, n = 208 years; nominal two-sided $p = 0.001$, not corrected for serial autocorrelation). During sustained wet epochs the archive tends to read slightly drier than the proxy, and during sustained dry epochs slightly wetter, which is the direction expected if the perceived baseline drifts toward recent prolonged conditions. Second, individual large offsets ($|\Delta| = 2$) occur in both epoch types and are not confined to epoch boundaries.

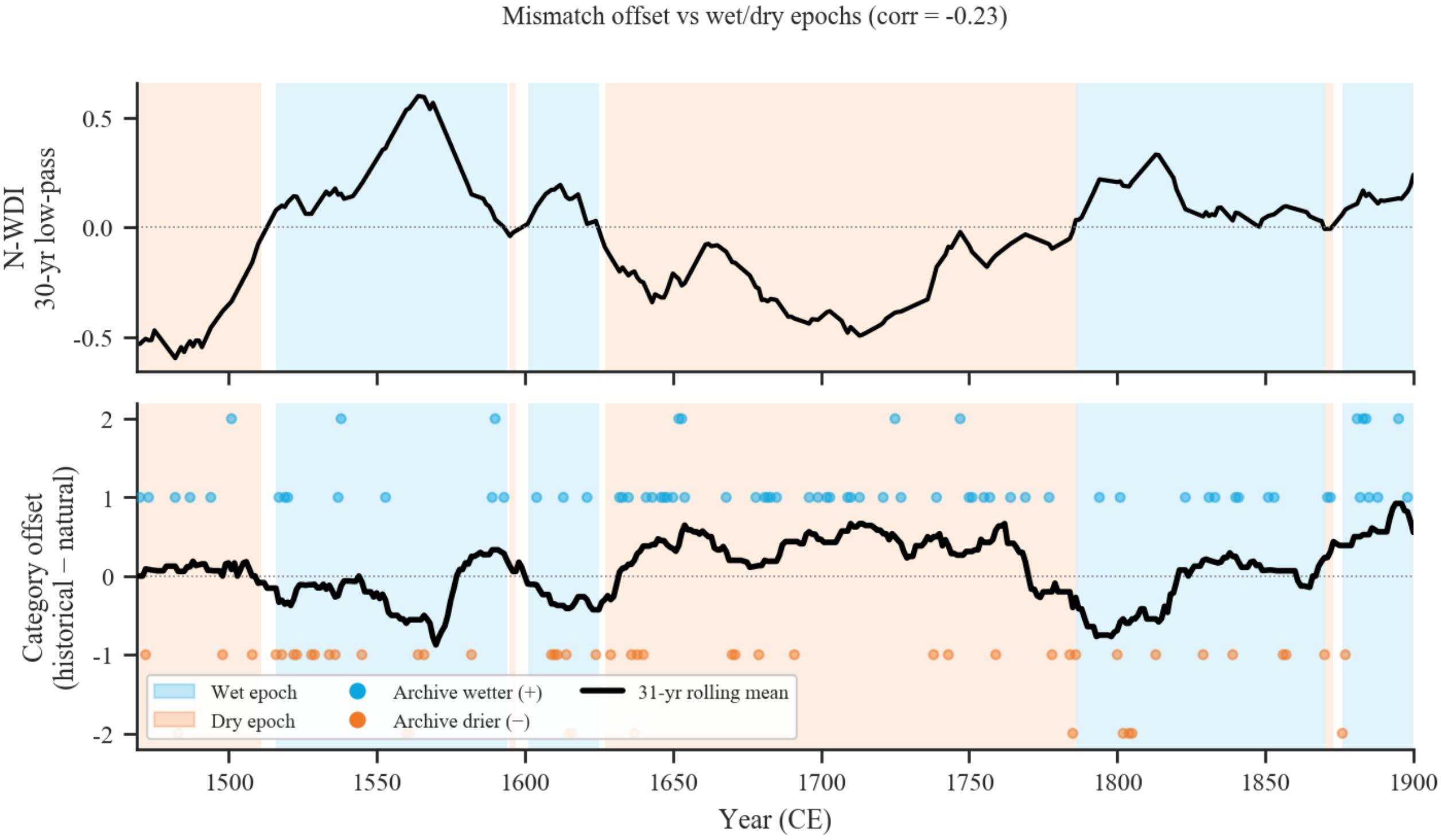

Figure S10 – Temporal structure of H-WDI/N-WDI mismatches over 1470–1900 CE. Top: the 30-year low-pass N-WDI, used to define sustained wet epochs (blue shading, smoothed trend positive) and dry epochs (orange shading, smoothed trend negative). Bottom: the per-year categorical offset between the archival and natural series (Δ = H-WDI level − N-WDI level); points mark mismatch years where the archive read wetter (blue, $\Delta > 0$) or drier (orange, $\Delta < 0$) than the proxy, and the black line is the 31-year rolling mean of the offset. The annual offset is weakly negatively associated with the smoothed wet/dry state (Pearson's $r = -0.23$, n = 208 years; nominal two-sided $p = 0.001$, not corrected for the serial autocorrelation of the low-pass series and therefore descriptive), i.e. the archive tends to read slightly drier than the proxy during wet epochs and slightly wetter during dry epochs, consistent with a baseline that drifts toward recent prolonged conditions.

As a null control, we performed the same mismatch analysis between the reconstructed N-WDI (from tree-ring data) and the instrument-measured N-WDI (Figure S11). The results show that their agreement is higher than that between N-WDI and H-WDI over the validation period (1901–2000 CE; Kendall's $\tau = 0.214$, two-sided $p = 0.012$; weighted $\kappa = 0.24$; n = 100 years; see main text Figure 3). Some cells reached $p < 0.05$ (two-sided Monte Carlo randomisation test, 10,000 iterations, not adjusted for multiple comparisons), but only in combinations with one to three mismatched pairs, and mostly with an offset of the opposite sign to the pattern in the historical archives: MD to MW at shift $= 1$ (n = 1, $p < 0.001$), shift $= 2$ (n = 1, $p = 0.001$) and shift $= 3$ (n = 1, $p < 0.001$), and MW to MD at shift $= 3$ (n = 3, $p = 0.045$). The directionally consistent pattern across several well-populated cells found for the H-WDI, therefore, does not appear in this comparison, which is consistent with the contrast-with-past-events mechanism being specific to perception-mediated records.

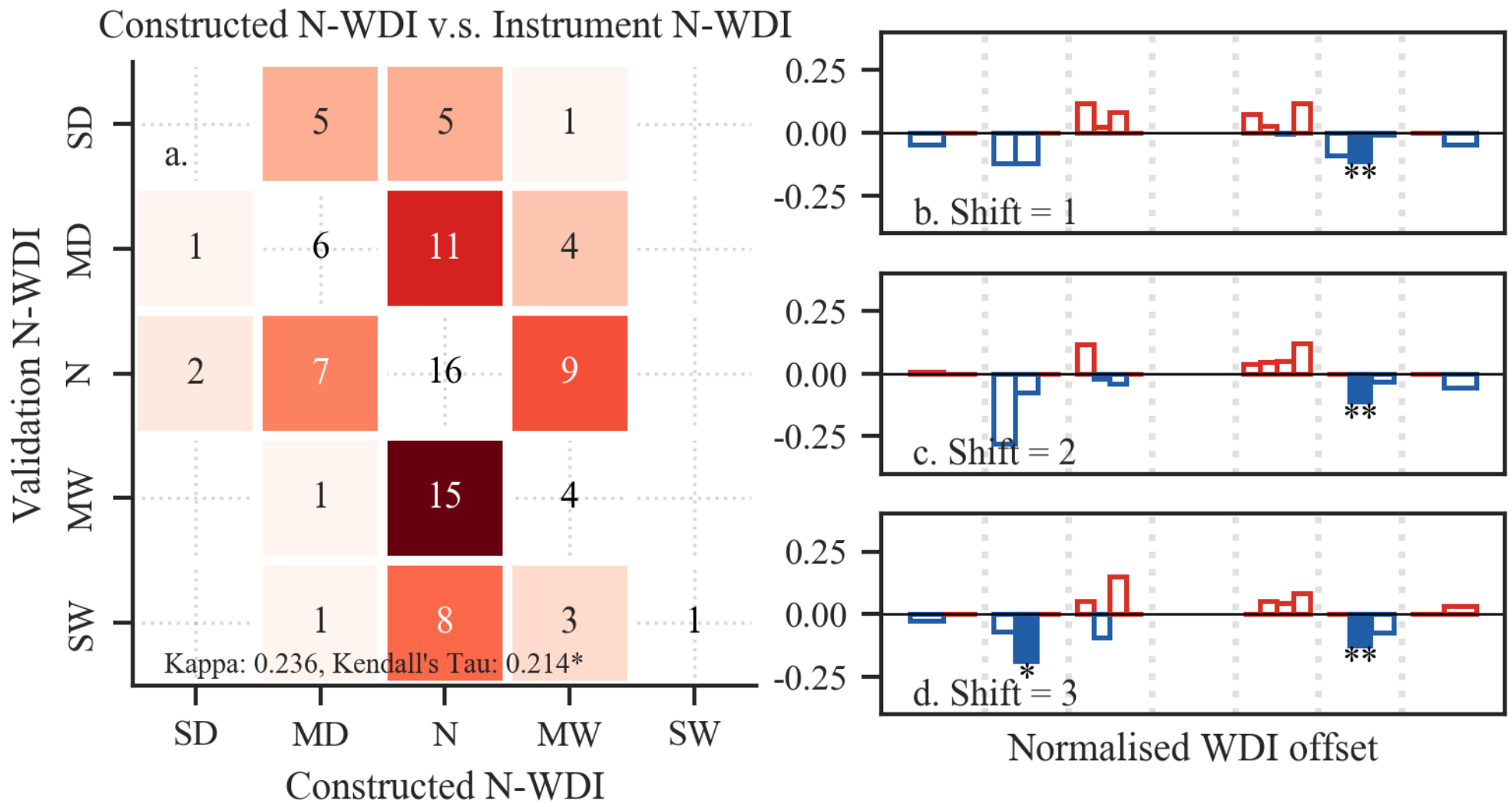

Figure S11 – Mismatch pattern of WDI levels. a. Confusion matrix, showing the number of matched (diagonal, white colour) and mismatched (off-diagonal, red colour) data of climatic extreme levels between the reconstructed N-WDI and the instrumental N-WDI over the validation period (1901–2000 CE, n = 100 years; the Kendall's τ annotation is marked by a two-sided test, * $p < 0.05$). b.-d. Normalised WDI offset between the reconstructed and instrumental N-WDI under different shifted cases. Significance is assessed by a two-sided Monte Carlo randomisation test (10,000 iterations): instrumental WDI levels are independently redrawn at fixed frequencies, synthetic natural values are drawn from $\mathcal{N}(0, 1)$, and the same look-back procedure (shift $= 1, 2, 3$) is applied; p values are not adjusted for multiple comparisons, and filled bars mark $p < 0.05$ (* denotes $p < 0.05$ and ** denotes $p < 0.01$; exact p values and numbers of mismatched pairs are given in the text above; see Methods).

578 5 Supplementary References

579 1. Yang, Y. *Millennium regional Extreme Drought and flood Atlas of China*. (China Cartographic Society, 2024).

580 2. Chen, Y., Syvitski, J. P. M., Gao, S., Overeem, I. & Kettner, A. J. Socio-economic Impacts on Flooding: A 4000-Year History of the Yellow River, China. *Ambio* **41**, 682–698 (2012).

581 3. Ge, Q. & Zhang, P. The evaluation on climatic information in the historical literatures. *Acta Geographic Sinica* **45**, 23–30 (1990).

582 4. Peng, S., Ding, Y., Liu, W. & Li, Z. 1 km monthly temperature and precipitation dataset for China from 1901 to 2017. *Earth System Science Data* **11**, 1931–1946 (2019).

583 5. Schneider, U., Hänsel, S., Finger, P., Rustemeier, E. & Ziese, M. GPCC Full Data Monthly Version 2022 at 0.5°: Monthly Land-Surface Precipitation from Rain-Gauges built on GTS-based and Historic Data: Globally Gridded Monthly Totals. min. 20 MB–max. 300 MB per gzip archive (10 years per archive) (2022) doi:10.5676/DWD_GPCC/FD_M_V2022_050.

584 6. Harris, I., Osborn, T. J., Jones, P. & Lister, D. Version 4 of the CRU TS monthly high-resolution gridded multivariate climate dataset. *Scientific Data* **7**, 109 (2020).

585 7. Li, Q. *et al.* The 225-year precipitation variability inferred from tree-ring records in Shanxi Province, the North China, and its teleconnection with Indian summer monsoon. *Global and Planetary Change* **132**, 11–19 (2015).

586 8. Xu, C. *et al.* Asian-Australian summer monsoons linkage to ENSO strengthened by global warming. *npj Climate and Atmospheric Science* **6**, 8 (2023).

587 9. Li, J. *et al.* Deciphering Human Contributions to Yellow River Flow Reductions and Downstream Drying Using Centuries-Long Tree Ring Records. *Geophysical Research Letters* **46**, 898–905 (2019).

588 10. Liu, Y. *et al.* Recent anthropogenic curtailing of Yellow River runoff and sediment load is unprecedented over the past 500 y. *Proceedings of the National Academy of Sciences* **117**, 18251–18257 (2020).

589 11. Yang, B., Wang, J. & Liu, J. A 1556 year-long early summer moisture reconstruction for the Hexi Corridor, Northwestern China. *Science China Earth Sciences* **62**, 953–963 (2019).

590 12. Yi, L. *et al.* Exploratory Precipitation in North-Central China during the Past Four Centuries. *Acta Geologica Sinica - English Edition* **84**, 223–229 (2010).

591 13. Yang, F. *et al.* The spatial distribution of precipitation over the West Qinling region, China, AD 1470–2000. *Palaeogeography, Palaeoclimatology, Palaeoecology* **443**, 278–285 (2016).

592 14. Yi, L. *et al.* Reconstructions of annual summer precipitation and temperature in north-central China since 1470 AD based on drought/flood index and tree-ring records. *Climatic Change* **110**, 469–498 (2012).

593 15. Tan, L. *et al.* Climate patterns in north central China during the last 1800 yr and their possible driving force. *Climate of the Past* **7**, 685–692 (2011).

594 16. Juárez, M. A. & Steel, M. F. J. Model-Based Clustering of Non-Gaussian Panel Data Based on Skew-t Distributions. *Journal of Business & Economic Statistics* **28**, 52–66 (2010).

595 17. Ge, J. *History of Chinese Population*. (Fudan University Press, Shanghai, 2005).

596 18. Baumeister, R. F., Bratslavsky, E., Finkenauer, C. & Vohs, K. D. Bad is Stronger than Good. *Review of General Psychology* **5**, 323–370 (2001).

597 19. Rozin, P. & Royzman, E. B. Negativity Bias, Negativity Dominance, and Contagion. *Personality and Social Psychology Review* **5**, 296–320 (2001).

598 20. Tversky, A. & Kahneman, D. Judgment under Uncertainty: Heuristics and Biases. *Science* **185**, 1124–1131 (1974).

599 21. de Guttry, C. & Ratter, B. Expiry date of a disaster: Memory anchoring and the storm surge 1962 in Hamburg, Germany. *International Journal of Disaster Risk Reduction* **70**, 102719 (2022).

600 22. Song, S., Wang, S., Jiao, C. & Ibarra, E. J. M. ABSESpy: An agent-based modeling framework for social-ecological systems. *Journal of Open Source Software* **9**, 6298 (2024).
