# Supplementary material for: Archival and palaeoenvironmental documentation of extreme climate events reveals generational amnesia in collective memory
Source: Nat Commun. 2026 Nov 9;17:11327. doi: 10.1038/s41467-026-78922-x (PMC13748574; doi:10.1038/s41467-026-78922-x)
Supplement: Supplementary file 2 [file 41467_2026_78922_MOESM2_ESM.pdf]

nature portfolio

Corresponding author(s): Shuang Song

Last updated by author(s): Sep 19, 2026

Reporting Summary

Nature Portfolio wishes to improve the reproducibility of the work that we publish. This form provides structure for consistency and transparency in reporting. For further information on Nature Portfolio policies, see our Editorial Policies and the Editorial Policy Checklist.

Statistics

For all statistical analyses, confirm that the following items are present in the figure legend, table legend, main text, or Methods section.

n/a	Confirmed	
☐	☒	The exact sample size (*n*) for each experimental group/condition, given as a discrete number and unit of measurement
☐	☒	A statement on whether measurements were taken from distinct samples or whether the same sample was measured repeatedly
☐	☒	The statistical test(s) used AND whether they are one- or two-sided<br>*Only common tests should be described solely by name; describe more complex techniques in the Methods section.*
☒	☐	A description of all covariates tested
☐	☒	A description of any assumptions or corrections, such as tests of normality and adjustment for multiple comparisons
☐	☒	A full description of the statistical parameters including central tendency (e.g. means) or other basic estimates (e.g. regression coefficient) AND variation (e.g. standard deviation) or associated estimates of uncertainty (e.g. confidence intervals)
☐	☒	For null hypothesis testing, the test statistic (e.g. *F*, *t*, *r*) with confidence intervals, effect sizes, degrees of freedom and *P* value noted<br>*Give P values as exact values whenever suitable.*
☐	☒	For Bayesian analysis, information on the choice of priors and Markov chain Monte Carlo settings
☐	☒	For hierarchical and complex designs, identification of the appropriate level for tests and full reporting of outcomes
☐	☒	Estimates of effect sizes (e.g. Cohen's *d*, Pearson's *r*), indicating how they were calculated

Our web collection on statistics for biologists contains articles on many of the points above.

Software and code

Policy information about availability of computer code

Data collection	No dedicated software was used for primary data collection. The study used previously published historical, palaeoclimate and instrumental datasets obtained from published sources and public data repositories. Dataset sources and access information are reported in the Data section below.
Data analysis	All statistical analyses, data processing, Bayesian data integration and agent-based simulations were conducted in Python 3.11. Analyses used NumPy 2.4.4, pandas 3.0.2, SciPy 1.17.1, scikit-learn 1.8.0, xarray 2026.4.0, PyMC 5.28.4, ArviZ 0.20.0 and SALib 1.5.2. The agent-based model was implemented using ABSESpy 0.11.5. Spatial data processing and figure preparation used GeoPandas 0.14.4, matplotlib 3.10.8 and QGIS 4.2.0. Statistical and computational procedures included Pearson correlation, Kendall's τ, quadratic-weighted Cohen's κ, Shapiro–Wilk tests, permutation tests, Monte Carlo randomisation tests, percentile bootstrap confidence intervals, sliding-window re-standardisation, Bayesian latent-variable modelling using NUTS MCMC, and Sobol global sensitivity analysis using Saltelli sampling. The analysis pipeline and agent-based model are available at https://github.com/SongshGeo/shifting_baseline.

For manuscripts utilizing custom algorithms or software that are central to the research but not yet described in published literature, software must be made available to editors and reviewers. We strongly encourage code deposition in a community repository (e.g. GitHub). See the Nature Portfolio guidelines for submitting code & software for further information.

Data

Policy information about availability of data

All manuscripts must include a data availability statement. This statement should provide the following information, where applicable:

- Accession codes, unique identifiers, or web links for publicly available datasets
- A description of any restrictions on data availability
- For clinical datasets or third party data, please ensure that the statement adheres to our policy

This study used existing historical, palaeoclimate and instrumental datasets. Historical drought and flood records were derived from the Atlas of Extreme Droughts and Floods over the Past Millennium in China (China Cartographic Publishing House and Zhonghua Map Society, 2024; ISBN 978-7-5588-0466-3). Tree-ring-based hydroclimate reconstructions used to construct the natural-proxy Wet/Dry Index (N-WDI) were obtained from the NOAA/NCEI World Data Service for Paleoclimatology; individual source datasets are listed in Supplementary Table S8. Instrumental validation datasets comprised the 1-km monthly temperature and precipitation dataset for China (https://doi.org/10.5281/zenodo.3114194), GPCC Full Data Monthly Version 2022 at 0.5° resolution (https://doi.org/10.5676/DWD_GPCC/FD_M_V2022_050), and CRU TS4.08 from the Centre for Environmental Data Analysis, for which free registration is required. Historical administrative geography was obtained from the China Historical Geographic Information System (CHGIS), Version 6, licensed for non-commercial academic use.

Datasets generated in this study include the annual N-WDI for 1470–2000 CE, with posterior mean, posterior standard deviation and 94% highest-density interval, and the regional historical-archive WDI (H-WDI) for 1470–1900 CE, including the number of contributing archive sites and five-level WDI classifications. These generated datasets are provided as Supplementary Data 1. The analysis code is available at https://github.com/SongshGeo/shifting_baseline.

Research involving human participants, their data, or biological material

Policy information about studies with human participants or human data. See also policy information about sex, gender (identity/presentation), and sexual orientation and race, ethnicity and racism.

Reporting on sex and gender	We did not conduct any sex or gender based analyses.
Reporting on race, ethnicity, or other socially relevant groupings	We did not conduct any analyses related to race, ethnicity or any other socially relevant grouping.
Population characteristics	See above
Recruitment	This study did not involve human participants.
Ethics oversight	This study did not involve human participants.

Note that full information on the approval of the study protocol must also be provided in the manuscript.

Field-specific reporting

Please select the one below that is the best fit for your research. If you are not sure, read the appropriate sections before making your selection.

☐ Life sciences ☐ Behavioural & social sciences ☒ Ecological, evolutionary & environmental sciences

For a reference copy of the document with all sections, see nature.com/documents/nr-reporting-summary-flat.pdf

Ecological, evolutionary & environmental sciences study design

All studies must disclose on these points even when the disclosure is negative.

Study description	This study uses a retrospective quantitative comparative design combining historical archives, tree-ring-based hydroclimate reconstructions, instrumental validation datasets and agent-based modelling. We compared annual Wet/Dry Index (WDI) severity levels derived from historical climatic archives with WDI estimates reconstructed from natural proxies for northern China during the pre-instrumental period. We then tested mismatch patterns, correlations, sliding-window restandardisation, and an agent-based model based on Shifting Baseline Syndrome to evaluate whether perceptual bias contributed to historical records of climatic extremes.
Research sample	The research sample consisted of historical drought/flood archive records and natural-proxy hydroclimate reconstructions for northern China, especially the Yellow River Basin. Historical records were derived from multi-source documentary materials, including official histories, local gazetteers, Veritable Records, personal diaries and inscriptions, and were classified into five WDI levels: Severe Drought, Moderate Drought, Normal, Moderate Wet and Severe Wet. Natural-proxy data consisted primarily of publicly available tree-ring-based reconstructions of summer precipitation and Yellow River runoff.
Sampling strategy	No prospective sampling or sample-size calculation was conducted because the study used existing archival and palaeoclimate datasets.
Data collection	Historical WDI data were derived from an existing published compilation of documentary drought and flood records, which integrates official histories, local gazetteers, Veritable Records, personal diaries and inscriptions. Records were screened and processed using predefined hydrometeorological criteria and classified into five WDI severity levels. Natural-proxy data were obtained from publicly available palaeoclimate reconstructions and integrated using a Bayesian latent-variable model. Instrument-based precipitation

datasets for 1901–2000 CE were used to validate the integrated natural-proxy WDI series.

Timing and spatial scale	The main pre-instrumental analysis covered 1470–1900 CE, with annual temporal resolution. Instrument-based validation used data from 1901–2000 CE. The spatial scale was regional, covering northern China and the Yellow River Basin.
Data exclusions	Several records were excluded or not used for WDI classification when they did not directly represent hydrometeorological extremes. The early period before 1470 CE was excluded from the main analysis because of low archive retention and stronger source bias. Proxy datasets with insufficient independence, weak calibration, inadequate temporal resolution or obvious dependence on previously used materials were not used in the main natural-proxy scenario.
Reproducibility	Reproducibility was addressed through predefined archival classification criteria, explicit WDI thresholds, uncertainty estimation through repeated simulations, validation of natural-proxy reconstructions against instrumental datasets, robustness tests using alternative proxy-data combinations, and sensitivity analyses of the agent-based model. The natural-proxy integration used documented Bayesian modelling procedures, including MCMC sampling and convergence checks. Complete model code and configuration files are available on GitHub.
Randomization	Random allocation was not relevant because this study did not involve experimental groups, treatments, organisms or participants.
Blinding	Blinding was not applicable because the study was based on historical, proxy and model datasets rather than experimental treatment groups.

Did the study involve field work? ☐ Yes ☒ No

Reporting for specific materials, systems and methods

We require information from authors about some types of materials, experimental systems and methods used in many studies. Here, indicate whether each material, system or method listed is relevant to your study. If you are not sure if a list item applies to your research, read the appropriate section before selecting a response.

Materials & experimental systems

n/a	Involved in the study	
☒	☐	Antibodies
☒	☐	Eukaryotic cell lines
☒	☐	Palaeontology and archaeology
☒	☐	Animals and other organisms
☒	☐	Clinical data
☒	☐	Dual use research of concern
☒	☐	Plants

Methods

n/a	Involved in the study	
☒	☐	ChIP-seq
☒	☐	Flow cytometry
☒	☐	MRI-based neuroimaging

Plants

Seed stocks	*Report on the source of all seed stocks or other plant material used. If applicable, state the seed stock centre and catalogue number. If plant specimens were collected from the field, describe the collection location, date and sampling procedures.*
Novel plant genotypes	*Describe the methods by which all novel plant genotypes were produced. This includes those generated by transgenic approaches, gene editing, chemical/radiation-based mutagenesis and hybridization. For transgenic lines, describe the transformation method, the number of independent lines analyzed and the generation upon which experiments were performed. For gene-edited lines, describe the editor used, the endogenous sequence targeted for editing, the targeting guide RNA sequence (if applicable) and how the editor was applied.*
Authentication	*Describe any authentication procedures for each seed stock used or novel genotype generated. Describe any experiments used to assess the effect of a mutation and, where applicable, how potential secondary effects (e.g. second site T-DNA insertions, mosiacism, off-target gene editing) were examined.*
