# Supplementary material for: Archival and palaeoenvironmental documentation of extreme climate events reveals generational amnesia in collective memory
Source: Nat Commun. 2026 Nov 9;17:11327. doi: 10.1038/s41467-026-78922-x (PMC13748574; doi:10.1038/s41467-026-78922-x)
Supplement: Supplementary file 3 [file 41467_2026_78922_MOESM4_ESM.pdf]

Supplementary Data 1: Annual Wet/Dry Index series generated in this study, cited in the Data Availability statement. Two sheets:

- **README** — definition of every column.
- **WDI series** — one row per calendar year: `year`; `nwdi_mean`, `nwdi_sd`, `nwdi_hdi_3`, `nwdi_hdi_97` and `nwdi_class` for the natural-proxy WDI (posterior mean, standard deviation, 94% highest-density interval and five-level class, 1470–2000 CE); `hwdi`, `hwdi_class` and `hwdi_n_sites` for the regional historical-archive WDI (value, five-level class and number of contributing sites, 1470–1900 CE).
