# Supplementary material for: Archival and palaeoenvironmental documentation of extreme climate events reveals generational amnesia in collective memory
Source: Nat Commun. 2026 Nov 9;17:11327. doi: 10.1038/s41467-026-78922-x (PMC13748574; doi:10.1038/s41467-026-78922-x)
Supplement: Supplementary file 4 [file 41467_2026_78922_MOESM5_ESM.pdf]

nature portfolio

Peer Review File

Archival and palaeoenvironmental documentation of historical extreme events reveals generational amnesia in collective memory

Corresponding Author: Dr Shuang Song

This file contains all reviewer reports in order by version, followed by all author rebuttals in order by version.

Version 0:

Reviewer comments:

Reviewer #1

(Remarks to the Author)
This is a fascinating and excellent study that brings scientific data, historical data and cultural data in the form of collective memory together in a way that demonstrates how these seemingly very different areas of knowledge can be aligned to make meaningful discoveries. While the model created by the authors offers a valuable way to assess, understand, provide for and even forecast and mitigate responses to extreme climate events, the alignment of this disparate data through the model is also an important achievement. The authors have been diligent not only in their use of this literature but in their broad selection of historical data. The discussion of collective memory is appropriate and is supported by an extensive literature and this paper extends and adds to that literature, providing an unusual and creative perspective and analyzing collective memory in a unique way. By aligning historical data/collective memory with data from the natural world, the authors have created a model that enables the measurement of collective memory itself. In particular, the introduction of the Shifting Base Syndrome casts an innovative light on both collective memory and perceptual bias.
The research supports the authors claims about perceptual bias as well as the possibility of utilizing their model in assessing extreme climate events. I see no flaws in their methodology and interpretations.

My field is Archival Studies where both historical data and collective memory are paramount concerns. This study offers exciting new perspectives and possible applications for greater understandings in both of these areas.
This research is extremely detailed and the authors have carefully described their formulas and model and so it can be replicated.

(Remarks on code availability)
While I have accessed the code, this is not my area of expertise and so I have not installed it.

Reviewer #2

(Remarks to the Author)
This manuscript combines a four-century archival Wet/Dry Index (WDI) for northern China with tree-ring-based hydroclimate reconstructions and an agent-based model (ABM) to argue that Shifting Baseline Syndrome (SBS) imprints generational-scale biases on collective memory of climate extremes. Empirically, the authors show systematic mismatches between a “subjective” archive-based WDI and an “objective” proxy-based WDI, and that re-standardising the proxy series within 20–40-year sliding windows increases rank correlation. An ABM with agents that remember only a finite past is then claimed to reproduce an optimal window of ~30 years. My overall assessment is that the topic and basic idea are interesting and potentially significant, but the current manuscript is far from the level of scientific accuracy and clarity required for publication.

Major comments

A central strength of the design is the use of an instrumental validation period (1901–2000 CE) in which the validation comparison is strictly between the integrated natural-proxy WDI and independent gridded precipitation datasets. In that

period the subjective archival WDI is not used to construct either series, so collective memory cannot contaminate this particular validation step. Historical archives certainly still exist after 1900, but they are not part of the proxy-based WDI or the instrumental datasets, and in that limited sense they are indeed excluded from the comparison.

However, the main research questions as formulated in the Introduction are not actually answered by the methods applied. The authors state that they "test the degree to which period-specific perception bias influences the formation of historical narratives of extreme climatic events" and that perceptual bias leads to a "generational offset in collective memory". What they measure empirically are (i) discrepancies between categorical WDI assignments in two already-constructed series, (ii) changes in Kendall's τ under sliding-window re-standardisation of the objective WDI, and (iii) the behaviour of a stylised ABM driven by i.i.d. normal climate noise. These are not direct measurements of the "formation of historical narratives" or of a "degree" of bias in narrative construction. The archival WDI is itself a heavily processed regional index, which has already collapsed many qualitative narratives into a single ordinal time series. Without an explicit, operational definition of "formation of narratives" and a corresponding quantitative metric, the strong claims about how narrative formation is influenced by period-specific perception bias are not supported by the current analyses. This could be solved by rewriting the research question.

Relatedly, the notion of "degree to which period-specific perception bias influences the formation of historical narratives" lacks a clear sense of direction or observable quantity. It is not specified whether "more formation" should mean more narratives per event, more detailed descriptions, higher probability of recording, or something else. Nor is there any attempt to count or otherwise quantify the number of distinct historical narratives as a function of climate anomalies. In practice, the paper infers perceptual bias solely from the relative alignment of an archive-based index and a proxy-based index. This is a much narrower question than the one formulated in the Introduction, and the framing should be revised accordingly or the analyses significantly expanded.

The "Monte Carlo approach" used in the mismatch analysis needs clearer definition and more precise presentation. In Methods 4.2.2, the authors describe generating synthetic climate series (random normal) and randomly assigning historical classifications according to observed category frequencies, repeating this 100 times to build a null distribution for the mismatch metric. Methodologically, this is a Monte Carlo randomisation under a specified null model. However, in the main text and Figure 3 caption it is referred to only as a "Monte Carlo approach", without stating what is randomised, what the null hypothesis is, or how many simulations are used. As it stands, this is too imprecise to count as a clearly described Monte Carlo analysis and will be opaque to many readers. The authors should explain in the main text that they perform a Monte Carlo randomisation test, specify the null model (independent random classifications with fixed marginal frequencies), and justify the number of iterations or increase it if necessary.

The ABM description in the main text is highly insufficient, even for readers familiar with ABMs. Section 2.4 and Figure 5 give only a schematic overview ("a batch of observers who perceive climatic extremes and may record their perceptions of severity levels", with two baseline options), while all substantive detail is pushed to SI S3. For a general-audience journal, the main text should summarize at least the nature of the climate process, the agents' memory structure, the decision rule for recording, the demographic process, and how the model outputs are converted into a WDI-like series. As written, it is impossible to understand from the main text alone how Figure 5 was produced or in what sense the model "reproduces" the empirical pattern. The description should also make explicit that anchoring and negativity bias, which are emphasised in the SI as theoretical foundations, are actually implemented in the recording propensity and baseline construction; at present, these cognitive mechanisms are not mentioned in the modelling section of the main text, and anchoring appears only later in the Discussion as a general cognitive process, not as something concretely encoded in the ABM.

In the ABM code, the z-score formula is wrong due to operation precedence: climate - baseline/std should be (climate - baseline)/std. This must completely change the results.

The temporal and climatic structure of the ABM needs stronger justification and exploration. Time advances in annual ticks and the climate series is generated as N(0,1) at each step. The annual timestep is chosen to match the yearly WDI, which is reasonable, but it ignores trends and persistence that are likely present in actual climate. The authors then interpret the ~30-year optimal sliding window emerging from this model as supporting a generational SBS mechanism. Given the simplistic climate process, the untested sensitivity of the model output to various assumption, this claim is fragile. The model's results may be highly sensitive to the arbitrary timestep (e.g. if the model ran at seasonal resolution, the effective memory window in years could change), there is a peculiar age structure, no population growth, and no trend or autocorrelation in weather. The statement that "collective climate records aligned with the continuous climate" is also misleading: in the model, climate is not a continuous physical process but independent Gaussian noise. At minimum, the authors should justify the annual step in terms of the empirical WDI and discuss whether and how alternative temporal resolutions or realistic climate processes (with trend and persistence) would affect the emergent memory window.

Demographic assumptions in the ABM also warrant more careful treatment. The model introduces a fixed number of new agents each year and removes agents at maximum age, with no dependence of births or deaths on current population size. This produces a flat age distribution between the minimum and maximum ages. Because each agent's memory window and thus their baseline length depends on age, the resulting collective memory is strongly shaped by this artificial age structure, which does not resemble actual historical demography. The authors link their empirical 20–40-year sliding window to "the generational life expectancy of humans in ancient China", yet the default model uses a maximum age of 40 years (in the SI) or 60 years (in the main Methods) and a recruitment process that is not tied to any empirical demographic data. They report an ANOVA-based sensitivity analysis on a limited set of parameters, for which they don't report effect sizes, but this is not a

global sensitivity analysis in the usual sense: there is no Sobol or Morris-type decomposition to quantify how much of the variance in the key output (the location and magnitude of the correlation peak) is attributable to each parameter and to interactions. Without such an analysis, it is hard to know whether the emergence of a ~30-year optimum is robust or depends delicately on a particular combination of lifespan, recruitment, loss rate, and climate variance, beyond all the remaining structural assumptions. I would strongly recommend either performing a basic global sensitivity analysis or, at minimum, substantially expanding the current sensitivity study and clearly describing how parameters are sampled and how their influence on the outputs is quantified.

More generally, the connection between the ABM and the data feels ad hoc. From the SI it is clear that the model is conceptually grounded in cognitive psychology—negativity bias and anchoring, and in SBS, but in the main text the modelling section is framed almost exclusively in terms of SBS, without stating which psychological mechanisms are actually encoded. There is no attempt to calibrate the model to the empirical WDI series, nor any formal model-data comparison beyond the qualitative observation that both exhibit an optimal window of ~30 years, which is arguable given the log scale. Alternative mechanisms that could also generate a similar sliding-window optimum (e.g. purely statistical effects of autocorrelation or of the rolling standardisation itself or the average age of agents) are not explored. As a result, the current ABM functions more as an non-convincing toy model than as a genuine explanatory or testing framework; the link to the empirical pattern needs to be made more rigorous.

The figures require substantial revision for clarity and scientific precision. Figure 1 combines three conceptually distinct elements (a study-area map, a conceptual SBS diagram, and a data-processing schematic—into a single figure). For readability it would be preferable to separate these or at least more clearly delineate them. In Figure 2a, the validation period (1901–2000 CE) appears to have larger fluctuations in the integrated proxy WDI than earlier periods; this noticeable change is not explained. The caption's phrase "the grey area indicates the uncertainties" is vague; the authors should specify whether this represents, for example, the 5–95% posterior credible interval or whatever else. Panel 2c includes a colour label that is not clearly identified in the caption (I assume it's the title of the plot). Panel 2d asserts that both WDI series "follow a normal distribution", but no normality test is reported; unless a formal test is performed and described, this should be phrased differently. Figure 3's legend seems unnecessary, panels c–f are not clearly labelled in the figure, the "validation period" shows up in panel f but I don't think it even belongs ther, and again the "Monte Carlo approach" is mentioned without any explanation. In Figure 4, plotting legends on top of error bars makes the legend hard to see. Figure 5's correlation-versus-window-size curves for the ABM are not interpretable without reading SI S3; the main text should explain how many runs were averaged, how events were discretized into levels, and how the curves relate to the observational analysis. At present, one effectively needs the SI to make sense of any of the modelling results, which is too heavy a dependence.

Finally, there are a few conceptual and numerical inconsistencies that should be resolved. In Section 2.3 the improvement in correlation after re-standardisation is described as "ranging from 0.1 to 0.2", while a few lines later the average optimal window size is said to yield a "maximum correlation increase of 0.12". From SI Table S6 it appears that 0.1–0.2 refers to the range across different proxy-integration and validation scenarios, and 0.12 is the maximum increase for the main pure-natural scenario averaged across temporal segments. This should be stated explicitly to avoid the impression of contradiction.

Minor comments

For clarity throughout the manuscript, it would be helpful to introduce explicit labels for the two WDI series, such as O-WDI for the objective proxy-based index and S-WDI for the subjective archival index, and use these consistently in text and figures. This would make it much easier for readers to track which series is being transformed, validated, or used as a baseline.

In Table S2, the coordinates are given as raw numbers; the authors should add N/S/E/W designations (e.g. 35.18°N, 103.73°E) to conform to standard geographic notation. In SI 1.4, the statement that self-comparison materials "may account for more than 10%" is described as a "subjective estimation"; the basis for this figure is unclear. If it is from an actual tally on a defined corpus, the method should be described; if not, a precise percentage should be avoided and replaced by a qualitative statement. In Table S3, the 1690 CE translation includes "releasing over a hundred dragons". If this is here a metaphor for floodwaters or water spirits, this should be clarified; otherwise, the current phrasing may mislead readers.

In SI S3 section 1.2, memory is described as "implemented as a deque storing recent climate experiences"; for non-programmer readers, it would be clearer to say explicitly that this is a first-in-first-out (FIFO) queue of past climate values up to the agent's memory capacity.

The last sentence before SI Section 2 ("using a standard-deviation filter applied to the right-hand series") is opaque; "right-hand series" should be replaced with a clear description such as "applied to the climate time series used as the objective reference" or similar. Likewise, the phrase that parameters "come from experience" in section 2.1 should be clarified: presumably the authors mean that parameter values are chosen based on expert judgment and not fitted to the data.

In SI section 2.4 the authors suddenly mention three possible baselines (personal memory, full model climate series, and collective archive) whereas earlier sections describe only two operational settings (personal vs collective). It should be clearly stated which baselines are actually used in the analyses and which are only conceptual possibilities. In 2.6 they write that "in its current configuration, the archive primarily serves as a tool for analysis and does not influence individual

decisions", which appears to conflict with earlier references to agents comparing to collective records; this ambiguity should be resolved. In 2.7, the last sentence about "prospective extensions with re-judgment" is difficult to understand and could be rephrased.

The rationale for representing ordinal severity levels via truncated-normal sampling to produce continuous values should be explained. Since the empirical analyses use ordinal categories and Kendall's τ, it is not obvious why a continuous mapping is needed for the ABM outputs; at least, the authors should argue that this choice does not materially affect the correlation patterns. In 2.9, record transmission loss is said to occur with probability loss_rate "(default 0.20, uniform distribution)"; this is not a uniform distribution but a Bernoulli process with parameter 0.2, and the terminology should be corrected. The choice of maximum age 40 in the default configuration also needs a brief justification in light of the discussion of generational life expectancy.

In 3.3 the phrase "filter side (default: True, on climate series)" is implementation jargon and not self-explanatory. This should be rewritten in ordinary language, explicitly stating which series is filtered and why.

In compare.py the correlation defaults likely underconstrain rolling windows, since min(log2(n),2) is almost always <=2.

It seems like the confuson matrix orinetation is not consistent, since you transpose, then the index and column names seem flipped, but I may have missed something here.

The reindexing of the matrix in calibration.py is happening at the level of the local variable (matrix) not applied to the matrix. So, in the end nothing is happening. Unless you are using a different version when this behavior is not the same.

(Remarks on code availability)
I skimmed the code briefly and there seems to be at least one key problem. See report.

Reviewer #3

(Remarks to the Author)
This paper was a pleasure to read. In the abstract, the paper's purpose is clearly stated and articulated in three steps:
A mismatch is shown between historical and instrumental observations;
The mismatch is mitigated by generation-sized sliding windows;
An ABM model reproduces this periodicity.

The paper, in the view of these reviewers, by any means deserves publication. There are, however, some improvements that we think could improve its readability.

From a theoretical standpoint, the paper appears conceptually robust. The Shifting Baseline Syndrome framework is highly compelling, and the attempt to reproduce its underlying mechanisms through an ABM is both coherent and methodologically promising. Furthermore, this approach seems potentially transferable to other domains (e.g., the perception of crime or urban disorder, where what is considered "normal" may also shift across generations). This represents a significant strength of the paper. The authors could give their opinion on transferability, and potentially add other examples to this regard.

It may also be worthwhile to further problematize the nature of the historical archives. These sources may not simply reflect aggregated individual perceptions, but could also be understood as institutional products shaped by administrative conventions and relatively stable classificatory criteria. Clarifying whether possible effects of administrative practices or evolving classification standards over time have been considered would strengthen the argument. Moreover, this issue connects to the 30 year threshold, within which the correlation between historical records and climatic proxy data appears to improve. While this window size produces the strongest statistical alignment, it does not necessarily imply that individuals retain a generational memory of exactly thirty years.

Yet, accuracy of data and coherence of statements should have been more carefully checked; it's really unsettling to see Kendall's tau jump between 0.17 and 0.19 in different parts of the text.

The ABM is presented as a "simple but robust ABM approach" and we agree to that. This usage of ABM sets an example of clever application of the methodology. Having already shown that a generational sliding window improves correlation, the authors use the ABM to reinforce the result and propose an explicit causal mechanism that puts their thesis to work.

While the statement that the paper is, "as an essential step in addressing the challenges of the Anthropocene," might sound, and be, slightly exaggerated, we agree that the paper constitutes an exemplary way of "combining empirical data from archives and paleo-environmental proxies with computational modelling". This sets an example of good practice for the field, that we hope will find diffused application. In the words of the authors, "co-evolutionary models being developed to study human-Earth system interaction should
find a way to incorporate systematic data on the role of collective actions in adaptations and extremes."

The ABM code is available, and documented in ODD, as all ABMs should. Development of the ABM has been made with the use of a platform (https://pypi.org/project/abses/) that remarkably improves replicability. the

In the following, we remark in a more punctual way on the state of the paper.

2 Results

The validation period is not mentioned in the introduction and appears quite abruptly, thus making it a surprise for the reader, disrupting the flow.

In this section, the p-tests don't seem to have been preregistered, and in the opinion of this reviewer $p<0.1$ should not be mentioned.

In section 2.2, if we understand it, tau is 1.18 for the data, 1.17 for validation. In section 2.3, tau is 1.19, same as in the relative figure. Are these different calculations, and why?

Methods

Apart from some typo here and there, the style of presentation can be improved. The reader gets absolutely nothing from the proxy equation. As a rule of thumb, no equations should be in a paper that don't get used later on. Same goes for the C(x) equation that can be better summarized in a table. For Cohen, it appears that, when you state that you have used the quadratic one, you give (and never use again) the linear one.

What is with the values in the C definition? The 10% threshold of the normal is at -1.28, not 1.17.

Small typos, missing verbs and other minor comments can be found in the attached PDF. They must be understood just as suggestions, or pointers to places where small improvement can be made.

(Remarks on code availability)
The code looks reproducible and usable.

Reviewer #4

(Remarks to the Author)
I co-reviewed this manuscript with one of the reviewers who provided the listed reports. This is part of the Nature Communications initiative to facilitate training in peer review and to provide appropriate recognition for Early Career Researchers who co-review manuscripts.

(Remarks on code availability)

Reviewer #5

(Remarks to the Author)
This article is quite fascinating. As someone who works with anecdotal evidence of effects of extremes on ecosystems I have long wondered about shifting baseline syndrome, and was excited to see this empirical test. It seems to me very novel and clever in the way it brings together historical records with climate proxies.

However, at present the work is extremely difficult to follow. Both methods and results are quite opaque. It would be good to make the work more broadly accessible with much clearer explanations and figures. Most of my suggestions below are aimed at improving the clarity of the presentation.

Given that the detailed methods are at the end of the paper, the introduction and results sections need to give some more information about the approach taken.

Figure 1b-c is intended to illustrate the conceptual idea and approach, but is not easy to follow. An extended caption to walk the reader through the diagram would help. In Figure 1b, I think the person on the left is seeing the grey dots as normal and the person on the right is seeing their grey dot as a drought? It's not clear what the green and orange dots are, or why there are now different green and orange dots in Figure 1c. Perhaps rethink Figure 1c to more clearly show how restandardisation works.

Lines 103-104: $r = 0.37$ seems like a relatively low coefficient for the comparison between the instrumental record and the proxies. Lines 105-106, not sure which series is being referred to in this sentence, and it's not clear what is meant by "is generally consistent with".

Figure 2 caption also needs to be extended to more clearly indicate what is shown on the figure. Panel 2a: Clarify what the black line and grey uncertainty region represent. What are the dashed horizontal coloured lines? 2b: What are the values shown here – the methods describe the classification of the historical archives into 5 categories, not the extraction of a numerical value? And why are there only three colours when there are five categories? 2c: The open dots showing the locations of the historical data obscure the region of most interest – consider if there is a better way to show this.

From Figure 2, it appears that there are distinct 'dry' and 'wet' periods in the record. It would be valuable to show the time

series of mismatches as well, as a simple visualisation of whether misclassifications are more likely to appear towards the end of these ‘wet’ and ‘dry’ periods.

Methods:
Line 324: Sentence is incomplete.
Line 346-347: It is not clear to me what probabilities are being extracted here. And why are the probabilities 10, 25, 30, 25 and 10% ? Ah, I see, these are not probabilities as such, but quantiles.
Line 350: The process of spatial integration is also unclear, and could be made more plain. Why does the WDI for each site need to be randomly simulated? What happens when a site is missing data for that year?

Line 358: ‘All the original reconstructions were calibrated with correlations above 0.4’ – not clear what this means.
Line 362: Give a simpler explanation for how these datasets were integrated. Unclear why a different approach for integration is taken for these data than for the historical data, i.e. why not just take means for each year?

Line 378: Please number equations. Note, these SD thresholds do not appear to give the right quantiles? I think the corresponding quantiles are 12, 37, 63, 88% ?

Section 4.2.2 Bias of mismatches detection
I have read through this methods section several times now, alongside the corresponding results and figure, and I am still having difficulty understanding what is being done here. I understand that we are looking at the years where the historical data and natural proxy data do not match. We then go back through the record to find the most recent year with the same historical value (although it is not clear whether this is numerical value or the classified value). We then determine the difference between the natural proxy values of the two years (delta t, t’). However, it is quite unclear to me what is done with this (delta t, t’) value. I cannot understand the results statement ‘In these mismatch cases, the severity level from historical archives is usually categorised as drier (or wetter) than those observed in natural proxies when WDI values are only slightly lower (or higher) compared to a previous year. This “shifted-correlation pattern” is most apparent when the shift is 2, in which case the absolute value of these WDI differences is approximately 0.04 on average’
This section and these results need to be made much clearer and plainer. What is the shift? Does WDI in this sentence refer to the historical data or the natural data? Which WDI differences are we talking about? It might be helpful to come up with some clear notation for each set of data being discussed, and use this notation consistently through the text.

Figure 3(c-f) I also find opaque. I am not sure what the ‘shifted categories’ are. An expanded caption that clarifies what is shown would help.

The re-standardisation approach is somewhat easier to follow. However, I would advocate not using the word ‘filter’ when referring to re-standardisation – this causes some confusion because of earlier processing steps including ‘low-pass’ filtering. I would stick with ‘re-standardised’ data.

Figure 4: Be careful about wording; Figure 4a compares ‘original’ to ‘filtered’ data but I think the ‘filtered’ data here are actually the re-standardised values?
Indicate the value of ‘s’ for Figure 4a. Figure 4b, scale needs an additional decimal place. What do Max tau = 0.22 and Avg tau = 0.10 refer to?

The length of the optimal restandardisation window looks from Figure 4a and b to be around 15-20 years, yet the text states that the outperformance occurs with windows of 20-40 years, with an optimal sliding window size of 34 years. This suggests that the results from the whole time period contradict the results from shorter time periods. I think it would be simpler and clearer to focus on the results from the whole time period, rather than subsets of the data.

The agent-based modelling approach is also novel and very interesting, but could be made clearer. With the ‘collective memory’ setting, which years does each agent use as reference? Do they have the collective memory of the full time period, or only access to the collective memory in their own lifespan? (Line 467, memory seems to start at e(t-2) – please correct).

The simulations could potentially be more interesting if there are long-term variations in the ‘real’ climate: at present, climate is a normal distribution with constant mean and sd, but this could be modified by adopting a low-frequency variation in the mean climate, to simulate inter-decadal variations in monsoon patterns.

Figure 5 only shows the outcome of the ABM after re-standardisation with a particular window size. It would be valuable to demonstrate the outcomes before re-standardisation as well, to show more clearly the effect of SBS.

Again, extending the caption to Figure 5 would assist in understanding. I’m not sure exactly what output is shown in panel (b): the label “correlation differences (times of sd)” is not at all clear. Is this the improvement in correlation between ‘true’ and ‘recorded’ climate with re-standardisation to a given window size? I don’t understand why this is monotonic for the ‘collective’ memory but peaked for the ‘personal’ (or ‘individual’?) memory.

It would also be useful to discuss how the recording lifespan compares with the timescale on which improvement occurs for the personal memory. The typical recording lifespan is 10-60 years – so why is the timescale for improvement so much shorter? I was expecting a more detailed sensitivity analysis around lifespans, but the sensitivity analysis in the Supplementary is quite cursory – and the relevant Figure S5 is not clearly captioned. Please do ensure that figures in supplementary have full explanatory captions.

(Remarks on code availability)
I took a very brief look at the code. I don't use python, so did not attempt to download and run. However, the directory looked to be well organised and well documented.

Reviewer #6

(Remarks to the Author)
The manuscript is well written and based on a very interesting combination of two rich and compelling datasets. The interdisciplinary approach, combining tree-ring data with historical records to explore the influence of collective memory on the interpretation of weather events, is particularly intriguing. I found both the introduction and discussion engaging and easy to follow, while the methods and results sections were somewhat harder to navigate due to their technical nature.
I also appreciated the examples from the historical sources provided in the supplementary material, especially Table S3. If space allows, I think this table could be moved to the main manuscript, as it conveys the nature of the historical sources, while illustrating how they were interpreted.
I am not entirely convinced about the added value of the ABM in its current form. While the idea of comparing individual memory with access to collective memory is interesting, it remains somewhat unclear what additional insights the model provides beyond the empirical comparison between tree-ring data and historical records. I would therefore encourage the authors to clarify more explicitly why the ABM is an integral part of the study.
I also wonder how sensitive ABM was to different remembering ages of individuals? Was the model stress-tested to assess whether the results were robust across different age assumptions? Also, was Lmax set to 40 or 60 years? There seems to be a discrepancy between the manuscript and the supplementary information.
The manuscript uses objective vs subjective when referring to the two datasets, where the tree-ring data is considered objective and the historical archives subjective. This distinction becomes somewhat reductive, since neither dataset is entirely straightforward. Tree-ring records are also interpreted proxy data, shaped by sampling, methodological choices, and assumptions about environmental responses. At the same time, historical archives should not simply be treated as "subjective". I think this distinction could simply be removed.
Figure 2d: how can there be more than five WDI index categories for the historical archival material?
Lines 107–109 state that over 0.8 of the historical archive sites were located in areas showing a positive correlation with the WDI series. What exactly does this mean? Does 0.8 refer to 80% of sites, or something else?
Lines 323–325 appear to form an incomplete sentence.
The figure labels are formatted inconsistently throughout the manuscript. In some figures the panels are marked as "a", while in others they are written as "a.". They are also positioned differently between figures, for example in the right vs left side of the panels.
In Figure 2, the text "Pearson correlation coefficient" also appears to be somewhat awkwardly positioned.
My overall assessment is that this is a very exciting and promising manuscript that could become even stronger with some minor revisions.

(Remarks on code availability)

Version 1:

Reviewer comments:

Reviewer #1

(Remarks to the Author)
This revised article meets my concerns and is now acceptable.

(Remarks on code availability)
This article meets my concerns and is now acceptable. I have not reviewed the code since it is not in my expertise.

Reviewer #2

(Remarks to the Author)
The authors have carefully considered and address all (many) the points I raised. I appreciate their care, the new climate scenarios, and that they did not dismiss any major points. The manuscript is much improved.

(Remarks on code availability)

Reviewer #3

(Remarks to the Author)

All our concerns have been addressed.

(Remarks on code availability)
All our concerns have been addressed.

Reviewer #4

(Remarks to the Author)
I co-reviewed this manuscript with one of the reviewers who provided the listed reports. This is part of the Nature Communications initiative to facilitate training in peer review and to provide appropriate recognition for Early Career Researchers who co-review manuscripts.

(Remarks on code availability)

Reviewer #5

(Remarks to the Author)
I have not gone through the paper in detail as I did not have time - I have just read through the response to my previous comments and the revised version of the manuscript. Nonetheless, I can see that the presentation is much improved - the aims, methods and results are much clearer now - and I am happy with the revisions. Overall, I think this is a really interesting, novel and clever piece of work and I strongly support publication.

(Remarks on code availability)

Reviewer #6

(Remarks to the Author)
I have carefully reviewed the revised manuscript and the authors' responses to the reviewers' comments. The revisions have satisfactorily addressed my concerns, and I have no further comments or suggestions. I recommend acceptance for publication.

(Remarks on code availability)

Open Access This Peer Review File is licensed under a Creative Commons Attribution 4.0 International License, which permits use, sharing, adaptation, distribution and reproduction in any medium or format, as long as you give appropriate credit to the original author(s) and the source, provide a link to the Creative Commons license, and indicate if changes were made.
In cases where reviewers are anonymous, credit should be given to 'Anonymous Referee' and the source.
The images or other third party material in this Peer Review File are included in the article’s Creative Commons license,

unless indicated otherwise in a credit line to the material. If material is not included in the article's Creative Commons license and your intended use is not permitted by statutory regulation or exceeds the permitted use, you will need to obtain permission directly from the copyright holder.
To view a copy of this license, visit https://creativecommons.org/licenses/by/4.0/

Author Response to Reviews of

Archival and palaeoenvironmental documentation of historical extreme events reveals perceptual bias in collective memory

Shuang Song, Bo Hu, David Max Findley, Xutong Wu, Wei Liu, Robert W. Middeke-Conlin, Victor L Caetano, Yuda Yang, Shuai Wang, Jürgen Renn, Patrick Roberts

Nature Communications

RC: *Reviewer Comment*, **AR:** Author Response, Manuscript text

1. Reviewer 1

RC: *This is a fascinating and excellent study that brings scientific data, historical data and cultural data in the form of collective memory together in a way that demonstrates how these seemingly very different areas of knowledge can be aligned to make meaningful discoveries. While the model created by the authors offers a valuable way to assess, understand, provide for and even forecast and mitigate responses to extreme climate events, the alignment of this disparate data through the model is also an important achievement. The authors have been diligent not only in their use of this literature but in their broad selection of historical data. The discussion of collective memory is appropriate and is supported by an extensive literature and this paper extends and adds to that literature, providing an unusual and creative perspective and analyzing collective memory in a unique way. By aligning historical data/collective memory with data from the natural world, the authors have created a model that enables the measurement of collective memory itself. In particular, the introduction of the Shifting Base Syndrome casts an innovative light on both collective memory and perceptual bias.*

AR: We are deeply grateful to the reviewer for this generous and insightful summary of our work, and for the exceptionally positive assessment. We are especially gratified that the interdisciplinary integration of scientific, historical, and cultural (collective-memory) data, the alignment of these heterogeneous sources, and the introduction of the Shifting Base Syndrome were seen as innovative and meaningful contributions.

RC: *The research supports the authors claims about perceptual bias as well as the possibility of utilizing their model in assessing extreme climate events. I see no flaws in their methodology and interpretations.*

AR: We sincerely thank the reviewer for the confidence expressed in the soundness of our methodology and interpretations. We greatly appreciate this endorsement. Incorporating the other reviewers' suggestions, we further strengthened the methods and model sections; we hope you will find the approach more convincing.

RC: *My field is Archival Studies where both historical data and collective memory are paramount concerns. This study offers exciting new perspectives and possible applications for greater understandings in both of these areas.*

AR: We are honoured that a scholar of Archival Studies, a field for which historical data and collective memory are central, finds our framework exciting and potentially applicable to these domains. We hope our approach can foster further dialogue across the natural and archival/historical sciences, and we thank the reviewer warmly for this perspective.

RC: *This research is extremely detailed and the authors have carefully described their formulas and model and so it can be replicated.*

AR: We thank the reviewer for recognising the level of detail in our description of the formulas and model, and for noting that the study is reproducible. Reproducibility was a key priority for us, and we are pleased that this goal was achieved.

RC: *While I have accessed the code, this is not my area of expertise and so I have not installed it.*

AR: We thank the reviewer for taking the time to access our code, and we fully understand that installation lies outside the reviewer's area of expertise. To facilitate reproducibility for readers with a computational background, the repository

includes step-by-step installation instructions, an environment specification, and runnable scripts that regenerate the main results; the code is openly available and documented in the Code Availability statement. We would be happy to provide any further clarification if helpful.

2. Reviewer 2

RC: *This manuscript combines a four-century archival Wet/Dry Index (WDI) for northern China with tree-ring-based hydroclimate reconstructions and an agent-based model (ABM) to argue that Shifting Baseline Syndrome (SBS) imprints generational-scale biases on collective memory of climate extremes. Empirically, the authors show systematic mismatches between a "subjective" archive-based WDI and an "objective" proxy-based WDI, and that re-standardising the proxy series within 20–40-year sliding windows increases rank correlation. An ABM with agents that remember only a finite past is then claimed to reproduce an optimal window of ~30 years. My overall assessment is that the topic and basic idea are interesting and potentially significant, but the current manuscript is far from the level of scientific accuracy and clarity required for publication.*

AR: We thank the reviewer for careful and constructive reviews and for recognising the interest and potential significance of this research. We have taken the comments seriously and substantially strengthened the codebase, analysis and writing. In particular, we: (i) corrected the code issues the reviewer identified. Two of them were merely code formatting or redundancy, and the changes do not affect the results. The other two have a limited impact on the results but does not challenge the core conclusions; (ii) added climate-forcing scenarios for a robustness matrix and a full two-stage global sensitivity analysis (Morris + Sobol) to the ABM; (iii) reframed the ABM as a mechanism-discrimination test of SBS ("Shifting Baseline Syndrome", generational amnesia v.s. collective memory illusion[1]); and (iv) revised the research-question framing, the Monte-Carlo description, the main-text ABM description, and the figures for clarity. The point-by-point responses below detail each change. We are pleased to report that none of the corrections alters our central conclusions and that they also significantly improve the framing of our manuscript and research. We thank you again for your thorough review and hope the new version addresses your concerns.

Major comments

RC: *A central strength of the design is the use of an instrumental validation period (1901–2000 CE) in which the validation comparison is strictly between the integrated natural-proxy WDI and independent gridded precipitation datasets. In that period the subjective archival WDI is not used to construct either series, so collective memory cannot contaminate this particular validation step. Historical archives certainly still exist after 1900, but they are not part of the proxy-based WDI or the instrumental datasets, and in that limited sense they are indeed excluded from the comparison.*

AR: We thank the reviewer for correctly summarising the design of our instrumental validation period and for affirming that collective memory cannot contaminate this validation. The 1901–2000 CE division is a deliberate design that isolates a window during which the proxy can be checked against instruments without archival input. It does not imply that collective memory ceases to operate after 1900.

RC: *However, the main research questions as formulated in the Introduction are not actually answered by the methods applied. The authors state that they "test the degree to which period-specific perception bias influences the formation of historical narratives of extreme climatic events" and that perceptual bias leads to a "generational offset in collective memory". What they measure empirically are (i) discrepancies between categorical WDI assignments in two already-constructed series, (ii) changes in Kendall's τ under sliding-window re-standardisation of the objective WDI, and (iii) the behaviour of a stylised ABM driven by i.i.d. normal climate noise. These are not direct measurements of the "formation of historical narratives" or of a "degree" of bias in narrative construction. The archival WDI is itself a heavily processed regional index, which has already collapsed many qualitative narratives into a single ordinal time series. Without an explicit, operational definition of "formation of narratives" and a corresponding quantitative metric, the strong claims about how narrative formation is influenced by period-specific perception bias are not supported by the current analyses. This could be solved by rewriting the research question.*

AR: We thank the reviewer for these valuable comments. We acknowledge that our approach lacks a direct measure of perceptual bias during the "formation of narratives"; instead, it reflects the offset and improvements in the aggregated Wet/Drought Index between the historical archives (H-WDI) and natural proxies (N-WDI). Therefore, we have rephrased the research question based on the reviewers' suggestions and emphasised more clearly how this difference reflects Shifting Baseline Syndrome (SBS), a perceptual bias highly relevant to collective memory studies. Modifications include:

- We no longer use the misleading phrasing "formation of historical narratives".

- Before introducing the specific research questions, we first supplement the current theoretical discussion of SBS by acknowledging that it is only one of many perceptual biases and by noting that SBS originates from collective factors. Thus, we clarify that we do not measure perception biases or historical narratives at the individual level, making it reasonable to aggregate existing historical archives (e.g., H-WDI) in this context.

Manuscript · Page 2, Line 57–68

Among the many perceptual biases, Shifting Baseline Syndrome (SBS)[2], also referred to as Environmental Generations Amnesia (**Figure 1a**)[1], suggests that the decayed memory of environmental changes across generations arises from comparisons to a "shifting" baseline. The baseline serves as a reference point for comparison and a perceived "normal" based on limited memory and experience[1], thereby resulting in cross-generational inconsistencies in environmental perception and undermining the potential for collective action[3,4]. This inconsistency in collective memory may stem from multiple sources, but collective factors, including generational amnesia or collective illusion, were suggested as the main roots of SBS (**Figure 1b**)[1]. Generational amnesia occurs when younger generations lack knowledge of past environmental conditions, whereas collective memory illusion arises when shared narratives or expectations distort how past conditions are remembered[1]. A recent study suggests that SBS is widespread across diverse contexts[5]. Nevertheless, long-term evidence remains lacking on whether and how SBS shapes collective memory of environmental change.

- Then, we explicitly attribute the current research gaps directly to discussions of "SBS" in order to verify whether it plays a role in collective memory (represented by historical texts) and how it works:

Manuscript · Page 2, Line 69–73

Here, we develop a novel approach to test whether SBS plays a significant role in period-specific perception bias and in shaping historical representations of extreme climatic events over long-time scales (here defined as centuries or cross-generational). We test two hypotheses: (1) historical archives of climatic extremes are influenced by contemporaneous perceptual bias and (2) this perceptual bias leads to a generational offset in collective memory relative to 'natural' observations of climatic variation.

- Finally, we explicitly explain how our approach helps fill the gaps at the end of the introduction, with an improved Figure 1 presenting the overall research design:

Manuscript · Page 3, Line 92–98

If SBS existed, mismatched records are expected to exhibit a systematic over- or underestimation pattern (static), which can be addressed by restandardization within sliding windows —i.e., correcting for generational differences (dynamic). Finally, to interpret the mechanism, we examine two hypotheses (generational amnesia or collective memory illusion) and further develop a heuristic agent-based model (ABM) to explain the identified SBS, testing whether it reproduces the same patterns (**Figure 1d**). From data to model, our approach aims to systematically and quantitatively reveal the long-term role of collective memory in climatic recording, experience, and, thus, adaptation.

RC: *Relatedly, the notion of "degree to which period-specific perception bias influences the formation of historical narratives" lacks a clear sense of direction or observable quantity. It is not specified whether "more formation" should mean more narratives per event, more detailed descriptions, higher probability of recording, or something else. Nor is there any attempt to count or otherwise quantify the number of distinct historical narratives as a function of climate anomalies. In practice, the paper infers perceptual bias solely from the relative alignment of an archive-based index and a proxy-based index. This is a much narrower question than the one formulated in the Introduction, and the framing should be revised accordingly or the analyses significantly expanded.*

AR: After revising the research questions (see above), we also removed overly broad expressions such as "measure degree to..." and "formation of historical narrative". The current statements are now reframed to (1) quantitatively demonstrate that SBS has played a role in collective memory represented by historical records; (2) use an agent-based model

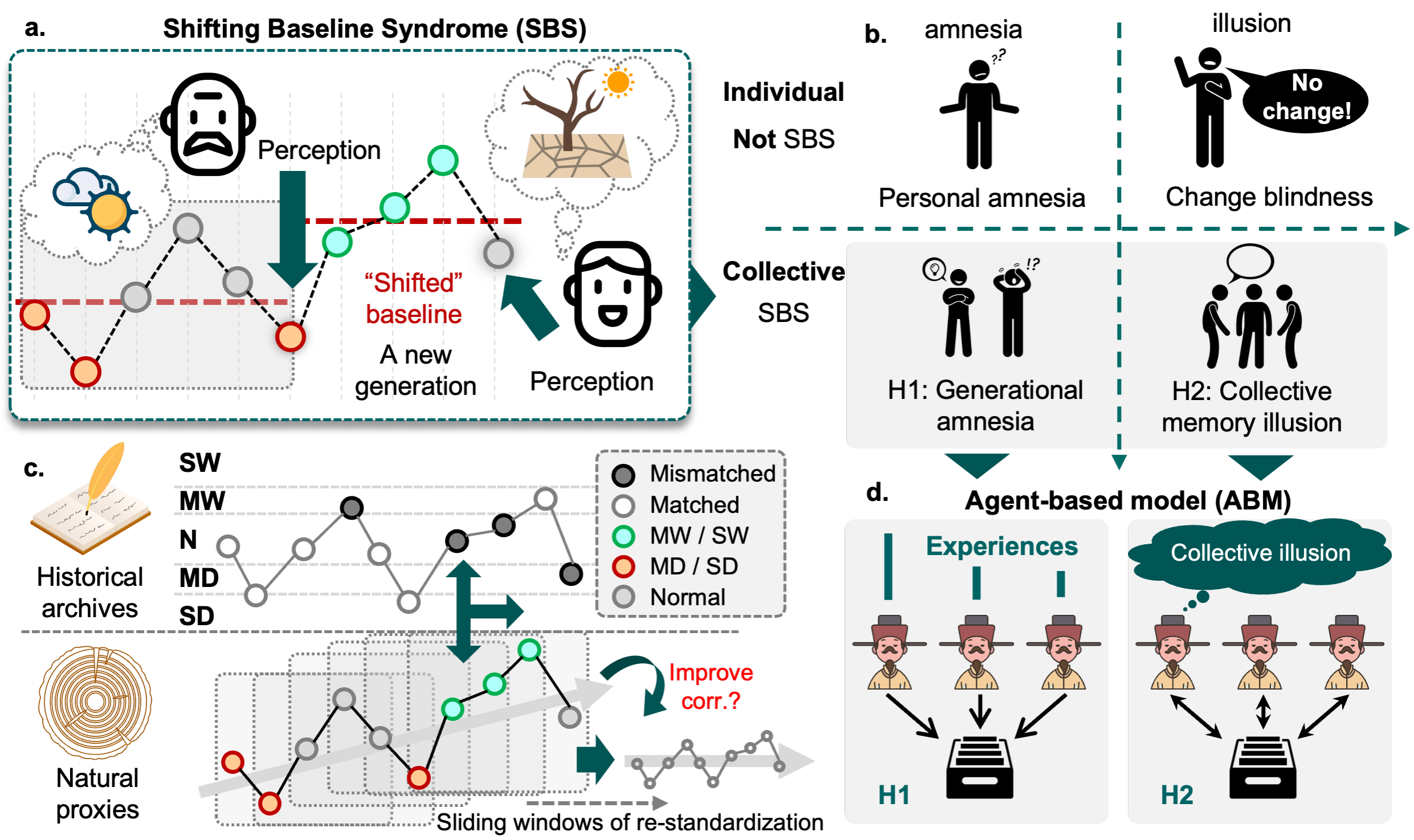

Figure 1: Methodology overview of this study. **a.** Diagram of the Shifting Baseline Syndrome (SBS). The new generation grew up in a different environment from the previous one, so their reference baseline for environmental change is also different. For example, in a climate that is becoming increasingly wet, early generations would consider a year that is actually relatively dry to be normal, while later generations would consider a normal year to be relatively dry. **b.** Perceptual bias behind inconsistencies in perceptions of environmental change. The theory of SBS suggests that this bias stems from collective factors, including differences in experiences across generations (**H1**: generational amnesia) and a collectively misleading consensus about the past environment (**H2**: collective memory illusion). **c.** Schematic diagram of how standardisation corrects the perceptual bias. We compare two climatic extreme datasets (historical archives and natural proxies, processed as a Wet/Dry Index, i.e., WDI), which can be categorised into five severity levels: Severe Wet (SW), Moderate Wet (MW), Normal (N), Moderate Dry (MD), and Severe Dry (SD) based on z-scores (see **Methods** and **Supplementary Information Figure S3** for details). We measure the mismatch patterns between two datasets and test whether their correlation can be improved by sliding-window restandardization, which assumes a continuously changing "baseline" within a specified window size (see **Methods** for details). **d.** We used an agent-based model to test two hypotheses behind SBS: that agents from different generations rely only on personal experience or on a collectively accumulated, documented record of the climate, to examine how the observed pattern of generational offset emerges.

(ABM) to suggest the underlying mechanisms of SBS. This reframe is directly reflected in our **Figure 1** embedded above. Thanks to the reviewer, this modification makes the research questions more clearly focused, but it does not narrow the study's significance.

RC: *The "Monte Carlo approach" used in the mismatch analysis needs clearer definition and more precise presentation. In Methods 4.2.2, the authors describe generating synthetic climate series (random normal) and randomly assigning historical classifications according to observed category frequencies, repeating this 100 times to build a null distribution for the mismatch metric. Methodologically, this is a Monte Carlo randomisation under a specified null model. However, in the main text and Figure 3 caption it is referred to only as a "Monte Carlo approach", without stating what is randomised, what the null hypothesis is, or how many simulations are used. As it stands, this is too imprecise to count as a clearly described Monte Carlo analysis and will be opaque to many readers. The authors should explain in the main text that they perform a Monte Carlo randomisation test, specify the null model (independent random classifications with fixed marginal frequencies), and justify the number of iterations or increase it if necessary.*

AR: We thank the reviewer for this helpful point and agree the test was under-described. We now (i) state explicitly in the main text that significance is established with a Monte Carlo randomisation test, (ii) specify the null model explicitly: in each of 1,000 iterations, archival severity categories are independently redrawn from fixed atlas-level marginal frequencies, continuous natural values are drawn from $N(0, 1)$ and classified with the same thresholds as the proxy data, and the bias matrix is recomputed with the same within-grade look-back procedure ($shift = 1, 2, 3$) as in the empirical analysis. This null preserves the archival marginals and the look-back mechanics but removes any dependence between archival assignments and the natural sequence; cell-wise significance is assessed against the resulting null distribution of bias matrices; and (iii) state the number of iterations is 1000: the previous texts in **Methods** mistakenly reported 100 iterations, whereas the analysis used 1,000; we have corrected accordingly.

- To keep it concise, we summarise the test in a single sentence in the main text and place the full specification in the **Methods** section. The revised main-text sentence now reads:

> **Manuscript · Page 5, Line 141–144**
>
> This "shifted-correlation" pattern in the historical archives exceeds that expected under a Monte Carlo Randomisation Null (1,000 iterations; see **Methods**), in which archival severity categories are independently redrawn at fixed marginal frequencies.

- The expanded Methods section now states the null hypothesis explicitly:

> **Manuscript · Page 18, Line 439–452**
>
> We assessed the statistical significance of this bias pattern with a Monte Carlo randomisation test that compares the observed bias matrix against a null model in which archival severity categories are assigned independently of the natural climate sequence. The null hypothesis is that archival classifications depend only on their fixed marginal frequencies $P(H = j) = p_j$ ($\{0.10, 0.25, 0.30, 0.25, 0.10\}$), with no association between archival grades and the natural series. Each iteration generated a synthetic dataset in which natural climate values $x_{\text{sim}} \sim \mathcal{N}(0, 1)$ were binned into five severity levels using the same thresholds applied to the proxy data, and archival classifications were independently redrawn from p_j. We ran 1,000 iterations, each with a sample size matching the empirical record length ($n = 432$). For each iteration k, the bias matrix $\Delta^{(k)}$, indexed by natural level i and archival level j, was recomputed with the identical look-back algorithm used on the empirical data (the same $shift$ and within-grade pairing $N_{t'} = N_t$, $t' < t$). This null preserves the archival marginals and the look-back mechanics but destroys any dependence between archival assignments and the natural climate sequence. For each matrix cell (i, j), we computed standardized scores $z_{ij} = (\Delta_{ij}^{\text{obs}} - \mu_{ij}^{\text{null}})/\sigma_{ij}^{\text{null}}$, where μ_{ij}^{null} and $\sigma_{ij}^{\text{null}}$ are the mean and standard deviation of $\Delta_{ij}^{(k)}$ across the 1,000 iterations, and obtained two-tailed p-values as $p_{ij} = 2(1 - \Phi(|z_{ij}|))$.

- The **Figure 3** caption now describes the same randomisation null rather than merely referring to a "Monte Carlo approach".

Manuscript · Figure 3

Significance is assessed by a Monte Carlo randomisation test: H-WDI levels are independently redrawn at fixed frequencies, and synthetic natural values are drawn from $\mathcal{N}(0,1)$, and the same look-back procedure ($shift = 1, 2, 3$) is applied (significant bars are filled in colour, where * denotes p < 0.05 and ** denotes p < 0.01; see **Methods**).

RC: *The ABM description in the main text is highly insufficient, even for readers familiar with ABMs. Section 2.4 and Figure 5 give only a schematic overview ("a batch of observers who perceive climatic extremes and may record their perceptions of severity levels", with two baseline options), while all substantive detail is pushed to SI S3. For a general-audience journal, the main text should summarize at least the nature of the climate process, the agents' memory structure, the decision rule for recording, the demographic process, and how the model outputs are converted into a WDI-like series. As written, it is impossible to understand from the main text alone how Figure 5 was produced or in what sense the model "reproduces" the empirical pattern. The description should also make explicit that anchoring and negativity bias, which are emphasised in the SI as theoretical foundations, are actually implemented in the recording propensity and baseline construction; at present, these cognitive mechanisms are not mentioned in the modelling section of the main text, and anchoring appears only later in the Discussion as a general cognitive process, not as something concretely encoded in the ABM.*

AR: We thank the reviewer for this constructive comment and agree that the main-text ABM description was too thin. We have expanded the main text to summarise the model while keeping the Results section concise, moved the full specification to Methods, and made the two cognitive mechanisms explicit and traceable to their implementation in the code.

- We added a compact "model in brief" to the main text (Results, **Section 2.4**) that names the climate process, the agents' memory structure, the recording decision rule, the demographic process, and the conversion of model output into a WDI-like series:

Manuscript · Page 7, Line 168–176

The heuristic ABM represents climate as a stationary AR(1) stochastic process ($c_t = \phi c_{t-1} + \varepsilon_t$, **Figure 5a**; alternative independent and trended forcings are tested for robustness in **Supplementary Information S3 and Figure S9**) observed by a continuously turning-over population of finite-lifespan agents. Each agent judges the current anomaly against a baseline, and is more likely to log an event the more it departs from that baseline (**Figure 5c**). We designed scenarios to represent the "baseline", based on lived experience or shared archives of agents, corresponding to the two hypotheses of "H1 generational amnesia and H2 collective illusion" (**Figure 5c**), with two other scenarios tested for robustness (see **Supplementary Information S3, Table S10 and Figure S8**). The archive contains logged severities from all agents and is aggregated and classified into the same five-level WDI series, so the simulated and empirical records are compared identically (see **Methods**).

- For the beginning part of the ABM section in **Methods**, we now make explicit that anchoring and negativity bias are concretely encoded:

Manuscript · Page 18, Line 470–476

We further developed an ABM to investigate how collective climate perception can emerge from individual observers' experiences and perceptual biases. The model simulates a population of climate observers who record climatic extremes based on two core cognitive mechanisms: (1) more "abnormal" extremes are more likely to be recorded, implementing a negativity bias[6] in the recording propensity (the probability p_t defined below); and (2) observers assess the severity of an extreme relative to a "memory anchoring"[7] (the standardised-deviation perception $z_{i,t}$ defined below). We test two scenarios (limited experience or collective archives) and expect similar patterns to emerge between the synthesised N-WDI and H-WDI and the real-world datasets.

- We also state explicitly the model architecture in the main text, including the nature of the climate process, the agents' memory structure, the decision rule for recording, the demographic process, and how the model outputs are converted into a WDI-like series:

> **Manuscript**
>
> **Model Architecture**
> The climate forcing c_t is generated by a stationary stochastic process over a simulation period of several centuries. In the main analysis it follows an AR(1) autocorrelated process, $c_t = \phi c_{t-1} + \varepsilon_t$, with climate persistence $\phi = 0.5$ and innovations scaled to climate variability $\sigma_c = 1$. Independent Gaussian noise ($\phi = 0$) and linear-trend variants are evaluated for robustness (see **Supplementary Information S3** and **Figure S9**). The model maintains a global archive $\mathcal{A}_t = \{e_{t,1}, e_{t,2}, \ldots, e_{t,n_t}\}$ recording all extreme events documented at time t, where n_t represents the number of active observers capable of recording events. Climate observers are implemented as N autonomous agents characterized by finite lifespans, where typically $L_{\min} = 10$ and $L_{\max} = 40$ years represent their start and end ages for recording. Each observer maintains memory capacity implemented $M_i(t) = \{c_{t-L_i+1}, c_{t-L_i+2}, ..., c_t\}$ constraining itself to recall experiences of climate. Climate perception operates through a standardized deviation calculation relative to the agent's reference baseline (personal or collective; see below). For agent i at time t, the perceived climate anomaly is $z_{i,t} = \frac{c_t - \mu_i(t)}{\sigma_i(t)}$; where $\mu_i(t)$ and $\sigma_i(t)$ are the mean and standard deviation of the "baseline" that agent i is referring to. The setup of baselines is shown in the section of experimental settings below.
> According to a probability p_t, the observer may record its perception in severity of climatic extreme s_i^t at each time step t, so that the global archive is the average judgment from all observers $e_t = \frac{1}{n}(\sum_i^n s_i), n \leq N$. Not every recorded judgement enters the public archive: each event is retained only with probability $1 - \lambda$ and is otherwise forgotten (the archive loss rate λ, default 0.4). The probability p_t is estimated by $p_t = f_0 + 0.5 - \Phi(|z_{i,t}|; \text{scale} = 1)$, where f_0 represents baseline recording probability typically set to 0.1, and $\Phi(\cdot)$ denotes the survival function of the normal distribution. This formulation ensures that extreme deviations approach recording probability of 1.0, while normal years maintain low but non-zero recording likelihood (see **Supplementary Information S3** for details). The per-year aggregate record e_t is then discretised into the same five ordinal WDI categories used for the archives (using the identical standardised thresholds applied to the empirical data), yielding a simulated archival series. This series and the underlying climate c_t are compared using the same sliding-window re-standardisation with Kendall's τ used in the empirical analysis. The model maintains demographic stability through continuous population turnover. At each time step, N_{new} agents enter the population while agents exceeding maximum lifespan are removed, thus creating overlapping generational cohorts with varying memory spans. Model outputs are produced only after the number of observers stabilises at the maximum N.

- In what sense the model "reproduces" the pattern is now stated in the **Results** paragraph:

> **Manuscript · Page 7, Line 176–181**
>
> Reproducing the empirical observation measured in historical archives, the correlations between recorded climate by agents and the input climate forcing show the unimodal pattern at an intermediate generational restandarised window under the scenario of "H1: generational amnesia" (**Figure 5b**, red and black lines). In contrast, the scenario "H2: collective illusion" shows a monotonically increasing trend with window length and no generational optimum (**Figure 5b**, blue line).

RC: *In the ABM code, the z-score formula is wrong due to operation precedence:* `climate - baseline/std` *should be* `(climate - baseline)/std`*. This must completely change the results.*

AR: We are grateful to the reviewer for catching this. It was a genuine bug in the observer's perception/standardisation step, and we have corrected it to the properly parenthesised. Fortunately, the fix does not change too much:

- The previous error occurred only in the modelling part and did not affect the preceding quantitative analysis.

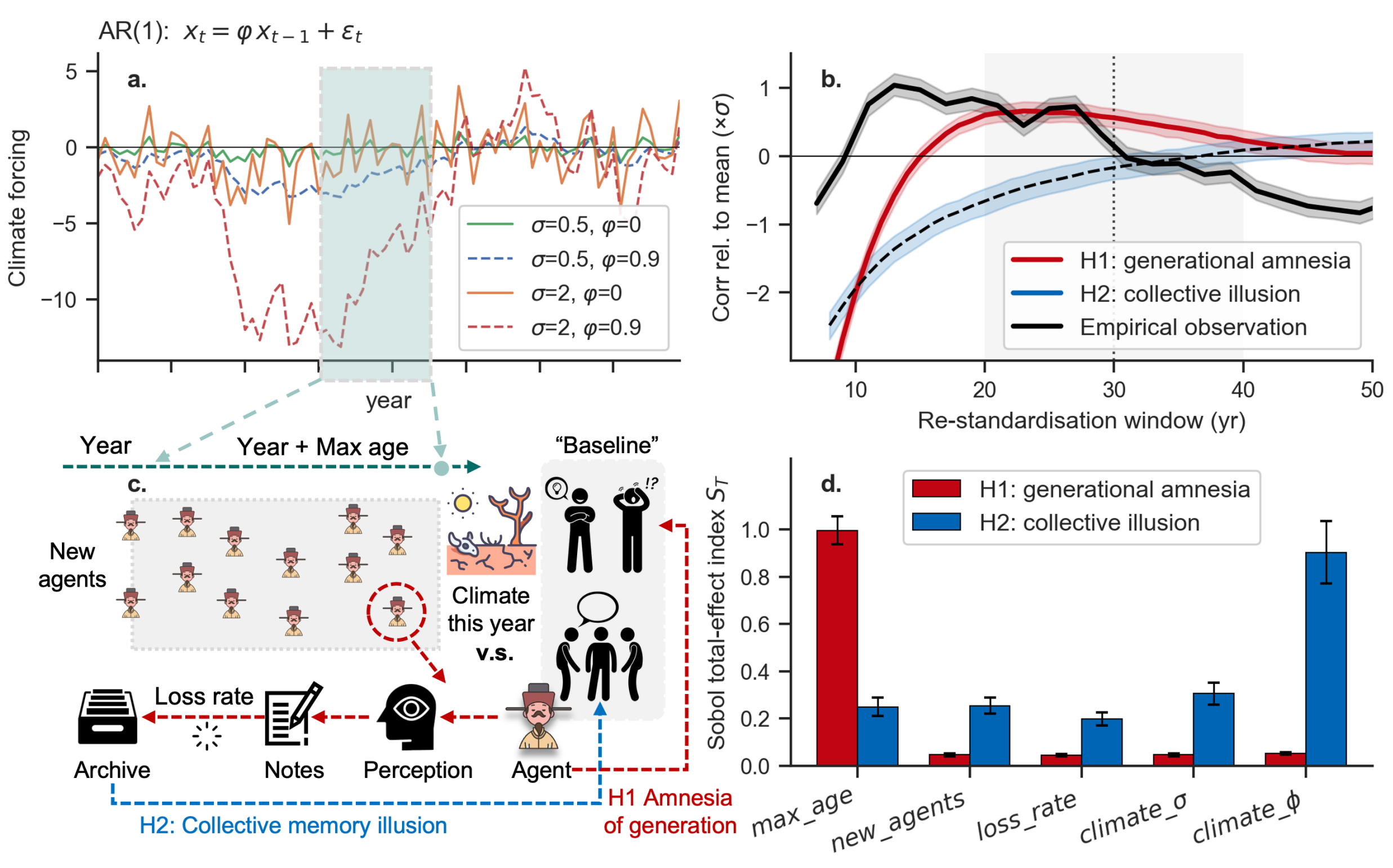

Figure 5: The schematic diagram and results of the agent-based model (ABM). **a.** The stochastic process generating the annual climate forcing c_t; the main analysis uses an AR(1) process ($c_t = \phi c_{t-1} + \varepsilon_t$), with i.i.d. and linear-trend variants tested for robustness (**Supplementary Information S3**). **b.** Under the hypothesis H1 (generational amnesia, red) rather than H2 (collective memory illusion, blue), we ran the ABM 100 times for each scenario. We then compared the severity differences between climate forcing and the recorded archives, and then computed the optimal sliding window size w using the same approach. The pattern of correlation between the model-simulated H-WDI and N-WDI changes with w more closely matches the empirical curve (black, normalised for comparability). **c.** During the specific time period, the number of agents present is related to the number of new agents per year and the lifespans of those agents. Each agent has a perception of that year's climate and may write it down in a public archive. Scenarios H1 and H2 concern whether the baseline agents use when perceiving comes from their lived experience or from shared information in the archive. **d.** Sobol total-effect (S_T) sensitivity analysis on the ABM's relevant parameters under two major scenarios (H1 v.s. H2).

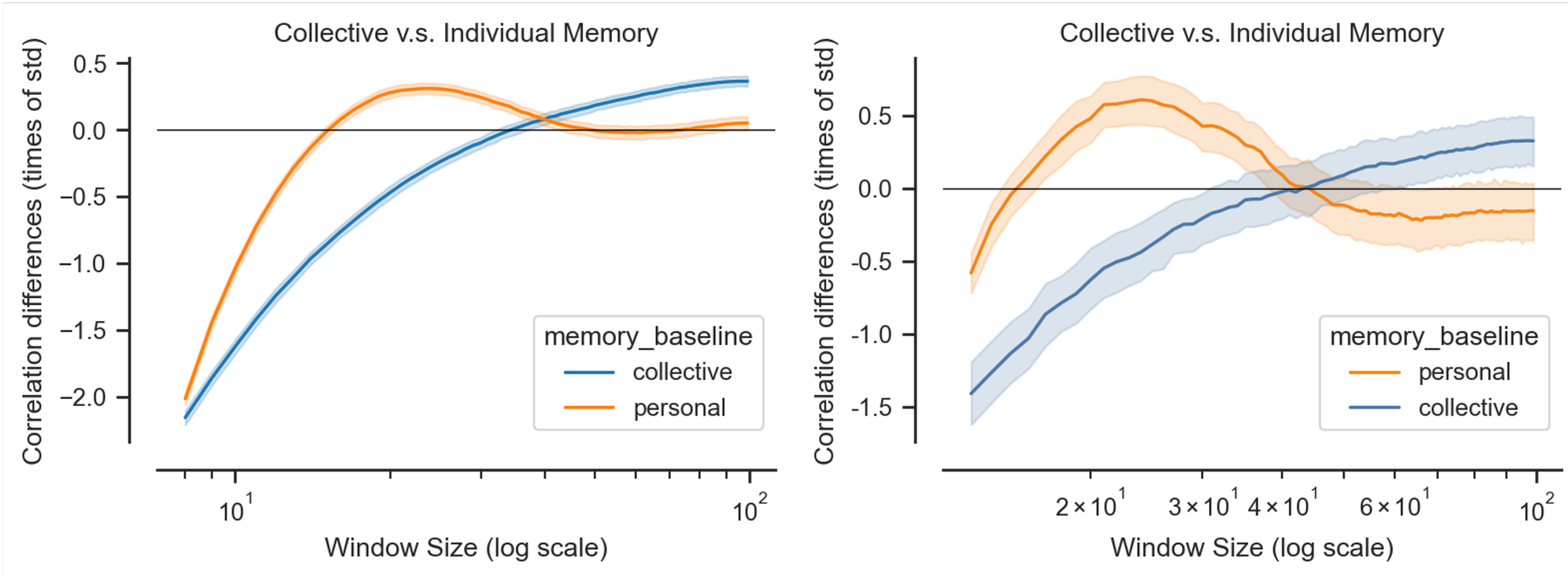

Figure R1: Code-bug comparison. The left panel shows the old figure under the wrong z-score algorithm, while the right panel shows the corrected figure. Note that both figures use the original `idd` climate model for direct comparison of the impact of this bug, while the new manuscript already uses the AR (1) climate forcing model.

- The error had escaped our checks because both variables were already standardised (z-scores), so the mis-grouped expression still produced numerically plausible values rather than visibly anomalous ones and was therefore overlooked. After the correction, the error/uncertainty band widens, but the previously reported pattern persists and remains statistically significant. We have re-run the affected figures with the corrected code. The differences are limited:

Response figure (code-bug comparison) R1.

RC: *The temporal and climatic structure of the ABM needs stronger justification and exploration. Time advances in annual ticks and the climate series is generated as N(0,1) at each step. The annual timestep is chosen to match the yearly WDI, which is reasonable, but it ignores trends and persistence that are likely present in actual climate. The authors then interpret the ~30-year optimal sliding window emerging from this model as supporting a generational SBS mechanism. Given the simplistic climate process, the untested sensitivity of the model output to various assumption, this claim is fragile. The model's results may be highly sensitive to the arbitrary timestep (e.g. if the model ran at seasonal resolution, the effective memory window in years could change), there is a peculiar age structure, no population growth, and no trend or autocorrelation in weather. The statement that "collective climate records aligned with the continuous climate" is also misleading: in the model, climate is not a continuous physical process but independent Gaussian noise. At minimum, the authors should justify the annual step in terms of the empirical WDI and discuss whether and how alternative temporal resolutions or realistic climate processes (with trend and persistence) would affect the emergent memory window.*

AR: Thank you for raising the concern about robustness of the climate forcings. We have addressed this directly with a climate-forcing robustness matrix. The ABM now supports three forcing processes: i.i.d. Gaussian white noise, AR(1) persistence, and a linear trend with noise, at two temporal resolutions (annual, and sub-annual at four steps per year aggregated back to annual values, so all peak-window statistics remain in yearly units). Changes have been made accordingly:

- As mentioned above, our main text uses experiments under the AR(1) climate forcing, which also allows us to examine the sensitivity of two parameters in the ABM:

> **Manuscript · Page 19, Line 478–480**
>
> The climate forcing c_t is generated by a stationary stochastic process over a simulation period of several centuries. In the main analysis it follows an AR(1) autocorrelated process, $c_t = \phi c_{t-1} + \varepsilon_t$, with climate persistence $\phi = 0.5$ and innovations scaled to climate variability $\sigma_c = 1$.

Manuscript · Page 20, Line 521–525

We also conduct a global, variance-based Sobol sensitivity analysis (Saltelli sampling, $N = 1024$ base samples, each parameter vector evaluated over 30 stochastic repeats), reporting both first-order (S_1) and total-effect (S_T) indices over five parameters: (1) maximum lifespan $L_{\max}$, (2) the number of new agents per year N_{new}, (3) the archive loss rate λ, (4) climate variability σ_c and (5) climate persistence ϕ, to identify which factors govern the location of the generational optimum.

- The main text's **Figure 5** already shows that under an AR(1) climate forcing (closer to real climate), "H1 Generational amnesia" still produces the pattern with an optimal window and is insensitive to climate parameters. The other scenario, "H2 collective illusion," is sensitive to climate parameters but cannot reproduce the patterns observed in empirical data.

Manuscript · Page 10, Line 181–187

Global (Sobol) sensitivity analysis traces this divergence to its causes by measuring all five parameters (max age, number of new agents, archive loss rate, climate persistence, and variability; **Figure 5d**). Under the scenario H1, the location of the optimal window is determined almost entirely by an agent's lifespan (maximum effect index $S_T = 1$; **Figure 5d**) and is directly proportional to it (see **Supplementary Information Figure S7**). In contrast, if one assumes that agents use the shared archive as the baseline (H2), the above pattern cannot be reproduced (**Figure 5d**), regardless of how long agents have access to the archives (**Supplementary Information Table S10 and Figure S8**).

- This recurrent pattern of ABM under the "H1 generational amnesia" scenario was also tested under different climate scenarios, and the results were stable. Across all six configurations (10 replicates each), the emergent optimal window remains within the ~20–40-year generational band, confirming the result is not an artefact of the previous i.i.d. assumption, of climate persistence/trend, or of the annual timestep:

RC: *Demographic assumptions in the ABM also warrant more careful treatment. The model introduces a fixed number of new agents each year and removes agents at maximum age, with no dependence of births or deaths on current population size. This produces a flat age distribution between the minimum and maximum ages. Because each agent's memory window and thus their baseline length depends on age, the resulting collective memory is strongly shaped by this artificial age structure, which does not resemble actual historical demography. The authors link their empirical 20−40-year sliding window to "the generational life expectancy of humans in ancient China", yet the default model uses a maximum age of 40 years (in the SI) or 60 years (in the main Methods) and a recruitment process that is not tied to any empirical demographic data. They report an ANOVA-based sensitivity analysis on a limited set of parameters, for which they don't report effect sizes, but this is not a global sensitivity analysis in the usual sense: there is no Sobol or Morris-type decomposition to quantify how much of the variance in the key output (the location and magnitude of the correlation peak) is attributable to each parameter and to interactions. Without such an analysis, it is hard to know whether the emergence of a ~30-year optimum is robust or depends delicately on a particular combination of lifespan, recruitment, loss rate, and climate variance, beyond all the remaining structural assumptions. I would strongly recommend either performing a basic global sensitivity analysis or, at minimum, substantially expanding the current sensitivity study and clearly describing how parameters are sampled and how their influence on the outputs is quantified.*

AR: Thank you to the reviewer for this comment, which significantly enhances the robustness of our method. We have now followed your recommendation and made improvements in three aspects: (1) using a more comprehensive sensitivity analysis; (2) explicitly linking demographic parameters to actual historical data; and (3) noting that, as we use it as a heuristic model, we do not intend to exactly copy historical population structures.

- We replaced the ANOVA with a full global sensitivity analysis (SALib: Morris screening followed by a Sobol variance decomposition with bootstrap confidence intervals), run independently for all scenarios over five parameters (**Figure 5d**). According to the results, for the H1 scenario that replicates the empirical data patterns, population life expectancy (max age of agents) is the most influential (almost dominant) factor.

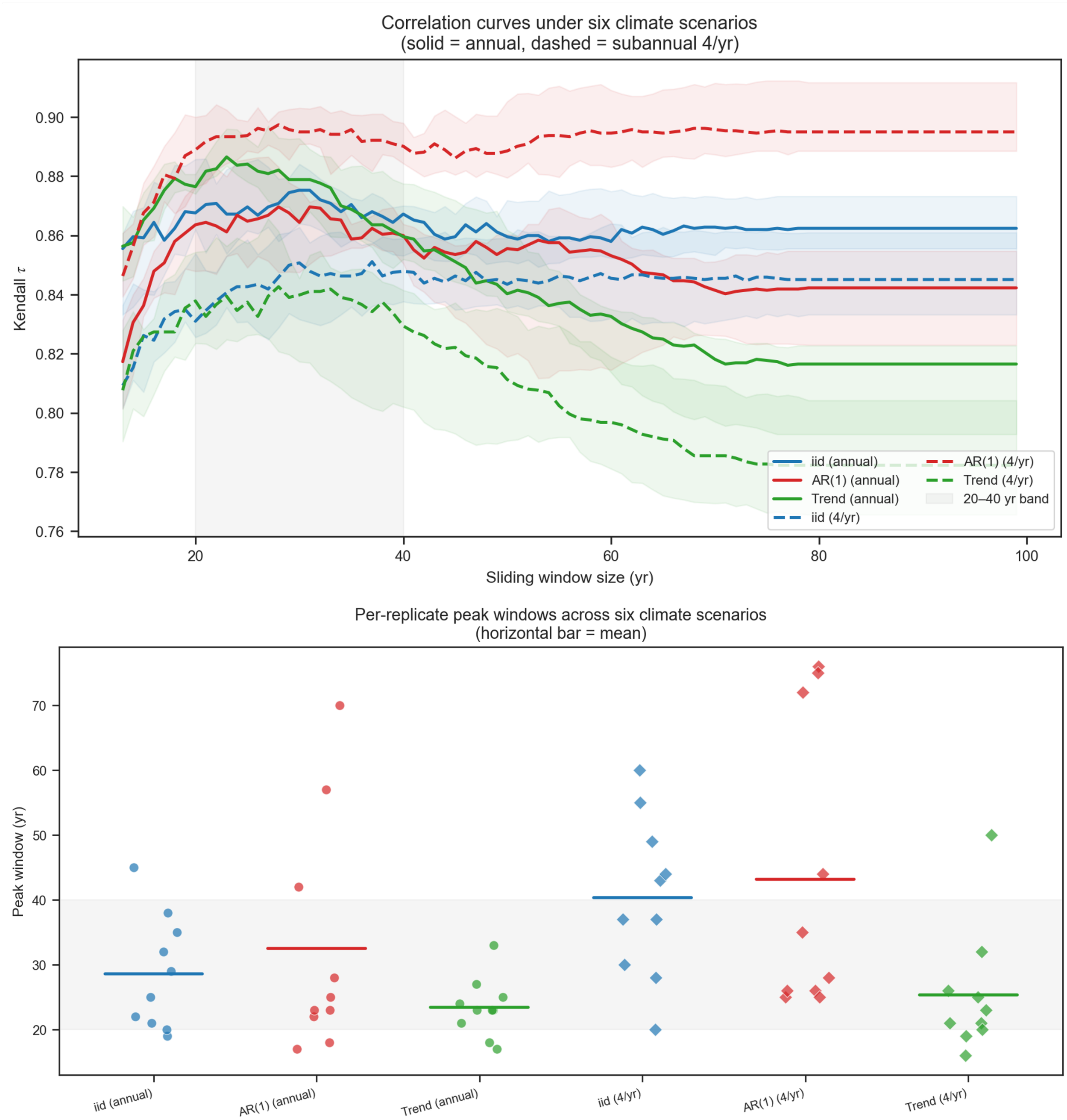

Figure S9: Climate-forcing robustness matrix for the H1 (`personal`) scenario. **Top:** window–τ correlation curves for six forcing scenarios —three stochastic processes (i.i.d., AR(1), trend-plus-noise) at two temporal resolutions (solid = annual; dashed = sub-annual, four steps per year aggregated to annual units); the shaded band marks the 20–40-year generational range. **Bottom:** per-replicate peak (optimal) windows for the same six scenarios (horizontal bars = means). Across every forcing the peak windows cluster within the generational band, confirming that the emergent generational optimum does not depend on the choice of climate process or temporal resolution.

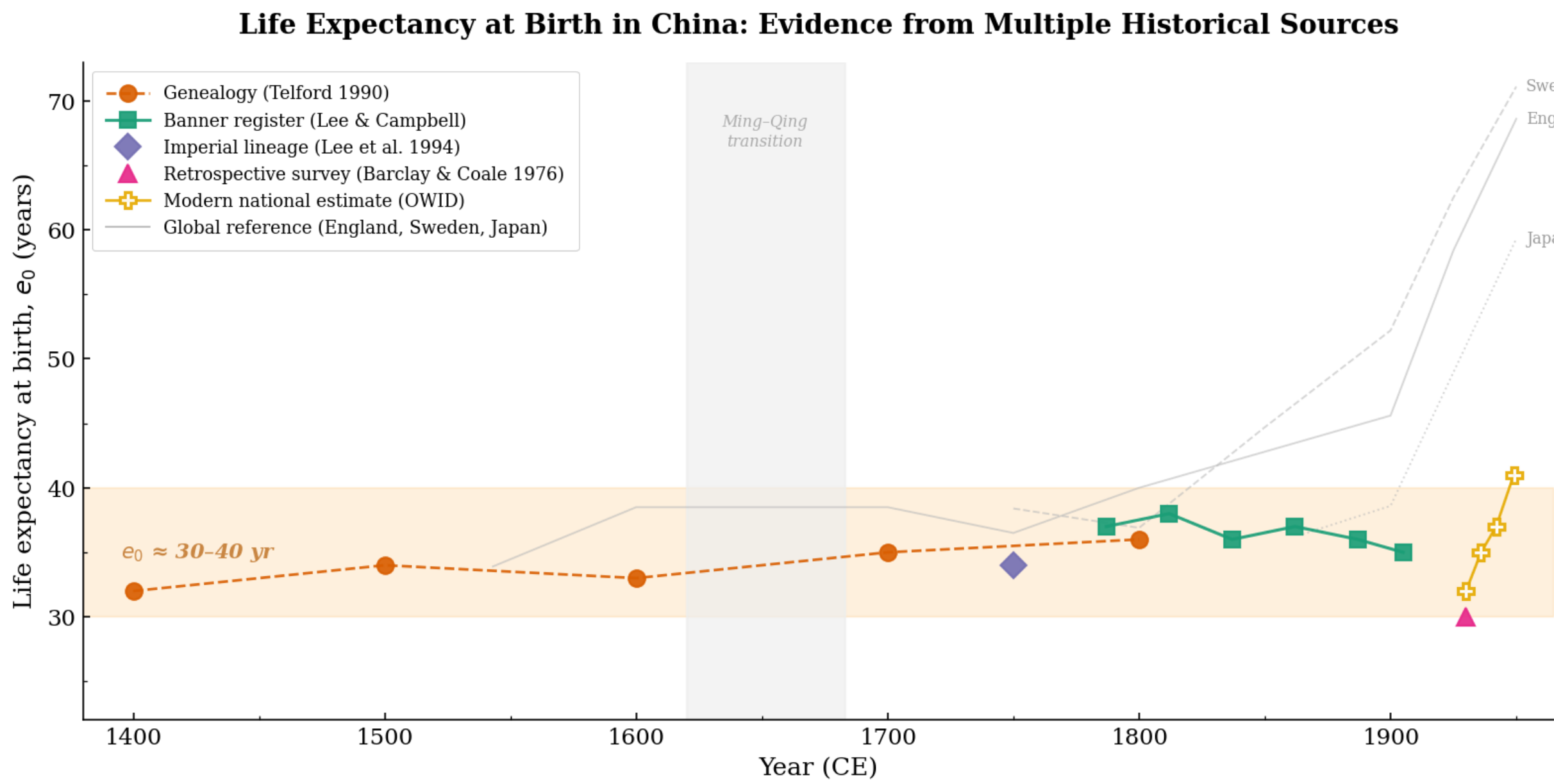

Figure S6: Life expectancy at birth (e_0) in historical China compiled from multiple independent historical sources (genealogies, banner registers, imperial-lineage and retrospective-survey estimates), with pre-industrial reference series for England, Sweden, and Japan shown in grey. Chinese estimates remain within $e_0 \approx 30-40$ years (shaded band) across 1400−1900 CE, consistent with the 20−40-year generational window recovered from the archives and with the ABM's `max_age` = 40 years.

- We reviewed existing results discussing past human life expectancy. In the **supplementary information**, we include a graph to demonstrate that our chosen parameter value, `max_age = 40`, is reasonable and that the expected lifespan it implies makes the optimal window we observe interpretable. Regarding this, we also corrected the inconsistency and apologise for the previous typo of 60 in the main text.

- Lastly, as we note above, reproducing exact changes in population structure was not our aim. In this paper, we focus more on historical documents as representations of collective memory, and on the fact that those who produce such collective memory do not represent the entire population. In fact, we conjecture (without finding direct evidence) that the time distribution of literate elites capable of writing would indeed be close to flat when the total population does not change much. For example, this is because the number of positions available at court to record history did not vary greatly (for compiling official histories), nor did the number of people in a given region increase (for compiling local gazetteers and diaries). We explicitly disclose this in the discussion of limitations:

Manuscript · Page 12, Line 265–269

Finally, the ABM is a deliberately minimal, heuristic model rather than a demographic reconstruction. This is mainly because the groups that produced the historical archives do not represent the entire population, and our global sensitivity analysis (**Figure 5d**) shows that their life expectancy matters. We have confirmed the key parameter in this simplified demography is consistent with population data we have collected (**Supplementary Information Figure S6**).

RC: *More generally, the connection between the ABM and the data feels ad hoc. From the SI it is clear that the model is conceptually grounded in cognitive psychology—negativity bias and anchoring, and in SBS, but in the main text the modelling section is framed almost exclusively in terms of SBS, without stating which psychological mechanisms are actually encoded. There is no attempt to calibrate the model to the empirical WDI series, nor any formal model-data comparison beyond the qualitative observation that both exhibit an optimal window of ~30 years, which is arguable*

given the log scale. Alternative mechanisms that could also generate a similar sliding-window optimum (e.g. purely statistical effects of autocorrelation or of the rolling standardisation itself or the average age of agents) are not explored. As a result, the current ABM functions more as an non-convincing toy model than as a genuine explanatory or testing framework; the link to the empirical pattern needs to be made more rigorous.

AR: Thank you to the reviewer for raising these concerns and these excellent points. We fully agree with your doubts about the expressed value and role of the ABM in previous versions. As you pointed out, we previously linked SBS to other cognitive psychology theories (e.g. negativity bias and anchoring), which stemmed from existing literature offering many cognition-rooted alternative explanations for the emergence of the SBS phenomenon[1,8,9]. After reorganising the research questions following your suggestion (see above responses), we refocused the research on SBS and reframed the ABM as a test of two competing hypotheses that could produce SBS. We also added a more comprehensive sensitivity analysis to strengthen the model's explanatory power. Specific revisions include:

1. Using the memory baseline as the lever for hypothesis testing. Under the exact same model, adopting a personal versus a collective memory baseline yields qualitatively different features: only the "H1: generational amnesia" produces the "correlated rise-then-fall" pattern, while the "H2 collective illusion" does not. The two mechanisms produce non-overlapping peak-window distributions, and only the H1 matches the empirical pattern.
2. The discussion showing two SBS-related conjectures does not conflict with deeper cognitive-psychological explanations, because they operate at different explanatory levels. For example, "negative bias" and "anchoring effects" operate at the individual level and explain why we form different baselines. In addition, when such baseline shifts occur, we show that SBS tends toward generational amnesia (H1) rather than collective illusion (H2) at the collective level.
3. Confirming via sensitivity analysis that the generational amnesia (H1) scenario, age dominates the results according to the Sobol analysis. Under the H2, instead, the window is more driven by climate_phi (ST ≈ 0.90). As for the max age of the agents under the H1 scenario, we also semi-quantitatively linked it to historical archives in the above revisions.
4. We no longer use a confusing logarithmic axis to show figures.

Accordingly, we now clearly position the ABM as a mechanism-discrimination test, a heuristic model, to detect which memory baseline is consistent with the empirical pattern, rather than a calibrated simulation. We hope the reviewer feels that this role and its connections are well reflected in the revised manuscript and the responses above.

RC: *The figures require substantial revision for clarity and scientific precision. Figure 1 combines three conceptually distinct elements (a study-area map, a conceptual SBS diagram, and a data-processing schematic—into a single figure). For readability it would be preferable to separate these or at least more clearly delineate them. In Figure 2a, the validation period (1901–2000 CE) appears to have larger fluctuations in the integrated proxy WDI than earlier periods; this noticeable change is not explained. The caption's phrase "the grey area indicates the uncertainties" is vague; the authors should specify whether this represents, for example, the 5–95% posterior credible interval or whatever else. Panel 2c includes a colour label that is not clearly identified in the caption (I assume it's the title of the plot). Panel 2d asserts that both WDI series "follow a normal distribution", but no normality test is reported; unless a formal test is performed and described, this should be phrased differently. Figure 3's legend seems unnecessary, panels c–f are not clearly labelled in the figure, the "validation period" shows up in panel f but I don't think it even belongs ther, and again the "Monte Carlo approach" is mentioned without any explanation. In Figure 4, plotting legends on top of error bars makes the legend hard to see. Figure 5's correlation-versus-window-size curves for the ABM are not interpretable without reading SI S3; the main text should explain how many runs were averaged, how events were discretized into levels, and how the curves relate to the observational analysis. At present, one effectively needs the SI to make sense of any of the modelling results, which is too heavy a dependence.*

AR: We thank the reviewer for these detailed figure comments and have revised the figures and captions accordingly. The figures have been reorganised, and every flagged panel has been corrected.

- For the **Figure 1**, we separated the three previously combined elements. The study area map is now a standalone figure in Supplementary Information (**Figure S1**). The conceptual SBS diagram and the data-processing schematic are consolidated into **Figure 1**, which now also includes the ABM design schematic.

- **Larger validation-period fluctuations (former Figure 2a).** We checked this, and what appears only in the 1901 − 2000 CE window is the overlaid instrumental validation series (the red lines). This is the well-known "variance attenuation of tree-ring reconstructions": calibration regression retains only the explained fraction of instrumental variance, so high-frequency weather variance is dampened in the proxy. We now explicitly mention this in the main text rather than leaving it unexplained:

Manuscript · Page 5, Line 108–110

The instrumental validation series (thin red line shown only for 1901–2000 CE) has a greater amplitude than the N-WDI, which reflects the variance attenuation inherent to tree-ring reconstructions (calibration regression damps high-frequency variance, retaining only the fraction explained).

- **"The grey area indicates the uncertainties" (former Figure 2a caption).** We now define the band explicitly as the 5 − 95% posterior credible interval of the Bayesian-integrated index (annual posterior mean ± 1.645 posterior SD). We also corrected the figure so that the band matches this definition:

Manuscript · Figure 2

The thin grey line is the annual N-WDI (posterior mean), the black line its 30-year low-pass reconstruction, and the grey band the 5 $\sim$ 95% posterior credible interval of the Bayesian-integrated N-WDI.

- **Panel 2c colour label.** This is the colour bar of the spatial correlation map. In the revised figure, it is a clean vertical colour bar labelled "Pearson r (Validation)" and the caption now iexplicitly identifies it
- **Panel 2d "follow a normal distribution".** We rephrased the sentence in the caption to:

Manuscript · Figure 2

Distributions of the N-WDI and H-WDI over the pre-instrumental period; both are approximately symmetric.

- **Mismatch figure (Figure 3): legend, panel labels, validation panel, Monte Carlo.** We removed the redundant legend and added explicit panel labels (c)–(f) to the figure. The Monte Carlo test is now fully defined (see our response to the earlier comment and the revised Methods and caption). We would note, however, that the existence of the panel "validation period" was correct. It was there to compare patterns that only appear in historical archives. We are sorry for the unclear annotation, which may have caused this confusion. We have now explicitly added this in the methods and the panel's caption:

Manuscript · Page 5, Line 144–146

Moreover, this shifted-correlation pattern is also absent between the reconstructed and the instrumental N-WDI during the validation period (from 1901 to 2000 CE) (**Figure 3f** and also **Supplementary Information Figure S11**), indicating that this is specific to the H-WDI.

Manuscript · Figure 3

f. No similar significant "shifted-correlation pattern" is detected for the instrumental data during the validation period (1901-2000 CE, $shift = 1$). More details about this null setup are available in **Supplementary Information Figure S11**.

- **Sliding-window figure (Figure 4): legend over error bars.** The legend has been repositioned to avoid overlap with the error bars.

- **ABM figure interpretability (Figure 5).** As detailed in our response to the previous comment, we redesigned this Figure. The caption now states how many replicates were averaged, how the model output is interpreted, and how the simulated curves relate to the observational analysis. We have aimed to make the modelling figure interpretable from the main text and **Methods** without the SI.

RC: *Finally, there are a few conceptual and numerical inconsistencies that should be resolved. In Section 2.3 the improvement in correlation after re-standardisation is described as "ranging from 0.1 to 0.2", while a few lines later the average optimal window size is said to yield a "maximum correlation increase of 0.12". From SI Table S6 it appears that 0.1–0.2 refers to the range across different proxy-integration and validation scenarios, and 0.12 is the maximum increase for the main pure-natural scenario averaged across temporal segments. This should be stated explicitly to avoid the impression of contradiction.*

AR: We thank the reviewer for raising this concern. The reviewer's understanding is entirely correct. We apologise for not having made this clearer in the manuscript before. We have now specified the increase in relevance for the main scenario in the text only as a percentage, and no longer referring to robustness checks of supplementary information as the range. We have made the following revisions to the wording:

> **Manuscript · Page 7, Line 152–158**
>
> Compared to the benchmark scenario without restandardisation (the whole-period Kendall's $\tau = 0.19$ between the unmodified N-WDI and H-WDI (before any re-standardisation), a greater correlation gradually emerges as the tested window size w increased to around 18 years, from 1470 to 1900 CE (maximum increased by 5.37%, **Figure 4b**). Across the different proxy-integration and validation scenarios we tested, the correlation improvements from restandardisation persist regardless of the method used to combine the composite datasets of N-WDI (see **Supplementary Information S2.2, Figure S5 and Table S6**)

Minor comments

RC: *For clarity throughout the manuscript, it would be helpful to introduce explicit labels for the two WDI series, such as O-WDI for the objective proxy-based index and S-WDI for the subjective archival index, and use these consistently in text and figures. This would make it much easier for readers to track which series is being transformed, validated, or used as a baseline.*

AR: We thank the reviewer for this practical suggestion and have adopted it, with one adjustment to the naming. Rather than O-WDI/S-WDI (objective/subjective), we introduce the neutral, descriptive pair H-WDI (historical-archive) and N-WDI (natural-proxy), which are used consistently throughout the text and figures. We made this choice so that the labels simultaneously resolve a concern raised by Reviewer #6 that an "objective vs subjective" framing is reductive, since the tree-ring series is itself an interpreted proxy.

RC: *In Table S2, the coordinates are given as raw numbers; the authors should add N/S/E/W designations (e.g. 35.18°N, 103.73°E) to conform to standard geographic notation. In SI 1.4, the statement that self-comparison materials "may account for more than 10%" is described as a "subjective estimation"; the basis for this figure is unclear. If it is from an actual tally on a defined corpus, the method should be described; if not, a precise percentage should be avoided and replaced by a qualitative statement. In Table S3, the 1690 CE translation includes "releasing over a hundred dragons". If this is here a metaphor for floodwaters or water spirits, this should be clarified; otherwise, the current phrasing may mislead readers.*

AR: Thank you to the reviewer for raising this point. We have made the formatting changes in the latest **Supplementary Information**, including modifying latitude and longitude formats, removing imprecise percentages, and deleting unnecessary metaphorical references.

RC: *In SI S3 section 1.2, memory is described as "implemented as a deque storing recent climate experiences"; for non-programmer readers, it would be clearer to say explicitly that this is a first-in-first-out (FIFO) queue of past climate values up to the agent's memory capacity.*

AR: We thank the reviewer for this point and have rephrased this for non-programmer readers. The **Supplementary Information S3** (ODD+D protocol) now describes the memory explicitly as a first-in–first-out (FIFO) queue rather than a "deque":

Supplementary Information · Page 20, Line 299–301

a bounded personal memory implemented as a "first-in–first-out (FIFO)" queue storing recent climate experiences up to the current age, a minimum age for recording eligibility (default 10 years), and a maximum lifespan (default 40 years).

RC: *The last sentence before SI Section 2 ("using a standard-deviation filter applied to the right-hand series") is opaque; "right-hand series" should be replaced with a clear description such as "applied to the climate time series used as the objective reference" or similar. Likewise, the phrase that parameters "come from experience" in section 2.1 should be clarified: presumably the authors mean that parameter values are chosen based on expert judgment and not fitted to the data.*

AR: We agree with both points and have revised the wording of the **Supplementary Information**.

- We replaced the opaque "right-hand series" with an explicit description of which series the filter is applied to:

Supplementary Information · Page 21, Line 328–330

After completion, the model computes rolling-window correlations between the collective series and the underlying climate using a standard-deviation filter applied to the natural climate series used as the objective reference.

- We clarified that the parameters are set by expert judgment rather than fitted:

Supplementary Information · Page 23, Line 371–373

This empirical phenomenon inspires the model, but no parameters are derived from data analysis. Therefore, conceptually, this is a heuristic model intended to analyse potential mechanisms of SBS.

RC: *In SI section 2.4 the authors suddenly mention three possible baselines (personal memory, full model climate series, and collective archive) whereas earlier sections describe only two operational settings (personal vs collective). It should be clearly stated which baselines are actually used in the analyses and which are only conceptual possibilities. In 2.6 they write that "in its current configuration, the archive primarily serves as a tool for analysis and does not influence individual decisions", which appears to conflict with earlier references to agents comparing to collective records; this ambiguity should be resolved. In 2.7, the last sentence about "prospective extensions with re-judgment" is difficult to understand and could be rephrased.*

AR: Sorry for the confusion. We have corrected these three points accordingly:

- In the main text, we used only two baseline scenarios to avoid confusing readers. But in robustness testing, we also tested more. We have only detailed the results of the other experiments now in the **Supplementary Information (Robustness Analyses)**.
- We removed the confusing statement "in its current configuration, ⋯" and listed the interactions between agents and the archive by scenario.
- We rewrote the previously hard-to-understand sentence in **Supplementary Information Section 2.7** like this:

Supplementary Information · Page 23, Line 367–371

Only the diagonal H1/H2 contrast is foregrounded in the main text; `collective_lifetime` and `model` are reported here as decomposition/benchmark controls, and their full correlation profiles are presented with the robustness analyses below (Figure S8). The empirical basis for the model is derived from analyses of historical climate records from northern China (1470–1900 CE), tree-ring reconstructions (validated for 1901–2000 CE), and the psychological literature on cognitive biases in environmental perception (see main texts).

RC: *The rationale for representing ordinal severity levels via truncated-normal sampling to produce continuous values should be explained. Since the empirical analyses use ordinal categories and Kendall's τ, it is not obvious why a continuous mapping is needed for the ABM outputs; at least, the authors should argue that this choice does not materially affect the correlation patterns. In 2.9, record transmission loss is said to occur with probability loss_rate "(default 0.20, uniform distribution)"; this is not a uniform distribution but a Bernoulli process with parameter 0.2, and the terminology should be corrected. The choice of maximum age 40 in the default configuration also needs a brief justification in light of the discussion of generational life expectancy.*

AR: We thank the reviewer and have addressed all three points.

- **Rationale for the truncated-normal mapping.** In any given year, several observers (agents in the ABM) or several archival sites (in the empirical data) may record different ordinal levels, and averaging raw integer codes into a single yearly/regional value has no natural metric. We therefore map each level to a continuous value drawn within its z-score interval and average. This is the same procedure used for the empirical H-WDI, so the ABM output and the archives are processed identically rather than by using two different conventions. The downstream statistic is Kendall's τ, a rank correlation, so the mapping affects only the absolute magnitudes assigned to levels and the breaking of ties among equal level-averages —not the cross-year rank order that, together with the sliding re-standardisation window, produces the window-dependence:

Manuscript · Page 15, Line 363–375

Generally, concrete determinations of floods or droughts, along with descriptions of severe societal outcomes of disasters, are classified as "Severe" level (SD or SW), while alone records of natural conditions (such as heavy rains or prolonged droughts) are classified as "Moderate" levels (MD or MW). In contrast, a normal (N) year refers to keywords "harvest", "abundant year", "ripe", and "bountiful", or non-abnormal events in the current year, with continuous records available in the preceding or following years (**Supplementary Information Table S3** provides more examples). To integrate archives across the study area, site-year ordinal grades were first converted to continuous z-scores, because ordinal levels cannot be averaged meaningfully. Each grade maps to a z-interval bounded by the same thresholds used for WDI classification (rather than a single point, see **Table 1**). It means, for the site i and year t, we drew 100 values from a truncated normal within that interval and used their mean as the site-level continuous WDI, $h_{i,t}$. The regional H-WDI was then $H_t = \text{mean}(\{h_{i,t}\})$ over all sites with non-missing records in the year t ($n_t \leq 30$). Sites without archival evidence were excluded rather than imputed, and years with no reporting sites were left missing. This stochastic mapping is a tie-breaking convenience for cross-site averaging.

Numerical confirmation: we reran the entire window−τ analysis, replacing stochastic sampling with a deterministic midpoint mapping ({−1.5, −0.5, 0, 0.5, 1.5}). The peak (optimal) re-standardisation window is **identical** under the two schemes, the profiles correlate at r ≥ 0.99, and |Δτ| ≤ 0.03 at every window, for both the empirical H-WDI/N-WDI comparison and the ABM collective archive. The two curves differ only by a small, near-constant vertical offset (≤ 0.03), which is expected and immaterial: the deterministic midpoints and the truncated-normal interval means assigning slightly different magnitudes to each level (e.g. ±1.66 vs ±1.50 for the extreme level). When the continuous series is re-classified against fixed thresholds (the empirical pipeline), sharper sampling magnitudes yield marginally higher τ, whereas in the direct continuous ABM comparison, the single-draw sampling noise marginally attenuates τ. In neither case does the offset shift the location of the correlation peak—the quantity that carries our conclusion (a generational, ~20–40-year optimum).

- **"uniform distribution" → Bernoulli.** Corrected: record transmission loss is a Bernoulli process, not a

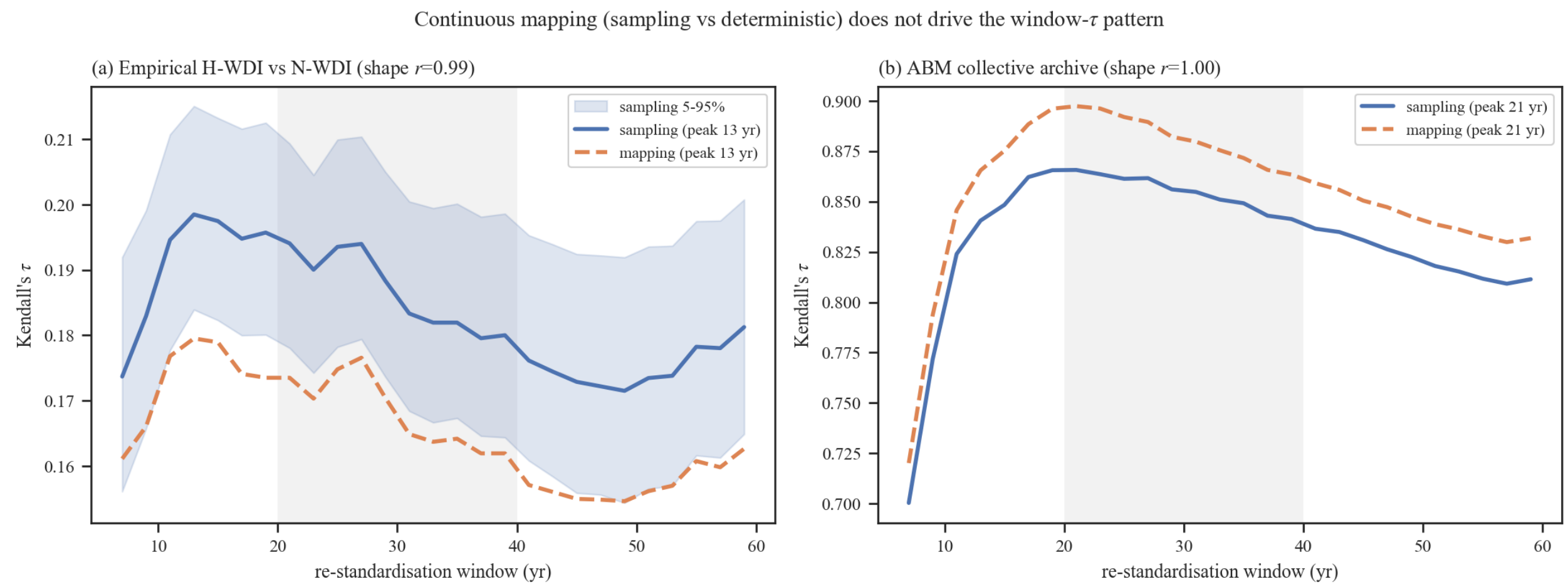

Figure R2: Numerically confirm that different sampling methods do not affect the pattern of the results. Left: sampling methods' impacts on empirical optimal sliding window size; right: impacts on the ABM's results.

uniform distribution. Additionally, this parameter actually takes the value 0.4 in the ABM (the previous 0.2 was a typo). Therefore, the SI now reads:

Supplementary Information · Page 23, Line 378–381

> Agents make decisions through a two-stage process: first, computing a z-score indicating how the current climate deviates from their personal memory; then, drawing a Bernoulli trial with probability determined by a survival function that assigns higher likelihoods to extreme deviations.

- **Justification for `max_age = 40`.** We added a brief justification tying it to generational life expectancy and noted that it is not load-bearing, with a data-based figure earlier to illustrate that this parameter is reasonable:

Supplementary Information · Page 20, Line 314–319

> The default `max_age = 40` approximates the adult recording lifespan of an observer in this historical context (roughly the working/adult span of a generation) and is consistent both with the life expectancy at birth estimated for historical China[10] ($e_0 \approx 30 - 40$ years; based on multiple independent sources[11–16], including Genealogy, Banner register, Imperical lineage, Retrospective survey, and global references for pre-industrial values in England, Sweden, and Japan (Figure S6)). Because the sensitivity analysis varies `max_age` over 15–80 years, the emergent optimal window scales with it.

RC: *In compare.py the correlation defaults likely underconstrain rolling windows, since `min(log2(n),2)` is almost always <=2.*

AR: The reviewer is right about this incorrect default value. We corrected the default from `min(log2(n), 2)` to `max(int(log2(n)), 2)` and added regression tests. The manuscript's **Figure 4c** sweep already passes an explicit `min_periods`, so its main optimum result is unchanged. The fix mainly corrects the library default for analyses that rely on `compare_corr` without an explicit `min_periods`.

RC: *It seems like the confusion matrix orientation is not consistent, since you transpose, then the index and column names seem flipped, but I may have missed something here.*

AR: The reviewer is right about the labels. The transpose is deliberate (so the matrix matches the downstream plotting and mismatch-analysis access pattern, rows = predicted/H, columns = true/N), but the `index.name`/`columns.name` metadata had been set the wrong way around. We corrected the axis names to `pred`/`true` (in both `_compute_confusion_matrix`

and the `false_count_matrix` property) and added a docstring explaining the transpose. The matrix **values** are byte-identical, and only the (previously swapped) axis-name metadata changed.

RC: *The reindexing of the matrix in calibration.py is happening at the level of the local variable (matrix) not applied to the matrix. So, in the end nothing is happening. Unless you are using a different version when this behavior is not the same. (Remarks on code availability)*

AR: Thanks for raising the concern. Reassigning the loop variable did not rebind the underlying `DataFrames`, so the reindex was confusing dead code. We fixed it to explicitly rebind both matrices. The dormant bug had no numerical effect because pandas' label-aligned arithmetic in the subsequent z-score step already padded to the full category axes. We verified that the resulting p-value matrix is identical before and after the fix.

RC: *I skimmed the code briefly and there seems to be at least one key problem. See report. (Remarks on code availability)*

AR: We thank the reviewer for the code-level scrutiny. The key problem flagged in the report is the ABM z-score operator-precedence bug, which we have fixed (see the dedicated response above, with a comparable figure). We have also resolved every other code issue raised and appreciate your careful review!

3. Reviewer 3

RC: *This paper was a pleasure to read. In the abstract, the paper's purpose is clearly stated and articulated in three steps: - A mismatch is shown between historical and instrumental observations; - The mismatch is mitigated by generation-sized sliding windows; - An ABM model reproduces this periodicity.*

AR: We sincerely thank the reviewer for the kind words and for the clear, accurate summary of our three-step argument. We are delighted that the paper's structure came across as intended.

RC: *The paper, in the view of these reviewers, by any means deserves publication. There are, however, some improvements that we think could improve its readability.*

AR: We are very grateful for this positive overall assessment and for the constructive, readability-focused suggestions. We have worked through each point below and revised the manuscript accordingly to improve clarity and flow.

RC: *From a theoretical standpoint, the paper appears conceptually robust. The Shifting Baseline Syndrome framework is highly compelling, and the attempt to reproduce its underlying mechanisms through an ABM is both coherent and methodologically promising. Furthermore, this approach seems potentially transferable to other domains (e.g., the perception of crime or urban disorder, where what is considered "normal" may also shift across generations). This represents a significant strength of the paper. The authors could give their opinion on transferability, and potentially add other examples to this regard.*

AR: We thank the reviewer for this generous assessment and for the excellent suggestion. We agree the mechanism transfers wherever the baseline against which "normal" is judged is itself reconstructed each generation, and we have made this explicit in the Discussion, adding the reviewer's own cross-domain example:

> **Manuscript · Page 13, Line 271–275**
>
> The implications of our findings extend beyond historical climatology. We demonstrate that perceptual biases can operate consistently across long timescales[17], suggesting that cognitive mechanisms of SBS identified in contemporary contexts[18] have deep historical roots and potentially broad applicability. Evidence of SBS and generational amnesia has been discussed across diverse fields, including public perceptions of urban social incidents[19], settlement in flood-prone areas[20], and assessments of the historical condition of degraded ecosystems[4].

RC: *It may also be worthwhile to further problematize the nature of the historical archives. These sources may not simply reflect aggregated individual perceptions, but could also be understood as institutional products shaped by administrative conventions and relatively stable classificatory criteria. Clarifying whether possible effects of administrative practices or evolving classification standards over time have been considered would strengthen the argument. Moreover, this issue connects to the 30 year threshold, within which the correlation between historical records and climatic proxy data appears to improve. While this window size produces the strongest statistical alignment, it does not necessarily imply that individuals retain a generational memory of exactly thirty years.*

AR: This is a valuable point, and we agree on both counts: (i) the archives are partly institutional products shaped by administrative conventions and classification standards, not purely aggregated individual perceptions, and (ii) the ~30-year window marks the scale of strongest statistical alignment and should not be read as a literal "exactly thirty years" of individual memory. We have added the discussion in the corresponding part of the main text:

- We first acknowledge that institutions are also important factors in shaping historical archives in our discussion of research limitations.

> **Manuscript · Page 12, Line 243–244**
>
> Several limitations warrant consideration. First, historical archives, shaped by institutions, also incorporate social responses that modulate the societal impact in addition to individual perceptions[21].

- Then, the fact of consistently improved correlation between historical and natural datasets across time indicates that different institutions don't affect the robustness of our analysis:

Manuscript · Page 12, Line 249–255

Moreover, our consideration of temporal slices addresses issues of changing political context, with similar results across two major Chinese dynasties (**Supplementary Information S1 and Figure S2**), suggesting the robustness of our analysis under different institutions (**Figure 4c**). For example, around the time of the Emperor Wanli (万历, 1573-1620 CE), a period of prosperity turned to decline for the Ming Dynasty and far fewer fiscal resources were allocated to disaster relief than from 1649 to 1849 CE, when the emperors in the Qing Dynasty established a complex disaster response system. Nevertheless, this difference did not lead to a deviation from the unimodal patterns we identified.

- To explain that the optimal window is about 30 years, we state more directly in the discussion that this is not fixed but is directly linked to population life expectancy.

Manuscript · Page 11, Line 220–227

Our optimal sliding window sizes (ranging from 20 to 40 years) are also approximately equal to the generational life expectancy of humans in ancient North China and worldwide[10] (see **Supplementary Information Figure S6**). This pattern and its peak in correlation improvements can be reproduced by a heuristic ABM under the assumption of "generational amnesia", but not under the assumption of "collective memory illusion" (**Figure 5**). This implies that the emergence of SBS can be attributed to biased perceptions shaped by members of the same generation based on their experience, not from the illusion of shared narratives. Further sensitivity analyses also show that the agents' life expectancy is the dominant parameter in this scenario, which leads to the empirical pattern (**Supplementary Information Figure S7**).

- We also added a comparative chart of population life expectancy in the supplementary information, along with newly added sensitivity experiments that have strengthened this association and support the generational amnesia hypothesis.

RC: *Yet, accuracy of data and coherence of statements should have been more carefully checked; it's really unsettling to see Kendall's tau jump between 0.17 and 0.19 in different parts of the text.*

AR: We apologise for the apparent inconsistency. We audited every reported Kendall's τ and confirmed that they constitute a single, internally consistent set. The "jumps" came from quoting three different comparisons without labelling them. We have now labelled each occurrence explicitly in the main text: $\boldsymbol{\tau}$ **= 0.176** is the agreement between the H-WDI and N-WDI **categories** over the pre-instrumental period (**Figure 3a**); $\boldsymbol{\tau}$ **= 0.177** is the agreement between the N-WDI and the independent instrumental series over the validation period (Figure 2a); and $\boldsymbol{\tau}$ **= 0.190** is the whole-period benchmark between the unmodified N-WDI (anomaly rather than categories) and H-WDI, before any re-standardisation. The first two are near-identical, which is exactly the "comparable agreement" point we make in Section 2.2; the third differs because it is a distinct whole-period, non-re-standardised benchmark. With these labels, the three values no longer read as a single quantity that "jumps". We have now retained three decimal places, which makes the differences clearer. For reference, the three values now appear in the text as:

Manuscript · Page 5, Line 124–130

During the pre-instrumental period (1470 to 1900 CE), the categorised H-WDI shows modest but significant agreement with the categorised N-WDI (Cohen's weighted $\kappa = 0.2$, classification accuracy = 0.39), with a slight but significant positive rank correlation between the two categorical series (Kendall's $\tau = 0.176$ for H-WDI v.s. N-WDI, $p < 0.05$; n = 208; **Figure 3a**). This level of agreement is comparable to that achieved by the proxy itself against independent instruments over the validation period (1901 – 2000 CE), where the N-WDI

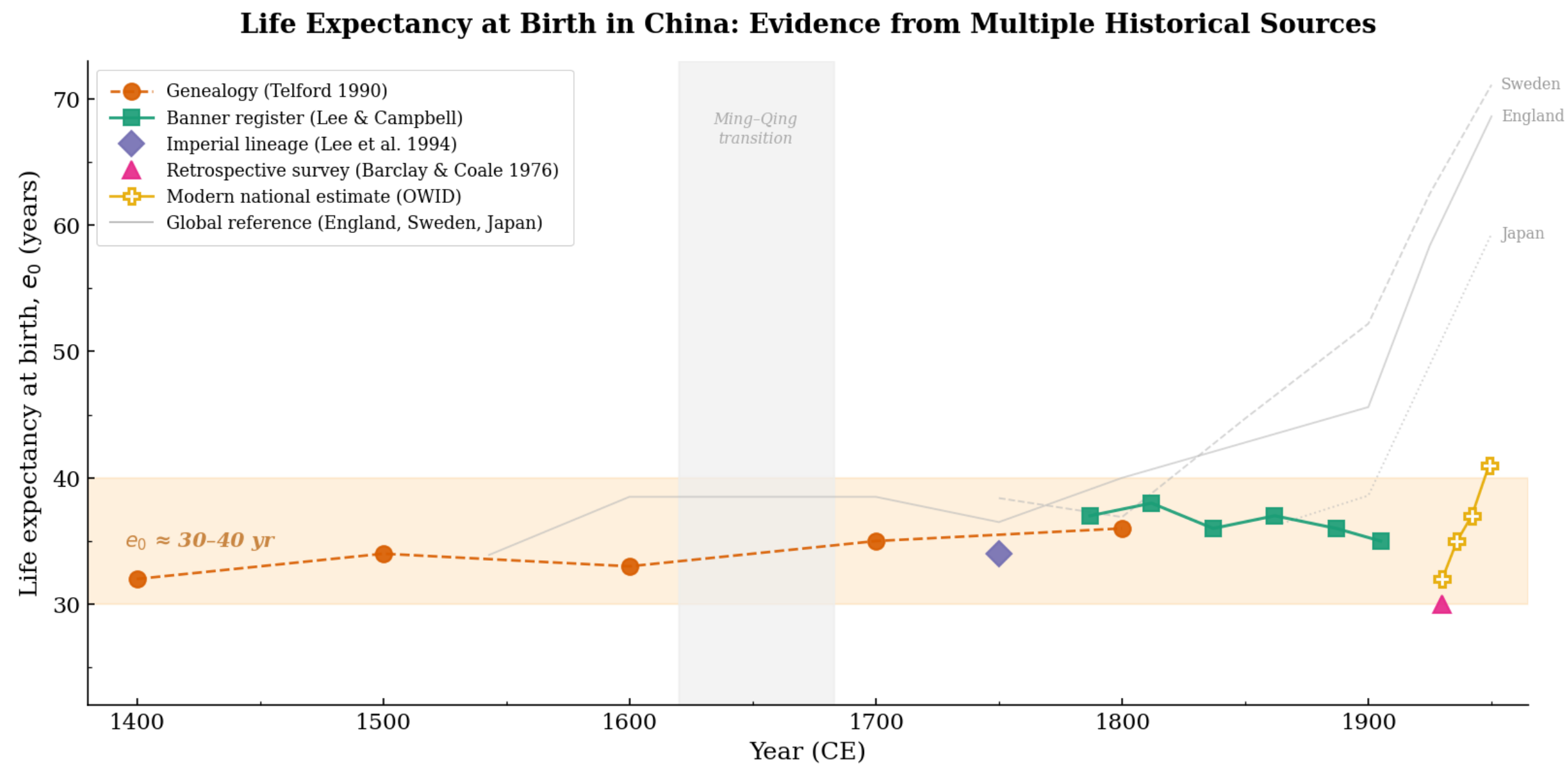

Figure S6: Life expectancy at birth (e_0) in historical China compiled from multiple independent historical sources (genealogies, banner registers, imperial-lineage and retrospective-survey estimates), with pre-industrial reference series for England, Sweden, and Japan shown in grey. Chinese estimates remain within $e_0 \approx 30-40$ years (shaded band) across 1400−1900 CE, consistent with the 20−40-year generational window recovered from the archives and with the ABM's `max_age` = 40 years.

and the instrumental series agree at Kendall's $\tau = 0.177$ (Cohen's weighted $\kappa = 0.2$, $p < 0.05$ n = 113, see **Supplementary Information Figure S11**)

Manuscript · Page 7, Line 152–158

Compared to the benchmark scenario without restandardisation (the whole-period Kendall's $\tau = 0.19$ between the unmodified N-WDI and H-WDI (before any re-standardisation), a greater correlation gradually emerges as the tested window size w increased to around 18 years, from 1470 to 1900 CE (maximum increased by 5.37%, **Figure 4b**). Across the different proxy-integration and validation scenarios we tested, the correlation improvements from restandardisation persist regardless of the method used to combine the composite datasets of N-WDI (see **Supplementary Information S2.2, Figure S5 and Table S6**)

RC: *The ABM is presented as a "simple but robust ABM approach" and we agree to that. This usage of ABM sets an example of clever application of the methodology. Having already shown that a generational sliding window improves correlation, the authors use the ABM to reinforce the result and propose an explicit causal mechanism that puts their thesis to work.*

AR: We thank the reviewer warmly for this endorsement of our modelling strategy. We have further strengthened the ABM in revision (a climate-forcing robustness matrix and a two-stage global sensitivity analysis), reinforcing exactly the "simple but robust" character the reviewer highlights. As we stated in our response to Reviewer 2 regarding the "disconnect" between the ABM and the empirical analysis, we explicitly position it as a "heuristic model" for hypothesis testing.

Manuscript · Page 10, Line 187–189

This heuristic model suggests that generational amnesia (assuming individuals rely on their lived experience) can cause generational inconsistencies, whereas collective illusion is insufficient to reproduce the observed mismatch pattern in the empirical WDIs.

RC: *While the statement that the paper is, "as an essential step in addressing the challenges of the Anthropocene," might sound, and be, slightly exaggerated, we agree that the paper constitutes an exemplary way of "combining empirical data from archives and paleo-environmental proxies with computational modelling". This sets an example of good practice for the field, that we hope will find diffused application. In the words of the authors, "co-evolutionary models being developed to study human-Earth system interaction should find a way to incorporate systematic data on the role of collective actions in adaptations and extremes."*

AR: We take the point and have toned down the "essential step in addressing the challenges of the Anthropocene" phrasing to a more measured claim, while retaining the framing the reviewer endorses (combining archival/paleo-environmental data with computational modelling). We emphasise more on our framework's transferability:

> **Manuscript · Page 13, Line 289–292**
>
> Moreover, our methodology provides a novel and transferable framework for studying long-term collective memory dynamics by combining empirical data from archives and paleo-environmental proxies with computational modelling. It demonstrates the impact of slow variables (such as collective memory) on co-evolution between humans and the environment during the Anthropocene[22].

RC: *The ABM code is available, and documented in ODD, as all ABMs should. Development of the ABM has been made with the use of a platform (https://pypi.org/project/abses/) that remarkably improves replicability.*

AR: We thank the reviewer for recognising the ODD documentation and the use of the `abses` package[23], and for valuing reproducibility as we do. In the new version, we further strengthened the description of the ABM, including sensitivity analyses and a more detailed presentation of the methodological foundations. The code for the new version is also openly available.

RC: *In the following, we remark in a more punctual way on the state of the paper.*

AR: We address each of the following specific remarks in turn below.

2 Results

RC: *The validation period is not mentioned in the introduction and appears quite abruptly, thus making it a surprise for the reader, disrupting the flow.*

AR: Agreed. Thanks for your valuable suggestion. We include this important first step in the last paragraph of the introduction now:

> **Manuscript · Page 3, Line 88–98**
>
> First, to verify the reliability of tree-ring data as an independent, non-perceptual reference, we independently validated the reconstructed N-WDI against instrumental data for the period 1901−2000 CE. Then, to explore the existence and nature of this phenomenon in the past, we performed a statistical comparison of the WDIs calculated from our two different, independent datasets, determining their matches/mismatches status and correlation (**Figure 1c**). If SBS existed, mismatched records are expected to exhibit a systematic over- or underestimation pattern (static), which can be addressed by restandardization within sliding windows —i.e., correcting for generational differences (dynamic). Finally, to interpret the mechanism, we examine two hypotheses (generational amnesia or collective memory illusion) and further develop a heuristic agent-based model (ABM) to explain the identified SBS, testing whether it reproduces the same patterns (**Figure 1d**). From data to model, our approach aims to systematically and quantitatively reveal the long-term role of collective memory in climatic recording, experience, and, thus, adaptation.

RC: *In this section, the p-tests don't seem to have been preregistered, and in the opinion of this reviewer p<0.1 should not be mentioned.*

AR: Agreed. The analyses were not preregistered, and we have removed every reference to the $p < 0.1$ threshold, reporting significance only at conventional levels (now * = $p < 0.05$, ** = $p < 0.01$). So, Figure 3 and every other significance-

annotated panel re-render without the p < 0.1 tier; the Methods Monte-Carlo passage and the **Figure 3** caption were updated to match as well.

RC: *In section 2.2, if we understand it, tau is 1.18 for the data, 1.17 for validation. In section 2.3, tau is 1.19, same as in the relative figure. Are these different calculations, and why?*

AR: Sorry for the confusion we caused. We have now distinguished the values explicitly in the text. In 2.2, the two τ are different comparisons: $\boldsymbol{\tau}$ = **0.176** is H-WDI vs N-WDI levels (the archive-vs-proxy agreement, pre-instrumental) and $\boldsymbol{\tau}$ = **0.177** is N-WDI vs the instrumental series (the proxy validation, 1901–2000). In §2.3, $\boldsymbol{\tau}$ = **0.190** is the whole-period benchmark between the unmodified N-WDI and H-WDI *before* re-standardisation (the value also shown as the baseline in Figure 4b); Each is now labelled correctly where it appears.

Methods

RC: *Apart from some typo here and there, the style of presentation can be improved. The reader gets absolutely nothing from the proxy equation. As a rule of thumb, no equations should be in a paper that don't get used later on. Same goes for the C(x) equation that can be better summarized in a table. For Cohen, it appears that, when you state that you have used the quadratic one, you give (and never use again) the linear one.*

AR: We thank the reviewer and have adopted these suggestions.

- **C(x) ⟶ table.** The piecewise $C(x)$ definition is now a table (category, symbol, standardised range), which is easier to read. Combining with a suggestion from another reviewer, we also include an archival example to differentiate the levels of severity (Table 1 in the revised manuscript):

Table 1: Five severity levels of the Wet/Dry Index (WDI) and representative archival examples.

Level	Abb.	Z-score	Archive example	Translation
Severe dry	SD	$\leq -1.17\sigma$	旱荒，发赈账济，全活甚众。	Due to the drought and famine, relief funds were distributed widely, saving many lives.
Moderate dry	MD	$[-1.17\sigma, -0.33\sigma]$	夏大旱，至八月始雨，禾尽槁	In the summer, there was a great drought; it did not rain until August, and all the grain withered.
Normal	N	$[-0.33\sigma, 0.33\sigma]$	大稔	Great harvest.
Moderate wet	MW	$[0.33\sigma, 1.17\sigma]$	九月葵未，诏顺天、保定、河间、永平四府水涝伤稼	In September, accumulated water soaked the grain. Yongding, Baoding, and Hejian prefectures were flooded.
Severe wet	SW	$\geq 1.17\sigma$	夏，汉江水涨，漂没庐舍甚众	In summer, the Han River flooded, sweeping away many households.

- **Cohen's κ.** We confirmed there is no quadratic/linear inconsistency in the revised text: the Methods state Cohen's *quadratic-weighted* κ and describe a penalty proportional to the squared category distance and no unused linear form remains.

Manuscript · Page 17, Line 412–415

Statistical validation of classification agreement was done employing Cohen's weighted Kappa coefficient[24] with quadratic weighting ($\kappa_w = \frac{p_o - p_e}{1 - p_e}$), where, p_o represents observed agreement and p_e denotes expected agreement under random classification

- **The proxy/integration equation.** We removed the displayed equation. The Bayesian integration described is retained and is now explained in plain language, including why a simple per-year average is not appropriate (see our response to Reviewer #5 on the integration).

RC: *What is with the values in the C definition? The 10% threshold of the normal is at -1.28, not 1.17.*

AR: The reviewer is right that -1.28σ is the exact 10% quantile of the standard normal. Our thresholds are not theoretical normal quantiles, and we have clarified this in the Methods. The five-grade scheme follows the WDI convention of the source atlas[25]: the grades are defined to reach target frequencies of about 10% for the most severe dry and wet grades, and the thresholds were set empirically from the observed frequency distribution of summer precipitation, which is only approximately Gaussian. The value $\pm 1.17\sigma$ is therefore the empirical boundary that reproduces the preset frequency of about 10%, and it does not contradict the theoretical $\mp 1.28\sigma$, since the two answer different questions. The realized cumulative probabilities at our thresholds are {0.121, 0.371, 0.629, 0.879}, giving frequencies of about 12/25/26/25/12%. We keep the thresholds and the nominal percentages unchanged, and no results change.

> **Manuscript**
>
> ⋯the standardized thresholds $[-1.17\sigma, -0.33\sigma, 0.33\sigma, 1.17\sigma]$ are empirical boundaries rather than theoretical normal quantiles: the Atlas compilers derived them from the observed frequency distribution of summer (May–September) precipitation so that the resulting grade frequencies match the preset targets (about 10% for the most severe dry and wet grades). Because precipitation is approximately but not exactly Gaussian, this empirical $\pm 1.17\sigma$ differs slightly from the theoretical $\pm 1.28\sigma$ that would bound the exact 10% normal quantile.

RC: *Small typos, missing verbs and other minor comments can be found in the attached PDF. They must be understood just as suggestions, or pointers to places where small improvement can be made. (Remarks on code availability)*

AR: We are grateful for the detailed annotations in the attached PDF and have worked through them.

RC: *The code looks reproducible and usable.*

AR: We thank the reviewer for this confirmation. We have additionally added tests alongside the four code fixes made in response to the other reviewers, further safeguarding reproducibility.

4. Reviewer 4 and 5

RC: *I co-reviewed this manuscript with one of the reviewers who provided the listed reports. This is part of the Nature Communications initiative to facilitate training in peer review and to provide appropriate recognition for Early Career Researchers who co-review manuscripts. (Remarks on code availability)*

AR: We thank the co-reviewer for their time and contribution, and we welcome Nature Communications' initiative to recognise the work of Early Career Researchers in peer review. No specific change is requested in this note.

RC: *This article is quite fascinating. As someone who works with anecdotal evidence of effects of extremes on ecosystems I have long wondered about shifting baseline syndrome, and was excited to see this empirical test. It seems to me very novel and clever in the way it brings together historical records with climate proxies.*

AR: We are delighted that the reviewer, with first-hand experience of shifting baselines in ecosystems, found the empirical test novel and compelling. We thank them warmly for this encouraging assessment!

RC: *However, at present the work is extremely difficult to follow. Both methods and results are quite opaque. It would be good to make the work more broadly accessible with much clearer explanations and figures. Most of my suggestions below are aimed at improving the clarity of the presentation.*

AR: We take this comment seriously and have prioritised clarity in the revision. We have (1) introduced consistent notation for the two WDI series (N-WDI v.s. H-WDI); (2) expanded all figure captions with more details of the method; (3) moved key derivations and archival examples into a clearer form; (4) and rewritten the most opaque methods (especially the mismatch analysis) and corresponding results. The point-by-point responses below detail each change.

RC: *Given that the detailed methods are at the end of the paper, the introduction and results sections need to give some more information about the approach taken.*

AR: Agreed. This has been addressed in the revision.

- The final paragraph of the Introduction now lays out the three-step approach before the detailed (end-placed) Methods:

Manuscript · Page 3, Line 88–98

First, to verify the reliability of tree-ring data as an independent, non-perceptual reference, we independently validated the reconstructed N-WDI against instrumental data for the period 1901−2000 CE. Then, to explore the existence and nature of this phenomenon in the past, we performed a statistical comparison of the WDIs calculated from our two different, independent datasets, determining their matches/mismatches status and correlation (**Figure 1c**). If SBS existed, mismatched records are expected to exhibit a systematic over- or underestimation pattern (static), which can be addressed by restandardization within sliding windows —i.e., correcting for generational differences (dynamic). Finally, to interpret the mechanism, we examine two hypotheses (generational amnesia or collective memory illusion) and further develop a heuristic agent-based model (ABM) to explain the identified SBS, testing whether it reproduces the same patterns (**Figure 1d**). From data to model, our approach aims to systematically and quantitatively reveal the long-term role of collective memory in climatic recording, experience, and, thus, adaptation.

- The Results are made self-contained through the substantially expanded figure captions and a compact introduction paragraph for the ABM's architecture, so a reader can follow the logic without first turning to the Methods. For two examples:

- (1) How is the " shifted-correlation pattern" detected:

Manuscript · Page 5, Line 133–137

To further explore the "comparing to the past" process implied by the SBS theory, we compared each mismatched H-WDI data with a similar prior event which occurred once, twice, or three times ($shift = 1, 2, 3$, respectively) in the past (**Figure 3b**). In these mismatch cases, the H-WDI is usually categorised as drier (or wetter) than N-WDI when the z-score values of N-WDI are only slightly lower (or higher) compared to a former year.

- (2) A brief ABM architecture overview in the results:

Manuscript · Page 7, Line 168–176

The heuristic ABM represents climate as a stationary AR(1) stochastic process ($c_t = \phi c_{t-1} + \varepsilon_t$, **Figure 5a**; alternative independent and trended forcings are tested for robustness in **Supplementary Information S3 and Figure S9**) observed by a continuously turning-over population of finite-lifespan agents. Each agent judges the current anomaly against a baseline, and is more likely to log an event the more it departs from that baseline (**Figure 5c**). We designed scenarios to represent the "baseline", based on lived experience or shared archives of agents, corresponding to the two hypotheses of "H1 generational amnesia and H2 collective illusion" (**Figure 5c**), with two other scenarios tested for robustness (see **Supplementary Information S3, Table S10 and Figure S8**). The archive contains logged severities from all agents and is aggregated and classified into the same five-level WDI series, so the simulated and empirical records are compared identically (see **Methods**).

RC: *Figure 1b-c is intended to illustrate the conceptual idea and approach, but is not easy to follow. An extended caption to walk the reader through the diagram would help. In Figure 1b, I think the person on the left is seeing the grey dots as normal and the person on the right is seeing their grey dot as a drought? It's not clear what the green and orange dots are, or why there are now different green and orange dots in Figure 1c. Perhaps rethink Figure 1c to more clearly show how restandardisation works.*

AR: We have redrawn **Figure 1** and now walk the reader through panels a–d step by step via the legend and an expanded caption. The meaning of the dots is stated explicitly in the legend, and how "shifting baseline syndrome" and "restandardisation" work have been redesigned and shown in panels **a.** and **c.** more clearly.

RC: *Lines 103-104: r = 0.37 seems like a relatively low coefficient for the comparison between the instrumental record and the proxies. Lines 105-106, not sure which series is being referred to in this sentence, and it's not clear what is meant by "is generally consistent with".*

AR: Done. We have added the context for $r = 0.37$ in the main text and removed the vague phrasing, including:

- The text now explains that this is an annual point-to-point Pearson correlation between a variance-attenuated tree-ring proxy and instrumental precipitation, for which a value around 0.4 is expected, limited by uncertainties in tree-ring reconstruction and explicitly write out what the previously unclear references refer to:

Manuscript · Page 3, Line 105–108

The year-to-year Pearson correlation coefficient between the N-WDI and the instrumental validation data is 0.35 ($p < 0.01$), which is similar to the collected natural proxy datasets (see **Supplementary Information Table S8** and **Figure 2a**).

RC: *Figure 2 caption also needs to be extended to more clearly indicate what is shown on the figure. Panel 2a: Clarify what the black line and grey uncertainty region represent. What are the dashed horizontal coloured lines? 2b: What are the values shown here –the methods describe the classification of the historical archives into 5 categories, not the extraction of a numerical value? And why are there only three colours when there are five categories? 2c: The open dots showing the locations of the historical data obscure the region of most interest –consider if there is a better way to show this.*

AR: Thanks for your suggestion. We have substantially expanded the Figure 2 caption to cover every panel, including:

- For **2a**, it now states that the black line is the 30-year low-pass reconstruction, the grey band is the 5 − 95% posterior credible interval of the Bayesian-integrated index, and the coloured horizontal dashed lines are the standardised thresholds separating the five WDI categories (SD/MD/N/MW/SW, labelled on the axis).
- For **2b**, it clarifies that the value shown is the regionally aggregated H-WDI, a continuous value ranging from 0 to 30 ordinal site records, displayed against the five severity levels and colour-coded dry⟶wet (the panel now uses the full five-level colour scheme, resolving the "only three colours" point).
- For **2c**, it identifies the colour bar ("Pearson r") and shrinks the site markers so they do not obscure the underlying correlation field.

RC: *From Figure 2, it appears that there are distinct 'dry' and 'wet' periods in the record. It would be valuable to show the time series of mismatches as well, as a simple visualisation of whether misclassifications are more likely to appear towards the end of these 'wet' and 'dry' periods.*

AR: We thank the reviewer for this insightful suggestion, which speaks directly to the SBS mechanism, and we have carried it out in full. We (i) plotted the time series of the per-year mismatch, expressed as the categorical offset between the archival and natural WDI (Δ = H-WDI level − N-WDI level; positive = the archive judges the year wetter than the proxy, negative = drier); and (ii) overlaid the wet/dry epochs, defined exactly as in Figure 2a (the sign of the 30-year low-pass N-WDI: wet where the trend is positive, dry where negative).

According to the figure, two findings emerge:

1. **The direction of the bias tracks the prevailing epoch, as SBS predicts.** The categorical offset is negatively correlated with the low-pass trend (Pearson $r = -0.23$, Spearman −0.23). Within sustained **dry** epochs, the archives systematically over-record wetness (mean offset +0.27; 46 wet-biased vs 20 dry-biased mismatches), whereas within **wet** epochs the bias is weakly reversed (mean offset −0.06). This is precisely the signature of a drifting baseline: inside a prolonged dry epoch, the "normal" reference shifts dry, so genuinely dry years feel ordinary and are under-recorded as dry (i.e. judged wetter than the proxy).

2. **The mismatches do *not* concentrate toward the *end* of epochs**, contrary to the specific timing the reviewer (reasonably) anticipated. Binning each year by its relative position within its epoch (0 = start, 1 = end), the mismatch rate is highest near the start (early 0.71 vs mid 0.55 vs late 0.55; corr(position, $|\Delta|$) = −0.09). In other words, misclassifications cluster just after a wet↔dry transition, when the lagging baseline disagrees most with the newly-established regime —still a lagging-baseline (SBS) phenomenon, but with a transition-onset rather than an epoch-end signature.

Because the epoch-level statistics rest on only six sustained epochs (≥ 10 yr; one of them 159 yr long), the findings are statistically underpowered, and we are careful not to over-interpret them. We have therefore added these analyses as a **Supplementary Figure** (**Supplementary Information S4, Figure S10**), summarised here, and we do **not** add a panel to the main-text figures, since the within-epoch timing pattern is not clean enough to feature, and the headline mismatch result (the shifted-comparison bias, **Figure 3**) is unchanged.

Methods

RC: *Line 324: Sentence is incomplete.*

AR: Done. The incomplete sentence was the "Due to poor retention rate … in the early period (1000−1469 CE)." fragment, which lacked a main clause. We have completed it:

> **Manuscript · Page 15, Line 343–346**
>
> Due to the poor retention rate (25.17%, measured as the ratio of archived meteorological data to the total quantity; see **Supplementary Information S1**) and obvious source bias (mainly based on official histories, with a relative lack of local and folk materials) in the early period (1000−1469 CE), we begin our analysis in

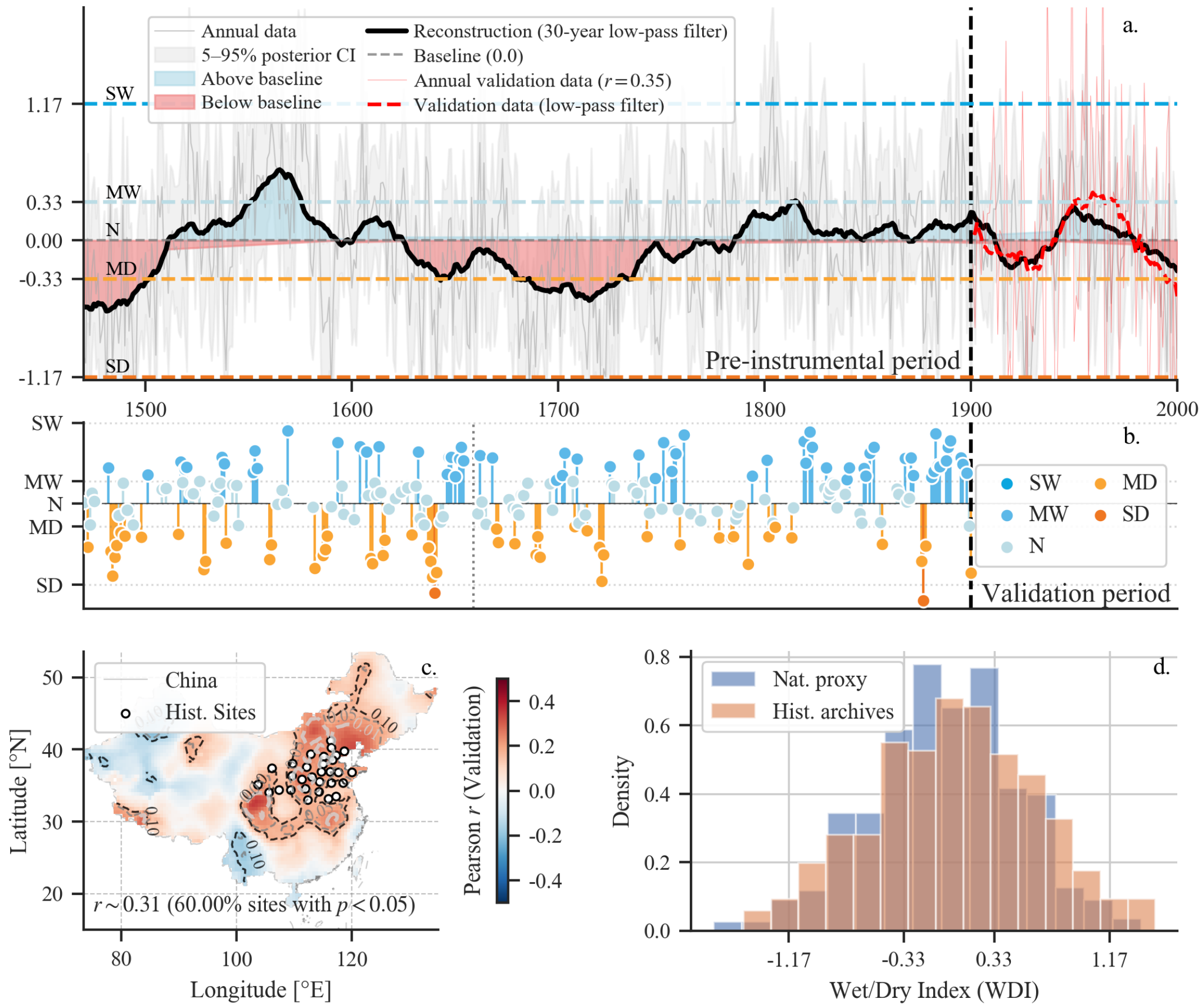

Figure 2: Reconstruction and validation of the WDI datasets. **a.** The natural-proxy WDI series (N-WDI) is integrated from tree-ring datasets (see **Methods** and **Supplementary Information S2** for details). The thin grey line is the annual N-WDI (posterior mean), the black line its 30-year low-pass reconstruction, and the grey band the 5 ∼ 95% posterior credible interval of the Bayesian-integrated N-WDI. Light-blue and light-red shading mark years above and below the baseline (z-score = 0), i.e. relatively wet and dry; the coloured horizontal dashed lines mark the standardised thresholds separating the five WDI categories, Severe Dry (SD), Moderate Dry (MD), Normal (N), Moderate Wet (MW), Severe Wet (SW), labelled on the left. For the validation period (1901–2000 CE), the thin and thick red lines show the annual and 30-year low-pass instrumental data (point-to-point $r = 0.35$). **b.** The regionally-aggregated historical-archive WDI (H-WDI) for each year, obtained by averaging the ordinal records of up to thirty sites; the resulting value is shown against the same five severity levels and colour-coded from orange (dry: SD, MD) through pale (N) to blue (wet: MW, SW). **c.** Spatial Pearson correlation between the integrated N-WDI and the gridded instrumental data over the validation period. Circles mark the thirty historical-archive sites (see **Supplementary Information S1** for details) (60% of sites significant at $p < 0.05$). **d.** Distributions of the N-WDI and H-WDI over the pre-instrumental period; both are approximately symmetric.

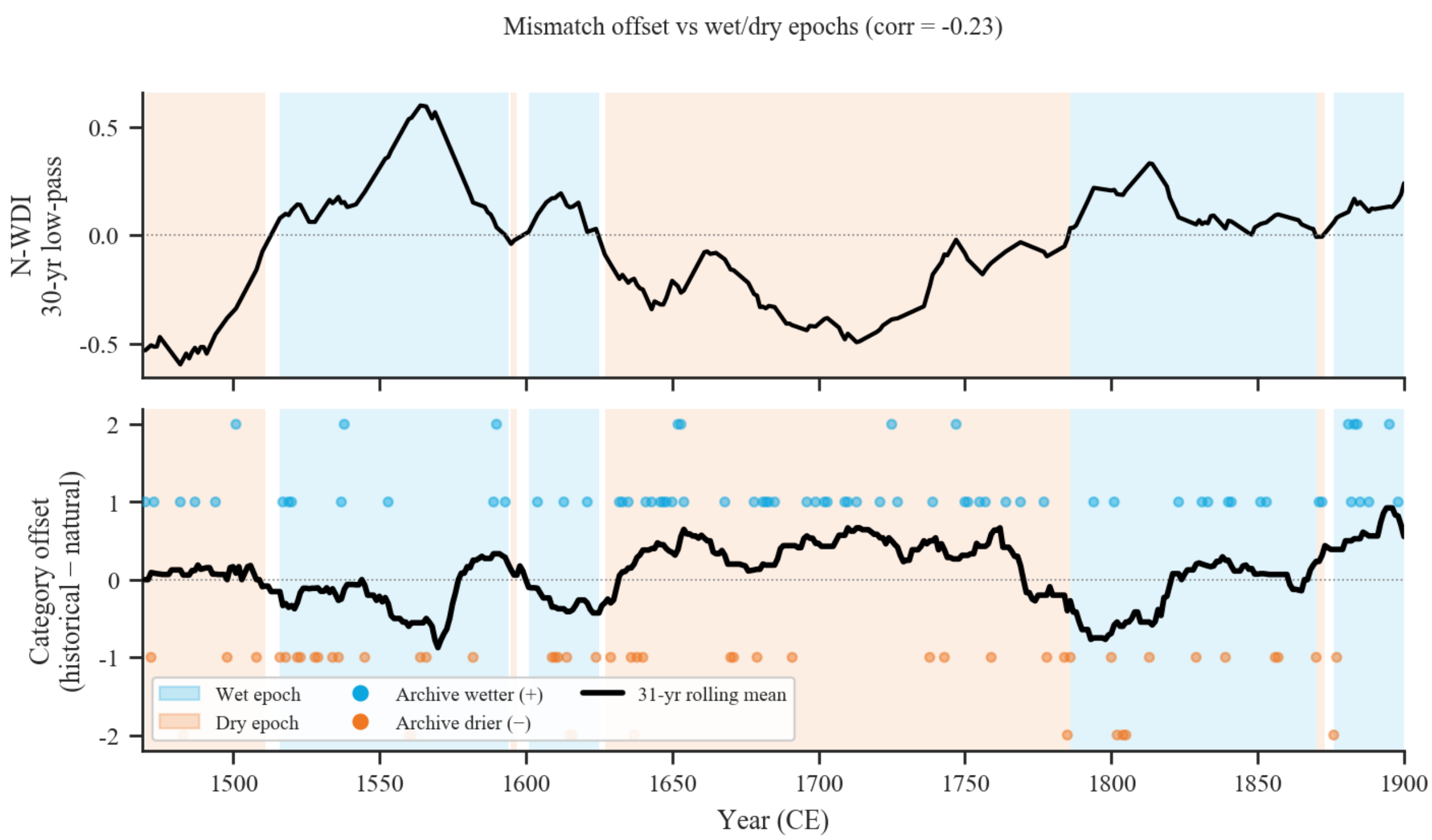

Figure S10: Temporal structure of H-WDI/N-WDI mismatches over 1470 – 1900 CE. **Top:** the 30-year low-pass N-WDI, used to define sustained wet epochs (blue shading, smoothed trend positive) and dry epochs (orange shading, smoothed trend negative). **Bottom:** the per-year categorical offset between the archival and natural series (Δ = H-WDI level − N-WDI level); points mark mismatch years where the archive read wetter (blue, $\Delta > 0$) or drier (orange, $\Delta < 0$) than the proxy, and the black line is the 31-year rolling mean of the offset. The rolling-mean offset is weakly negatively associated with the smoothed wet/dry state (overall correlation ≈ -0.23), i.e. the archive tends to read slightly drier than the proxy during wet epochs and slightly wetter during dry epochs, consistent with a baseline that drifts toward recent prolonged conditions.

1470 CE (see **Supplementary Information Figure S2**).

RC: *Line 346-347: It is not clear to me what probabilities are being extracted here. And why are the probabilities 10, 25, 30, 25 and 10% ? Ah, I see, these are not probabilities as such, but quantiles.*

AR: Thanks for raising this concern. The reviewer's reading is correct. These are the target category frequencies (quantile widths) used to bin the index, not probabilities in the inferential sense. We have corrected the wording in Methods at both places where the 10/25/30/25/10% appear; they now read:

Manuscript · Page 16, Line 396–397

To maintain consistency with the classification standards in H-WDI, we have chosen the same target category frequencies (quantile widths) for the five levels with same names (see **Table 1**).

RC: *Line 350: The process of spatial integration is also unclear, and could be made more plain. Why does the WDI for each site need to be randomly simulated? What happens when a site is missing data for that year?*

AR: After each site-year was assigned an ordinal drought–flood grade, we converted grades to continuous z-scores before spatial averaging, because ordinal levels cannot be averaged meaningfully. Each grade maps to a z-interval (not a single point); we therefore drew 100 values per site-year from a truncated normal within that interval and used the mean as the site-level continuous WDI. The regional series is the arithmetic mean across sites with available records in that year. Missing sites are excluded. If there are no site reports, the year is left missing. The regional mean is then reclassified to the five ordinal levels. This stochastic mapping is a tie-breaking convenience for cross-site averaging; a deterministic midpoint mapping yields nearly identical Kendall-τ window curves.

Manuscript · Page 15, Line 363–375

Generally, concrete determinations of floods or droughts, along with descriptions of severe societal outcomes of disasters, are classified as "Severe" level (SD or SW), while alone records of natural conditions (such as heavy rains or prolonged droughts) are classified as "Moderate" levels (MD or MW). In contrast, a normal (N) year refers to keywords "harvest", "abundant year", "ripe", and "bountiful", or non-abnormal events in the current year, with continuous records available in the preceding or following years (**Supplementary Information Table S3** provides more examples). To integrate archives across the study area, site-year ordinal grades were first converted to continuous z-scores, because ordinal levels cannot be averaged meaningfully. Each grade maps to a z-interval bounded by the same thresholds used for WDI classification (rather than a single point, see **Table 1**). It means, for the site i and year t, we drew 100 values from a truncated normal within that interval and used their mean as the site-level continuous WDI, $h_{i,t}$. The regional H-WDI was then $H_t = \text{mean}(\{h_{i,t}\})$ over all sites with non-missing records in the year t ($n_t \leq 30$). Sites without archival evidence were excluded rather than imputed, and years with no reporting sites were left missing. This stochastic mapping is a tie-breaking convenience for cross-site averaging.

RC: *Line 358: 'All the original reconstructions were calibrated with correlations above 0.4' –not clear what this means.*

AR: We have rephrased this for clarity. The sentence now states:

Manuscript · Page 16, Line 377–381

The reconstructed datasets are primarily collected from publicly available NOAA repositories (see **Supplementary Information S2** for details of data selection and processing). They are mainly based on tree-ring proxies of summer precipitation and Yellow River streamflow in northern China[26], and each original tree-ring reconstruction had achieved, in its source publication, calibration-period correlations with instrumental climate above 0.4 (see **Supplementary Information S2**, **Table S8**).

RC: *Line 362: Give a simpler explanation for how these datasets were integrated. Unclear why a different approach for integration is taken for these data than for the historical data, i.e. why not just take means for each year?*

AR: We have rewritten this in plain language and added the rationale for using a Bayesian model rather than a simple average. The natural reconstructions are few and differ in reliability, so we treat each as a noisy observation of one shared latent anomaly, using a Student-t observation model that is robust to outliers. The model then down-weights noisier or lower-skill series and carries each year's spread among the reconstructions into the posterior credible interval, whereas a per-year mean would weight every series equally and discard this uncertainty. The historical archives differ in kind, being ordinal site grades rather than continuous reconstructions, so they are aggregated using the spatial procedure described above (each grade is mapped to a continuous value and averaged across sites).

Manuscript · Page 16, Line 384–392

We then employed a Bayesian latent-variable model to integrate these datasets into a standardised N-WDI from 1470 to 2000 CE. Each year's "true" anomaly was given a weakly informative $\mathcal{N}(0,1)$ prior, and each reconstruction was modelled as a Student's t observation centred on it, with a shared degree of freedom ν estimated jointly rather than fixed (posterior $\hat{\nu} \approx 36$, i.e. close to Gaussian; see **Supplementary Information S2.3**). Then, weighting each series by its local (within-series) rolling variability, the model downweights noisier series and carries their year-to-year disagreement into the posterior credible interval. It was fitted by Markov Chain Monte Carlo (MCMC) in PyMC (version 5.28.4) using four No-U-Turn Sampler (NUTS) chains of 2000 draws each (1000 tuning steps, target acceptance 0.95).

RC: *Line 378: Please number equations. Note, these SD thresholds do not appear to give the right quantiles? I think the corresponding quantiles are 12, 37, 63, 88% ?*

AR: We will number all equations. The reviewer is right that $[-1.17, -0.33, 0.33, 1.17]\sigma$ correspond to cumulative probabilities of about 12/37/63/88%, not 10/25/30/25/10%. These thresholds are empirical rather than theoretical quantiles, and we have clarified this in the Methods. The five-grade scheme of the source atlas[25] is defined to reach target grade frequencies of about 10% for the most severe grades, and the thresholds were fitted to the observed frequency distribution of summer precipitation, which is only approximately Gaussian. The 12/37/63/88% the reviewer notes is exactly this small, expected departure of the empirical thresholds from the idealised normal quantiles. We keep the thresholds and the nominal percentages unchanged, and no results change.

Manuscript · Page 15, Line 357–362

All archives related to drought-flood events were then classified into five levels of severity: Severe Drought (SD), Moderate Drought (MD), Normal (N), Moderate Wet (MW) and Severe Wet (SW), with the target frequencies of the five levels approximately controlled at 10%, 25%, 30%, 25%, and 10% (i.e., category quantile widths, see **Table 1** and **Supplementary Information S1 and Figure S3**). This approach, a common practice for inferring Wet/Dry Index (WDI) levels from historical archives[27,28], was applied to each historical site (see **Supplementary Information Table S2**).

RC: *Section 4.2.2 Bias of mismatches detection. I have read through this methods section several times now, alongside the corresponding results and figure, and I am still having difficulty understanding what is being done here. I understand that we are looking at the years where the historical data and natural proxy data do not match. We then go back through the record to find the most recent year with the same historical value (although it is not clear whether this is numerical value or the classified value). We then determine the difference between the natural proxy values of the two years (delta t, t'). However, it is quite unclear to me what is done with this (delta t, t') value. I cannot understand the results statement 'In these mismatch cases, the severity level from historical archives is usually categorised as drier (or wetter) than those observed in natural proxies when WDI values are only slightly lower (or higher) compared to a previous year. This "shifted-correlation pattern" is most apparent when the shift is 2, in which case the absolute value of these WDI differences is approximately 0.04 on average'. This section and these results need to be made much clearer and plainer. What is the shift? Does WDI in this sentence refer to the historical data or the natural data? Which WDI differences are we talking about? It might be helpful to come up with some clear notation for each set of data being discussed, and use this notation consistently through the text.*

AR: We agree this was the most opaque section and have rewritten it around explicit notation. The revised the corresponding sections in Results and Methods as follows:

- We concretely define the symbols and use them accordingly throughout. For instance, the two datasets are N-WDI and H-WDI. To distinguish continuous and categorised values, we also define N_t and H_t as the five-level N-WDI and H-WDI categories data for the year t, and x_t is the continuous N-WDI value. So, a "mismatch" is a year with $H_t \neq N_t$ and the actual difference of natural climate at this mismatched point is "$\Delta_{t,t'} = x_t - x_{t'}$".
- We now make explicit that the "look-back match" is on the discrete category H, not a continuous value. This means for $t' < t$, there is $N_{t'} = N_t$, where $shift \in \{1, 2, 3\}$ is defined as selecting the $shift$-th most recent earlier year with $H_{t'} = H_t$;
- We now state plainly what the standardized difference $\Delta_{t,t'} = (x_t - x_{t'})/\sigma_{\text{global}}$ measures (how much the *underlying* climate actually differed between two years the archive recorded at the same severity) and how its per-cell average forms the bias matrix that the Monte-Carlo test then evaluates.

Taken together, this whole section's approach now reads like this:

Manuscript · Page 17, Line 422–452

We write N_t and H_t for the five-level (ordinal) categories assigned at year t by the natural proxy (N-WDI) and the historical archive (H-WDI), respectively, and x_t for the underlying continuous N-WDI value. A mismatch is a year where $H_t \neq N_t$. We tested whether mismatches show a systematic signature of comparing the current year to earlier years with the same natural severity category. For each mismatch year t we therefore identified a reference year t' at which the N-WDI had previously been assigned the same category. Formally, t' is the calendar year of the $shift$ times prior observation with the same natural severity category, i.e. $N_{t'} = N_t$, $t' < t$, where the integer $shift \in \{1, 2, 3\}$ denotes the first, second, or third such prior occurrence (**Figure 3b**). Note that the matching is on the discrete category N, not on a continuous scale. We then asked how much the underlying natural climate had actually changed between these two years sharing the same natural category of N-WDI, quantified as the z-score difference of the continuous N-WDI values x_t and $x_{t'}$ only for the mismatches ($H_t \neq N_t$), i.e., $\Delta_{t,t'} = x_t - x_{t'}$; where x_t and $x_{t'}$ are the continuous N-WDI (natural-proxy) values at the current and reference years. As a control comparison, we also input the severity levels inferred from instrumental data (validation period, 1901–2000 CE) as a synthetic "historical sequence" H_t into the above approach to calculate $\Delta_{t,t'}$, assessing whether the mismatching pattern appears only in historical archives.
A non-zero $\Delta_{t,t'}$ of continuous natural values, therefore, indicates the baseline has drifted and its sign gives the direction (a positive $\Delta_{t,t'}$ indicates the archive over-estimated severity relative to the earlier event, and vice versa). Averaging $\Delta_{t,t'}$ within each cell (i, j) (natural level i against archival level j) yields the observed bias matrix Δ_{ij}^{obs} over mismatch years only. We assessed the statistical significance of this bias pattern with a Monte Carlo randomisation test that compares the observed bias matrix against a null model in which archival severity categories are assigned independently of the natural climate sequence. The null hypothesis is that archival classifications depend only on their fixed marginal frequencies $P(H = j) = p_j$ ($\{0.10, 0.25, 0.30, 0.25, 0.10\}$), with no association between archival grades and the natural series. Each iteration generated a synthetic dataset in which natural climate values $x_{\text{sim}} \sim \mathcal{N}(0, 1)$ were binned into five severity levels using the same thresholds applied to the proxy data, and archival classifications were independently redrawn from p_j. We ran 1,000 iterations, each with a sample size matching the empirical record length ($n = 432$). For each iteration k, the bias matrix $\Delta^{(k)}$, indexed by natural level i and archival level j, was recomputed with the identical look-back algorithm used on the empirical data (the same $shift$ and within-grade pairing $N_{t'} = N_t$, $t' < t$). This null preserves the archival marginals and the look-back mechanics but destroys any dependence between archival assignments and the natural climate sequence. For each matrix cell (i, j), we computed standardized scores $z_{ij} = (\Delta_{ij}^{\text{obs}} - \mu_{ij}^{\text{null}})/\sigma_{ij}^{\text{null}}$, where μ_{ij}^{null} and $\sigma_{ij}^{\text{null}}$ are the mean and standard deviation of $\Delta_{ij}^{(k)}$ across the 1,000 iterations, and obtained two-tailed p-values as $p_{ij} = 2(1 - \Phi(|z_{ij}|))$.

RC: *Figure 3(c-f) I also find opaque. I am not sure what the 'shifted categories' are. An expanded caption that clarifies what is shown would help.*

AR: Sorry about the previous confusion. Figure 3(c–f) now uses the consistent H-WDI/N-WDI notation, and the expanded caption explains the "shifted categories". The revised results based on these panels now read:

Manuscript · Page 5, Line 133–137

To further explore the "comparing to the past" process implied by the SBS theory, we compared each mismatched H-WDI data with a similar prior event which occurred once, twice, or three times ($shift = 1, 2, 3$, respectively) in the past (**Figure 3b**). In these mismatch cases, the H-WDI is usually categorised as drier (or wetter) than N-WDI when the z-score values of N-WDI are only slightly lower (or higher) compared to a former year.

RC: *The re-standardisation approach is somewhat easier to follow. However, I would advocate not using the word 'filter' when referring to re-standardisation –this causes some confusion because of earlier processing steps including 'low-pass' filtering. I would stick with 're-standardised' data.*

AR: Agreed, and this echoes a related comment from another reviewer about the "filter" terminology. We have removed "filter" wherever it refers to re-standardisation and use "re-standardised" consistently, reserving "filter" only for the genuine low-pass step (and for unrelated data-screening).

RC: *Figure 4: Be careful about wording; Figure 4a compares 'original' to 'filtered' data but I think the 'filtered' data here are actually the re-standardised values? Indicate the value of 's' for Figure 4a. Figure 4b, scale needs an additional decimal place. What do Max tau = 0.22 and Avg tau = 0.10 refer to?*

AR: We thank the reviewer and address each point as follows:

- **"filtered" → "re-standardised" (4a).** The reviewer is right: the second series in 4a is the re-standardised one, not a low-pass-filtered one. We have relabelled it "re-standardised" in the figure legend (and use that term consistently, per the related comment above); the caption already refers to "restandardisation ⋯to the original (non-re-standardised) case".
- **Value of "s" in 4a.** The horizontal axis of 4a is the sliding-window size (labelled "Window size, w"); it is swept continuously across the panel, so it is displayed directly along the axis rather than being a single fixed value to annotate. (The symbol "s" was used only for the mismatch "shift", which we kept distinct.)
- **4b "scale".** The two axes of 4b are the sliding-window *parameters* —window size and the minimum-sample requirement which are integer-valued, so additional decimal places on these ticks would not be meaningful. The colour bar (the change in τ) already carries two decimals, so we have made the axis labels and units explicit instead.
- **"Max τ" and "Avg τ" (panel c).** These are averages across the time segments: **"Max τ" is the mean of the maximum *re-standardised* Kendall's** τ (each segment at its own optimal window), and **"Avg τ" is the mean *benchmark* (non-restandardised) Kendall's** τ. Their difference is the improvement in mean correlation. We agree that the bare labels are easy to misread and have spelt this out in the revised caption.

RC: *The length of the optimal restandardisation window looks from Figure 4a and b to be around 15-20 years, yet the text states that the outperformance occurs with windows of 20-40 years, with an optimal sliding window size of 34 years. This suggests that the results from the whole time period contradict the results from shorter time periods. I think it would be simpler and clearer to focus on the results from the whole time period, rather than subsets of the data.*

AR: The apparent contradiction came from mixing the whole-period and per-segment optima without labelling them. We have separated the two in the text and now present the whole-period result as the main line, with the per-segment values kept as a clearly labelled, more local measure. We also note that the rolling-window minimum-sample correction raised by another reviewer slightly shifted the whole-period optimum. After that correction, the mean best window is 33 years, consistent with the 20–40-year band.

RC: *The agent-based modelling approach is also novel and very interesting, but could be made clearer. With the 'collective memory' setting, which years does each agent use as reference? Do they have the collective memory of the full time period, or only access to the collective memory in their own lifespan? (Line 467, memory seems to start at e(t-2) –please correct).*

AR: Thank you. Under the collective baseline (now corresponding to "H2"), each agent references the shared archive accumulated over the full elapsed record, not only the years within its own lifespan. Under the personal baseline (now corresponding to "H1"), each agent references only its own lived experience. We have made this explicit in the Methods:

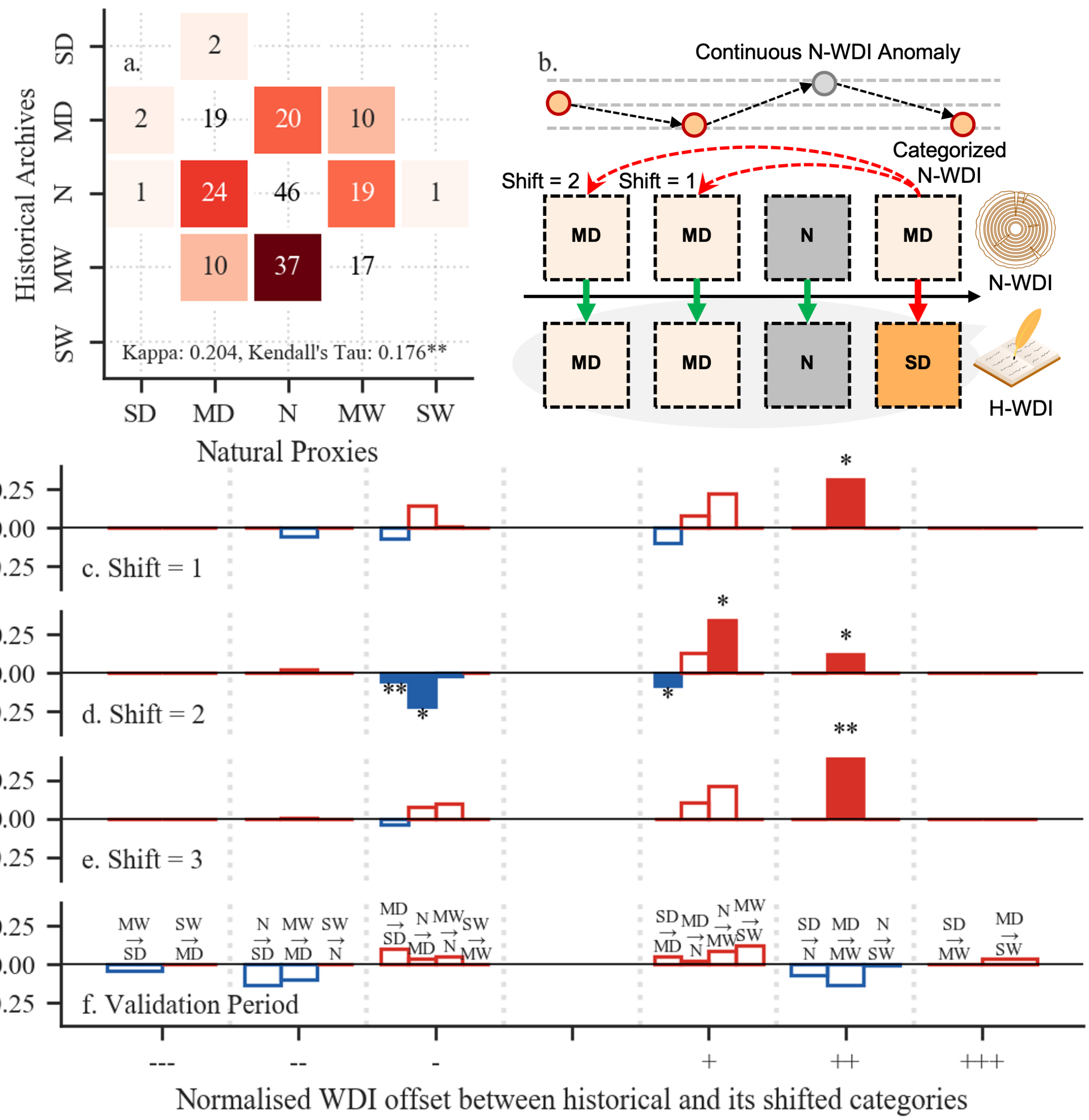

Figure 3: Mismatch pattern between N- and H- WDI levels. **a.** Confusion matrix, showing the number of matched (diagonal, white colour) and mismatched (off-diagonal, red colour) data of climatic extreme levels between natural and archival data. **b.** A schematic diagram illustrating the shifted-comparison of mismatches. The baseline is the WDI data from a natural proxy (N-WDI), taken as the non-perceptual reference severity of extremes. Mismatched data points (with red arrow) from historical data (H-WDI) are compared with a look-back data (where $shift$ indicates a prior event with a similar N-WDI level) to observe whether there is a difference in N-WDI anomalies. **c.-e.** WDI offset between natural and archival data with a different shifted comparison. The y-axis represents the offset of N-WDI z-scores between current and previous extreme events, and the x-axis represents how different H-WDI levels are underestimated or overestimated relative to the corresponding N-WDI levels. (each "+" denotes one level wetter, e.g., Normal to Moderate Wet; each "-" denotes drier. e.g., Normal to Moderate Dry) Significance is assessed by a Monte Carlo randomisation test: H-WDI levels are independently redrawn at fixed frequencies, and synthetic natural values are drawn from $\mathcal{N}(0,1)$, and the same look-back procedure ($shift = 1, 2, 3$) is applied (significant bars are filled in colour, where * denotes p < 0.05 and ** denotes p < 0.01; see **Methods**). **f.** No similar significant "shifted-correlation pattern" is detected for the instrumental data during the validation period (1901-2000 CE, $shift = 1$). More details about this null setup are available in **Supplementary Information Figure S11**.

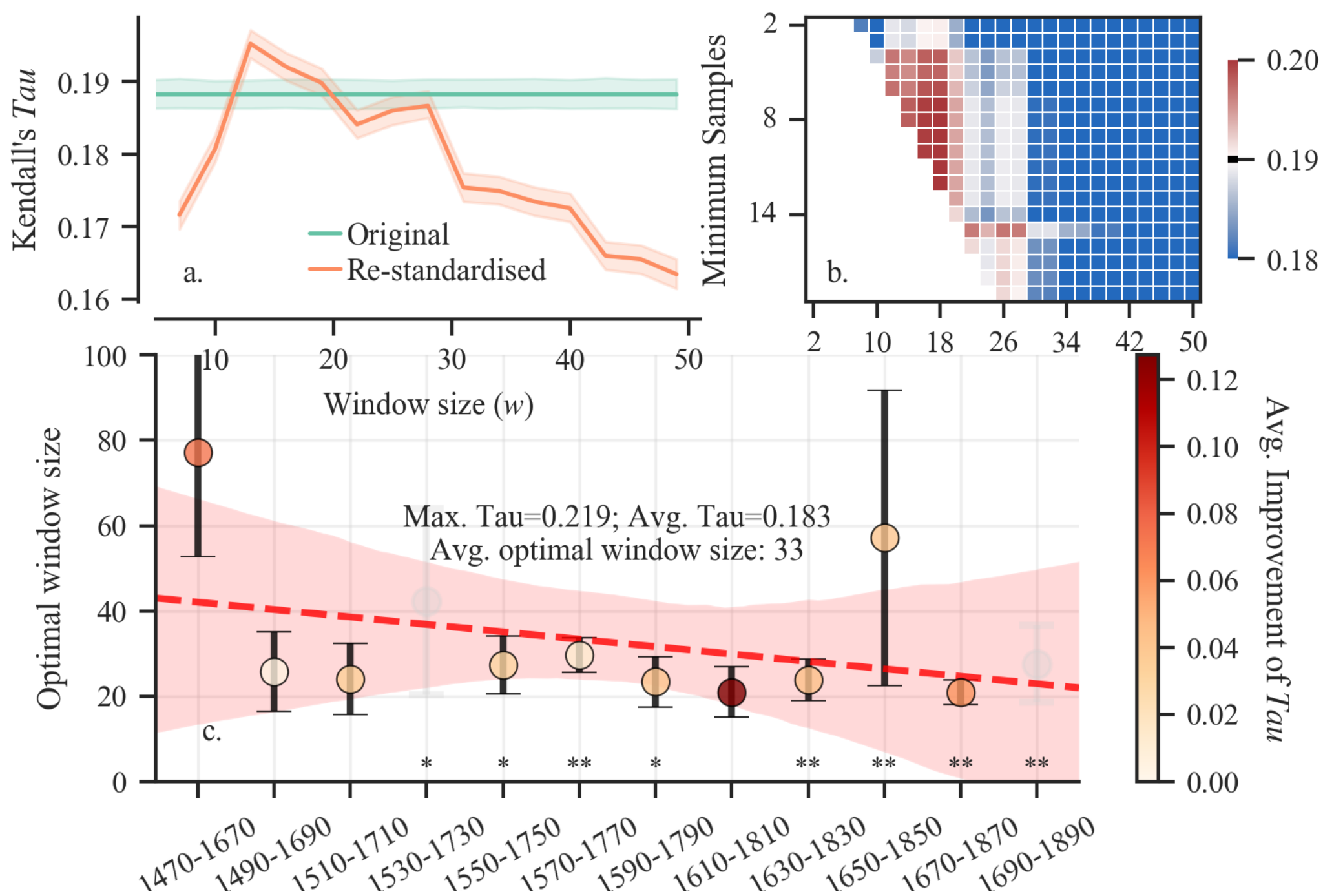

Figure 4: Correlation between N-WDI and H-WDI. **a.** Comparing the average Kendall's τ after application of a restandardisation to the N-WDI under different sliding window sizes (w) to the original (non-restandardised) case. **b.** The same test with variation under various combined parameters (window size and required minimum samples) of the sliding window and changes in correlation. Red areas indicate parameter combinations that increase relative to the whole-period correlation benchmark ($\tau = 0.190$). **c.** Applies the restandardisation approach across different time segments (starting from 1470 CE, each segment covers 200 years, and tests every 20 years). The window sizes with the top 10% highest Kendall's τ between the N-WDI and the H-WDI are shown. The optimal window size w indicates where sliding windows increase the correlation between N- and H- WDIs. The increments spanning different segments show a general significant improvement despite differences in historical institutions and archives (see **Supplementary Information S1 and Figure S2** for details): "Max τ" and is the maximum re-standardised value across segments (each segment evaluated at its own optimal window), and "Avg τ" is the mean benchmark (non-restandardised) Kendall's τ. The average optimal sliding window size is 33 years for the segments. A transparent bar indicates no relevance improvement in that segment, and the $5 \sim 95\%$ credible interval comes from the fitted line.

Manuscript · Page 19, Line 507–509

In the model, there are two types of baselines available for agents comparison: a personal baseline (i.e., μ_i and σ_i are calculated from the agent's own memory M_i) and a collective baseline (i.e., μ_i and σ_i are calculated from the public climate record accumulated over the full elapsed period, $e_1, \ldots, e_{t-1}, e_t$).

To answer the horizon question directly, the source and the temporal span of the baseline are now an explicit part of the design. Besides the personal and full-archive collective baselines reported in the main text, we also tested a collective archive restricted to each agent's own lifespan. This lifespan-windowed collective baseline still does not reproduce the generational optimum and behaves more like the full-archive collective than the personal baseline (see **Supplementary Information Figure S9**), indicating that the generational window arises from individual lived experience rather than a bounded memory horizon alone.

Supplementary Information · Page 22, Line 343–348

The model achieves this by setting up a baseline comparison for each agent before recording. Four baselines are implemented; they are most easily understood as a 2×2 design that crosses two binary factors (Table S10) (i) the *source* of the remembered baseline (the agent's own experience vs. the shared collective archive) and (ii) its *temporal horizon* (restricted to a single agent-lifetime, i.e. generational, vs. the full elapsed history, i.e. cumulative):

Table S10: The 2x2 baseline scenario matrix of the ABM, crossing the source of the remembered baseline (the agent's own experience vs the shared collective archive) with its temporal horizon (a single agent-lifetime vs the full elapsed record). The diagonal cells are the two main-text hypotheses (personal, H1 generational amnesia; collective, H2 collective illusion), while collective_lifetime and model are the decomposition and non-perceptual benchmark controls.

	**Generational horizon (one agent-lifetime)**	**Cumulative horizon (full elapsed history)**
Individual source (own memory)	personal —H1: generational amnesia	(idealised limit → model, see below)
Collective source (shared archive)	collective_lifetime	collective —H2: collective illusion

Only the diagonal H1/H2 contrast is foregrounded in the main text, but all four scenarios can be compared at the **Supplementary Information Figure S8**:

Supplementary Information · Page 23, Line 367–371

Only the diagonal H1/H2 contrast is foregrounded in the main text; `collective_lifetime` and `model` are reported here as decomposition/benchmark controls, and their full correlation profiles are presented with the robustness analyses below (Figure S8). The empirical basis for the model is derived from analyses of historical climate records from northern China (1470–1900 CE), tree-ring reconstructions (validated for 1901–2000 CE), and the psychological literature on cognitive biases in environmental perception (see main texts).

RC: *The simulations could potentially be more interesting if there are long-term variations in the 'real' climate: at present, climate is a normal distribution with constant mean and sd, but this could be modified by adopting a low-frequency variation in the mean climate, to simulate inter-decadal variations in monsoon patterns.*

AR: We have done exactly this in this revision. The main analysis now uses an AR(1) persistence forcing, and we additionally run a trend-plus-noise forcing that introduces a low-frequency variation in the mean, together with the original i.i.d. case, as a climate-forcing robustness matrix. Across all of these, the emergent optimal window stays within the 20–40-year generational band, so the result is not an artefact of a constant-mean white-noise assumption.

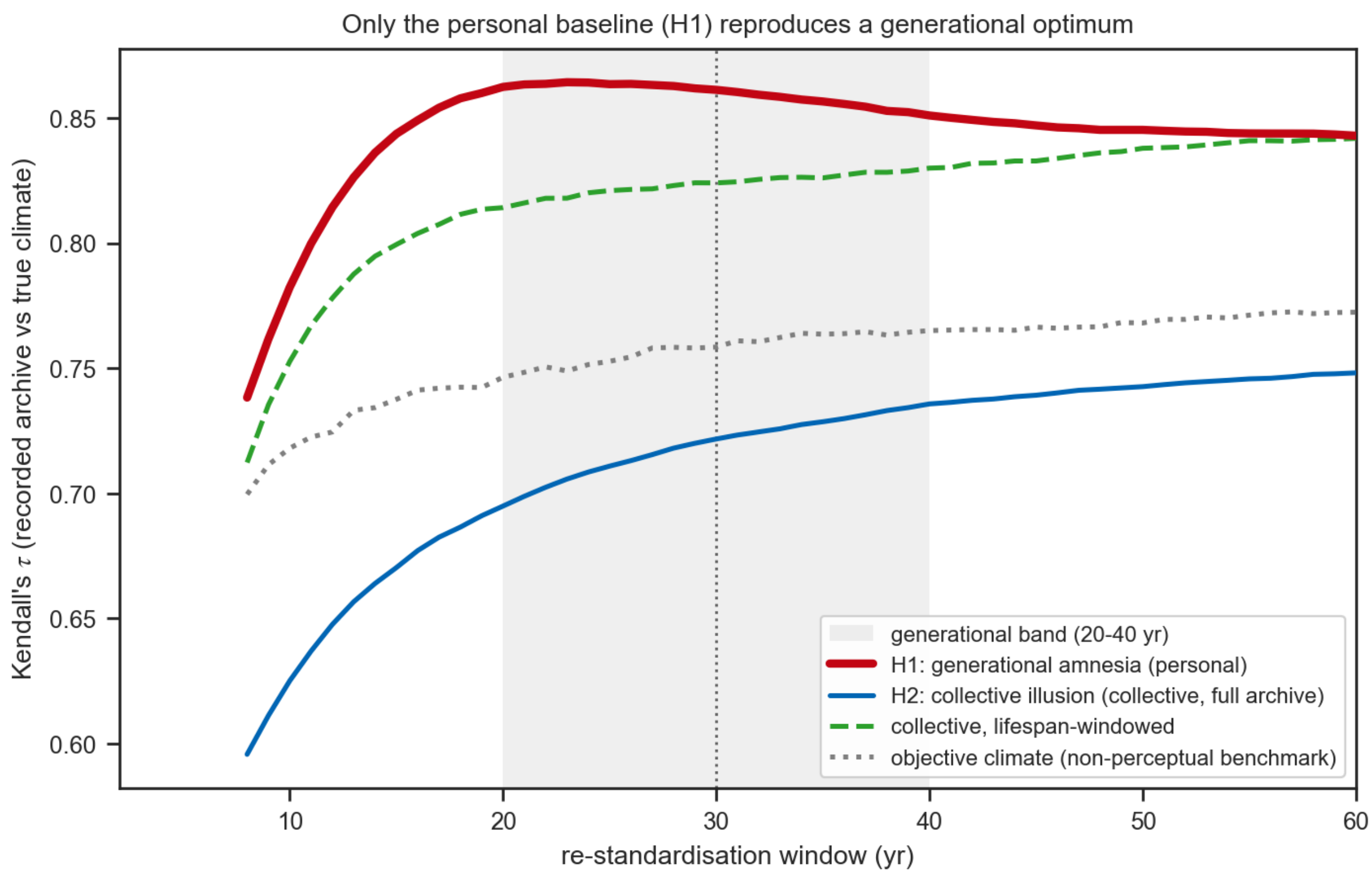

Figure S8: Window–τ correlation profiles for all four baselines of the 2×2 design, each averaged over replicate runs under the AR(1) main forcing. Only the `personal` baseline (**H1: generational amnesia**, red) peaks within the 20–40-year generational band (shaded; dotted line at 30 yr). The full-archive `collective` baseline (**H2: collective illusion**, blue) rises monotonically with window length; the `collective_lifetime` control (green dashed) tracks H2 rather than H1 despite sharing H1's bounded horizon; and the non-perceptual `model` benchmark (grey dotted), referenced to the objective climate, shows no generational peak. The contrast isolates individual lived experience — not a bounded memory horizon per se —as the source of the generational optimum.

Manuscript · Page 7, Line 168–176

The heuristic ABM represents climate as a stationary AR(1) stochastic process ($c_t = \phi c_{t-1} + \varepsilon_t$, **Figure 5a**; alternative independent and trended forcings are tested for robustness in **Supplementary Information S3 and Figure S9**) observed by a continuously turning-over population of finite-lifespan agents. Each agent judges the current anomaly against a baseline, and is more likely to log an event the more it departs from that baseline (**Figure 5c**). We designed scenarios to represent the "baseline", based on lived experience or shared archives of agents, corresponding to the two hypotheses of "H1 generational amnesia and H2 collective illusion" (**Figure 5c**), with two other scenarios tested for robustness (see **Supplementary Information S3, Table S10 and Figure S8**). The archive contains logged severities from all agents and is aggregated and classified into the same five-level WDI series, so the simulated and empirical records are compared identically (see **Methods**).

RC: *Figure 5 only shows the outcome of the ABM after re-standardisation with a particular window size. It would be valuable to demonstrate the outcomes before re-standardisation as well, to show more clearly the effect of SBS.*

AR: Because the unrenormalized data showed no clear pattern, we followed the other reviewer's suggestion and included a Sabol sensitivity analysis figure. We hope you will agree that this comprehensive parameter sensitivity analysis better communicates our core conclusion: generational amnesia based on specific max-age (life expectancy) plays a central mechanistic role behind SBS.

RC: *Again, extending the caption to Figure 5 would assist in understanding. I'm not sure exactly what output is shown in panel (b): the label "correlation differences (times of sd)" is not at all clear. Is this the improvement in correlation between 'true' and 'recorded' climate with re-standardisation to a given window size? I don't understand why this is monotonic for the 'collective' memory but peaked for the 'personal' (or 'individual'?) memory.*

AR: The reviewer's interpretation of panel (b) is correct. The correlation improvements between the "true" and "recorded" climate as a function of restandardisation window size. The monotonic-vs-peaked contrast is, in fact, the key result.

- In collective memory, the reference baseline tracks the climate's (autocorrelation) structure, so improvement grows monotonically with window size; in personal memory, the baseline is bounded by agents' finite lifespans, producing an inverted-U that peaks at the generational scale. Our new global sensitivity analysis (revised **Figure 5**) also confirms this: the personal-memory peak location is driven by lifespan, whereas the collective-memory window is driven almost entirely by climate persistence. The revised related results now read:

Manuscript · Page 7, Line 176–181

Reproducing the empirical observation measured in historical archives, the correlations between recorded climate by agents and the input climate forcing show the unimodal pattern at an intermediate generational restandarised window under the scenario of "H1: generational amnesia" (**Figure 5b**, red and black lines). In contrast, the scenario "H2: collective illusion" shows a monotonically increasing trend with window length and no generational optimum (**Figure 5b**, blue line).

- Regarding why collective memory produces a monotonic curve, our hypothesis is that this precisely reflects a "learning from the past": if we can perceive (through historical archive) farther back, we can correspondingly match perceived climatic changes with actual normalities over longer time scales. Based on this finding, we also briefly stated the importance of "remembering the past as resilience" in the introduction:

Manuscript · Page 2, Line 48–50

Understanding the relationship between collective memory and environmental extremes can thus play a significant role in long-term social resilience and adaptation to hazards[29] (i.e., remembering as a form of resilience[30]).

- and in the discussion:

Manuscript · Page 13, Line 276–279

As collective memory can be purposefully cultivated to enhance resilience[30], it underscores the value of science and history education in establishing correct public awareness of climate change. This is still significant nowadays. Although we have instrument-based measurements and more accurate data, the duration of collective memory can disappear more rapidly[31], with similar decaying patterns across different loss rates[32].

RC: *It would also be useful to discuss how the recording lifespan compares with the timescale on which improvement occurs for the personal memory. The typical recording lifespan is 10-60 years – so why is the timescale for improvement so much shorter? I was expecting a more detailed sensitivity analysis around lifespans, but the sensitivity analysis in the Supplementary is quite cursory – and the relevant Figure S5 is not clearly captioned. Please do ensure that figures in supplementary have full explanatory captions.*

AR: We have substantially expanded the sensitivity analysis exactly as the reviewer hoped: the cursory ANOVA is replaced by a full two-stage global analysis (Morris screening + Sobol decomposition with bootstrap CIs), reported separately for personal and collective memory. This both confirms that lifespan dominates the personal-memory peak:

Manuscript · Page 10, Line 181–187

Global (Sobol) sensitivity analysis traces this divergence to its causes by measuring all five parameters (max age, number of new agents, archive loss rate, climate persistence, and variability; **Figure 5d**). Under the scenario H1, the location of the optimal window is determined almost entirely by an agent's lifespan (maximum effect index $S_T = 1$; **Figure 5d**) and is directly proportional to it (see **Supplementary Information Figure S7**). In contrast, if one assumes that agents use the shared archive as the baseline (H2), the above pattern cannot be reproduced (**Figure 5d**), regardless of how long agents have access to the archives (**Supplementary Information Table S10 and Figure S8**).

- Regarding the issue of the time scale, we first apologise for the typo in the main text: we actually used 40 years as the value for the max age parameter. We initially guessed that the literate elite could live to 60 years (wrongly) and tested that number, but later found literature supporting ~40 years and forgot to update it (sorry!). In addition, we have now added to the supplementary information a summary of life expectancy based on more extensive sources, which is 40 years. We hope this explanation resolves your concerns about the time scale.

RC: *I took a very brief look at the code. I don't use python, so did not attempt to download and run. However, the directory looked to be well organised and well documented.*

AR: We thank the reviewer for looking at the repository and for the kind words on its organisation and documentation. In this revision, we have additionally fixed the code issues raised by other reviewers and added regression tests, further improving the codebase.

5. Reviewer 6

RC: *The manuscript is well written and based on a very interesting combination of two rich and compelling datasets. The interdisciplinary approach, combining tree-ring data with historical records to explore the influence of collective memory on the interpretation of weather events, is particularly intriguing. I found both the introduction and discussion engaging and easy to follow, while the methods and results sections were somewhat harder to navigate due to their technical nature.*

AR: We thank the reviewer for this warm and encouraging assessment, and for highlighting the interdisciplinary combination of tree-ring and historical data as a strength. We have taken the point about the methods and results being harder to follow very seriously. In revision, we introduce consistent notation for the two series, expand the figure captions, and rewrite the most technical passages for accessibility (echoing similar suggestions from other reviewers).

RC: *I also appreciated the examples from the historical sources provided in the supplementary material, especially Table S3. If space allows, I think this table could be moved to the main manuscript, as it conveys the nature of the historical sources, while illustrating how they were interpreted.*

AR: We are glad Table S3 was helpful, and we agree it conveys the texture of the historical sources well. Due to space constraints, we will not move the full Table S3 into the main text, but we have included one corresponding example for each category when explaining the research methods.

RC: *I am not entirely convinced about the added value of the ABM in its current form. While the idea of comparing individual memory with access to collective memory is interesting, it remains somewhat unclear what additional insights the model provides beyond the empirical comparison between tree-ring data and historical records. I would therefore encourage the authors to clarify more explicitly why the ABM is an integral part of the study.*

AR: We thank the reviewer and agree that we understated this. The empirical comparison establishes that a roughly 30-year re-standardisation window improves the alignment between the archival and the natural WDI, but on its own, it cannot say why that window exists or which mechanism produces it. The SBS literature offers two candidates that are indistinguishable in the archives alone: generational amnesia, in which each generation's baseline is bounded by its own lived experience, and collective-memory illusion, in which shared narratives reshape how earlier conditions are remembered. The ABM is what lets us separate them. Running the same model under a personal (lived-experience) baseline and a collective (shared-archive) baseline, only the personal baseline reproduces the empirical generational optimum, whereas the collective baseline continues to improve toward much longer windows (**Figure 5b**). The global sensitivity analysis traces this difference to its cause: under the personal baseline, the optimal window is set almost entirely by agent lifespan, while under the collective baseline, it merely tracks the climate's own persistence and so cannot produce a generational scale. A collective archive restricted to each agent's lifespan also fails to yield the optimum, indicating that the effect stems from individual experience rather than a bounded memory horizon alone. The ABM's contribution is therefore causal rather than illustrative: it identifies generational amnesia, not collective illusion, as the SBS mechanism consistent with the data, an inference the empirical comparison cannot make on its own. We now state this explicitly in the main text and present the ABM as a mechanism-discrimination test rather than a fitted predictor.

> **Manuscript · Page 10, Line 187–189**
>
> This heuristic model suggests that generational amnesia (assuming individuals rely on their lived experience) can cause generational inconsistencies, whereas collective illusion is insufficient to reproduce the observed mismatch pattern in the empirical WDIs.

To reaffirm the importance of ABM, we made many changes to the manuscript, including revised **Figures 1 and 5** and an entirely revised **Section 2.4**. Relevant citations can be found in the reviewers' comments in our response and in the revised main text.

RC: *I also wonder how sensitive ABM was to different remembering ages of individuals? Was the model stress-tested to assess whether the results were robust across different age assumptions? Also, was Lmax set to 40 or 60 years? There seems to be a discrepancy between the manuscript and the supplementary information.*

AR: Yes, the model has now been stress-tested along exactly this axis. We replaced the earlier ANOVA with a global,

variance-based Sobol sensitivity analysis, sampling agent lifespan (L_{max}) across the full 15 to 80-year range, well beyond any single plausible value. Two results follow. First, the peak-window location varies smoothly and monotonically with lifespan and stays within the generational band throughout, so the optimum is not a knife-edge that holds only at one age assumption. This dependence is itself the mechanism, since finite memory is what generates the window, rather than a fragility. Second, within that range lifespan is by far the dominant driver of the peak-window location under the personal-memory baseline, accounting for about 94% of the variance (first-order $S_1 \approx 0.94$, total-effect $S_T \approx 1.00$), while recruitment, archive loss, and the climate's variability and persistence together contribute less than 6% (each total-effect below 0.06). The roughly 30-year optimum that matches the archives is therefore robust to the other assumptions and arises, to first order, from the historically appropriate lifespan alone.

Manuscript · Page 12, Line 265–269

> Finally, the ABM is a deliberately minimal, heuristic model rather than a demographic reconstruction. This is mainly because the groups that produced the historical archives do not represent the entire population, and our global sensitivity analysis (**Figure 5d**) shows that their life expectancy matters. We have confirmed the key parameter in this simplified demography is consistent with population data we have collected (**Supplementary Information Figure S6**).

On the discrepancy the reviewer caught, the intended default is $L_{max} = 40$ years, the value used throughout the code, the run configurations, and the Supplementary Information. The stray $L_{max} = 60$ in the Methods was a typographical error, now corrected to 40 so that the main text, the **Supplementary Information**, and the released code all agree.

RC: *The manuscript uses objective vs subjective when referring to the two datasets, where the tree-ring data is considered objective and the historical archives subjective. This distinction becomes somewhat reductive, since neither dataset is entirely straightforward. Tree-ring records are also interpreted proxy data, shaped by sampling, methodological choices, and assumptions about environmental responses. At the same time, historical archives should not simply be treated as "subjective". I think this distinction could simply be removed.*

AR: We fully agree, and we have adopted this suggestion throughout. We have:

- removed the "objective vs subjective" dichotomy from the text and figures and replaced it with two neutral, descriptive labels (the historical-archive WDI: H-WDI and the natural-proxy WDI: N-WDI) and used them consistently. (As a side benefit, this neutral labelling also implements the consistent two-series notation requested by Reviewer #2.)
- no longer described the tree-ring series as "objective", but state explicitly that it too is an interpreted proxy shaped by sampling and methodological choices; what distinguishes it is only that it does not pass through human memory, making it a relatively independent, non-perceptual reference, whereas the archival series is mediated by contemporaneous perception and is therefore comparatively more exposed to perceptual bias. This relative asymmetry, rather than an absolute objective/subjective split, is what our analysis actually relies on. The corresponding sentences in the main text now read:

Manuscript · Page 14, Line 322–327

> We treat the natural-proxy WDI (N-WDI) reconstructed from tree-ring data as a relatively independent, non-perceptual reference for climatic anomalies. It is independent not because it is free of interpretation (it, too, is a reconstructed proxy shaped by sampling and methodological choices), but because it does not pass through human memory and is compared with the historical-archive WDI (H-WDI) estimated from documentary records, which is mediated by contemporaneous perception. The differences and mismatched patterns between the two, therefore, can reflect the perceptual biases behind "historical archives" as a carrier of collective memory.

RC: *Figure 2d: how can there be more than five WDI index categories for the historical archival material?*

AR: The site-level archival records are five ordinal categories; the apparent "more than five" values in Figure 2d arise because the panel shows the regional H-WDI, which is the mean of up to thirty site-level ordinal records and is therefore continuous. We now state this explicitly in the Figure 2 caption.

RC: *Lines 107–109 state that over 0.8 of the historical archive sites were located in areas showing a positive correlation with the WDI series. What exactly does this mean? Does 0.8 refer to 80% of sites, or something else?*

AR: The reviewer is right that the bare "0.8" was ambiguous. We have removed it and now report the spatial agreement as a percentage of sites. In the same revision, we also dropped the $p < 0.1$ threshold (at Reviewer #3's request), so the sentence now reads in terms of the conventional $p < 0.05$ level: **18 of the 30 historical-archive sites (60%)** lie in areas significantly positively correlated with the N-WDI series ($p < 0.05$). The **Figure 2c** annotation and caption already report this same 60% / $p < 0.05$:

> **Manuscript · Page 5, Line 113–114**
>
> Spatially, 18 of the 30 historical-archive sites we used were located in areas that showed a significant positive correlation with the N-WDI series ($p < 0.05$; **Figure 2c**)

RC: *Lines 323–325 appear to form an incomplete sentence.*

AR: This is the same fragment flagged by Reviewer #5. The dangling "Due to poor retention rate … in the early period (1000–1469 CE)." sentence (which had no main clause) now reads:

> **Manuscript · Page 15, Line 343–346**
>
> Due to the poor retention rate (25.17%, measured as the ratio of archived meteorological data to the total quantity; see **Supplementary Information S1**) and obvious source bias (mainly based on official histories, with a relative lack of local and folk materials) in the early period (1000−1469 CE), we begin our analysis in 1470 CE (see **Supplementary Information Figure S2**).

RC: *The figure labels are formatted inconsistently throughout the manuscript. In some figures the panels are marked as "a", while in others they are written as "a.". They are also positioned differently between figures, for example in the right vs left side of the panels.*

AR: We have adjusted the subfigure labels for all figures, but because each figure has a different layout, placing them all in the same position does not seem feasible; we have done our best to make the layout neater.

RC: *In Figure 2, the text "Pearson correlation coefficient" also appears to be somewhat awkwardly positioned.*

AR: Done. In the revised Figure 2 the awkward free-floating "Pearson correlation coefficient" text has been replaced by a clean vertical colour-bar label, "Pearson r (Validation)", placed alongside panel 2c, and the caption now identifies it explicitly.

RC: *My overall assessment is that this is a very exciting and promising manuscript that could become even stronger with some minor revisions.*

AR: We are grateful for this positive overall assessment and for the constructive, focused suggestions. We have addressed each of the points above, and we believe the revised manuscript is clearer and stronger as a result.

1. Papworth, S. k., Rist, J., Coad, L. & Milner-Gulland, E. j. Evidence for shifting baseline syndrome in conservation. *Conserv. Lett.* **2**, 93–100 (2009).
2. Pauly, D. Anecdotes and the shifting baseline syndrome of fisheries. *Trends in Ecology & Evolution* **10**, 430 (1995).
3. Fritsche, I. & Masson, T. Collective climate action: When do people turn into collective environmental agents? *Current Opinion in Psychology* **42**, 114–119 (2021).
4. Soga, M. & Gaston, K. J. Shifting baseline syndrome: Causes, consequences, and implications. *Front. Ecol. Environ.* **16**, 222–230 (2018).
5. Soga, M. & Gaston, K. J. Global synthesis indicates widespread occurrence of shifting baseline syndrome. *BioScience* **74**, 686–694 (2024).
6. Rozin, P. & Royzman, E. B. Negativity bias, negativity dominance, and contagion. *Pers Soc Psychol Rev* **5**, 296–320 (2001).

7. de Guttry, C. & Ratter, B. Expiry date of a disaster: Memory anchoring and the storm surge 1962 in hamburg, germany. *International Journal of Disaster Risk Reduction* **70**, 102719 (2022).
8. O'Connor, P. The unanchored past: Three modes of collective memory. *Mem. Stud.* **15**, 634–649 (2022).
9. Gongaware, T. B. Collective memory anchors: Collective identity and continuity in social movements. *Sociological Focus* **43**, 214–239 (2010).
10. Ge, J. *History of Chinese Population.* (Fudan University Press, Shanghai, 2005).
11. Barclay, G. W., Coale, A. J., Stoto, M. A. & Trussell, T. J. A reassessment of the demography of traditional rural china. *Popul. Index* **42**, 606–635 (1976).
12. Lee, J. Z. & Campbell, C. D. *Fate and Fortune in Rural China: Social Organization and Population Behavior in Liaoning, 1774–1873.* (Cambridge University Press, 1997). doi:10.1017/cbo9780511563058.
13. Lee, J. Z., Feng, W. & Campbell, C. D. Infant and child mortality among the qing nobility: Implications for two types of positive check. *Popul. Stud.* **48**, 395–411 (1994).
14. Campbell, C. D. & Lee, J. Z. Long-term mortality consequences of childhood family context in liaoning, china, 1749–1909. *Soc. Sci. Med.* **68**, 1641–1648 (2009).
15. Telford, T. A. Patching the holes in chinese genealogies: Mortality in the lineage populations of tongcheng county, 1300–1880. *Late Imp. China* **11**, 116–136 (1990).
16. Riley, J. C. *Rising Life Expectancy: A Global History.* (Cambridge University Press, 2001). doi:10.1017/cbo9781316036495.
17. Burke, A. *et al.* The archaeology of climate change: A blueprint for integrating environmental and cultural systems. *Nat Commun* **16**, 5289 (2025).
18. Sherif, M., Taub, D. & Hovland, C. I. Assimilation and contrast effects of anchoring stimuli on judgments. *J. Exp. Psychol.* **55**, 150–155 (1958).
19. Gray, E., Grasso, M., Farrall, S., Jennings, W. & Hay, C. Political socialization, worry about crime and antisocial behaviour: An analysis of age, period and cohort effects. *Br J Criminol* **59**, 435–460 (2019).
20. Fanta, V., Šálek, M. & Sklenicka, P. How long do floods throughout the millennium remain in the collective memory? *Nat Commun* **10**, 1105 (2019).
21. Fang, X. *et al. The Social Impacts of Climate Change in China over the Past 2000 Years.* (Springer Nature, 2024).
22. Roberts, P. *et al.* Uncovering the multibiome environmental and earth system legacies of past human societies. *Annu. Rev. Environ. Resour.* (2024) doi:10.1146/annurev-environ-112321-101257.
23. Song, S., Wang, S., Jiao, C. & Ibarra, E. J. M. ABSESpy: An agent-based modeling framework for social-ecological systems. *J. Open Source Softw.* **9**, 6298 (2024).
24. Cohen, J. A coefficient of agreement for nominal scales. *Educ. Psychol. Meas.* **20**, 37–46 (1960).
25. Yang, Y. *Millennium Regional Extreme Drought and Flood Atlas of China.* (China Cartographic Society, 2024).
26. Liu, Y. *et al.* Recent anthropogenic curtailing of yellow river runoff and sediment load is unprecedented over the past 500 y. *Proc. Natl. Acad. Sci. U.S.A.* **117**, 18251–18257 (2020).
27. Yi, L. *et al.* Reconstructions of annual summer precipitation and temperature in north-central china since 1470 AD based on drought/flood index and tree-ring records. *Climatic Change* **110**, 469–498 (2012).
28. Ljungqvist, F. C. *et al.* Northern hemisphere hydroclimate variability over the past twelve centuries. *Nature* **532**, 94–98 (2016).
29. Hoffman, S. M. Chapter 17 - culture: The crucial factor in hazard, risk, and disaster recovery: The anthropological perspective. in *Hazards, risks, and disasters in society* (eds Shroder, J. F., Collins, A. E., Jones, S., Manyena, B. & Jayawickrama, J.) 289–305 (Academic Press, Boston, 2015). doi:10.1016/B978-0-12-396451-9.00017-2.
30. Garde-Hansen, J., McEwen, L., Holmes, A. & Jones, O. Sustainable flood memory: Remembering as resilience. *Mem. Stud.* **10**, 384–405 (2017).
31. García-Gavilanes, R., Mollgaard, A., Tsvetkova, M. & Yasseri, T. The memory remains: Understanding collective memory in the digital age. *Sci. Adv.* **3**, e1602368 (2017).
32. Candia, C., Jara-Figueroa, C., Rodriguez-Sickert, C., Barabasi, A.-L. & Hidalgo, C. A. The universal decay of collective memory and attention. *Nat. Hum. Behav.* **3**, 82–91 (2019).

Author Response to Reviews of

Archival and palaeoenvironmental documentation of historical extreme events reveals generational amnesia in collective memory

Shuang Song 宋爽, Bo Hu 胡博, David Max Findley, Xutong Wu 武旭同, Wei Liu 刘威, Robert W. Middeke-Conlin, Victor L Caetano-Andrade, Yuda Yang 杨煜达, Shuai Wang 王帅, Jürgen Renn, Patrick Roberts

Nature Communications

RC: *Reviewer Comment*, **AR:** Author Response, Manuscript text

RC: *This is a fascinating and excellent study that brings scientific data, historical data and cultural data in the form of collective memory together in a way that demonstrates how these seemingly very different areas of knowledge can be aligned to make meaningful discoveries. While the model created by the authors offers a valuable way to assess, understand, provide for and even forecast and mitigate responses to extreme climate events, the alignment of this disparate data through the model is also an important achievement. The authors have been diligent not only in their use of this literature but in their broad selection of historical data. The discussion of collective memory is appropriate and is supported by an extensive literature and this paper extends and adds to that literature, providing an unusual and creative perspective and analyzing collective memory in a unique way. By aligning historical data/collective memory with data from the natural world, the authors have created a model that enables the measurement of collective memory itself. In particular, the introduction of the Shifting Base Syndrome casts an innovative light on both collective memory and perceptual bias.*

AR: We are deeply grateful to the reviewer for this generous and insightful summary of our work, and for the exceptionally positive assessment. We are especially gratified that the interdisciplinary integration of scientific, historical, and cultural (collective-memory) data, the alignment of these heterogeneous sources, and the introduction of the Shifting Base Syndrome were seen as innovative and meaningful contributions.

RC: *The research supports the authors claims about perceptual bias as well as the possibility of utilizing their model in assessing extreme climate events. I see no flaws in their methodology and interpretations.*

AR: We sincerely thank the reviewer for the confidence expressed in the soundness of our methodology and interpretations. We greatly appreciate this endorsement. Incorporating the other reviewers' suggestions, we further strengthened the methods and model sections; we hope you will find the approach more convincing.

RC: *My field is Archival Studies where both historical data and collective memory are paramount concerns. This study offers exciting new perspectives and possible applications for greater understandings in both of these areas.*

AR: We are honoured that a scholar of Archival Studies, a field for which historical data and collective memory are central, finds our framework exciting and potentially applicable to these domains. We hope our approach can foster further dialogue across the natural and archival/historical sciences, and we thank the reviewer warmly for this perspective.

RC: *This research is extremely detailed and the authors have carefully described their formulas and model and so it can be replicated.*

AR: We thank the reviewer for recognising the level of detail in our description of the formulas and model, and for noting that the study is reproducible. Reproducibility was a key priority for us, and we are pleased that this goal was achieved.

RC: *While I have accessed the code, this is not my area of expertise and so I have not installed it.*

AR: We thank the reviewer for taking the time to access our code, and we fully understand that installation lies outside the reviewer's area of expertise. To facilitate reproducibility for readers with a computational background, the repository includes step-by-step installation instructions, an environment specification, and runnable scripts that regenerate the main results; the code is openly available and documented in the Code Availability statement. We would be happy to provide any further clarification if helpful.

RC: *This manuscript combines a four-century archival Wet/Dry Index (WDI) for northern China with tree-ring-based hydroclimate reconstructions and an agent-based model (ABM) to argue that Shifting Baseline Syndrome (SBS) imprints generational-scale biases on collective memory of climate extremes. Empirically, the authors show systematic mismatches between a "subjective" archive-based WDI and an "objective" proxy-based WDI, and that re-standardising the proxy series within 20–40-year sliding windows increases rank correlation. An ABM with agents that remember only a finite past is then claimed to reproduce an optimal window of ~30 years. My overall assessment is that the topic and basic idea are interesting and potentially significant, but the current manuscript is far from the level of scientific accuracy and clarity required for publication.*

AR: We thank the reviewer for careful and constructive reviews and for recognising the interest and potential significance of this research. We have taken the comments seriously and substantially strengthened the codebase, analysis and writing. In particular, we: (i) corrected the code issues the reviewer identified. Two of them were merely code formatting or redundancy, and the changes do not affect the results. The other two have a limited impact on the results but does not challenge the core conclusions; (ii) added climate-forcing scenarios for a robustness matrix and a full two-stage global sensitivity analysis (Morris + Sobol) to the ABM; (iii) reframed the ABM as a mechanism-discrimination test of SBS ("Shifting Baseline Syndrome", generational amnesia v.s. collective memory illusion[1]); and (iv) revised the research-question framing, the Monte-Carlo description, the main-text ABM description, and the figures for clarity. The point-by-point responses below detail each change. We are pleased to report that none of the corrections alters our central conclusions and that they also significantly improve the framing of our manuscript and research. We thank you again for your thorough review and hope the new version addresses your concerns.

Major comments

RC: *A central strength of the design is the use of an instrumental validation period (1901–2000 CE) in which the validation comparison is strictly between the integrated natural-proxy WDI and independent gridded precipitation datasets. In that period the subjective archival WDI is not used to construct either series, so collective memory cannot contaminate this particular validation step. Historical archives certainly still exist after 1900, but they are not part of the proxy-based WDI or the instrumental datasets, and in that limited sense they are indeed excluded from the comparison.*

AR: We thank the reviewer for correctly summarising the design of our instrumental validation period and for affirming that collective memory cannot contaminate this validation. The 1901–2000 CE division is a deliberate design that isolates a window during which the proxy can be checked against instruments without archival input. It does not imply that collective memory ceases to operate after 1900.

RC: *However, the main research questions as formulated in the Introduction are not actually answered by the methods applied. The authors state that they "test the degree to which period-specific perception bias influences the formation of historical narratives of extreme climatic events" and that perceptual bias leads to a "generational offset in collective memory". What they measure empirically are (i) discrepancies between categorical WDI assignments in two already-constructed series, (ii) changes in Kendall's τ under sliding-window re-standardisation of the objective WDI, and (iii) the behaviour of a stylised ABM driven by i.i.d. normal climate noise. These are not direct measurements of the "formation of historical narratives" or of a "degree" of bias in narrative construction. The archival WDI is itself a heavily processed regional index, which has already collapsed many qualitative narratives into a single ordinal time series. Without an explicit, operational definition of "formation of narratives" and a corresponding quantitative metric, the strong claims about how narrative formation is influenced by period-specific perception bias are not supported by the current analyses. This could be solved by rewriting the research question.*

AR: We thank the reviewer for these valuable comments. We acknowledge that our approach lacks a direct measure of perceptual bias during the "formation of narratives"; instead, it reflects the offset and improvements in the aggregated Wet/Drought Index between the historical archives (H-WDI) and natural proxies (N-WDI). Therefore, we have rephrased the research question based on the reviewers' suggestions and emphasised more clearly how this difference reflects Shifting Baseline Syndrome (SBS), a perceptual bias highly relevant to collective memory studies. Modifications include:

- We no longer use the misleading phrasing "formation of historical narratives".
- Before introducing the specific research questions, we first supplement the current theoretical discussion of SBS by acknowledging that it is only one of many perceptual biases and by noting that SBS originates from

collective factors. Thus, we clarify that we do not measure perception biases or historical narratives at the individual level, making it reasonable to aggregate existing historical archives (e.g., H-WDI) in this context.

Manuscript · Page 2, Line 57–68

Among the many perceptual biases, Shifting Baseline Syndrome (SBS)[2], also referred to as Environmental Generations Amnesia (Figure 1a)[1], suggests that the decayed memory of environmental changes across generations arises from comparisons to a shifting baseline. The baseline serves as a reference point for comparison and defines what is perceived as normal, based on limited memory and experience[1], and is accordingly associated with cross-generational inconsistencies in environmental perception and with a weakened potential for collective action[3,4]. This inconsistency in collective memory may stem from multiple sources, but collective factors, including generational amnesia or collective illusion, were suggested as the main roots of SBS (Figure 1b)[1]. Generational amnesia occurs when younger generations lack knowledge of past environmental conditions, whereas collective memory illusion arises when shared narratives or expectations distort how past conditions are remembered[1]. A recent study suggests that SBS is widespread across diverse contexts[5]. Nevertheless, long-term evidence remains lacking on whether and how SBS shapes collective memory of environmental change.

- Then, we explicitly attribute the current research gaps directly to discussions of "SBS" in order to verify whether it plays a role in collective memory (represented by historical texts) and how it works:

Manuscript · Page 2, Line 69–73

Therefore, we test two hypotheses: (1) historical archives of climatic extremes show signatures consistent with contemporaneous perceptual bias and (2) this perceptual bias is associated with a generational offset in collective memory relative to natural observations of climatic variation.

- Finally, we explicitly explain how our approach helps fill the gaps at the end of the introduction, with an improved Figure 1 presenting the overall research design:

Manuscript · Page 3, Line 92–98

Re-standardising the natural series within sliding windows of $20-40$ years, roughly one human generation, increases the correlation between the two records, and the ABM reproduces this generational optimum when agents judge climate against their own lived experience (generational amnesia) but not against a shared archive (collective memory illusion). These results indicate that historical archives carry a generational-scale perceptual imprint relevant to how past societies recorded, experienced and adapted to climatic extremes.

RC: *Relatedly, the notion of "degree to which period-specific perception bias influences the formation of historical narratives" lacks a clear sense of direction or observable quantity. It is not specified whether "more formation" should mean more narratives per event, more detailed descriptions, higher probability of recording, or something else. Nor is there any attempt to count or otherwise quantify the number of distinct historical narratives as a function of climate anomalies. In practice, the paper infers perceptual bias solely from the relative alignment of an archive-based index and a proxy-based index. This is a much narrower question than the one formulated in the Introduction, and the framing should be revised accordingly or the analyses significantly expanded.*

AR: After revising the research questions (see above), we also removed overly broad expressions such as "measure degree to..." and "formation of historical narrative". The current statements are now reframed to (1) quantitatively demonstrate that SBS has played a role in collective memory represented by historical records; (2) use an agent-based model (ABM) to suggest the underlying mechanisms of SBS. This reframe is directly reflected in our **Figure 1** embedded above. Thanks to the reviewer, this modification makes the research questions more clearly focused, but it does not narrow the study's significance.

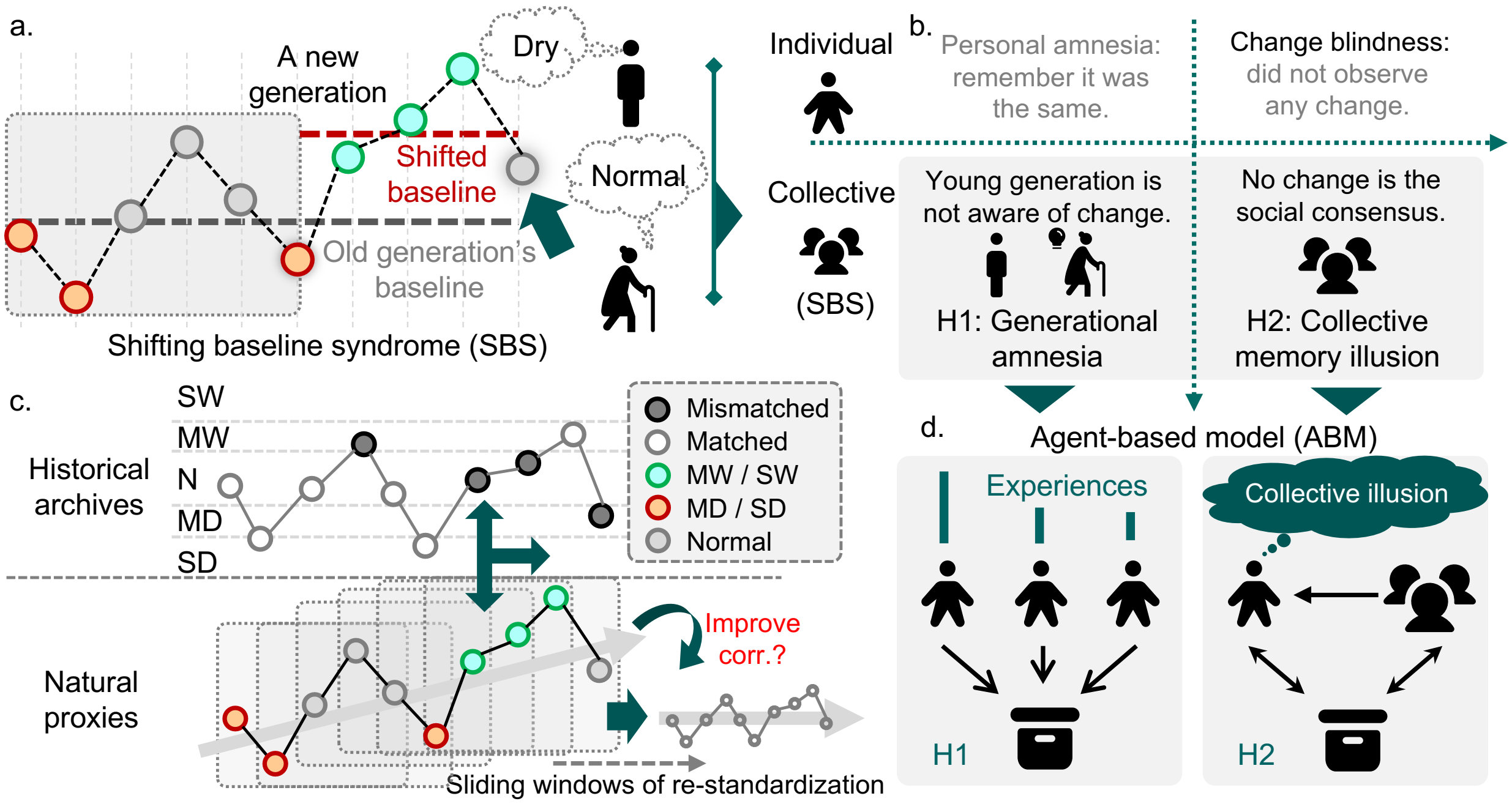

Figure 1: Study design. a. Shifting Baseline Syndrome (SBS): each generation grows up in a different environment and adopts a different reference baseline; in a climate that becomes wetter, a relatively dry year appears normal to earlier generations, whereas a normal year appears dry to later ones. b. Two collective sources of perceptual bias proposed by SBS theory: differences in lived experience across generations (H1: generational amnesia) and a shared, misleading consensus about the past environment (H2: collective memory illusion). c. The two Wet/Dry Index (WDI) series, from historical archives and natural proxies, are each classified by z-score into five levels: Severe Wet (SW), Moderate Wet (MW), Normal (N), Moderate Dry (MD) and Severe Dry (SD) (Supplementary Information Figure S3). We quantify their mismatches and test whether re-standardising the natural series within sliding windows, that is, against a moving baseline, increases their correlation (Methods). d. An agent-based model tests whether agents that judge the climate against their own experience (H1) or against a shared archive (H2) reproduce the observed generational offset.

RC: *The "Monte Carlo approach" used in the mismatch analysis needs clearer definition and more precise presentation. In Methods 4.2.2, the authors describe generating synthetic climate series (random normal) and randomly assigning historical classifications according to observed category frequencies, repeating this 100 times to build a null distribution for the mismatch metric. Methodologically, this is a Monte Carlo randomisation under a specified null model. However, in the main text and Figure 3 caption it is referred to only as a "Monte Carlo approach", without stating what is randomised, what the null hypothesis is, or how many simulations are used. As it stands, this is too imprecise to count as a clearly described Monte Carlo analysis and will be opaque to many readers. The authors should explain in the main text that they perform a Monte Carlo randomisation test, specify the null model (independent random classifications with fixed marginal frequencies), and justify the number of iterations or increase it if necessary.*

AR: We thank the reviewer for this helpful point and agree the test was under-described. We now (i) state explicitly in the main text that significance is established with a Monte Carlo randomisation test, (ii) specify the null model explicitly: in each of 1,000 iterations, archival severity categories are independently redrawn from fixed atlas-level marginal frequencies, continuous natural values are drawn from $N(0,1)$ and classified with the same thresholds as the proxy data, and the bias matrix is recomputed with the same within-grade look-back procedure ($shift = 1,2,3$) as in the empirical analysis. This null preserves the archival marginals and the look-back mechanics but removes any dependence between archival assignments and the natural sequence; cell-wise significance is assessed against the resulting null distribution of bias matrices; and (iii) state the number of iterations is 1000: the previous texts in **Methods** mistakenly reported 100 iterations, whereas the analysis used 1,000; we have corrected accordingly.

- To keep it concise, we summarise the test in a single sentence in the main text and place the full specification in the **Methods** section. The revised main-text sentence now reads:

Manuscript · Page 5, Line 141–144

This shifted-correlation pattern in the historical archives exceeds that expected under a Monte Carlo Randomisation Null (10,000 iterations; see Methods), in which archival severity categories are independently redrawn at fixed marginal frequencies. Five of the eight tested cells reached two-sided significance ($p < 0.05$) at shift = 2 (exact p = 0.0096, 0.012, 0.019, 0.037 and 0.047, not adjusted for multiple comparisons), compared with one cell at shift = 1 ($p = 0.028$) and one at shift = 3 ($p = 0.002$).

- The expanded Methods section now states the null hypothesis explicitly:

Manuscript · Page 18, Line 439–452

We assessed the statistical significance of this bias pattern with a Monte Carlo randomisation test that compares the observed bias matrix against a null model in which archival severity categories are assigned independently of the natural climate sequence. The null hypothesis is that archival classifications depend only on their fixed marginal frequencies $P(H = j) = p_j$ ($\{0.10, 0.25, 0.30, 0.25, 0.10\}$), with no association between archival grades and the natural series. Each iteration generated a synthetic dataset in which natural climate values $x_{\text{sim}} \sim \mathcal{N}(0,1)$ were binned into five severity levels using the same thresholds applied to the proxy data, and archival classifications were independently redrawn from p_j. We ran 10,000 iterations, each with a sample size matching the empirical record length ($n = 431$). For each iteration k, the bias matrix $\Delta^{(k)}$, indexed by natural level i and archival level j, was recomputed with the identical look-back algorithm used on the empirical data (the same shift and within-grade pairing $N_{t'} = N_t$, $t' < t$). This null preserves the archival marginals and the look-back mechanics but destroys any dependence between archival assignments and the natural climate sequence. For each matrix cell (i,j), we computed standardized scores $z_{ij} = (\Delta_{ij}^{\text{obs}} - \mu_{ij}^{\text{null}})/\sigma_{ij}^{\text{null}}$, where μ_{ij}^{null} and $\sigma_{ij}^{\text{null}}$ are the mean and standard deviation of $\Delta_{ij}^{(k)}$ across the 10,000 iterations, and obtained two-tailed p-values as $p_{ij} = 2(1 - \Phi(|z_{ij}|))$, which assumes that the null distribution of each cell is approximately normal. These cell-level p values were not adjusted for multiple comparisons. The null simulations are seeded (random seed 42) so that the reported p values are reproducible.

- The **Figure 3** caption now describes the same randomisation null rather than merely referring to a "Monte Carlo approach".

Manuscript · Figure 3

Significance was assessed by a two-sided Monte Carlo randomisation test (10,000 iterations; Methods), not adjusted for multiple comparisons; filled bars mark $p < 0.05$ (* $p < 0.05$; ** $p < 0.01$). Exact p values (natural to archival level; n, mismatched pairs): c. MD to MW, $p = 0.028$ (n = 10); d. MD to SD, $p = 0.0096$ (n = 2); SD to MD, $p = 0.012$ (n = 1); N to MD, $p = 0.019$ (n = 19); MD to MW, $p = 0.037$ (n = 10); N to MW, $p = 0.047$ (n = 37); e. MD to MW, $p = 0.002$ (n = 10).

RC: *The ABM description in the main text is highly insufficient, even for readers familiar with ABMs. Section 2.4 and Figure 5 give only a schematic overview ("a batch of observers who perceive climatic extremes and may record their perceptions of severity levels", with two baseline options), while all substantive detail is pushed to SI S3. For a general-audience journal, the main text should summarize at least the nature of the climate process, the agents' memory structure, the decision rule for recording, the demographic process, and how the model outputs are converted into a WDI-like series. As written, it is impossible to understand from the main text alone how Figure 5 was produced or in what sense the model "reproduces" the empirical pattern. The description should also make explicit that anchoring and negativity bias, which are emphasised in the SI as theoretical foundations, are actually implemented in the recording propensity and baseline construction; at present, these cognitive mechanisms are not mentioned in the modelling section of the main text, and anchoring appears only later in the Discussion as a general cognitive process, not as something concretely encoded in the ABM.*

AR: We thank the reviewer for this constructive comment and agree that the main-text ABM description was too thin. We have expanded the main text to summarise the model while keeping the Results section concise, moved the full specification to Methods, and made the two cognitive mechanisms explicit and traceable to their implementation in the code.

- We added a compact "model in brief" to the main text (Results, **Section 2.4**) that names the climate process, the agents' memory structure, the recording decision rule, the demographic process, and the conversion of model output into a WDI-like series:

Manuscript · Page 7, Line 168–176

The heuristic ABM represents climate as a stationary AR(1) stochastic process ($c_t = \phi c_{t-1} + \varepsilon_t$, Figure 5a; alternative independent and trended forcings are tested for robustness in Supplementary Note 3 and Figure S9) observed by a continuously turning-over population of finite-lifespan agents. Each agent judges the current anomaly against a baseline, and is more likely to log an event the more it departs from that baseline (Figure 5c). We designed scenarios to represent the baseline, based on lived experience or shared archives of agents, corresponding to the two hypotheses of H1 (generational amnesia) and H2 (collective illusion) (Figure 5c), with two other scenarios tested for robustness (see Supplementary Note 3, Table S11 and Figure S8). The archive contains logged severities from all agents and is aggregated and classified into the same five-level WDI series, so the simulated and empirical records are compared identically (see Methods).

- For the beginning part of the ABM section in **Methods**, we now make explicit that anchoring and negativity bias are concretely encoded:

Manuscript · Page 18, Line 470–476

We further developed an ABM to investigate how collective climate perception can emerge from individual observers' experiences and perceptual biases. The model simulates a population of climate observers who record climatic extremes based on two core cognitive mechanisms: (1) more abnormal extremes are more likely to be recorded, implementing a negativity bias[6] in the recording propensity (the probability p_t defined below); and (2) observers assess the severity of an extreme relative to a memory anchor[7] (the standardised-deviation perception $z_{i,t}$ defined below). We test two scenarios (limited experience or collective archives) and expect similar patterns to emerge between the synthesised N-WDI and H-WDI and the real-world datasets.

- We also state explicitly the model architecture in the main text, including the nature of the climate process, the agents' memory structure, the decision rule for recording, the demographic process, and how the model outputs are converted into a WDI-like series:

Manuscript

Model Architecture
The climate forcing c_t is generated by a stationary stochastic process over a simulation period of several centuries. In the main analysis it follows an AR(1) autocorrelated process, $c_t = \phi c_{t-1} + \varepsilon_t$, with climate persistence $\phi = 0.5$ and innovations scaled to climate variability $\sigma_c = 1$. Independent Gaussian noise ($\phi = 0$) and linear-trend variants are evaluated for robustness (see Supplementary Note 3 and Figure S9). The model maintains a global archive $\mathcal{A}_t = \{e_{t,1}, e_{t,2}, \dots, e_{t,n_t}\}$ recording all extreme events documented at time t, where n_t represents the number of active observers capable of recording events. Climate observers are implemented as N_{agent} autonomous agents characterized by finite lifespans, where typically $L_{\min} = 10$ and $L_{\max} = 40$ years represent their start and end ages for recording. Each observer maintains memory capacity implemented $M_i(t) = \{c_{t-L_i+1}, c_{t-L_i+2}, ..., c_t\}$ constraining itself to recall experiences of climate. Climate perception operates through a standardized deviation calculation relative to the agent's reference baseline (personal or collective; see below). For agent i at time t, the perceived climate anomaly is $z_{i,t} = \frac{c_t - \mu_i(t)}{\sigma_i(t)}$; where $\mu_i(t)$ and $\sigma_i(t)$ are the mean and standard deviation of the baseline that agent i is referring to. The setup of baselines is shown in the section of experimental settings below.
According to a probability p_t, the observer may record its perception in severity of climatic extreme s_i^t at each time step t, so that the global archive is the average judgment from all observers $e_t = \frac{1}{n}(\sum_i^n s_i), n \leq N_{\text{agent}}$. Not every recorded judgement enters the public archive: each event is retained only with probability $1 - \lambda$ and is otherwise forgotten (the archive loss rate λ, default 0.4). The probability p_t is estimated by $p_t = f_0 + 0.5 - S(|z_{i,t}|)$, where f_0 represents baseline recording probability typically set to 0.1, and $S(\cdot) = 1 - \Phi(\cdot)$ denotes the survival function of the standard normal distribution (scale 1). This formulation ensures that extreme deviations approach a maximum recording probability of $f_0 + 0.5$ (0.6 by default), while normal years maintain low but non-zero recording likelihood (see Supplementary Note 3 for details). The per-year aggregate record e_t is then discretised into the same five ordinal WDI categories used for the archives (using the identical standardised thresholds applied to the empirical data), yielding a simulated archival series. This series and the underlying climate c_t are compared using the same sliding-window re-standardisation with Kendall's τ used in the empirical analysis. The model maintains demographic stability through continuous population turnover. At each time step, N_{new} agents enter the population while agents exceeding maximum lifespan are removed, thus creating overlapping generational cohorts with varying memory spans. Model outputs are produced only after the number of observers stabilises at the maximum N_{agent}.

- In what sense the model "reproduces" the pattern is now stated in the **Results** paragraph:

Manuscript · Page 7, Line 176–181

Reproducing the empirical observation measured in historical archives, the correlations between recorded climate by agents and the input climate forcing show the unimodal pattern at an intermediate generational re-standardisation window under the scenario of H1 (generational amnesia) (Figure 5b, red and black lines). In contrast, the H2 (collective illusion) scenario shows a monotonically increasing trend with window length and no generational optimum (Figure 5b, blue line).

RC: *In the ABM code, the z-score formula is wrong due to operation precedence:* `climate - baseline/std` *should be* `(climate - baseline)/std`*. This must completely change the results.*

AR: We are grateful to the reviewer for catching this. It was a genuine bug in the observer's perception/standardisation step, and we have corrected it to the properly parenthesised. Fortunately, the fix does not change too much:

- The previous error occurred only in the modelling part and did not affect the preceding quantitative analysis.

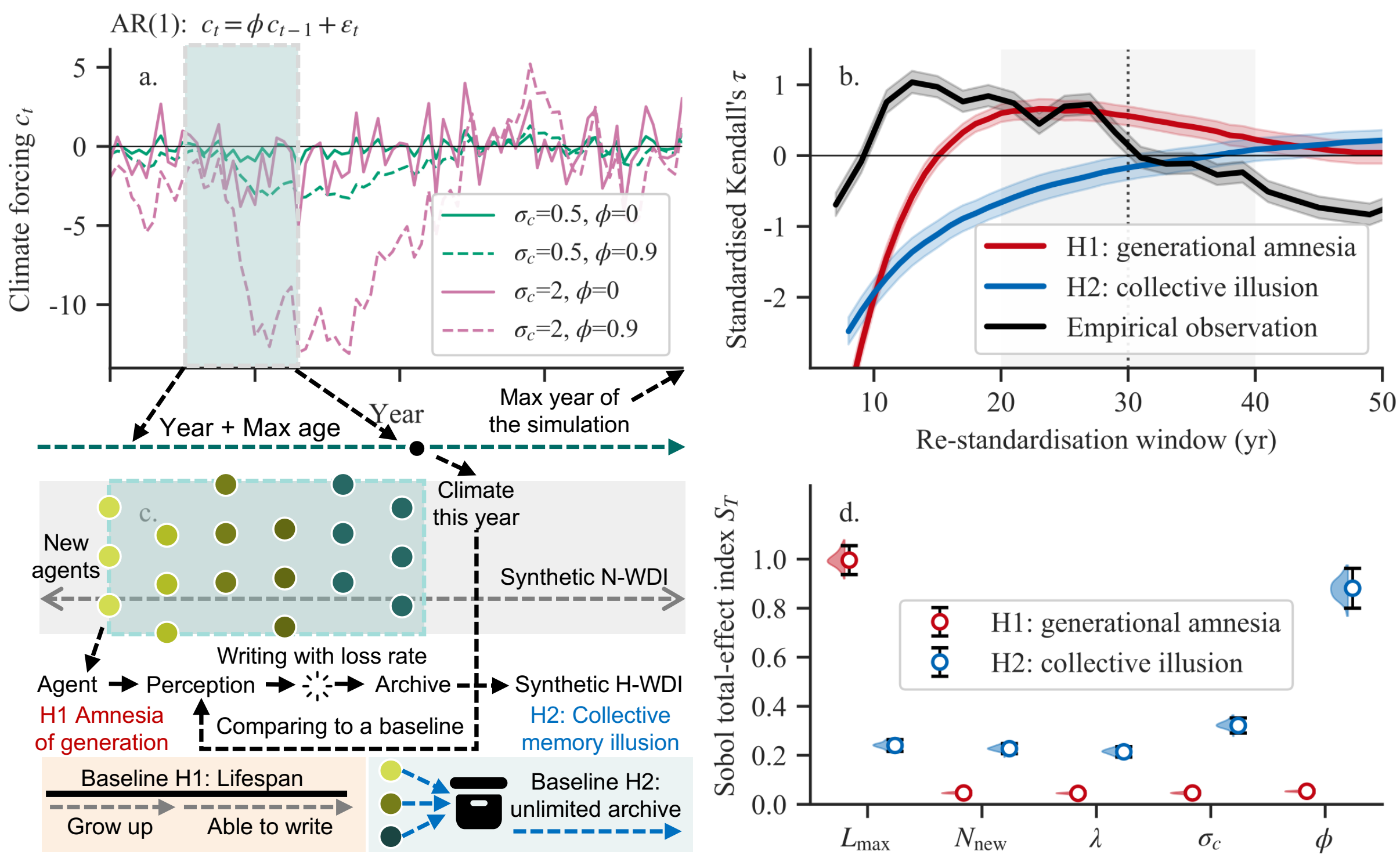

Figure 5: The agent-based model (ABM) and its results. a. Annual climate forcing c_t generated by an AR(1) process ($c_t = \phi c_{t-1} + \varepsilon_t$), shown for the four corners of the Sobol parameter range (colour, climate variability σ_c; line style, climate persistence ϕ); independent and linear-trend forcings are tested in Supplementary Note 3. The shaded box marks the lifespan of one agent cohort, detailed in c. b. Kendall's τ between the simulated archive (synthetic H-WDI) and the climate forcing (synthetic N-WDI) as a function of the re-standardisation window under H1 (generational amnesia, red) and H2 (collective memory illusion, blue), compared with the empirical H-WDI versus N-WDI curve (black). Each curve is standardised by its own mean and standard deviation across windows so that their shapes can be compared. Lines, mean of n = 100 model runs per scenario (empirical curve: n = 100 stochastic realisations of the H-WDI, excluding correlations with $p \geq 0.05$); bands, 95% bootstrap confidence interval of the mean (1,000 resamples). Grey shading, 20–40-year generational band; dotted line, 30 years. c. Model structure. New agents enter every year, can write records from adulthood until their maximum lifespan, perceive each year's climate relative to a baseline, and may write it into a shared archive that loses records at a fixed rate. The baseline is the agent's own lived experience under H1 and the whole shared archive under H2. d. Sobol total-effect index S_T of the location of the optimal window for lifespan L_{max}, new agents per year N_{new}, archive loss rate λ, climate variability σ_c and climate persistence ϕ. Half-violins, distribution of the n = 100 bootstrap estimates of S_T; open circles and error bars, the estimate and its 95% bootstrap confidence interval; N = 1,024 base samples per scenario (7,168 parameter sets, each averaged over 30 stochastic runs).

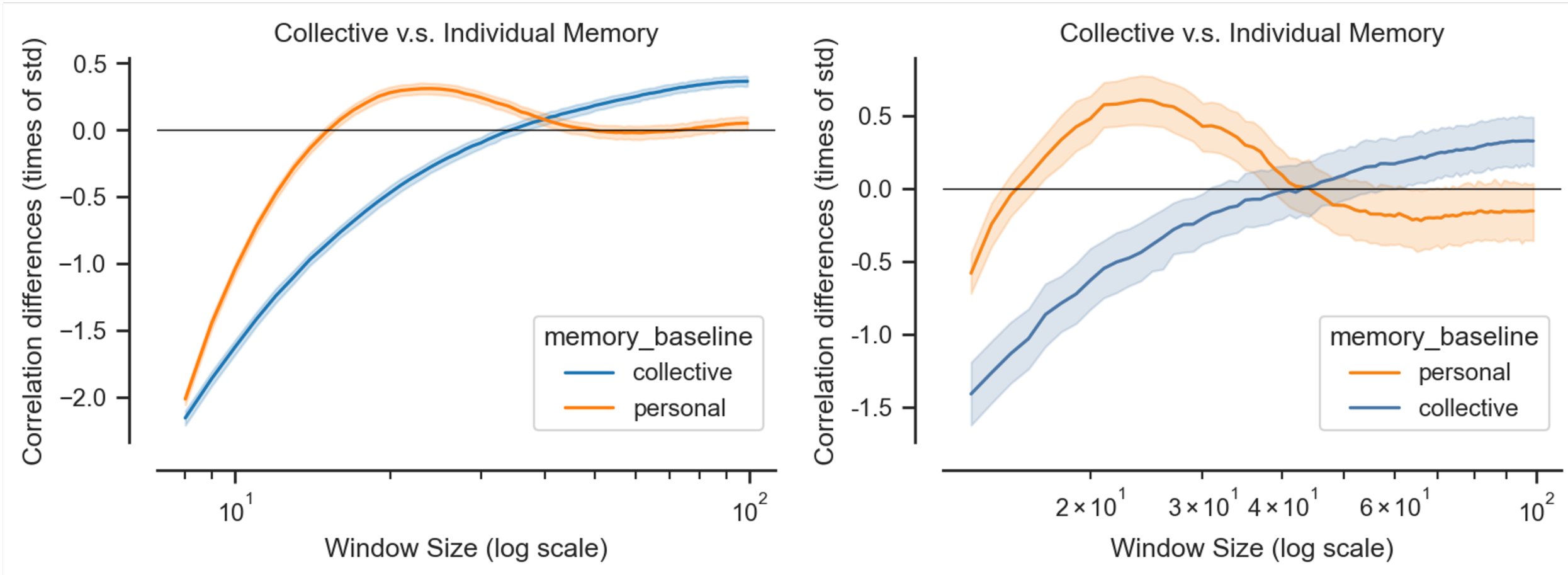

Figure R1: Code-bug comparison. The left panel shows the old figure under the wrong z-score algorithm, while the right panel shows the corrected figure. Note that both figures use the original `idd` climate model for direct comparison of the impact of this bug, while the new manuscript already uses the AR (1) climate forcing model.

- The error had escaped our checks because both variables were already standardised (z-scores), so the mis-grouped expression still produced numerically plausible values rather than visibly anomalous ones and was therefore overlooked. After the correction, the error/uncertainty band widens, but the previously reported pattern persists and remains statistically significant. We have re-run the affected figures with the corrected code. The differences are limited:

Response figure (code-bug comparison) R1.

RC: *The temporal and climatic structure of the ABM needs stronger justification and exploration. Time advances in annual ticks and the climate series is generated as N(0,1) at each step. The annual timestep is chosen to match the yearly WDI, which is reasonable, but it ignores trends and persistence that are likely present in actual climate. The authors then interpret the ~30-year optimal sliding window emerging from this model as supporting a generational SBS mechanism. Given the simplistic climate process, the untested sensitivity of the model output to various assumption, this claim is fragile. The model's results may be highly sensitive to the arbitrary timestep (e.g. if the model ran at seasonal resolution, the effective memory window in years could change), there is a peculiar age structure, no population growth, and no trend or autocorrelation in weather. The statement that "collective climate records aligned with the continuous climate" is also misleading: in the model, climate is not a continuous physical process but independent Gaussian noise. At minimum, the authors should justify the annual step in terms of the empirical WDI and discuss whether and how alternative temporal resolutions or realistic climate processes (with trend and persistence) would affect the emergent memory window.*

AR: Thank you for raising the concern about robustness of the climate forcings. We have addressed this directly with a climate-forcing robustness matrix. The ABM now supports three forcing processes: i.i.d. Gaussian white noise, AR(1) persistence, and a linear trend with noise, at two temporal resolutions (annual, and sub-annual at four steps per year aggregated back to annual values, so all peak-window statistics remain in yearly units). Changes have been made accordingly:

- As mentioned above, our main text uses experiments under the AR(1) climate forcing, which also allows us to examine the sensitivity of two parameters in the ABM:

> **Manuscript · Page 19, Line 478–480**
>
> The climate forcing c_t is generated by a stationary stochastic process over a simulation period of several centuries. In the main analysis it follows an AR(1) autocorrelated process, $c_t = \phi c_{t-1} + \varepsilon_t$, with climate persistence $\phi = 0.5$ and innovations scaled to climate variability $\sigma_c = 1$.

Manuscript · Page 20, Line 521–525

We also conduct a global, variance-based Sobol sensitivity analysis (Saltelli sampling, $N = 1024$ base samples, each parameter vector evaluated over 30 stochastic repeats), reporting both first-order (S_1) and total-effect (S_T) indices over five parameters: (1) maximum lifespan $L_{\max}$, (2) the number of new agents per year N_{new}, (3) the archive loss rate λ, (4) climate variability σ_c and (5) climate persistence ϕ, to identify which factors govern the location of the generational optimum.

- The main text's **Figure 5** already shows that under an AR(1) climate forcing (closer to real climate), "H1 Generational amnesia" still produces the pattern with an optimal window and is insensitive to climate parameters. The other scenario, "H2 collective illusion," is sensitive to climate parameters but cannot reproduce the patterns observed in empirical data.

Manuscript · Page 10, Line 181–187

Global (Sobol) sensitivity analysis identifies which of the five parameters (max age, number of new agents, archive loss rate, climate persistence, and variability; Figure 5d) account for this divergence. Under the scenario H1, the location of the optimal window is dominated by an agent's lifespan (total-effect index $S_T = 1.00 \pm 0.06$, 95% bootstrap confidence interval; Figure 5d) and is directly proportional to it (see Supplementary Information Figure S7). In contrast, if one assumes that agents use the shared archive as the baseline (H2), the simulations did not reproduce the above pattern within the tested parameter ranges (Figure 5d), including when the length of archive access was varied (Supplementary Information Table S11 and Figure S8).

- This recurrent pattern of ABM under the "H1 generational amnesia" scenario was also tested under different climate scenarios, and the results were stable. Across all six configurations (10 replicates each), the emergent optimal window remains within the ~20–40-year generational band, confirming the result is not an artefact of the previous i.i.d. assumption, of climate persistence/trend, or of the annual timestep:

RC: *Demographic assumptions in the ABM also warrant more careful treatment. The model introduces a fixed number of new agents each year and removes agents at maximum age, with no dependence of births or deaths on current population size. This produces a flat age distribution between the minimum and maximum ages. Because each agent's memory window and thus their baseline length depends on age, the resulting collective memory is strongly shaped by this artificial age structure, which does not resemble actual historical demography. The authors link their empirical 20– 40-year sliding window to "the generational life expectancy of humans in ancient China", yet the default model uses a maximum age of 40 years (in the SI) or 60 years (in the main Methods) and a recruitment process that is not tied to any empirical demographic data. They report an ANOVA-based sensitivity analysis on a limited set of parameters, for which they don't report effect sizes, but this is not a global sensitivity analysis in the usual sense: there is no Sobol or Morris-type decomposition to quantify how much of the variance in the key output (the location and magnitude of the correlation peak) is attributable to each parameter and to interactions. Without such an analysis, it is hard to know whether the emergence of a ~30-year optimum is robust or depends delicately on a particular combination of lifespan, recruitment, loss rate, and climate variance, beyond all the remaining structural assumptions. I would strongly recommend either performing a basic global sensitivity analysis or, at minimum, substantially expanding the current sensitivity study and clearly describing how parameters are sampled and how their influence on the outputs is quantified.*

AR: Thank you to the reviewer for this comment, which significantly enhances the robustness of our method. We have now followed your recommendation and made improvements in three aspects: (1) using a more comprehensive sensitivity analysis; (2) explicitly linking demographic parameters to actual historical data; and (3) noting that, as we use it as a heuristic model, we do not intend to exactly copy historical population structures.

- We replaced the ANOVA with a full global sensitivity analysis (SALib: Morris screening followed by a Sobol variance decomposition with bootstrap confidence intervals), run independently for all scenarios over five parameters (**Figure 5d**). According to the results, for the H1 scenario that replicates the empirical data patterns, population life expectancy (max age of agents) is the most influential (almost dominant) factor.

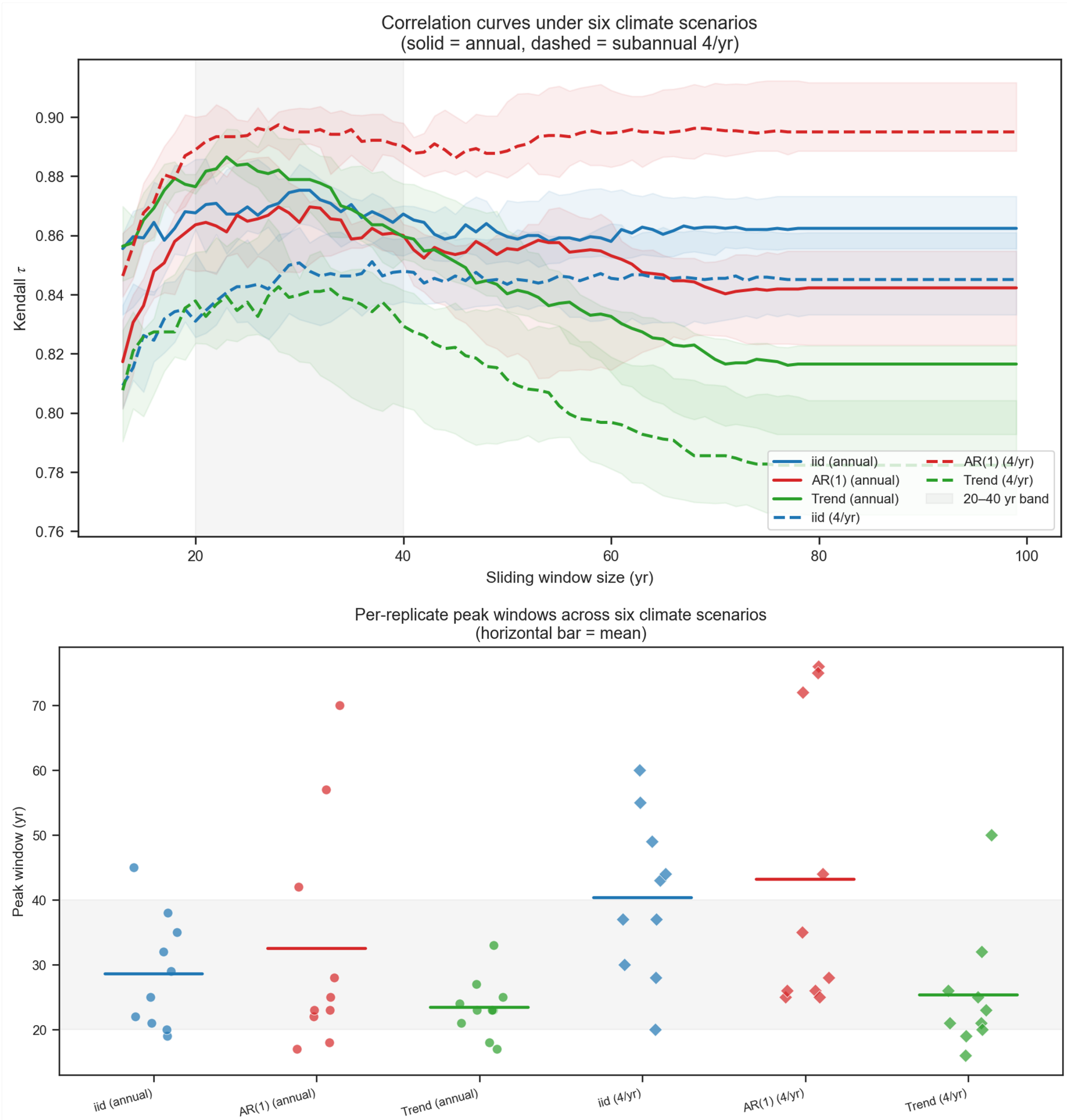

Figure S9: Climate-forcing robustness matrix for the H1 (personal baseline) scenario. Top: window−τ correlation curves for six forcing scenarios —three stochastic processes (i.i.d., AR(1), trend-plus-noise) at two temporal resolutions (solid = annual; dashed = sub-annual, four steps per year aggregated to annual units); the shaded band marks the 20–40-year generational range. Bottom: per-replicate peak (optimal) windows for the same six scenarios (points, individual runs; horizontal bars, means; $n = 10$ runs per scenario). Across every forcing the peak windows cluster within the generational band, confirming that the emergent generational optimum does not depend on the choice of climate process or temporal resolution.

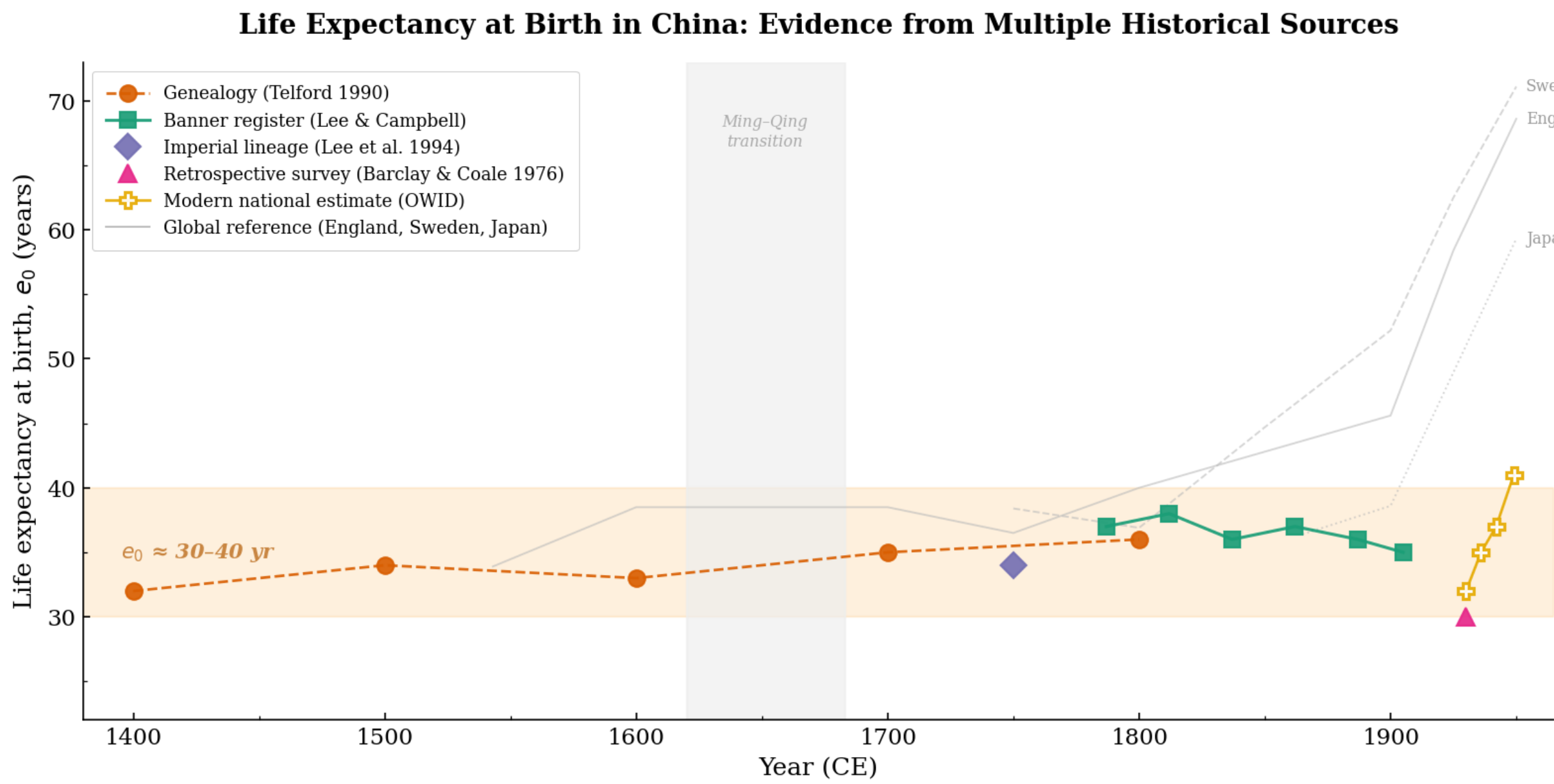

Figure S6: Life expectancy at birth (e_0) in historical China compiled from multiple independent historical sources (genealogies, banner registers, imperial-lineage and retrospective-survey estimates), with pre-industrial reference series for England, Sweden, and Japan shown in grey. Chinese estimates remain within $e_0 \approx 30-40$ years (shaded band) across 1400−1900 CE, consistent with the 20−40-year generational window recovered from the archives and with the ABM's maximum lifespan of 40 years.

- We reviewed existing results discussing past human life expectancy. In the **supplementary information**, we include a graph to demonstrate that our chosen parameter value, `max_age = 40`, is reasonable and that the expected lifespan it implies makes the optimal window we observe interpretable. Regarding this, we also corrected the inconsistency and apologise for the previous typo of 60 in the main text.

- Lastly, as we note above, reproducing exact changes in population structure was not our aim. In this paper, we focus more on historical documents as representations of collective memory, and on the fact that those who produce such collective memory do not represent the entire population. In fact, we conjecture (without finding direct evidence) that the time distribution of literate elites capable of writing would indeed be close to flat when the total population does not change much. For example, this is because the number of positions available at court to record history did not vary greatly (for compiling official histories), nor did the number of people in a given region increase (for compiling local gazetteers and diaries). We explicitly disclose this in the discussion of limitations:

Manuscript · Page 12, Line 265–269

Finally, the ABM is a deliberately minimal, heuristic model rather than a demographic reconstruction. This is mainly because the groups that produced the historical archives do not represent the entire population, and our global sensitivity analysis (Figure 5d) shows that their life expectancy matters. We have confirmed the key parameter in this simplified demography is consistent with population data we have collected (Supplementary Information Figure S6).

RC: *More generally, the connection between the ABM and the data feels ad hoc. From the SI it is clear that the model is conceptually grounded in cognitive psychology—negativity bias and anchoring, and in SBS, but in the main text the modelling section is framed almost exclusively in terms of SBS, without stating which psychological mechanisms are actually encoded. There is no attempt to calibrate the model to the empirical WDI series, nor any formal model-data comparison beyond the qualitative observation that both exhibit an optimal window of ~30 years, which is arguable*

given the log scale. Alternative mechanisms that could also generate a similar sliding-window optimum (e.g. purely statistical effects of autocorrelation or of the rolling standardisation itself or the average age of agents) are not explored. As a result, the current ABM functions more as an non-convincing toy model than as a genuine explanatory or testing framework; the link to the empirical pattern needs to be made more rigorous.

AR: Thank you to the reviewer for raising these concerns and these excellent points. We fully agree with your doubts about the expressed value and role of the ABM in previous versions. As you pointed out, we previously linked SBS to other cognitive psychology theories (e.g. negativity bias and anchoring), which stemmed from existing literature offering many cognition-rooted alternative explanations for the emergence of the SBS phenomenon[1,8,9]. After re-organising the research questions following your suggestion (see above responses), we refocused the research on SBS and reframed the ABM as a test of two competing hypotheses that could produce SBS. We also added a more comprehensive sensitivity analysis to strengthen the model's explanatory power. Specific revisions include:

1. Using the memory baseline as the lever for hypothesis testing. Under the exact same model, adopting a personal versus a collective memory baseline yields qualitatively different features: only the "H1: generational amnesia" produces the "correlated rise-then-fall" pattern, while the "H2 collective illusion" does not. The two mechanisms produce non-overlapping peak-window distributions, and only the H1 matches the empirical pattern.
2. The discussion showing two SBS-related conjectures does not conflict with deeper cognitive-psychological explanations, because they operate at different explanatory levels. For example, "negative bias" and "anchoring effects" operate at the individual level and explain why we form different baselines. In addition, when such baseline shifts occur, we show that SBS tends toward generational amnesia (H1) rather than collective illusion (H2) at the collective level.
3. Confirming via sensitivity analysis that the generational amnesia (H1) scenario, age dominates the results according to the Sobol analysis. Under the H2, instead, the window is more driven by climate_phi (ST ≈ 0.90). As for the max age of the agents under the H1 scenario, we also semi-quantitatively linked it to historical archives in the above revisions.
4. We no longer use a confusing logarithmic axis to show figures.

Accordingly, we now clearly position the ABM as a mechanism-discrimination test, a heuristic model, to detect which memory baseline is consistent with the empirical pattern, rather than a calibrated simulation. We hope the reviewer feels that this role and its connections are well reflected in the revised manuscript and the responses above.

RC: *The figures require substantial revision for clarity and scientific precision. Figure 1 combines three conceptually distinct elements (a study-area map, a conceptual SBS diagram, and a data-processing schematic—into a single figure). For readability it would be preferable to separate these or at least more clearly delineate them. In Figure 2a, the validation period (1901–2000 CE) appears to have larger fluctuations in the integrated proxy WDI than earlier periods; this noticeable change is not explained. The caption's phrase "the grey area indicates the uncertainties" is vague; the authors should specify whether this represents, for example, the 5–95% posterior credible interval or whatever else. Panel 2c includes a colour label that is not clearly identified in the caption (I assume it's the title of the plot). Panel 2d asserts that both WDI series "follow a normal distribution", but no normality test is reported; unless a formal test is performed and described, this should be phrased differently. Figure 3's legend seems unnecessary, panels c–f are not clearly labelled in the figure, the "validation period" shows up in panel f but I don't think it even belongs ther, and again the "Monte Carlo approach" is mentioned without any explanation. In Figure 4, plotting legends on top of error bars makes the legend hard to see. Figure 5's correlation-versus-window-size curves for the ABM are not interpretable without reading SI S3; the main text should explain how many runs were averaged, how events were discretized into levels, and how the curves relate to the observational analysis. At present, one effectively needs the SI to make sense of any of the modelling results, which is too heavy a dependence.*

AR: We thank the reviewer for these detailed figure comments and have revised the figures and captions accordingly. The figures have been reorganised, and every flagged panel has been corrected.

- For the **Figure 1**, we separated the three previously combined elements. The study area map is now a standalone figure in Supplementary Information (**Figure S1**). The conceptual SBS diagram and the data-processing schematic are consolidated into **Figure 1**, which now also includes the ABM design schematic.

- **Larger validation-period fluctuations (former Figure 2a).** We checked this, and what appears only in the 1901 – 2000 CE window is the overlaid instrumental validation series (the red lines). This is the well-known "variance attenuation of tree-ring reconstructions": calibration regression retains only the explained fraction of instrumental variance, so high-frequency weather variance is dampened in the proxy. We now explicitly mention this in the main text rather than leaving it unexplained:

Manuscript · Page 5, Line 108–110

The instrumental validation series (thin red line shown only for 1901–2000 CE) has a greater amplitude than the N-WDI, which reflects the variance attenuation inherent to tree-ring reconstructions (calibration regression damps high-frequency variance, retaining only the fraction explained).

- **"The grey area indicates the uncertainties" (former Figure 2a caption).** We now define the band explicitly as the 5 – 95% posterior credible interval of the Bayesian-integrated index (annual posterior mean ± 1.645 posterior SD). We also corrected the figure so that the band matches this definition:

Manuscript · Figure 2

Thin grey line, annual posterior mean; black line, 30-year low-pass filter; grey band, posterior mean $\pm$ 1.645 posterior standard deviations (approximate 90% credible interval).

- **Panel 2c colour label.** This is the colour bar of the spatial correlation map. In the revised figure, it is a clean vertical colour bar labelled "Pearson r (Validation)" and the caption now iexplicitly identifies it
- **Panel 2d "follow a normal distribution".** We rephrased the sentence in the caption to:

Manuscript · Figure 2

Distributions of the N-WDI (n = 431 years) and H-WDI (n = 208 years) over the pre-instrumental period (two-sided Shapiro–Wilk test: N-WDI $W = 0.998$, $p = 0.812$; H-WDI $W = 0.982$, $p = 0.008$).

- **Mismatch figure (Figure 3): legend, panel labels, validation panel, Monte Carlo.** We removed the redundant legend and added explicit panel labels (c)–(f) to the figure. The Monte Carlo test is now fully defined (see our response to the earlier comment and the revised Methods and caption). We would note, however, that the existence of the panel "validation period" was correct. It was there to compare patterns that only appear in historical archives. We are sorry for the unclear annotation, which may have caused this confusion. We have now explicitly added this in the methods and the panel's caption:

Manuscript · Page 5, Line 144–146

Between the reconstructed and the instrumental N-WDI during the validation period (from 1901 to 2000 CE), only one cell reached $p < 0.05$ at shift $= 1$ (Moderate Dry to Moderate Wet, n = 1 pair, two-sided $p < 0.001$), and its offset has the opposite sign to the pattern in the historical archives (Figure 3f and also Supplementary Information Figure S11).

Manuscript · Figure 3

f. The same analysis for the reconstructed versus instrumental N-WDI over the validation period (1901–2000 CE, n = 100 years, shift $= 1$); the only cell with $p < 0.05$ (MD to MW, n = 1 pair, $p < 0.001$) has the opposite sign (Supplementary Information Figure S11).

- **Sliding-window figure (Figure 4): legend over error bars.** The legend has been repositioned to avoid overlap with the error bars.
- **ABM figure interpretability (Figure 5).** As detailed in our response to the previous comment, we redesigned this Figure. The caption now states how many replicates were averaged, how the model output is interpreted, and how the simulated curves relate to the observational analysis. We have aimed to make the modelling figure interpretable from the main text and **Methods** without the SI.

RC: *Finally, there are a few conceptual and numerical inconsistencies that should be resolved. In Section 2.3 the improvement in correlation after re-standardisation is described as "ranging from 0.1 to 0.2", while a few lines later the average optimal window size is said to yield a "maximum correlation increase of 0.12". From SI Table S6 it appears that 0.1–0.2 refers to the range across different proxy-integration and validation scenarios, and 0.12 is the maximum increase for the main pure-natural scenario averaged across temporal segments. This should be stated explicitly to avoid the impression of contradiction.*

AR: We thank the reviewer for raising this concern. The reviewer's understanding is entirely correct. We apologise for not having made this clearer in the manuscript before. We have now specified the increase in relevance for the main scenario in the text only as a percentage, and no longer referring to robustness checks of supplementary information as the range. We have made the following revisions to the wording:

Manuscript · Page 7, Line 152–158

Compared to the benchmark scenario without restandardisation (the whole-period Kendall's $\tau = 0.19$ between the H-WDI and the N-WDI before any re-standardisation; two-sided $p < 0.001$, n = 208 years), a greater correlation gradually emerges as the tested window size w increased to around 18 years, from 1470 to 1900 CE (maximum increased by 5.37%, Figure 4b). Across the different proxy-integration and validation scenarios we tested, the correlation improvements from restandardisation persist regardless of the method used to combine the composite datasets of N-WDI (see Supplementary Note 2, Figure S5 and Table S6)

Minor comments

RC: *For clarity throughout the manuscript, it would be helpful to introduce explicit labels for the two WDI series, such as O-WDI for the objective proxy-based index and S-WDI for the subjective archival index, and use these consistently in text and figures. This would make it much easier for readers to track which series is being transformed, validated, or used as a baseline.*

AR: We thank the reviewer for this practical suggestion and have adopted it, with one adjustment to the naming. Rather than O-WDI/S-WDI (objective/subjective), we introduce the neutral, descriptive pair H-WDI (historical-archive) and N-WDI (natural-proxy), which are used consistently throughout the text and figures. We made this choice so that the labels simultaneously resolve a concern raised by Reviewer #6 that an "objective vs subjective" framing is reductive, since the tree-ring series is itself an interpreted proxy.

RC: *In Table S2, the coordinates are given as raw numbers; the authors should add N/S/E/W designations (e.g. 35.18°N, 103.73°E) to conform to standard geographic notation. In SI 1.4, the statement that self-comparison materials "may account for more than 10%" is described as a "subjective estimation"; the basis for this figure is unclear. If it is from an actual tally on a defined corpus, the method should be described; if not, a precise percentage should be avoided and replaced by a qualitative statement. In Table S3, the 1690 CE translation includes "releasing over a hundred dragons". If this is here a metaphor for floodwaters or water spirits, this should be clarified; otherwise, the current phrasing may mislead readers.*

AR: Thank you to the reviewer for raising this point. We have made the formatting changes in the latest **Supplementary Information**, including modifying latitude and longitude formats, removing imprecise percentages, and deleting unnecessary metaphorical references.

RC: *In SI S3 section 1.2, memory is described as "implemented as a deque storing recent climate experiences"; for non-programmer readers, it would be clearer to say explicitly that this is a first-in-first-out (FIFO) queue of past climate values up to the agent's memory capacity.*

AR: We thank the reviewer for this point and have rephrased this for non-programmer readers. The **Supplementary**

Note 3 (ODD+D protocol) now describes the memory explicitly as a first-in–first-out (FIFO) queue rather than a "deque":

> **Supplementary Information · Page 20, Line 299–301**
>
> a bounded personal memory implemented as a "first-in–first-out (FIFO)" queue storing recent climate experiences up to the current age, a minimum age for recording eligibility (default 10 years), and a maximum lifespan (default 40 years).

RC: *The last sentence before SI Section 2 ("using a standard-deviation filter applied to the right-hand series") is opaque; "right-hand series" should be replaced with a clear description such as "applied to the climate time series used as the objective reference" or similar. Likewise, the phrase that parameters "come from experience" in section 2.1 should be clarified: presumably the authors mean that parameter values are chosen based on expert judgment and not fitted to the data.*

AR: We agree with both points and have revised the wording of the **Supplementary Information**.

- We replaced the opaque "right-hand series" with an explicit description of which series the filter is applied to:

> **Supplementary Information · Page 21, Line 328–330**
>
> After completion, the model computes rolling-window correlations between the collective series and the underlying climate using a standard-deviation filter applied to the natural climate series used as the objective reference.

- We clarified that the parameters are set by expert judgment rather than fitted:

> **Supplementary Information · Page 23, Line 371–373**
>
> This empirical phenomenon inspires the model, but no parameters are derived from data analysis. Therefore, conceptually, this is a heuristic model intended to analyse potential mechanisms of SBS.

RC: *In SI section 2.4 the authors suddenly mention three possible baselines (personal memory, full model climate series, and collective archive) whereas earlier sections describe only two operational settings (personal vs collective). It should be clearly stated which baselines are actually used in the analyses and which are only conceptual possibilities. In 2.6 they write that "in its current configuration, the archive primarily serves as a tool for analysis and does not influence individual decisions", which appears to conflict with earlier references to agents comparing to collective records; this ambiguity should be resolved. In 2.7, the last sentence about "prospective extensions with re-judgment" is difficult to understand and could be rephrased.*

AR: Sorry for the confusion. We have corrected these three points accordingly:

- In the main text, we used only two baseline scenarios to avoid confusing readers. But in robustness testing, we also tested more. We have only detailed the results of the other experiments now in the **Supplementary Information (Robustness Analyses)**.
- We removed the confusing statement "in its current configuration, ⋯" and listed the interactions between agents and the archive by scenario.
- We rewrote the previously hard-to-understand sentence in the ODD+D protocol (**Supplementary Note 3**) like this:

Supplementary Information · Page 23, Line 367–371

Only the diagonal H1/H2 contrast is foregrounded in the main text; `collective_lifetime` and `model` are reported here as decomposition/benchmark controls, and their full correlation profiles are presented with the robustness analyses below (Figure S8). The empirical basis for the model is derived from analyses of historical climate records from northern China (1470–1900 CE), tree-ring reconstructions (validated for 1901–2000 CE), and the psychological literature on cognitive biases in environmental perception (see main texts).

RC: *The rationale for representing ordinal severity levels via truncated-normal sampling to produce continuous values should be explained. Since the empirical analyses use ordinal categories and Kendall's τ, it is not obvious why a continuous mapping is needed for the ABM outputs; at least, the authors should argue that this choice does not materially affect the correlation patterns. In 2.9, record transmission loss is said to occur with probability loss_rate "(default 0.20, uniform distribution)"; this is not a uniform distribution but a Bernoulli process with parameter 0.2, and the terminology should be corrected. The choice of maximum age 40 in the default configuration also needs a brief justification in light of the discussion of generational life expectancy.*

AR: We thank the reviewer and have addressed all three points.

- **Rationale for the truncated-normal mapping.** In any given year, several observers (agents in the ABM) or several archival sites (in the empirical data) may record different ordinal levels, and averaging raw integer codes into a single yearly/regional value has no natural metric. We therefore map each level to a continuous value drawn within its z-score interval and average. This is the same procedure used for the empirical H-WDI, so the ABM output and the archives are processed identically rather than by using two different conventions. The downstream statistic is Kendall's τ, a rank correlation, so the mapping affects only the absolute magnitudes assigned to levels and the breaking of ties among equal level-averages —not the cross-year rank order that, together with the sliding re-standardisation window, produces the window-dependence:

Manuscript · Page 15, Line 363–375

Generally, concrete determinations of floods or droughts, along with descriptions of severe societal outcomes of disasters, are classified at the Severe level (SD or SW), while alone records of natural conditions (such as heavy rains or prolonged droughts) are classified at the Moderate level (MD or MW). In contrast, a normal (N) year refers to records containing keywords such as harvest, abundant year, ripe, and bountiful, or non-abnormal events in the current year, with continuous records available in the preceding or following years (Supplementary Information Table S3 provides more examples). To integrate archives across the study area, site-year ordinal grades were first converted to continuous z-scores, because ordinal levels cannot be averaged meaningfully. Each grade maps to a z-interval bounded by the same thresholds used for WDI classification (rather than a single point, see Table 1). It means, for the site i and year t, we drew 100 values from a truncated normal within that interval and used their mean as the site-level continuous WDI, $h_{i,t}$. The regional H-WDI was then $H_t = \text{mean}(\{h_{i,t}\})$ over all sites with non-missing records in the year t ($n_t \leq 32$). Sites without archival evidence were excluded rather than imputed, and years with no reporting sites were left missing. This stochastic mapping is a tie-breaking convenience for cross-site averaging.

Numerical confirmation: we reran the entire window–τ analysis, replacing stochastic sampling with a deterministic midpoint mapping ({−1.5, −0.5, 0, 0.5, 1.5}). The peak (optimal) re-standardisation window is **identical** under the two schemes, the profiles correlate at $r \geq 0.99$, and $|\Delta\tau| \leq 0.03$ at every window, for both the empirical H-WDI/N-WDI comparison and the ABM collective archive. The two curves differ only by a small, near-constant vertical offset (≤ 0.03), which is expected and immaterial: the deterministic midpoints and the truncated-normal interval means assigning slightly different magnitudes to each level (e.g. ±1.66 vs ±1.50 for the extreme level). When the continuous series is re-classified against fixed thresholds (the empirical pipeline), sharper sampling magnitudes yield marginally higher τ, whereas in the direct continuous ABM comparison, the single-draw sampling noise marginally attenuates τ. In neither case does the offset shift the location of the correlation peak—the quantity that carries our conclusion (a generational, ~20–40-year optimum).

- **"uniform distribution" → Bernoulli.** Corrected: record transmission loss is a Bernoulli process, not a

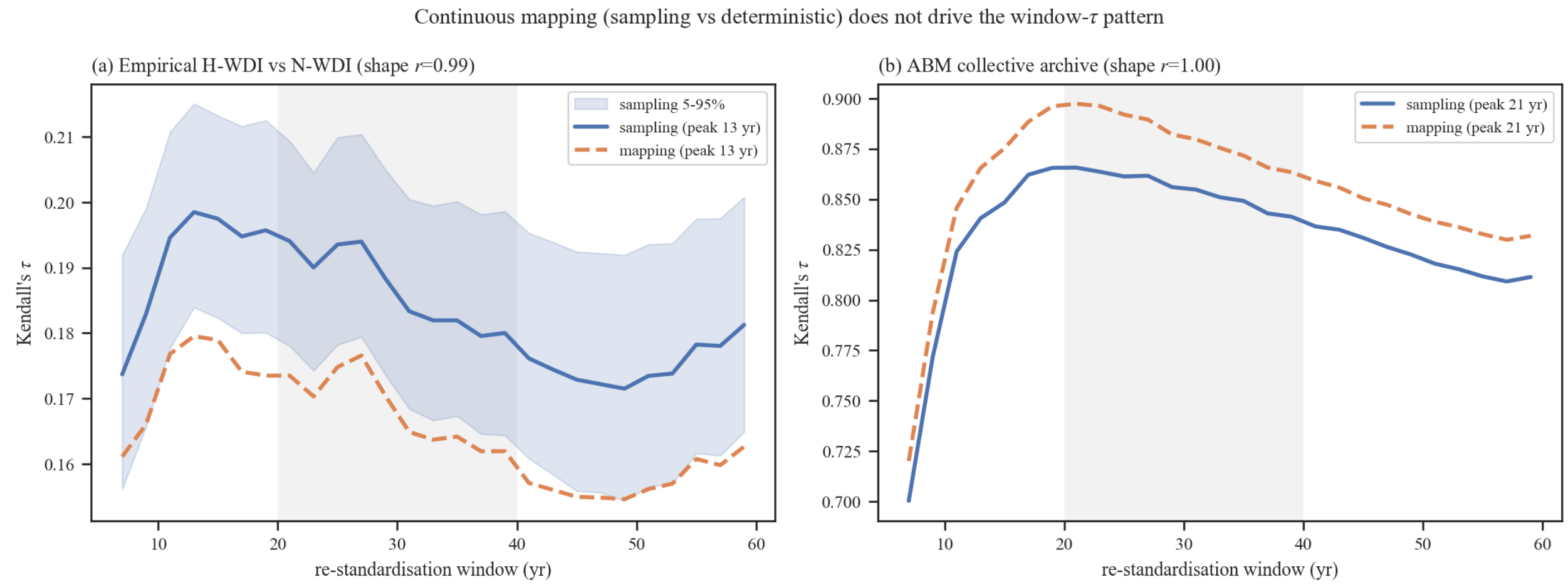

Figure R2: Numerically confirm that different sampling methods do not affect the pattern of the results. Left: sampling methods' impacts on empirical optimal sliding window size; right: impacts on the ABM's results.

uniform distribution. Additionally, this parameter actually takes the value 0.4 in the ABM (the previous 0.2 was a typo). Therefore, the SI now reads:

Supplementary Information · Page 23, Line 378–381

Agents make decisions through a two-stage process: first, computing a z-score indicating how the current climate deviates from their personal memory; then, drawing a Bernoulli trial with probability determined by a survival function that assigns higher likelihoods to extreme deviations.

- **Justification for `max_age = 40`.** We added a brief justification tying it to generational life expectancy and noted that it is not load-bearing, with a data-based figure earlier to illustrate that this parameter is reasonable:

Supplementary Information · Page 20, Line 314–319

The default `max_age = 40` approximates the adult recording lifespan of an observer in this historical context (roughly the working/adult span of a generation) and is consistent both with the life expectancy at birth estimated for historical China[10] ($e_0 \approx 30-40$ years; based on multiple independent sources[11–16], including Genealogy, Banner register, Imperial lineage, Retrospective survey, and global references for pre-industrial values in England, Sweden, and Japan (Figure S6)). Because the sensitivity analysis varies `max_age` over 15–80 years, the emergent optimal window scales with it.

RC: *In compare.py the correlation defaults likely underconstrain rolling windows, since `min(log2(n),2)` is almost always <=2.*

AR: The reviewer is right about this incorrect default value. We corrected the default from `min(log2(n), 2)` to `max(int(log2(n)), 2)` and added regression tests. The manuscript's **Figure 4c** sweep already passes an explicit `min_periods`, so its main optimum result is unchanged. The fix mainly corrects the library default for analyses that rely on `compare_corr` without an explicit `min_periods`.

RC: *It seems like the confusion matrix orientation is not consistent, since you transpose, then the index and column names seem flipped, but I may have missed something here.*

AR: The reviewer is right about the labels. The transpose is deliberate (so the matrix matches the downstream plotting and mismatch-analysis access pattern, rows = predicted/H, columns = true/N), but the `index.name`/`columns.name` metadata had been set the wrong way around. We corrected the axis names to `pred`/`true` (in both `_compute_confusion_matrix`

and the `false_count_matrix` property) and added a docstring explaining the transpose. The matrix **values** are byte-identical, and only the (previously swapped) axis-name metadata changed.

RC: *The reindexing of the matrix in calibration.py is happening at the level of the local variable (matrix) not applied to the matrix. So, in the end nothing is happening. Unless you are using a different version when this behavior is not the same. (Remarks on code availability)*

AR: Thanks for raising the concern. Reassigning the loop variable did not rebind the underlying `DataFrames`, so the reindex was confusing dead code. We fixed it to explicitly rebind both matrices. The dormant bug had no numerical effect because pandas' label-aligned arithmetic in the subsequent z-score step already padded to the full category axes. We verified that the resulting p-value matrix is identical before and after the fix.

RC: *I skimmed the code briefly and there seems to be at least one key problem. See report. (Remarks on code availability)*

AR: We thank the reviewer for the code-level scrutiny. The key problem flagged in the report is the ABM z-score operator-precedence bug, which we have fixed (see the dedicated response above, with a comparable figure). We have also resolved every other code issue raised and appreciate your careful review!

RC: *This paper was a pleasure to read. In the abstract, the paper's purpose is clearly stated and articulated in three steps: - A mismatch is shown between historical and instrumental observations; - The mismatch is mitigated by generation-sized sliding windows; - An ABM model reproduces this periodicity.*

AR: We sincerely thank the reviewer for the kind words and for the clear, accurate summary of our three-step argument. We are delighted that the paper's structure came across as intended.

RC: *The paper, in the view of these reviewers, by any means deserves publication. There are, however, some improvements that we think could improve its readability.*

AR: We are very grateful for this positive overall assessment and for the constructive, readability-focused suggestions. We have worked through each point below and revised the manuscript accordingly to improve clarity and flow.

RC: *From a theoretical standpoint, the paper appears conceptually robust. The Shifting Baseline Syndrome framework is highly compelling, and the attempt to reproduce its underlying mechanisms through an ABM is both coherent and methodologically promising. Furthermore, this approach seems potentially transferable to other domains (e.g., the perception of crime or urban disorder, where what is considered "normal" may also shift across generations). This represents a significant strength of the paper. The authors could give their opinion on transferability, and potentially add other examples to this regard.*

AR: We thank the reviewer for this generous assessment and for the excellent suggestion. We agree the mechanism transfers wherever the baseline against which "normal" is judged is itself reconstructed each generation, and we have made this explicit in the Discussion, adding the reviewer's own cross-domain example:

> **Manuscript · Page 13, Line 271–275**
>
> The implications of our findings extend beyond historical climatology. Our results suggest that perceptual biases can operate consistently over long timescales, and that the cognitive mechanisms of SBS identified in contemporary contexts[5,17] have deep historical roots and potentially broad applicability. Evidence of SBS and generational amnesia has been discussed across diverse fields, including public perceptions of urban social incidents[18], settlement in flood-prone areas[19], and assessments of the historical condition of degraded ecosystems[4].

RC: *It may also be worthwhile to further problematize the nature of the historical archives. These sources may not simply reflect aggregated individual perceptions, but could also be understood as institutional products shaped by administrative conventions and relatively stable classificatory criteria. Clarifying whether possible effects of administrative practices or evolving classification standards over time have been considered would strengthen the argument. Moreover, this issue connects to the 30 year threshold, within which the correlation between historical records and climatic proxy data appears to improve. While this window size produces the strongest statistical alignment, it does not necessarily imply that individuals retain a generational memory of exactly thirty years.*

AR: This is a valuable point, and we agree on both counts: (i) the archives are partly institutional products shaped by administrative conventions and classification standards, not purely aggregated individual perceptions, and (ii) the ~30-year window marks the scale of strongest statistical alignment and should not be read as a literal "exactly thirty years" of individual memory. We have added the discussion in the corresponding part of the main text:

- We first acknowledge that institutions are also important factors in shaping historical archives in our discussion of research limitations.

Manuscript · Page 12, Line 243–244

Several limitations warrant consideration. First, historical archives, shaped by institutions, also incorporate social responses that modulate the societal impact in addition to individual perceptions[20].

- Then, the fact of consistently improved correlation between historical and natural datasets across time indicates that different institutions don't affect the robustness of our analysis:

Manuscript · Page 12, Line 249–255

Moreover, our consideration of temporal slices addresses issues of changing political context, with similar results across two major Chinese dynasties (Supplementary Note 1 and Figure S2), suggesting the robustness of our analysis under different institutions (Figure 4c). For example, around the time of the Emperor Wanli (万历, 1573-1620 CE), a period of prosperity turned to decline for the Ming Dynasty and far fewer fiscal resources were allocated to disaster relief than from 1649 to 1849 CE, when the emperors in the Qing Dynasty established a complex disaster response system. Nevertheless, the unimodal pattern was also observed in both periods (Figure 4c).

- To explain that the optimal window is about 30 years, we state more directly in the discussion that this is not fixed but is directly linked to population life expectancy.

Manuscript · Page 11, Line 220–227

Our optimal sliding window sizes (ranging from 20 to 40 years) are also approximately equal to the generational life expectancy of humans in ancient North China[10] (see Supplementary Information Figure S6). This pattern and its peak in correlation improvements can be reproduced by a heuristic ABM under the assumption of generational amnesia, but not under the assumption of collective memory illusion (Figure 5). This implies that the emergence of SBS is consistent with biased perceptions shaped by members of the same generation through their experiences, rather than the illusion of shared narratives. Further sensitivity analyses also show that the agents' life expectancy is the dominant parameter in this scenario, which is sufficient to reproduce the empirical pattern (Supplementary Information Figure S7).

- We also added a comparative chart of population life expectancy in the supplementary information, along with newly added sensitivity experiments that have strengthened this association and support the generational amnesia hypothesis.

RC: *Yet, accuracy of data and coherence of statements should have been more carefully checked; it's really unsettling to see Kendall's tau jump between 0.17 and 0.19 in different parts of the text.*

AR: We apologise for the apparent inconsistency. We audited every reported Kendall's τ and confirmed that they constitute a single, internally consistent set. The "jumps" came from quoting three different comparisons without labelling them. We have now labelled each occurrence explicitly in the main text: $\boldsymbol{\tau} = \mathbf{0.176}$ is the agreement between the H-WDI and N-WDI **categories** over the pre-instrumental period (**Figure 3a**); $\tau = 0.214$ is the agreement between

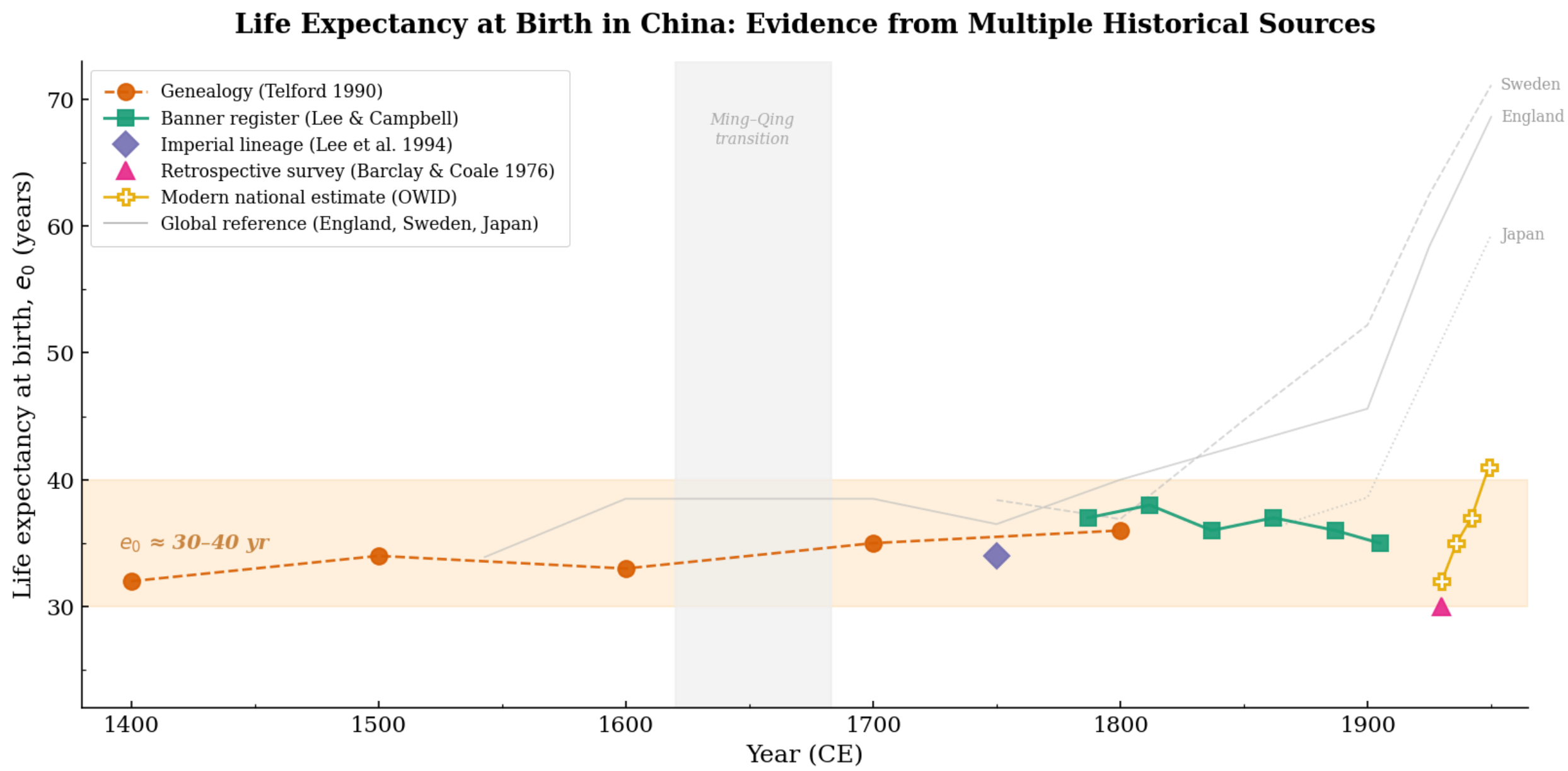

Figure S6: Life expectancy at birth (e_0) in historical China compiled from multiple independent historical sources (genealogies, banner registers, imperial-lineage and retrospective-survey estimates), with pre-industrial reference series for England, Sweden, and Japan shown in grey. Chinese estimates remain within $e_0 \approx 30-40$ years (shaded band) across 1400−1900 CE, consistent with the 20−40-year generational window recovered from the archives and with the ABM's maximum lifespan of 40 years.

the N-WDI and the independent instrumental series over the validation period (Figure 2a); and $\boldsymbol{\tau}$ = **0.190** is the whole-period benchmark between the unmodified N-WDI (anomaly rather than categories) and H-WDI, before any re-standardisation. The first two are near-identical, which is exactly the "comparable agreement" point we make in Section 2.2; the third differs because it is a distinct whole-period, non-re-standardised benchmark. With these labels, the three values no longer read as a single quantity that "jumps". We have now retained three decimal places, which makes the differences clearer. For reference, the three values now appear in the text as:

Manuscript · Page 5, Line 124–130

During the pre-instrumental period (1470 to 1900 CE), the categorised H-WDI shows modest but significant agreement with the categorised N-WDI (Cohen's weighted κ = 0.2, 95% bootstrap confidence interval 0.07−0.33, two-sided permutation p = 0.004; classification accuracy = 0.39), with a slight but significant positive rank correlation between the two categorical series (Kendall's τ = 0.176 for H-WDI versus N-WDI, 95% bootstrap confidence interval 0.054−0.293, two-sided p = 0.004; n = 208 years; Figure 3a). This level of agreement is lower than, but of the same order as, that achieved by the proxy itself against independent instruments over the validation period (1901−2000 CE), where the N-WDI and the instrumental series agree at Kendall's τ = 0.214 (95% bootstrap confidence interval 0.063−0.361, two-sided p = 0.012; Cohen's weighted κ = 0.24, 95% bootstrap confidence interval 0.08−0.38, two-sided permutation p = 0.009; n = 100 years; see Supplementary Note 4 and Figure S11)

Manuscript · Page 7, Line 152–158

Compared to the benchmark scenario without restandardisation (the whole-period Kendall's τ = 0.19 between the H-WDI and the N-WDI before any re-standardisation; two-sided $p < 0.001$, n = 208 years), a greater correlation gradually emerges as the tested window size w increased to around 18 years, from 1470 to 1900 CE (maximum increased by 5.37%, Figure 4b). Across the different proxy-integration and validation scenarios we tested, the correlation improvements from restandardisation persist regardless of the method used to combine

the composite datasets of N-WDI (see Supplementary Note 2, Figure S5 and Table S6)

RC: *The ABM is presented as a "simple but robust ABM approach" and we agree to that. This usage of ABM sets an example of clever application of the methodology. Having already shown that a generational sliding window improves correlation, the authors use the ABM to reinforce the result and propose an explicit causal mechanism that puts their thesis to work.*

AR: We thank the reviewer warmly for this endorsement of our modelling strategy. We have further strengthened the ABM in revision (a climate-forcing robustness matrix and a two-stage global sensitivity analysis), reinforcing exactly the "simple but robust" character the reviewer highlights. As we stated in our response to Reviewer 2 regarding the "disconnect" between the ABM and the empirical analysis, we explicitly position it as a "heuristic model" for hypothesis testing.

Manuscript · Page 10, Line 187–189

This heuristic model suggests that generational amnesia (assuming individuals rely on their lived experience) is sufficient to generate generational inconsistencies in the model, whereas collective illusion did not reproduce the observed mismatch pattern in the empirical WDIs.

RC: *While the statement that the paper is, "as an essential step in addressing the challenges of the Anthropocene," might sound, and be, slightly exaggerated, we agree that the paper constitutes an exemplary way of "combining empirical data from archives and paleo-environmental proxies with computational modelling". This sets an example of good practice for the field, that we hope will find diffused application. In the words of the authors, "co-evolutionary models being developed to study human-Earth system interaction should find a way to incorporate systematic data on the role of collective actions in adaptations and extremes."*

AR: We take the point and have toned down the "essential step in addressing the challenges of the Anthropocene" phrasing to a more measured claim, while retaining the framing the reviewer endorses (combining archival/paleo-environmental data with computational modelling). We emphasise more on our framework's transferability:

Manuscript · Page 13, Line 289–292

Moreover, our methodology provides a transferable framework for studying long-term collective memory dynamics by combining empirical data from archives and paleo-environmental proxies with computational modelling. It illustrates how slow variables (such as collective memory[21]) can be studied on co-evolution between humans and the environment during the Anthropocene[22].

RC: *The ABM code is available, and documented in ODD, as all ABMs should. Development of the ABM has been made with the use of a platform (https://pypi.org/project/abses/) that remarkably improves replicability.*

AR: We thank the reviewer for recognising the ODD documentation and the use of the `abses` package[23], and for valuing reproducibility as we do. In the new version, we further strengthened the description of the ABM, including sensitivity analyses and a more detailed presentation of the methodological foundations. The code for the new version is also openly available.

RC: *In the following, we remark in a more punctual way on the state of the paper.*

AR: We address each of the following specific remarks in turn below.

2 Results

RC: *The validation period is not mentioned in the introduction and appears quite abruptly, thus making it a surprise for the reader, disrupting the flow.*

AR: Agreed. Thanks for your valuable suggestion. We include this important first step in the last paragraph of the introduction now:

Manuscript · Page 3, Line 88–98

Here, we show that mismatches between archival and natural records of climatic extremes in the Yellow River Basin follow a systematic pattern: archival severity is over- or underestimated when a year is only slightly wetter or drier than comparable earlier years. Re-standardising the natural series within sliding windows of 20 − 40 years, roughly one human generation, increases the correlation between the two records, and the ABM reproduces this generational optimum when agents judge climate against their own lived experience (generational amnesia) but not against a shared archive (collective memory illusion). These results indicate that historical archives carry a generational-scale perceptual imprint relevant to how past societies recorded, experienced and adapted to climatic extremes.

RC: *In this section, the p-tests don't seem to have been preregistered, and in the opinion of this reviewer p<0.1 should not be mentioned.*

AR: Agreed. The analyses were not preregistered, and we have removed every reference to the $p < 0.1$ threshold, reporting significance only at conventional levels (now * = $p < 0.05$, ** = $p < 0.01$). So, Figure 3 and every other significance-annotated panel re-render without the $p < 0.1$ tier; the Methods Monte-Carlo passage and the **Figure 3** caption were updated to match as well.

RC: *In section 2.2, if we understand it, tau is 1.18 for the data, 1.17 for validation. In section 2.3, tau is 1.19, same as in the relative figure. Are these different calculations, and why?*

AR: Sorry for the confusion we caused. We have now distinguished the values explicitly in the text. In 2.2, the two τ are different comparisons: $\boldsymbol{\tau}$ **= 0.176** is H-WDI vs N-WDI levels (the archive-vs-proxy agreement, pre-instrumental) and $\tau = 0.214$ is N-WDI vs the instrumental series (the proxy validation, 1901–2000). In §2.3, $\boldsymbol{\tau}$ **= 0.190** is the whole-period benchmark between the unmodified N-WDI and H-WDI *before* re-standardisation (the value also shown as the baseline in Figure 4b); Each is now labelled correctly where it appears.

Methods

RC: *Apart from some typo here and there, the style of presentation can be improved. The reader gets absolutely nothing from the proxy equation. As a rule of thumb, no equations should be in a paper that don't get used later on. Same goes for the C(x) equation that can be better summarized in a table. For Cohen, it appears that, when you state that you have used the quadratic one, you give (and never use again) the linear one.*

AR: We thank the reviewer and have adopted these suggestions.

- **C(x) ⟶ table.** The piecewise $C(x)$ definition is now a table (category, symbol, standardised range), which is easier to read. Combining with a suggestion from another reviewer, we also include an archival example to differentiate the levels of severity (Table 1 in the revised manuscript):

Table 1: Five severity levels of the Wet/Dry Index (WDI) and representative archival examples.

Level	Abb.	Z-score	Archive example	Translation
Severe dry	SD	$\leq -1.17\sigma$	旱荒，发赈账济，全活甚众。	Due to the drought and famine, relief funds were distributed widely, saving many lives.
Moderate dry	MD	$[-1.17\sigma, -0.33\sigma]$	夏大旱，至八月始雨，禾尽稿	In the summer, there was a great drought; it did not rain until August, and all the grain withered.
Normal	N	$[-0.33\sigma, 0.33\sigma]$	大稔	Great harvest.
Moderate wet	MW	$[0.33\sigma, 1.17\sigma]$	九月葵未，诏顺天、保定、河间、永平四府水涝伤稼	In September, accumulated water soaked the grain. Yongding, Baoding, and Hejian prefectures were flooded.
Severe wet	SW	$\geq 1.17\sigma$	夏，汉江水涨，漂没庐舍甚众	In summer, the Han River flooded, sweeping away many households.

- **Cohen's κ.** We confirmed there is no quadratic/linear inconsistency in the revised text: the Methods state

Cohen's *quadratic-weighted* κ and describe a penalty proportional to the squared category distance and no unused linear form remains.

Manuscript · Page 17, Line 412–415

Statistical validation of classification agreement was done employing Cohen's weighted Kappa coefficient[24] with quadratic weighting ($\kappa_w = \frac{p_o - p_e}{1 - p_e}$), where, p_o represents observed agreement and p_e denotes expected agreement under random classification

- **The proxy/integration equation.** We removed the displayed equation. The Bayesian integration described is retained and is now explained in plain language, including why a simple per-year average is not appropriate (see our response to Reviewer #5 on the integration).

RC: *What is with the values in the C definition? The 10% threshold of the normal is at -1.28, not 1.17.*

AR: The reviewer is right that −1.28σ is the exact 10% quantile of the standard normal. Our thresholds are not theoretical normal quantiles, and we have clarified this in the Methods. The five-grade scheme follows the WDI convention of the source atlas[25]: the grades are defined to reach target frequencies of about 10% for the most severe dry and wet grades, and the thresholds were set empirically from the observed frequency distribution of summer precipitation, which is only approximately Gaussian. The value ±1.17σ is therefore the empirical boundary that reproduces the preset frequency of about 10%, and it does not contradict the theoretical ∓1.28σ, since the two answer different questions. The realized cumulative probabilities at our thresholds are {0.121, 0.371, 0.629, 0.879}, giving frequencies of about 12/25/26/25/12%. We keep the thresholds and the nominal percentages unchanged, and no results change.

Manuscript

···the standardized thresholds [−1.17σ, −0.33σ, 0.33σ, 1.17σ] are empirical boundaries rather than theoretical normal quantiles: the Atlas compilers derived them from the observed frequency distribution of summer (May–September) precipitation so that the resulting grade frequencies match the preset targets (about 10% for the most severe dry and wet grades). Because precipitation is approximately but not exactly Gaussian, this empirical ±1.17σ differs slightly from the theoretical ±1.28σ that would bound the exact 10% normal quantile.

RC: *Small typos, missing verbs and other minor comments can be found in the attached PDF. They must be understood just as suggestions, or pointers to places where small improvement can be made. (Remarks on code availability)*

AR: We are grateful for the detailed annotations in the attached PDF and have worked through them.

RC: *The code looks reproducible and usable.*

AR: We thank the reviewer for this confirmation. We have additionally added tests alongside the four code fixes made in response to the other reviewers, further safeguarding reproducibility.

RC: *I co-reviewed this manuscript with one of the reviewers who provided the listed reports. This is part of the Nature Communications initiative to facilitate training in peer review and to provide appropriate recognition for Early Career Researchers who co-review manuscripts. (Remarks on code availability)*

AR: We thank the co-reviewer for their time and contribution, and we welcome Nature Communications' initiative to recognise the work of Early Career Researchers in peer review. No specific change is requested in this note.

RC: *This article is quite fascinating. As someone who works with anecdotal evidence of effects of extremes on ecosystems I have long wondered about shifting baseline syndrome, and was excited to see this empirical test. It seems to me very novel and clever in the way it brings together historical records with climate proxies.*

AR: We are delighted that the reviewer, with first-hand experience of shifting baselines in ecosystems, found the empirical test novel and compelling. We thank them warmly for this encouraging assessment!

RC: *However, at present the work is extremely difficult to follow. Both methods and results are quite opaque. It would be good to make the work more broadly accessible with much clearer explanations and figures. Most of my suggestions below are aimed at improving the clarity of the presentation.*

AR: We take this comment seriously and have prioritised clarity in the revision. We have (1) introduced consistent notation for the two WDI series (N-WDI v.s. H-WDI); (2) expanded all figure captions with more details of the method; (3) moved key derivations and archival examples into a clearer form; (4) and rewritten the most opaque methods (especially the mismatch analysis) and corresponding results. The point-by-point responses below detail each change.

RC: *Given that the detailed methods are at the end of the paper, the introduction and results sections need to give some more information about the approach taken.*

AR: Agreed. This has been addressed in the revision.

- The final paragraph of the Introduction now lays out the three-step approach before the detailed (end-placed) Methods:

> **Manuscript · Page 3, Line 88–98**
>
> Here, we show that mismatches between archival and natural records of climatic extremes in the Yellow River Basin follow a systematic pattern: archival severity is over- or underestimated when a year is only slightly wetter or drier than comparable earlier years. Re-standardising the natural series within sliding windows of 20 − 40 years, roughly one human generation, increases the correlation between the two records, and the ABM reproduces this generational optimum when agents judge climate against their own lived experience (generational amnesia) but not against a shared archive (collective memory illusion). These results indicate that historical archives carry a generational-scale perceptual imprint relevant to how past societies recorded, experienced and adapted to climatic extremes.

- The Results are made self-contained through the substantially expanded figure captions and a compact introduction paragraph for the ABM's architecture, so a reader can follow the logic without first turning to the Methods. For two examples:
- (1) How is the " shifted-correlation pattern" detected:

> **Manuscript · Page 5, Line 133–137**
>
> To further explore the process of comparing to the past implied by the SBS theory, we compared each mismatched H-WDI data with a similar prior event which occurred once, twice, or three times (shift $= 1, 2, 3$, respectively) in the past (Figure 3b). In these mismatch cases, the H-WDI is usually categorised as drier (or wetter) than N-WDI when the z-score values of N-WDI are only slightly lower (or higher) compared to a former year.

- (2) A brief ABM architecture overview in the results:

> **Manuscript · Page 7, Line 168–176**
>
> The heuristic ABM represents climate as a stationary AR(1) stochastic process ($c_t = \phi c_{t-1} + \varepsilon_t$, Figure 5a; alternative independent and trended forcings are tested for robustness in Supplementary Note 3 and Figure S9) observed by a continuously turning-over population of finite-lifespan agents. Each agent judges the current anomaly against a baseline, and is more likely to log an event the more it departs from that baseline (Figure 5c). We designed scenarios to represent the baseline, based on lived experience or shared archives of agents, corresponding to the two hypotheses of H1 (generational amnesia) and H2 (collective illusion) (Figure 5c), with

two other scenarios tested for robustness (see Supplementary Note 3, Table S11 and Figure S8). The archive contains logged severities from all agents and is aggregated and classified into the same five-level WDI series, so the simulated and empirical records are compared identically (see Methods).

RC: *Figure 1b-c is intended to illustrate the conceptual idea and approach, but is not easy to follow. An extended caption to walk the reader through the diagram would help. In Figure 1b, I think the person on the left is seeing the grey dots as normal and the person on the right is seeing their grey dot as a drought? It's not clear what the green and orange dots are, or why there are now different green and orange dots in Figure 1c. Perhaps rethink Figure 1c to more clearly show how restandardisation works.*

AR: We have redrawn **Figure 1** and now walk the reader through panels a–d step by step via the legend and an expanded caption. The meaning of the dots is stated explicitly in the legend, and how "shifting baseline syndrome" and "re-standardisation" work have been redesigned and shown in panels **a.** and **c.** more clearly.

RC: *Lines 103-104: r = 0.37 seems like a relatively low coefficient for the comparison between the instrumental record and the proxies. Lines 105-106, not sure which series is being referred to in this sentence, and it's not clear what is meant by "is generally consistent with".*

AR: Done. We have added the context for $r = 0.37$ in the main text and removed the vague phrasing, including:

- The text now explains that this is an annual point-to-point Pearson correlation between a variance-attenuated tree-ring proxy and instrumental precipitation, for which a value around 0.4 is expected, limited by uncertainties in tree-ring reconstruction and explicitly write out what the previously unclear references refer to:

Manuscript · Page 3, Line 105–108

The year-to-year Pearson correlation coefficient between the N-WDI and the instrumental validation data is $r(98) = 0.35$ (two-sided $p < 0.001$; 95% confidence interval 0.16−0.51; n = 100 years; both series consistent with normality, see Methods), which is similar to the collected natural proxy datasets (see Supplementary Information Table S8 and Figure 2a).

RC: *Figure 2 caption also needs to be extended to more clearly indicate what is shown on the figure. Panel 2a: Clarify what the black line and grey uncertainty region represent. What are the dashed horizontal coloured lines? 2b: What are the values shown here −the methods describe the classification of the historical archives into 5 categories, not the extraction of a numerical value? And why are there only three colours when there are five categories? 2c: The open dots showing the locations of the historical data obscure the region of most interest −consider if there is a better way to show this.*

AR: Thanks for your suggestion. We have substantially expanded the Figure 2 caption to cover every panel, including:

- For **2a**, it now states that the black line is the 30-year low-pass reconstruction, the grey band is the 5 − 95% posterior credible interval of the Bayesian-integrated index, and the coloured horizontal dashed lines are the standardised thresholds separating the five WDI categories (SD/MD/N/MW/SW, labelled on the axis).
- For **2b**, it clarifies that the value shown is the regionally aggregated H-WDI, a continuous value aggregated from up to 32 ordinal site records, displayed against the five severity levels and colour-coded dry⟶wet (the panel now uses the full five-level colour scheme, resolving the "only three colours" point).
- For **2c**, it identifies the colour bar ("Pearson r") and shrinks the site markers so they do not obscure the underlying correlation field.

RC: *From Figure 2, it appears that there are distinct 'dry' and 'wet' periods in the record. It would be valuable to show the time series of mismatches as well, as a simple visualisation of whether misclassifications are more likely to appear towards the end of these 'wet' and 'dry' periods.*

AR: We thank the reviewer for this insightful suggestion, which speaks directly to the SBS mechanism, and we have carried it out in full. We (i) plotted the time series of the per-year mismatch, expressed as the categorical offset

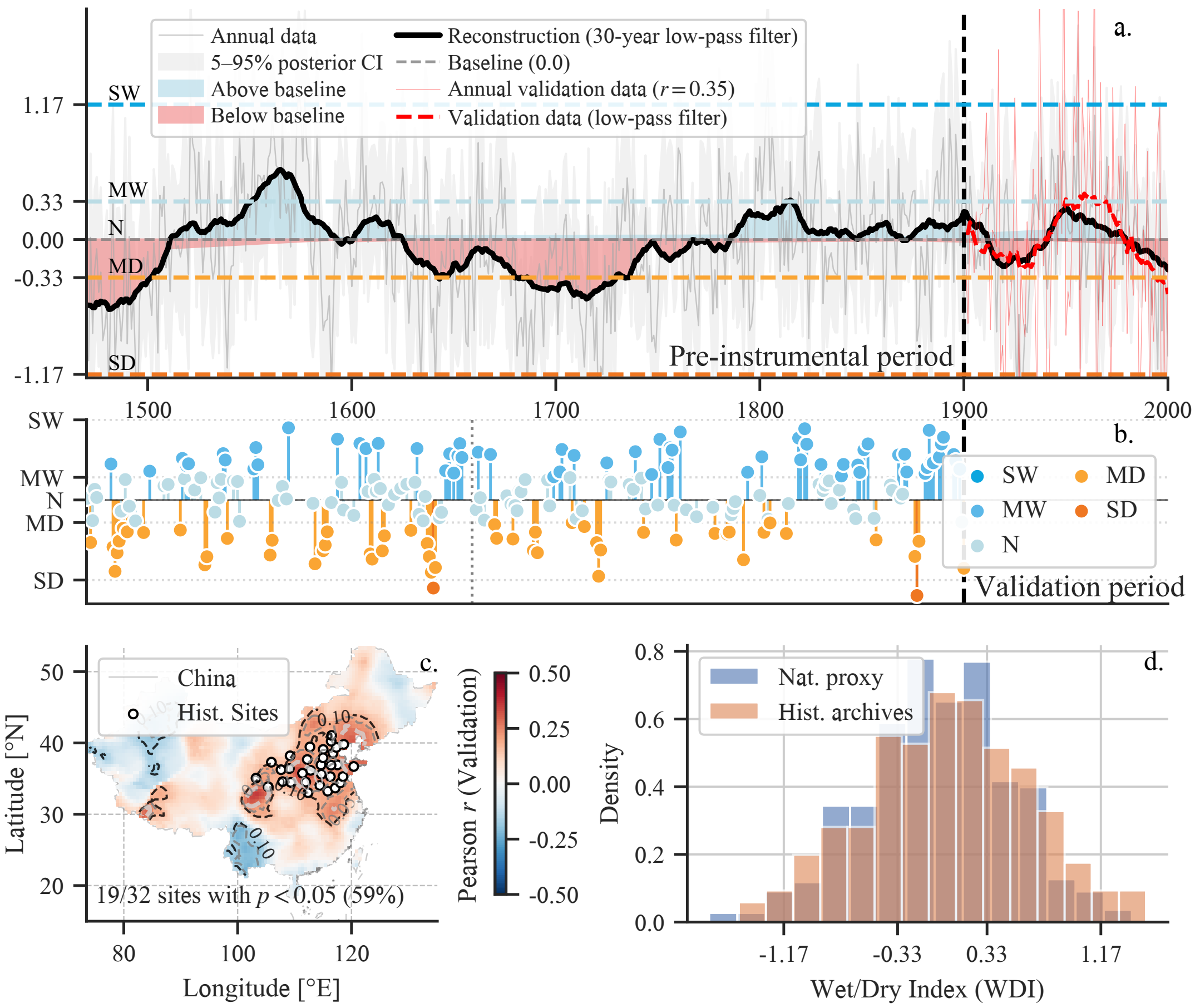

Figure 2: Reconstruction and validation of the WDI datasets. a. Natural-proxy WDI (N-WDI) integrated from tree-ring reconstructions (Methods; Supplementary Note 2). Thin grey line, annual posterior mean; black line, 30-year low-pass filter; grey band, posterior mean ± 1.645 posterior standard deviations (approximate 90% credible interval). Blue and red shading mark years above and below the baseline (z-score = 0); dashed lines mark the thresholds between the five WDI levels (SD, MD, N, MW, SW). Over the validation period (1901–2000 CE), thin and thick red lines show the annual and 30-year low-pass instrumental series ($r = 0.35$). b. Regional historical-archive WDI (H-WDI), the mean of up to 32 site records per year, coloured by level from orange (dry) to blue (wet). c. Pearson correlation between the N-WDI and the gridded instrumental data over the validation period (n = 100 years). Circles mark the 32 archive sites, 19 of which are significant at $p < 0.05$ (two-sided test at the nearest grid cell, not adjusted for multiple comparisons; exact r and p in Supplementary Information Table S2). Dashed contours mark two-sided p = 0.10 (black), 0.05 (grey) and 0.01 (light grey). d. Distributions of the N-WDI (n = 431 years) and H-WDI (n = 208 years) over the pre-instrumental period (two-sided Shapiro–Wilk test: N-WDI $W = 0.998$, $p = 0.812$; H-WDI $W = 0.982$, $p = 0.008$).

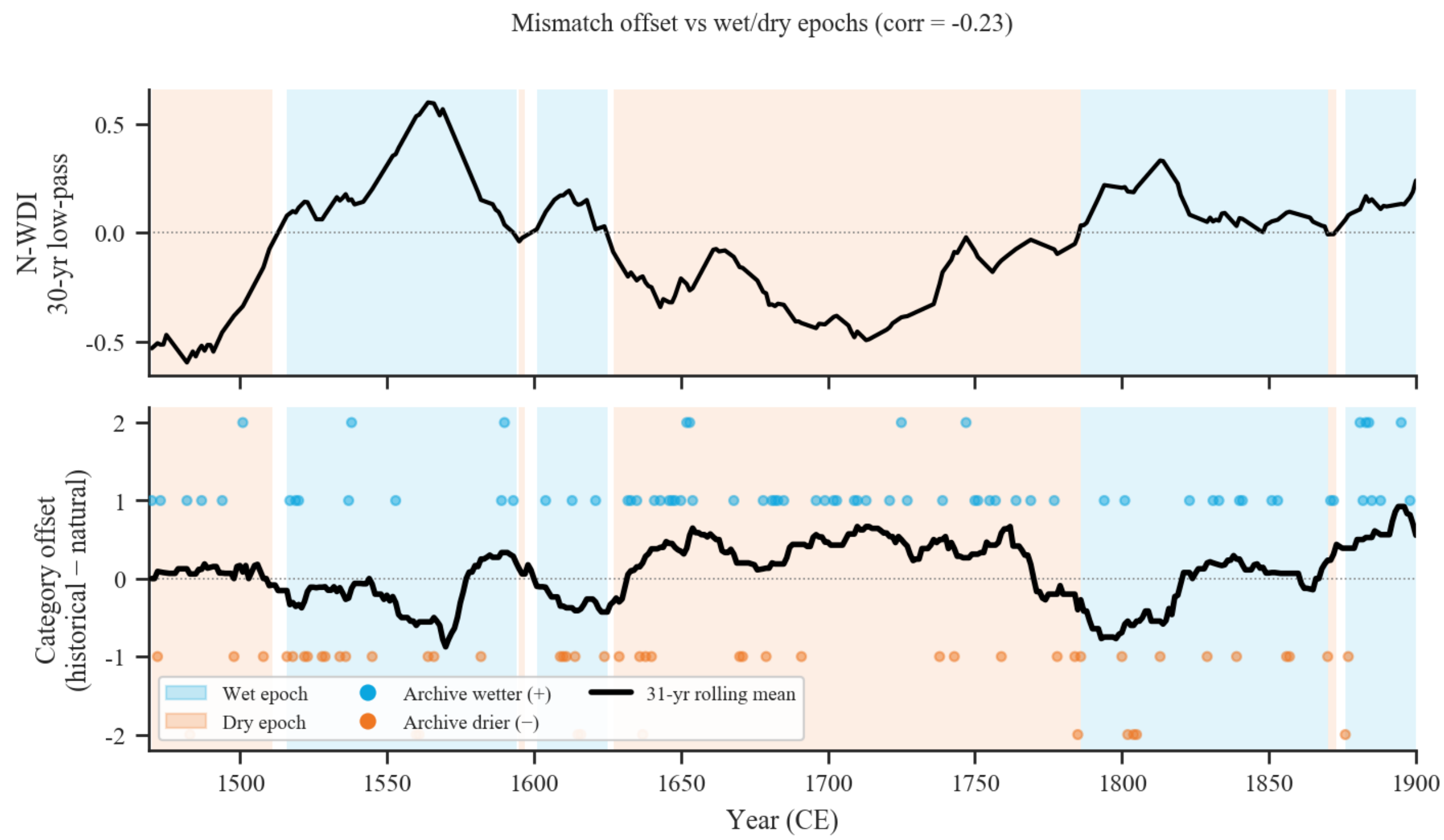

Figure S10: Temporal structure of H-WDI/N-WDI mismatches over 1470 – 1900 CE. Top: the 30-year low-pass N-WDI, used to define sustained wet epochs (blue shading, smoothed trend positive) and dry epochs (orange shading, smoothed trend negative). Bottom: the per-year categorical offset between the archival and natural series (Δ = H-WDI level − N-WDI level); points mark mismatch years where the archive read wetter (blue, $\Delta > 0$) or drier (orange, $\Delta < 0$) than the proxy, and the black line is the 31-year rolling mean of the offset. The annual offset is weakly negatively associated with the smoothed wet/dry state (Pearson's $r = -0.23$, n = 208 years; nominal two-sided $p = 0.001$, not corrected for the serial autocorrelation of the low-pass series and therefore descriptive), i.e. the archive tends to read slightly drier than the proxy during wet epochs and slightly wetter during dry epochs, consistent with a baseline that drifts toward recent prolonged conditions.

between the archival and natural WDI (Δ = H-WDI level − N-WDI level; positive = the archive judges the year wetter than the proxy, negative = drier); and (ii) overlaid the wet/dry epochs, defined exactly as in Figure 2a (the sign of the 30-year low-pass N-WDI: wet where the trend is positive, dry where negative).

According to the figure, two findings emerge:

1. **The direction of the bias tracks the prevailing epoch, as SBS predicts.** The categorical offset is negatively correlated with the low-pass trend (Pearson $r = -0.23$, Spearman −0.23). Within sustained **dry** epochs, the archives systematically over-record wetness (mean offset +0.27; 46 wet-biased vs 20 dry-biased mismatches), whereas within **wet** epochs the bias is weakly reversed (mean offset −0.06). This is precisely the signature of a drifting baseline: inside a prolonged dry epoch, the "normal" reference shifts dry, so genuinely dry years feel ordinary and are under-recorded as dry (i.e. judged wetter than the proxy).

2. **The mismatches do *not* concentrate toward the *end* of epochs**, contrary to the specific timing the reviewer (reasonably) anticipated. Binning each year by its relative position within its epoch (0 = start, 1 = end), the mismatch rate is highest near the start (early 0.71 vs mid 0.55 vs late 0.55; corr(position, $|\Delta|$) = −0.09). In other words, misclassifications cluster just after a wet↔dry transition, when the lagging baseline disagrees most with the newly-established regime —still a lagging-baseline (SBS) phenomenon, but with a transition-onset rather than an epoch-end signature.

Because the epoch-level statistics rest on only six sustained epochs (≥ 10 yr; one of them 159 yr long), the findings are

statistically underpowered, and we are careful not to over-interpret them. We have therefore added these analyses as a **Supplementary Figure** (**Supplementary Note 4, Figure S10**), summarised here, and we do **not** add a panel to the main-text figures, since the within-epoch timing pattern is not clean enough to feature, and the headline mismatch result (the shifted-comparison bias, **Figure 3**) is unchanged.

Methods

RC: *Line 324: Sentence is incomplete.*

AR: Done. The incomplete sentence was the "Due to poor retention rate ... in the early period (1000–1469 CE)." fragment, which lacked a main clause. We have completed it:

> **Manuscript · Page 15, Line 343–346**
>
> Due to the poor retention rate (25.17%, measured as the ratio of archived meteorological data to the total quantity; see Supplementary Note 1) and obvious source bias (mainly based on official histories, with a relative lack of local and folk materials) in the early period (1000 − 1469 CE), we begin our analysis in 1470 CE (see Supplementary Information Figure S2).

RC: *Line 346-347: It is not clear to me what probabilities are being extracted here. And why are the probabilities 10, 25, 30, 25 and 10% ? Ah, I see, these are not probabilities as such, but quantiles.*

AR: Thanks for raising this concern. The reviewer's reading is correct. These are the target category frequencies (quantile widths) used to bin the index, not probabilities in the inferential sense. We have corrected the wording in Methods at both places where the 10/25/30/25/10% appear; they now read:

> **Manuscript · Page 16, Line 396–397**
>
> To maintain consistency with the classification standards in H-WDI, we have chosen the same target category frequencies (quantile widths) for the five levels with same names (see Table 1).

RC: *Line 350: The process of spatial integration is also unclear, and could be made more plain. Why does the WDI for each site need to be randomly simulated? What happens when a site is missing data for that year?*

AR: After each site-year was assigned an ordinal drought–flood grade, we converted grades to continuous z-scores before spatial averaging, because ordinal levels cannot be averaged meaningfully. Each grade maps to a z-interval (not a single point); we therefore drew 100 values per site-year from a truncated normal within that interval and used the mean as the site-level continuous WDI. The regional series is the arithmetic mean across sites with available records in that year. Missing sites are excluded. If there are no site reports, the year is left missing. The regional mean is then reclassified to the five ordinal levels. This stochastic mapping is a tie-breaking convenience for cross-site averaging; a deterministic midpoint mapping yields nearly identical Kendall-τ window curves.

> **Manuscript · Page 15, Line 363–375**
>
> Generally, concrete determinations of floods or droughts, along with descriptions of severe societal outcomes of disasters, are classified at the Severe level (SD or SW), while alone records of natural conditions (such as heavy rains or prolonged droughts) are classified at the Moderate level (MD or MW). In contrast, a normal (N) year refers to records containing keywords such as harvest, abundant year, ripe, and bountiful, or non-abnormal events in the current year, with continuous records available in the preceding or following years (Supplementary Information Table S3 provides more examples). To integrate archives across the study area, site-year ordinal grades were first converted to continuous z-scores, because ordinal levels cannot be averaged meaningfully. Each grade maps to a z-interval bounded by the same thresholds used for WDI classification (rather than a single point, see Table 1). It means, for the site i and year t, we drew 100 values from a truncated normal within that interval and used their mean as the site-level continuous WDI, $h_{i,t}$. The regional H-WDI was then $H_t = \text{mean}(\{h_{i,t}\})$ over all sites with non-missing records in the year t ($n_t \leq 32$). Sites without archival evidence were excluded rather than imputed, and years with no reporting sites were left missing.

This stochastic mapping is a tie-breaking convenience for cross-site averaging.

RC: *Line 358: 'All the original reconstructions were calibrated with correlations above 0.4' –not clear what this means.*

AR: We have rephrased this for clarity. The sentence now states:

Manuscript · Page 16, Line 377–381

The reconstructed datasets are primarily collected from publicly available NOAA repositories (see Supplementary Note 2 for details of data selection and processing). They are mainly based on tree-ring proxies of summer precipitation and Yellow River streamflow in northern China[26], and each original tree-ring reconstruction had achieved, in its source publication, calibration-period correlations with instrumental climate above 0.4 (see Supplementary Note 2, Table S8).

RC: *Line 362: Give a simpler explanation for how these datasets were integrated. Unclear why a different approach for integration is taken for these data than for the historical data, i.e. why not just take means for each year?*

AR: We have rewritten this in plain language and added the rationale for using a Bayesian model rather than a simple average. The natural reconstructions are few and differ in reliability, so we treat each as a noisy observation of one shared latent anomaly, using a Student-t observation model that is robust to outliers. The model then down-weights noisier or lower-skill series and carries each year's spread among the reconstructions into the posterior credible interval, whereas a per-year mean would weight every series equally and discard this uncertainty. The historical archives differ in kind, being ordinal site grades rather than continuous reconstructions, so they are aggregated using the spatial procedure described above (each grade is mapped to a continuous value and averaged across sites).

Manuscript · Page 16, Line 384–392

We then employed a Bayesian latent-variable model to integrate these datasets into a standardised N-WDI from 1470 to 2000 CE. Each year's true anomaly received a weakly informative $\mathcal{N}(0,1)$ prior, matching the unit variance of the standardised inputs, and each reconstruction was modelled as a Student's t observation centred on it. The shared degrees of freedom ν were estimated under a weakly informative Gamma$(2, 0.1)$ prior[27] (posterior mean 36, 94% highest-density interval [18, 58], i.e. close to Gaussian; see Supplementary Note 2 and Table S9). Weighting each series by its local (within-series) rolling variability, the model downweights noisier series and carries their year-to-year disagreement into the posterior credible interval. It was fitted by Markov Chain Monte Carlo (MCMC) in PyMC (version 5.28.4) using four No-U-Turn Sampler (NUTS) chains of 2000 draws each (1000 tuning steps, target acceptance 0.95; all $\hat{R} \leq 1.01$), and posteriors were analysed with ArviZ (version 0.20.0). The N-WDI is the per-year posterior mean; the main results did not change under seven alternative priors (Supplementary Note 2 and Table S10).

RC: *Line 378: Please number equations. Note, these SD thresholds do not appear to give the right quantiles? I think the corresponding quantiles are 12, 37, 63, 88% ?*

AR: We will number all equations. The reviewer is right that $[-1.17, -0.33, 0.33, 1.17]\sigma$ correspond to cumulative probabilities of about 12/37/63/88%, not 10/25/30/25/10%. These thresholds are empirical rather than theoretical quantiles, and we have clarified this in the Methods. The five-grade scheme of the source atlas[25] is defined to reach target grade frequencies of about 10% for the most severe grades, and the thresholds were fitted to the observed frequency distribution of summer precipitation, which is only approximately Gaussian. The 12/37/63/88% the reviewer notes is exactly this small, expected departure of the empirical thresholds from the idealised normal quantiles. We keep the thresholds and the nominal percentages unchanged, and no results change.

Manuscript · Page 15, Line 357–362

All archives related to drought-flood events were then classified into five levels of severity: Severe Dry (SD), Moderate Dry (MD), Normal (N), Moderate Wet (MW) and Severe Wet (SW), with the target frequencies of the five levels approximately controlled at 10%, 25%, 30%, 25%, and 10% (i.e., category quantile widths, see Table

1 and Supplementary Note 1 and Figure S3). This approach, a common practice for inferring Wet/Dry Index (WDI) levels from historical archives[25,28], was applied to each historical site (see Supplementary Information Table S2).

RC: *Section 4.2.2 Bias of mismatches detection. I have read through this methods section several times now, alongside the corresponding results and figure, and I am still having difficulty understanding what is being done here. I understand that we are looking at the years where the historical data and natural proxy data do not match. We then go back through the record to find the most recent year with the same historical value (although it is not clear whether this is numerical value or the classified value). We then determine the difference between the natural proxy values of the two years (delta t, t'). However, it is quite unclear to me what is done with this (delta t, t') value. I cannot understand the results statement 'In these mismatch cases, the severity level from historical archives is usually categorised as drier (or wetter) than those observed in natural proxies when WDI values are only slightly lower (or higher) compared to a previous year. This "shifted-correlation pattern" is most apparent when the shift is 2, in which case the absolute value of these WDI differences is approximately 0.04 on average'. This section and these results need to be made much clearer and plainer. What is the shift? Does WDI in this sentence refer to the historical data or the natural data? Which WDI differences are we talking about? It might be helpful to come up with some clear notation for each set of data being discussed, and use this notation consistently through the text.*

AR: We agree this was the most opaque section and have rewritten it around explicit notation. The revised the corresponding sections in Results and Methods as follows:

- We concretely define the symbols and use them accordingly throughout. For instance, the two datasets are N-WDI and H-WDI. To distinguish continuous and categorised values, we also define N_t and H_t as the five-level N-WDI and H-WDI categories data for the year t, and x_t is the continuous N-WDI value. So, a "mismatch" is a year with $H_t \neq N_t$ and the actual difference of natural climate at this mismatched point is "$\Delta_{t,t'} = x_t - x_{t'}$".
- We now make explicit that the "look-back match" is on the discrete category H, not a continuous value. This means for $t' < t$, there is $N_{t'} = N_t$, where $shift \in \{1, 2, 3\}$ is defined as selecting the $shift$-th most recent earlier year with $H_{t'} = H_t$;
- We now state plainly what the standardized difference $\Delta_{t,t'} = (x_t - x_{t'})/\sigma_{\text{global}}$ measures (how much the *underlying* climate actually differed between two years the archive recorded at the same severity) and how its per-cell average forms the bias matrix that the Monte-Carlo test then evaluates.

Taken together, this whole section's approach now reads like this:

Manuscript · Page 17, Line 422–452

We write N_t and H_t for the five-level (ordinal) categories assigned at year t by the natural proxy (N-WDI) and the historical archive (H-WDI), respectively, and x_t for the underlying continuous N-WDI value. A mismatch is a year where $H_t \neq N_t$. We tested whether mismatches show a systematic signature of comparing the current year to earlier years with the same natural severity category. For each mismatch year t we therefore identified a reference year t' at which the N-WDI had previously been assigned the same category. Formally, t' is the calendar year of the shift times prior observation with the same natural severity category, i.e. $N_{t'} = N_t$, $t' < t$, where the integer shift $\in \{1, 2, 3\}$ denotes the first, second, or third such prior occurrence (Figure 3b). Note that the matching is on the discrete category N, not on a continuous scale. We then asked how much the underlying natural climate had actually changed between these two years sharing the same natural category of N-WDI, quantified as the z-score difference of the continuous N-WDI values x_t and $x_{t'}$ only for the mismatches ($H_t \neq N_t$), i.e., $\Delta_{t,t'} = x_t - x_{t'}$; where x_t and $x_{t'}$ are the continuous N-WDI (natural-proxy) values at the current and reference years. As a control comparison, we also input the severity levels inferred from instrumental data (validation period, 1901 – 2000 CE) as a synthetic historical sequence H_t into the above approach to calculate $\Delta_{t,t'}$, assessing whether the mismatching pattern appears only in historical archives.
A non-zero $\Delta_{t,t'}$ of continuous natural values, therefore, indicates the baseline has drifted and its sign gives the direction (a positive $\Delta_{t,t'}$ indicates the archive over-estimated severity relative to the earlier event, and vice versa). Averaging $\Delta_{t,t'}$ within each cell (i, j) (natural level i against archival level j) yields the observed bias matrix Δ^{obs}_{ij} over mismatch years only. We assessed the statistical significance of this bias pattern with a Monte

Carlo randomisation test that compares the observed bias matrix against a null model in which archival severity categories are assigned independently of the natural climate sequence. The null hypothesis is that archival classifications depend only on their fixed marginal frequencies $P(H = j) = p_j$ ({0.10, 0.25, 0.30, 0.25, 0.10}), with no association between archival grades and the natural series. Each iteration generated a synthetic dataset in which natural climate values $x_{\text{sim}} \sim \mathcal{N}(0, 1)$ were binned into five severity levels using the same thresholds applied to the proxy data, and archival classifications were independently redrawn from p_j. We ran 10,000 iterations, each with a sample size matching the empirical record length ($n = 431$). For each iteration k, the bias matrix $\Delta^{(k)}$, indexed by natural level i and archival level j, was recomputed with the identical look-back algorithm used on the empirical data (the same shift and within-grade pairing $N_{t'} = N_t$, $t' < t$). This null preserves the archival marginals and the look-back mechanics but destroys any dependence between archival assignments and the natural climate sequence. For each matrix cell (i, j), we computed standardized scores $z_{ij} = (\Delta^{\text{obs}}_{ij} - \mu^{\text{null}}_{ij})/\sigma^{\text{null}}_{ij}$, where μ^{null}_{ij} and $\sigma^{\text{null}}_{ij}$ are the mean and standard deviation of $\Delta^{(k)}_{ij}$ across the 10,000 iterations, and obtained two-tailed p-values as $p_{ij} = 2(1 - \Phi(|z_{ij}|))$, which assumes that the null distribution of each cell is approximately normal. These cell-level p values were not adjusted for multiple comparisons. The null simulations are seeded (random seed 42) so that the reported p values are reproducible.

RC: *Figure 3(c-f) I also find opaque. I am not sure what the 'shifted categories' are. An expanded caption that clarifies what is shown would help.*

AR: Sorry about the previous confusion. Figure 3(c–f) now uses the consistent H-WDI/N-WDI notation, and the expanded caption explains the "shifted categories". The revised results based on these panels now read:

Manuscript · Page 5, Line 133–137

To further explore the process of comparing to the past implied by the SBS theory, we compared each mismatched H-WDI data with a similar prior event which occurred once, twice, or three times (shift $= 1, 2, 3$, respectively) in the past (Figure 3b). In these mismatch cases, the H-WDI is usually categorised as drier (or wetter) than N-WDI when the z-score values of N-WDI are only slightly lower (or higher) compared to a former year.

RC: *The re-standardisation approach is somewhat easier to follow. However, I would advocate not using the word 'filter' when referring to re-standardisation –this causes some confusion because of earlier processing steps including 'low-pass' filtering. I would stick with 're-standardised' data.*

AR: Agreed, and this echoes a related comment from another reviewer about the "filter" terminology. We have removed "filter" wherever it refers to re-standardisation and use "re-standardised" consistently, reserving "filter" only for the genuine low-pass step (and for unrelated data-screening).

RC: *Figure 4: Be careful about wording; Figure 4a compares 'original' to 'filtered' data but I think the 'filtered' data here are actually the re-standardised values? Indicate the value of 's' for Figure 4a. Figure 4b, scale needs an additional decimal place. What do Max tau = 0.22 and Avg tau = 0.10 refer to?*

AR: We thank the reviewer and address each point as follows:

- **"filtered" → "re-standardised" (4a).** The reviewer is right: the second series in 4a is the re-standardised one, not a low-pass-filtered one. We have relabelled it "re-standardised" in the figure legend (and use that term consistently, per the related comment above); the caption already refers to "restandardisation ⋯to the original (non-re-standardised) case".
- **Value of "s" in 4a.** The horizontal axis of 4a is the sliding-window size (labelled "Window size, w"); it is swept continuously across the panel, so it is displayed directly along the axis rather than being a single fixed value to annotate. (The symbol "s" was used only for the mismatch "shift", which we kept distinct.)
- **4b "scale".** The two axes of 4b are the sliding-window *parameters* —window size and the minimum-sample requirement which are integer-valued, so additional decimal places on these ticks would not be meaningful. The colour bar (the change in τ) already carries two decimals, so we have made the axis labels and units explicit instead.
- **"Max τ" and "Avg τ" (panel c).** These are averages across the time segments: **"Max τ" is the mean of the**

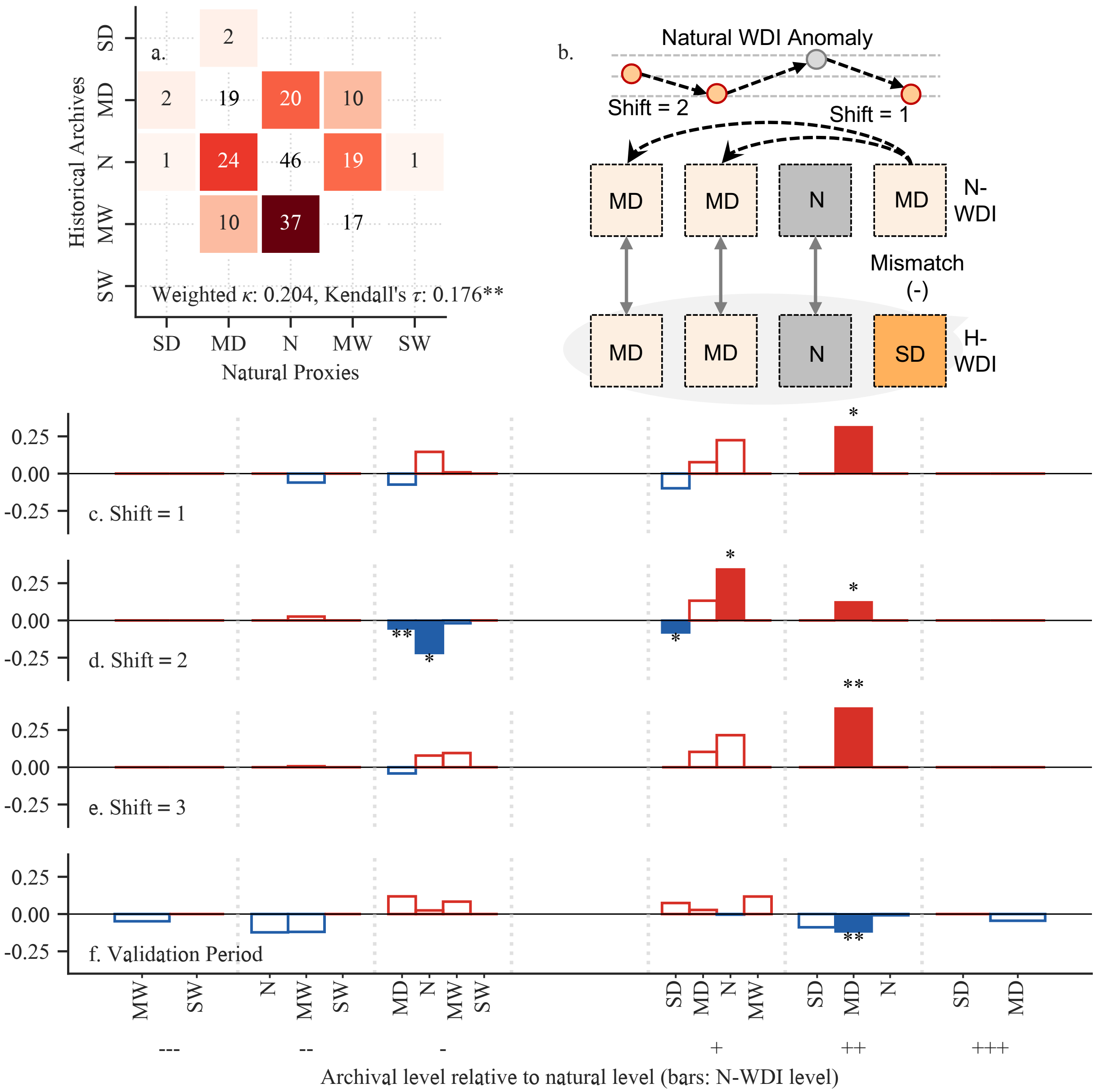

Figure 3: Mismatches between N-WDI and H-WDI levels. a. Confusion matrix of the five WDI levels from natural proxies and historical archives (diagonal, matches; off-diagonal, mismatches; n = 208 years), annotated with Cohen's weighted κ and Kendall's τ (two-sided p = 0.004). b. Shifted comparison: each mismatched archival record (orange box) is compared with the shift-th earlier year in which the N-WDI had the same level (curved dashed arrows), to test whether the misclassification follows the change in N-WDI anomaly between the two years. c.–e. Mean offset of the N-WDI z-score between the current and the earlier year (y-axis) for each type of archival misclassification (x-axis: groups give how many levels wetter (+) or drier (−) the archival level is; bars are labelled with the N-WDI level) at shift = 1, 2 and 3. Significance was assessed by a two-sided Monte Carlo randomisation test (10,000 iterations; Methods), not adjusted for multiple comparisons; filled bars mark $p < 0.05$ (* $p < 0.05$; ** $p < 0.01$). Exact p values (natural to archival level; n, mismatched pairs): c. MD to MW, $p = 0.028$ (n = 10); d. MD to SD, $p = 0.0096$ (n = 2); SD to MD, $p = 0.012$ (n = 1); N to MD, $p = 0.019$ (n = 19); MD to MW, $p = 0.037$ (n = 10); N to MW, $p = 0.047$ (n = 37); e. MD to MW, $p = 0.002$ (n = 10). f. The same analysis for the reconstructed versus instrumental N-WDI over the validation period (1901−2000 CE, n = 100 years, shift = 1); the only cell with $p < 0.05$ (MD to MW, n = 1 pair, $p < 0.001$) has the opposite sign (Supplementary Information Figure S11).

maximum *re-standardised* Kendall's τ (each segment at its own optimal window), and **"Avg τ" is the mean *benchmark* (non-restandardised) Kendall's τ.** Their difference is the improvement in mean correlation. We agree that the bare labels are easy to misread and have spelt this out in the revised caption.

RC: *The length of the optimal restandardisation window looks from Figure 4a and b to be around 15-20 years, yet the text states that the outperformance occurs with windows of 20-40 years, with an optimal sliding window size of 34 years. This suggests that the results from the whole time period contradict the results from shorter time periods. I think it would be simpler and clearer to focus on the results from the whole time period, rather than subsets of the data.*

AR: The apparent contradiction came from mixing the whole-period and per-segment optima without labelling them. We have separated the two in the text and now present the whole-period result as the main line, with the per-segment values kept as a clearly labelled, more local measure. We also note that the rolling-window minimum-sample correction raised by another reviewer slightly shifted the whole-period optimum. After that correction, the mean best window is 33 years, consistent with the 20–40-year band.

RC: *The agent-based modelling approach is also novel and very interesting, but could be made clearer. With the 'collective memory' setting, which years does each agent use as reference? Do they have the collective memory of the full time period, or only access to the collective memory in their own lifespan? (Line 467, memory seems to start at e(t-2) –please correct).*

AR: Thank you. Under the collective baseline (now corresponding to "H2"), each agent references the shared archive accumulated over the full elapsed record, not only the years within its own lifespan. Under the personal baseline (now corresponding to "H1"), each agent references only its own lived experience. We have made this explicit in the Methods:

Manuscript · Page 19, Line 507–509

In the model, there are two types of baselines available for agents comparison: a personal baseline (i.e., μ_i and σ_i are calculated from the agent's own memory M_i) and a collective baseline (i.e., μ_i and σ_i are calculated from the public climate record accumulated over the full elapsed period, $e_1, \ldots, e_{t-1}, e_t$).

To answer the horizon question directly, the source and the temporal span of the baseline are now an explicit part of the design. Besides the personal and full-archive collective baselines reported in the main text, we also tested a collective archive restricted to each agent's own lifespan. This lifespan-windowed collective baseline still does not reproduce the generational optimum and behaves more like the full-archive collective than the personal baseline (see **Supplementary Information Figure S9**), indicating that the generational window arises from individual lived experience rather than a bounded memory horizon alone.

Supplementary Information · Page 22, Line 343–348

The model achieves this by setting up a baseline comparison for each agent before recording. Four baselines are implemented; they are most easily understood as a 2×2 design that crosses two binary factors (Table S11) (i) the source of the remembered baseline (the agent's own experience vs. the shared collective archive) and (ii) its temporal horizon (restricted to a single agent-lifetime, i.e. generational, vs. the full elapsed history, i.e. cumulative):

Table S11: The 2x2 baseline scenario matrix of the ABM, crossing the source of the remembered baseline (the agent's own experience vs the shared collective archive) with its temporal horizon (a single agent-lifetime vs the full elapsed record). The diagonal cells are the two main-text hypotheses (personal, H1 generational amnesia; collective, H2 collective illusion), while collective_lifetime and model are the decomposition and non-perceptual benchmark controls.

	**Generational horizon (one agent-lifetime)**	**Cumulative horizon (full elapsed history)**
Individual source (own memory)	personal —H1: generational amnesia	(idealised limit → model, see below)
Collective source (shared archive)	collective_lifetime	collective —H2: collective illusion

Only the diagonal H1/H2 contrast is foregrounded in the main text, but all four scenarios can be compared at the

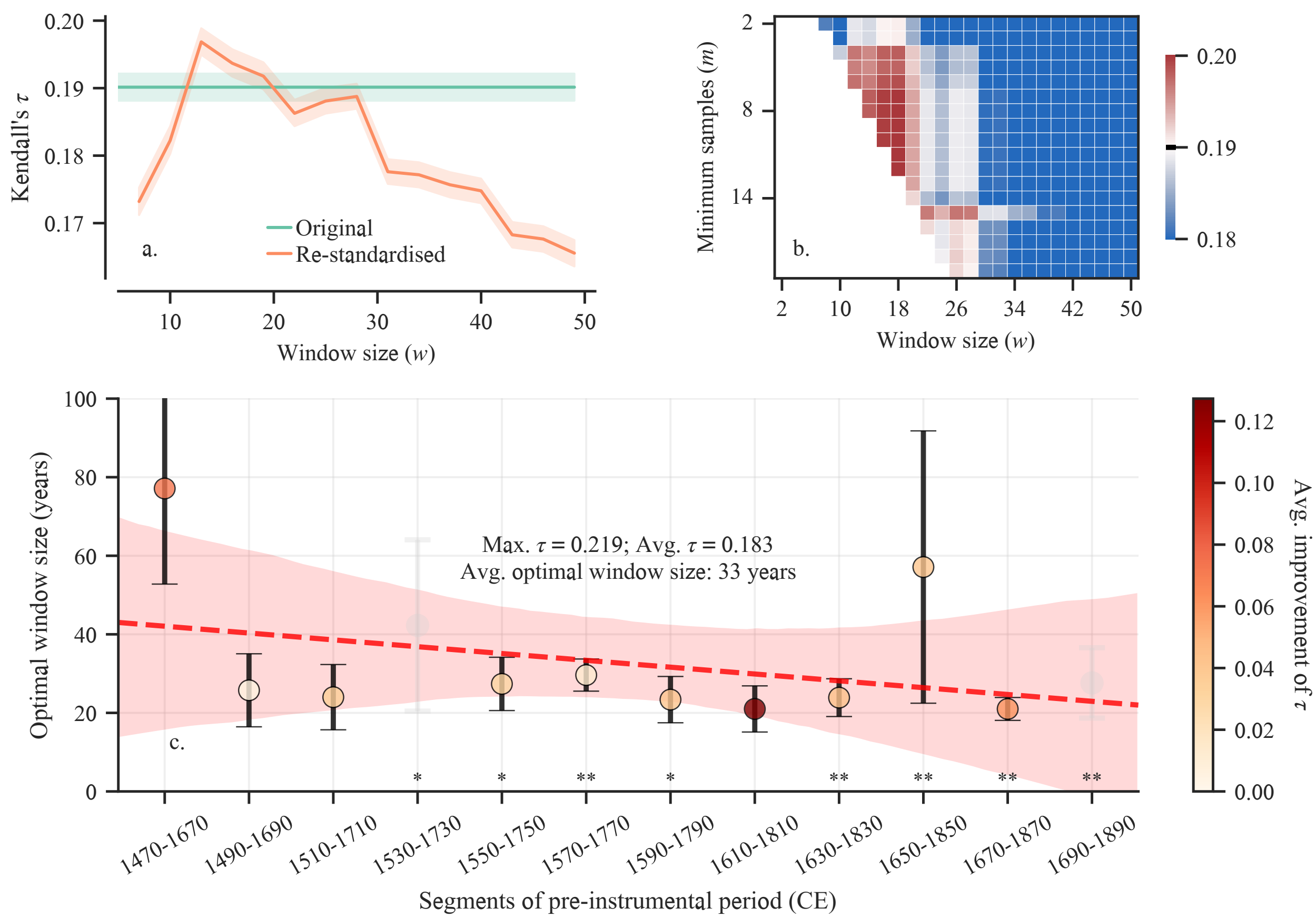

Figure 4: Correlation between the N-WDI and H-WDI under sliding-window re-standardisation. a. Kendall's τ between the H-WDI and the N-WDI with (Re-standardised) and without (Original) re-standardisation of the N-WDI, by window size w. Lines, mean of n = 100 stochastic realisations of the H-WDI (n = 208 years, 205 after re-standardisation); bands, 95% bootstrap confidence interval of the mean (1,000 resamples). b. Kendall's τ across window sizes w and minimum sample sizes m relative to the whole-period benchmark ($\tau = 0.190$, two-sided $p < 0.001$, n = 208 years): red, increase; blue, decrease; blank, $m > w$. All plotted cells are significant (two-sided $p \leq 0.004$); n per cell is the 208 years minus those whose window holds fewer than m values (197 to 207 years). c. Optimal window size in 200-year segments starting every 20 years from 1470 CE. Points and error bars, mean and standard deviation of the windows with the top 10% of τ in each segment (n = 8 windows); colour, mean improvement of τ; transparent points, no improvement; red dashed line and shading, linear trend with its 95% bootstrap confidence interval. Max. τ and Avg. τ, segment means of the maximum re-standardised and the benchmark τ. Asterisks mark segments with a significant benchmark τ (two-sided, not adjusted for multiple comparisons; * $p < 0.05$, ** $p < 0.01$): 1530−1730, $p = 0.013$ (n = 100 years); 1550−1750, $p = 0.027$ (n = 98); 1570−1770, $p = 0.005$ (n = 98); 1590−1790, $p = 0.046$ (n = 99); 1630−1830, $p = 0.005$ (n = 93); 1650−1850, $p = 0.004$ (n = 90); 1670−1870, $p = 0.005$ (n = 90); 1690−1890, $p = 0.004$ (n = 92); not statistically significant: 1470−1670, 1490−1690, 1510−1710 and 1610−1810, p = 0.098, 0.112, 0.093 and 0.088 (n = 101, 99, 103 and 97).

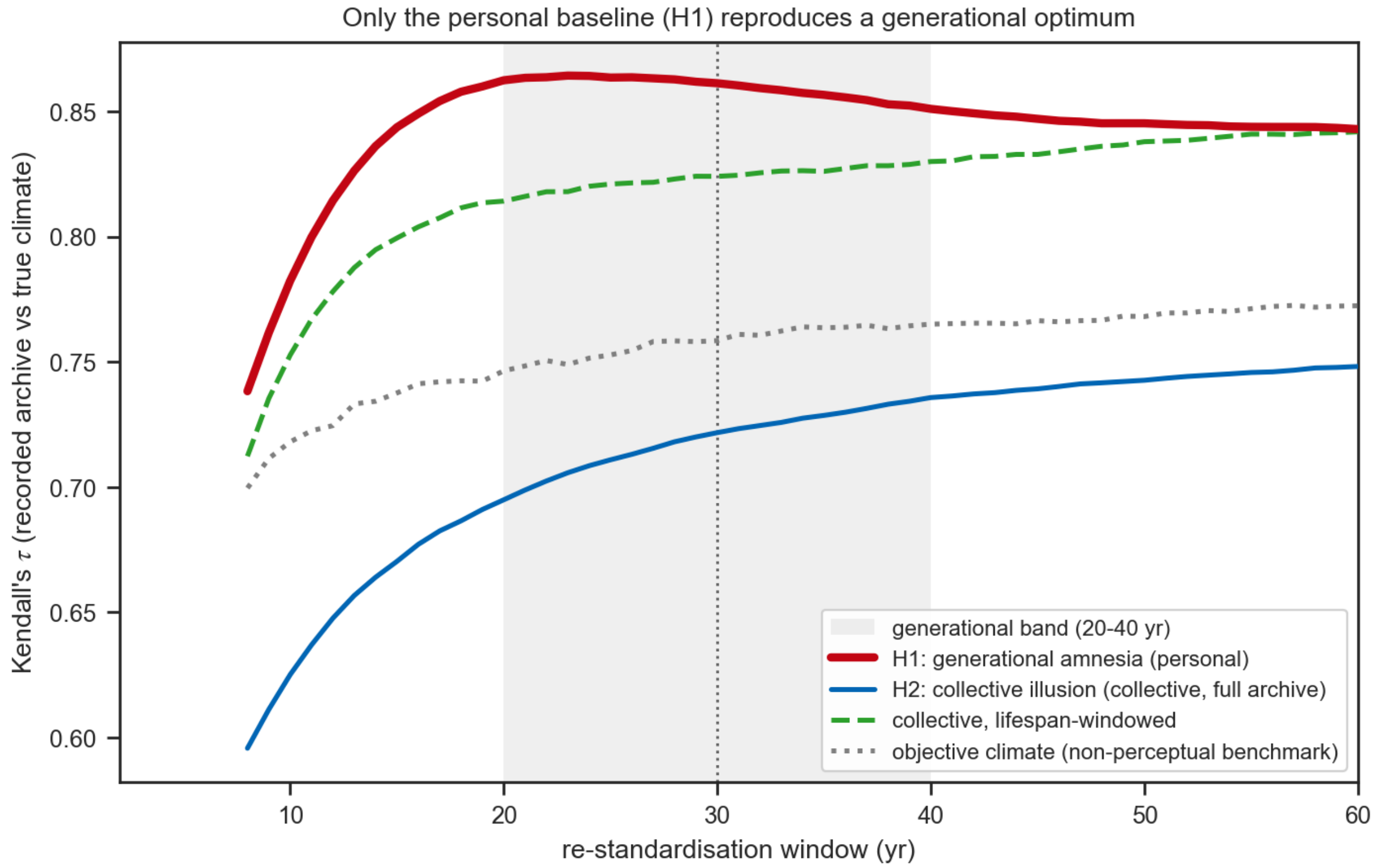

Figure S8: Window−τ correlation profiles for all four baselines of the 2×2 design, each averaged over replicate runs under the AR(1) main forcing (n = 100 runs for H1 and H2, 24 for the lifetime-bounded collective control and 10 for the objective-climate benchmark). Only the personal baseline (H1: generational amnesia, red) peaks within the 20–40-year generational band (shaded; dotted line at 30 yr). The full-archive collective baseline (H2: collective illusion, blue) rises monotonically with window length; the lifetime-bounded collective control (green dashed) tracks H2 rather than H1 despite sharing H1's bounded horizon; and the non-perceptual objective-climate benchmark (grey dotted), referenced to the objective climate, shows no generational peak. The contrast isolates individual lived experience — not a bounded memory horizon per se —as the source of the generational optimum.

Supplementary Information Figure S8:

Supplementary Information · Page 23, Line 367–371

Only the diagonal H1/H2 contrast is foregrounded in the main text; `collective_lifetime` and `model` are reported here as decomposition/benchmark controls, and their full correlation profiles are presented with the robustness analyses below (Figure S8). The empirical basis for the model is derived from analyses of historical climate records from northern China (1470–1900 CE), tree-ring reconstructions (validated for 1901–2000 CE), and the psychological literature on cognitive biases in environmental perception (see main texts).

RC: *The simulations could potentially be more interesting if there are long-term variations in the 'real' climate: at present, climate is a normal distribution with constant mean and sd, but this could be modified by adopting a low-frequency variation in the mean climate, to simulate inter-decadal variations in monsoon patterns.*

AR: We have done exactly this in this revision. The main analysis now uses an AR(1) persistence forcing, and we additionally run a trend-plus-noise forcing that introduces a low-frequency variation in the mean, together with the original i.i.d. case, as a climate-forcing robustness matrix. Across all of these, the emergent optimal window stays within the 20–40-year generational band, so the result is not an artefact of a constant-mean white-noise assumption.

Manuscript · Page 7, Line 168–176

The heuristic ABM represents climate as a stationary AR(1) stochastic process ($c_t = \phi c_{t-1} + \varepsilon_t$, Figure 5a; alternative independent and trended forcings are tested for robustness in Supplementary Note 3 and Figure S9) observed by a continuously turning-over population of finite-lifespan agents. Each agent judges the current anomaly against a baseline, and is more likely to log an event the more it departs from that baseline (Figure 5c). We designed scenarios to represent the baseline, based on lived experience or shared archives of agents, corresponding to the two hypotheses of H1 (generational amnesia) and H2 (collective illusion) (Figure 5c), with two other scenarios tested for robustness (see Supplementary Note 3, Table S11 and Figure S8). The archive contains logged severities from all agents and is aggregated and classified into the same five-level WDI series, so the simulated and empirical records are compared identically (see Methods).

RC: *Figure 5 only shows the outcome of the ABM after re-standardisation with a particular window size. It would be valuable to demonstrate the outcomes before re-standardisation as well, to show more clearly the effect of SBS.*

AR: Because the unrenormalized data showed no clear pattern, we followed the other reviewer's suggestion and included a Sabol sensitivity analysis figure. We hope you will agree that this comprehensive parameter sensitivity analysis better communicates our core conclusion: generational amnesia based on specific max-age (life expectancy) plays a central mechanistic role behind SBS.

RC: *Again, extending the caption to Figure 5 would assist in understanding. I'm not sure exactly what output is shown in panel (b): the label "correlation differences (times of sd)" is not at all clear. Is this the improvement in correlation between 'true' and 'recorded' climate with re-standardisation to a given window size? I don't understand why this is monotonic for the 'collective' memory but peaked for the 'personal' (or 'individual'?) memory.*

AR: The reviewer's interpretation of panel (b) is correct. The correlation improvements between the "true" and "recorded" climate as a function of restandardisation window size. The monotonic-vs-peaked contrast is, in fact, the key result.

- In collective memory, the reference baseline tracks the climate's (autocorrelation) structure, so improvement grows monotonically with window size; in personal memory, the baseline is bounded by agents' finite lifespans, producing an inverted-U that peaks at the generational scale. Our new global sensitivity analysis (revised **Figure 5**) also confirms this: the personal-memory peak location is driven by lifespan, whereas the collective-memory window is driven almost entirely by climate persistence. The revised related results now read:

Manuscript · Page 7, Line 176–181

Reproducing the empirical observation measured in historical archives, the correlations between recorded climate by agents and the input climate forcing show the unimodal pattern at an intermediate generational re-standardisation window under the scenario of H1 (generational amnesia) (Figure 5b, red and black lines). In contrast, the H2 (collective illusion) scenario shows a monotonically increasing trend with window length and no generational optimum (Figure 5b, blue line).

- Regarding why collective memory produces a monotonic curve, our hypothesis is that this precisely reflects a "learning from the past": if we can perceive (through historical archive) farther back, we can correspondingly match perceived climatic changes with actual normalities over longer time scales. Based on this finding, we also briefly stated the importance of "remembering the past as resilience" in the introduction:

Manuscript · Page 2, Line 48–50

Understanding the relationship between collective memory and environmental extremes can thus play a significant role in long-term social resilience and adaptation to hazards[29] (i.e., remembering as a form of resilience[30]).

- and in the discussion:

Manuscript · Page 13, Line 276–279

As collective memory can be purposefully cultivated to enhance resilience[30], it underscores the value of science and history education in establishing correct public awareness of climate change. This is still significant nowadays. Although we have instrument-based measurements and more accurate data, the duration of collective memory can disappear more rapidly[31], with similar decaying patterns across different loss rates[32].

RC: *It would also be useful to discuss how the recording lifespan compares with the timescale on which improvement occurs for the personal memory. The typical recording lifespan is 10-60 years – so why is the timescale for improvement so much shorter? I was expecting a more detailed sensitivity analysis around lifespans, but the sensitivity analysis in the Supplementary is quite cursory – and the relevant Figure S5 is not clearly captioned. Please do ensure that figures in supplementary have full explanatory captions.*

AR: We have substantially expanded the sensitivity analysis exactly as the reviewer hoped: the cursory ANOVA is replaced by a full two-stage global analysis (Morris screening + Sobol decomposition with bootstrap CIs), reported separately for personal and collective memory. This both confirms that lifespan dominates the personal-memory peak:

Manuscript · Page 10, Line 181–187

Global (Sobol) sensitivity analysis identifies which of the five parameters (max age, number of new agents, archive loss rate, climate persistence, and variability; Figure 5d) account for this divergence. Under the scenario H1, the location of the optimal window is dominated by an agent's lifespan (total-effect index $S_T = 1.00 \pm 0.06$, 95% bootstrap confidence interval; Figure 5d) and is directly proportional to it (see Supplementary Information Figure S7). In contrast, if one assumes that agents use the shared archive as the baseline (H2), the simulations did not reproduce the above pattern within the tested parameter ranges (Figure 5d), including when the length of archive access was varied (Supplementary Information Table S11 and Figure S8).

- Regarding the issue of the time scale, we first apologise for the typo in the main text: we actually used 40 years as the value for the max age parameter. We initially guessed that the literate elite could live to 60 years (wrongly) and tested that number, but later found literature supporting ~40 years and forgot to update it (sorry!). In addition, we have now added to the supplementary information a summary of life expectancy based on more extensive sources, which is 40 years. We hope this explanation resolves your concerns about the time scale.

RC: *I took a very brief look at the code. I don't use python, so did not attempt to download and run. However, the directory looked to be well organised and well documented.*

AR: We thank the reviewer for looking at the repository and for the kind words on its organisation and documentation. In this revision, we have additionally fixed the code issues raised by other reviewers and added regression tests, further improving the codebase.

RC: *The manuscript is well written and based on a very interesting combination of two rich and compelling datasets. The interdisciplinary approach, combining tree-ring data with historical records to explore the influence of collective memory on the interpretation of weather events, is particularly intriguing. I found both the introduction and discussion engaging and easy to follow, while the methods and results sections were somewhat harder to navigate due to their technical nature.*

AR: We thank the reviewer for this warm and encouraging assessment, and for highlighting the interdisciplinary combination of tree-ring and historical data as a strength. We have taken the point about the methods and results being harder to follow very seriously. In revision, we introduce consistent notation for the two series, expand the figure captions, and rewrite the most technical passages for accessibility (echoing similar suggestions from other reviewers).

RC: *I also appreciated the examples from the historical sources provided in the supplementary material, especially Table S3. If space allows, I think this table could be moved to the main manuscript, as it conveys the nature of the historical sources, while illustrating how they were interpreted.*

AR: We are glad Table S3 was helpful, and we agree it conveys the texture of the historical sources well. Due to space

constraints, we will not move the full Table S3 into the main text, but we have included one corresponding example for each category when explaining the research methods.

RC: *I am not entirely convinced about the added value of the ABM in its current form. While the idea of comparing individual memory with access to collective memory is interesting, it remains somewhat unclear what additional insights the model provides beyond the empirical comparison between tree-ring data and historical records. I would therefore encourage the authors to clarify more explicitly why the ABM is an integral part of the study.*

AR: We thank the reviewer and agree that we understated this. The empirical comparison establishes that a roughly 30-year re-standardisation window improves the alignment between the archival and the natural WDI, but on its own, it cannot say why that window exists or which mechanism produces it. The SBS literature offers two candidates that are indistinguishable in the archives alone: generational amnesia, in which each generation's baseline is bounded by its own lived experience, and collective-memory illusion, in which shared narratives reshape how earlier conditions are remembered. The ABM is what lets us separate them. Running the same model under a personal (lived-experience) baseline and a collective (shared-archive) baseline, only the personal baseline reproduces the empirical generational optimum, whereas the collective baseline continues to improve toward much longer windows (**Figure 5b**). The global sensitivity analysis traces this difference to its cause: under the personal baseline, the optimal window is set almost entirely by agent lifespan, while under the collective baseline, it merely tracks the climate's own persistence and so cannot produce a generational scale. A collective archive restricted to each agent's lifespan also fails to yield the optimum, indicating that the effect stems from individual experience rather than a bounded memory horizon alone. The ABM's contribution is therefore causal rather than illustrative: it identifies generational amnesia, not collective illusion, as the SBS mechanism consistent with the data, an inference the empirical comparison cannot make on its own. We now state this explicitly in the main text and present the ABM as a mechanism-discrimination test rather than a fitted predictor.

> **Manuscript · Page 10, Line 187–189**
>
> This heuristic model suggests that generational amnesia (assuming individuals rely on their lived experience) is sufficient to generate generational inconsistencies in the model, whereas collective illusion did not reproduce the observed mismatch pattern in the empirical WDIs.

To reaffirm the importance of ABM, we made many changes to the manuscript, including revised **Figures 1 and 5** and an entirely revised **Section 2.4**. Relevant citations can be found in the reviewers' comments in our response and in the revised main text.

RC: *I also wonder how sensitive ABM was to different remembering ages of individuals? Was the model stress-tested to assess whether the results were robust across different age assumptions? Also, was Lmax set to 40 or 60 years? There seems to be a discrepancy between the manuscript and the supplementary information.*

AR: Yes, the model has now been stress-tested along exactly this axis. We replaced the earlier ANOVA with a global, variance-based Sobol sensitivity analysis, sampling agent lifespan ($L_{\max}$) across the full 15 to 80-year range, well beyond any single plausible value. Two results follow. First, the peak-window location varies smoothly and monotonically with lifespan and stays within the generational band throughout, so the optimum is not a knife-edge that holds only at one age assumption. This dependence is itself the mechanism, since finite memory is what generates the window, rather than a fragility. Second, within that range lifespan is by far the dominant driver of the peak-window location under the personal-memory baseline, accounting for about 94% of the variance (first-order $S_1 \approx 0.94$, total-effect $S_T \approx 1.00$), while recruitment, archive loss, and the climate's variability and persistence together contribute less than 6% (each total-effect below 0.06). The roughly 30-year optimum that matches the archives is therefore robust to the other assumptions and arises, to first order, from the historically appropriate lifespan alone.

> **Manuscript · Page 12, Line 265–269**
>
> Finally, the ABM is a deliberately minimal, heuristic model rather than a demographic reconstruction. This is mainly because the groups that produced the historical archives do not represent the entire population, and our global sensitivity analysis (Figure 5d) shows that their life expectancy matters. We have confirmed the key parameter in this simplified demography is consistent with population data we have collected (Supplementary

Information Figure S6).

On the discrepancy the reviewer caught, the intended default is $L_{\max} = 40$ years, the value used throughout the code, the run configurations, and the Supplementary Information. The stray $L_{\max} = 60$ in the Methods was a typographical error, now corrected to 40 so that the main text, the **Supplementary Information**, and the released code all agree.

RC: *The manuscript uses objective vs subjective when referring to the two datasets, where the tree-ring data is considered objective and the historical archives subjective. This distinction becomes somewhat reductive, since neither dataset is entirely straightforward. Tree-ring records are also interpreted proxy data, shaped by sampling, methodological choices, and assumptions about environmental responses. At the same time, historical archives should not simply be treated as "subjective". I think this distinction could simply be removed.*

AR: We fully agree, and we have adopted this suggestion throughout. We have:

- removed the "objective vs subjective" dichotomy from the text and figures and replaced it with two neutral, descriptive labels (the historical-archive WDI: H-WDI and the natural-proxy WDI: N-WDI) and used them consistently. (As a side benefit, this neutral labelling also implements the consistent two-series notation requested by Reviewer #2.)
- no longer described the tree-ring series as "objective", but state explicitly that it too is an interpreted proxy shaped by sampling and methodological choices; what distinguishes it is only that it does not pass through human memory, making it a relatively independent, non-perceptual reference, whereas the archival series is mediated by contemporaneous perception and is therefore comparatively more exposed to perceptual bias. This relative asymmetry, rather than an absolute objective/subjective split, is what our analysis actually relies on. The corresponding sentences in the main text now read:

Manuscript · Page 14, Line 322–327

We treat the natural-proxy WDI (N-WDI) reconstructed from tree-ring data as a relatively independent, non-perceptual reference for climatic anomalies. It is independent not because it is free of interpretation (it, too, is a reconstructed proxy shaped by sampling and methodological choices), but because it does not pass through human memory and is compared with the historical-archive WDI (H-WDI), estimated from documentary records and mediated by contemporaneous perception. The differences and mismatched patterns between the two, therefore, can reflect the perceptual biases behind historical archives as a carrier of collective memory.

RC: *Figure 2d: how can there be more than five WDI index categories for the historical archival material?*

AR: The site-level archival records are five ordinal categories; the apparent "more than five" values in Figure 2d arise because the panel shows the regional H-WDI, which is the mean of up to 32 site-level ordinal records and is therefore continuous. We now state this explicitly in the Figure 2 caption.

RC: *Lines 107–109 state that over 0.8 of the historical archive sites were located in areas showing a positive correlation with the WDI series. What exactly does this mean? Does 0.8 refer to 80% of sites, or something else?*

AR: The reviewer is right that the bare "0.8" was ambiguous. We have removed it and now report the spatial agreement as a percentage of sites. In the same revision, we also dropped the $p < 0.1$ threshold (at Reviewer #3's request), so the sentence now reads in terms of the conventional $p < 0.05$ level: **19 of the 32 historical-archive sites (59%)** lie in areas significantly positively correlated with the N-WDI series ($p < 0.05$). The **Figure 2c** annotation and caption already report this same 60% / $p < 0.05$:

Manuscript · Page 5, Line 113–114

Spatially, 19 of the 32 historical-archive sites we used were located in areas that showed a significant positive correlation with the N-WDI series (two-sided test of Pearson's r at the nearest grid cell, $n = 100$ years, not adjusted for multiple comparisons; site locations are listed in Supplementary Information Table S2; Figure 2c; other validation datasets in Supplementary Information Figure S4)

RC: *Lines 323–325 appear to form an incomplete sentence.*

AR: This is the same fragment flagged by Reviewer #5. The dangling "Due to poor retention rate … in the early period (1000–1469 CE)." sentence (which had no main clause) now reads:

> **Manuscript · Page 15, Line 343–346**
>
> Due to the poor retention rate (25.17%, measured as the ratio of archived meteorological data to the total quantity; see Supplementary Note 1) and obvious source bias (mainly based on official histories, with a relative lack of local and folk materials) in the early period (1000 – 1469 CE), we begin our analysis in 1470 CE (see Supplementary Information Figure S2).

RC: *The figure labels are formatted inconsistently throughout the manuscript. In some figures the panels are marked as "a", while in others they are written as "a.". They are also positioned differently between figures, for example in the right vs left side of the panels.*

AR: We have adjusted the subfigure labels for all figures, but because each figure has a different layout, placing them all in the same position does not seem feasible; we have done our best to make the layout neater.

RC: *In Figure 2, the text "Pearson correlation coefficient" also appears to be somewhat awkwardly positioned.*

AR: Done. In the revised Figure 2 the awkward free-floating "Pearson correlation coefficient" text has been replaced by a clean vertical colour-bar label, "Pearson r (Validation)", placed alongside panel 2c, and the caption now identifies it explicitly.

RC: *My overall assessment is that this is a very exciting and promising manuscript that could become even stronger with some minor revisions.*

AR: We are grateful for this positive overall assessment and for the constructive, focused suggestions. We have addressed each of the points above, and we believe the revised manuscript is clearer and stronger as a result.

1. Papworth, S. k., Rist, J., Coad, L. & Milner-Gulland, E. j. Evidence for shifting baseline syndrome in conservation. *Conservation Letters* **2**, 93–100 (2009).
2. Pauly, D. Anecdotes and the shifting baseline syndrome of fisheries. *Trends in Ecology & Evolution* **10**, 430 (1995).
3. Fritsche, I. & Masson, T. Collective climate action: When do people turn into collective environmental agents? *Current Opinion in Psychology* **42**, 114–119 (2021).
4. Soga, M. & Gaston, K. J. Shifting baseline syndrome: Causes, consequences, and implications. *Frontiers in Ecology and the Environment* **16**, 222–230 (2018).
5. Soga, M. & Gaston, K. J. Global synthesis indicates widespread occurrence of shifting baseline syndrome. *BioScience* **74**, 686–694 (2024).
6. Rozin, P. & Royzman, E. B. Negativity Bias, Negativity Dominance, and Contagion. *Personality and Social Psychology Review* **5**, 296–320 (2001).
7. de Guttry, C. & Ratter, B. Expiry date of a disaster: Memory anchoring and the storm surge 1962 in Hamburg, Germany. *International Journal of Disaster Risk Reduction* **70**, 102719 (2022).
8. O'Connor, P. The unanchored past: Three modes of collective memory. *Memory Studies* **15**, 634–649 (2022).
9. Gongaware, T. B. Collective Memory Anchors: Collective Identity and Continuity in Social Movements. *Sociological Focus* **43**, 214–239 (2010).
10. Ge, J. *History of Chinese Population.* (Fudan University Press, Shanghai, 2005).
11. Barclay, G. W., Coale, A. J., Stoto, M. A. & Trussell, T. J. A reassessment of the demography of traditional rural China. *Population Index* **42**, 606–635 (1976).
12. Lee, J. Z. & Campbell, C. D. *Fate and Fortune in Rural China: Social Organization and Population Behavior in Liaoning, 1774–1873.* (Cambridge University Press, 1997). doi:10.1017/cbo9780511563058.
13. Lee, J. Z., Feng, W. & Campbell, C. D. Infant and child mortality among the Qing nobility: Implications for two types of positive check. *Population Studies* **48**, 395–411 (1994).
14. Campbell, C. D. & Lee, J. Z. Long-term mortality consequences of childhood family context in Liaoning, China, 1749–1909. *Social Science & Medicine* **68**, 1641–1648 (2009).

15. Telford, T. A. Patching the holes in Chinese genealogies: Mortality in the lineage populations of Tongcheng county, 1300–1880. *Late Imperial China* **11**, 116–136 (1990).
16. Riley, J. C. *Rising Life Expectancy: A Global History.* (Cambridge University Press, 2001). doi:10.1017/cbo9781316036495.
17. Sherif, M., Taub, D. & Hovland, C. I. Assimilation and contrast effects of anchoring stimuli on judgments. *Journal of Experimental Psychology* **55**, 150–155 (1958).
18. Gray, E., Grasso, M., Farrall, S., Jennings, W. & Hay, C. Political Socialization, Worry about Crime and Antisocial Behaviour: An Analysis of Age, Period and Cohort Effects. *The British Journal of Criminology* **59**, 435–460 (2019).
19. Fanta, V., Šálek, M. & Sklenicka, P. How long do floods throughout the millennium remain in the collective memory? *Nature Communications* **10**, 1105 (2019).
20. Fang, X. *et al. The Social Impacts of Climate Change in China Over the Past 2000 Years.* (Springer Nature, 2024).
21. Song, S. *et al.* Improving representation of collective memory in socio-hydrological models and new insights into flood risk management. *Journal of Flood Risk Management* **14**, (2021).
22. Roberts, P. *et al.* Uncovering the Multibiome Environmental and Earth System Legacies of Past Human Societies. *Annual Review of Environment and Resources* (2024) doi:10.1146/annurev-environ-112321-101257.
23. Song, S., Wang, S., Jiao, C. & Ibarra, E. J. M. ABSESpy: An agent-based modeling framework for social-ecological systems. *Journal of Open Source Software* **9**, 6298 (2024).
24. Cohen, J. Weighted kappa: Nominal scale agreement provision for scaled disagreement or partial credit. *Psychological Bulletin* **70**, 213–220 (1968).
25. Yang, Y. *Millennium regional Extreme Drought and flood Atlas of China.* (China Cartographic Society, 2024).
26. Liu, Y. *et al.* Recent anthropogenic curtailing of Yellow River runoff and sediment load is unprecedented over the past 500 y. *Proceedings of the National Academy of Sciences* **117**, 18251–18257 (2020).
27. Juárez, M. A. & Steel, M. F. J. Model-Based Clustering of Non-Gaussian Panel Data Based on Skew-t Distributions. *Journal of Business & Economic Statistics* **28**, 52–66 (2010).
28. Yi, L. *et al.* Reconstructions of annual summer precipitation and temperature in north-central China since 1470 AD based on drought/flood index and tree-ring records. *Climatic Change* **110**, 469–498 (2012).
29. Hoffman, S. M. Chapter 17 - Culture: The Crucial Factor in Hazard, Risk, and Disaster Recovery: The Anthropological Perspective. in *Hazards, Risks, and Disasters in Society* (eds Shroder, J. F., Collins, A. E., Jones, S., Manyena, B. & Jayawickrama, J.) 289–305 (Academic Press, Boston, 2015). doi:10.1016/B978-0-12-396451-9.00017-2.
30. Garde-Hansen, J., McEwen, L., Holmes, A. & Jones, O. Sustainable flood memory: Remembering as resilience. *Memory Studies* **10**, 384–405 (2017).
31. García-Gavilanes, R., Mollgaard, A., Tsvetkova, M. & Yasseri, T. The memory remains: Understanding collective memory in the digital age. *Science Advances* **3**, e1602368 (2017).
32. Candia, C., Jara-Figueroa, C., Rodriguez-Sickert, C., Barabasi, A.-L. & Hidalgo, C. A. The universal decay of collective memory and attention. *Nature Human Behaviour* **3**, 82–91 (2019).
